# Estimating the evidence of selection and the reliability of inference in unigenic evolution

**DOI:** 10.1186/1748-7188-5-35

**Published:** 2010-11-08

**Authors:** Andrew D Fernandes, Benjamin P Kleinstiver, David R Edgell, Lindi M Wahl, Gregory B Gloor

**Affiliations:** 1 Department of Biochemistry, The University of Western Ontario, N6A 5C1 Canada; 2 Department of Applied Mathematics, The University of Western Ontario, N6A 5B7 Canada

## Abstract

**Background:**

Unigenic evolution is a large-scale mutagenesis experiment used to identify residues that are potentially important for protein function. Both currently-used methods for the analysis of unigenic evolution data analyze 'windows' of contiguous sites, a strategy that increases statistical power but incorrectly assumes that functionally-critical sites are contiguous. In addition, both methods require the questionable assumption of asymptotically-large sample size due to the presumption of approximate normality.

**Results:**

We develop a novel approach, termed the Evidence of Selection (EoS), removing the assumption that functionally important sites are adjacent in sequence and and explicitly modelling the effects of limited sample-size. Precise statistical derivations show that the EoS score can be easily interpreted as an expected log-odds-ratio between two competing hypotheses, namely, the hypothetical presence or absence of functional selection for a given site. Using the EoS score, we then develop selection criteria by which functionally-important yet non-adjacent sites can be identified. An approximate power analysis is also developed to estimate the reliability of inference given the data. We validate and demonstrate the the practical utility of our method by analysis of the homing endonuclease I-Bmol, comparing our predictions with the results of existing methods.

**Conclusions:**

Our method is able to assess both the evidence of selection at individual amino acid sites and estimate the reliability of those inferences. Experimental validation with I-Bmol proves its utility to identify functionally-important residues of poorly characterized proteins, demonstrating increased sensitivity over previous methods without loss of specificity. With the ability to guide the selection of precise experimental mutagenesis conditions, our method helps make unigenic analysis a more broadly applicable technique with which to probe protein function.

**Availability:**

Software to compute, plot, and summarize EoS data is available as an open-source package called 'unigenic' for the 'R' programming language at http://www.fernandes.org/txp/article/13/an-analytical-framework-for-unigenic-evolution.

## Background

One of the principal reasons for studying molecular evolution is that the function of a novel protein can be deduced, in part, by comparing it with a similar previously-characterized protein. But what recourse do we have if the novel protein does not exhibit significant sequence similarity to other proteins? More problematically, what if it is similar only to proteins of unknown function? In practice, even when the novel protein shares regions of extensive similarity to proteins of *known *function, it may be difficult to elucidate the importance of individual sites in the novel protein.

### Unigenic Evolution

One innovative experimental approach that can help identify specific domains or residues required for function is *unigenic evolution*, first described and developed by Deminoff *et al*. [1]. Unigenic evolution can be applied to any protein where the loss of function can be used as a selectable phenotype [1-5].

The procedure consists of random mutagenesis and amplification of a single wild-type sequence via mutagenic polymerase chain reaction (PCR) with subsequent cloning and functional selection [6]. Functional clones are isolated and characterized by DNA sequencing. In contrast to traditional structure-based mutagenesis screening, unigenic evolution experiments produce an *unbiased *estimate of functionally-important residues regardless of putative structural role or conservation.

### Deminoff's Analysis

The selection process ensures that, in functional clones, amino acids essential for function will be conserved relative to non-essential sites. However, differential mutation sensitivity can be caused by more than structural or functional constraints. Mutation rates of residues may differ due to differential transition/transversion rates, codon usage, and genetic code degeneracy. To correct for these confounding factors, Deminoff *et al*. developed a statistical analysis that compared the expected versus the observed mutation frequency for each codon, where the expected frequencies were derived from a population of clones that had not been subject to selection.

Deminoff *et al*. clearly demonstrated the importance of accounting for non-uniform transition versus transversion probabilities when computing expected mutational frequencies. To increase the inferential power of their analyses, they also developed a 'sliding-window' *χ*^2^-analysis, binning together a 'window' of adjacent codons, assuming that residues critical for protein function would be nearby in primary structure. By comparing the probabilities of *silent *versus *missense *mutation in these windows, regions of either restrained or excessive mutability were identified as hypo- or hyper-mutable, respectively.

### Behrsin's Analysis

The subsequent analysis of Behrsin *et al*. [7] advanced the statistical framework of Deminoff *et al*. by improving three key features. These features were (a) the fixed window size of the *χ*^2^-analysis, and (b) the effect of sample-size on the codon mutation probability, and (c) accounting for multiple nucleotide mutations per codon. First, window size for the *χ*^2^-analysis was addressed by using windows of different sizes and comparing estimated false-discovery rates. The 'best' window was selected via tradeoff between the estimated sensitivity and specificity for classifying hypo- or hyper-mutable residues. Second, nucleotide substitution frequencies were computed using the continuity correction of Yates [8] resulting in more consistent codon mutation frequencies. Third, codon mutation frequencies were computed analytically from nucleotide substitution frequencies without the assumption that only one substitution per codon was likely.

### Further Improvements

The statistical framework of Deminoff *et al*. and the modifications suggested by Behrsin *et al*. allow for the reliable identification of hypo-mutable regions via unigenic evolution. Nonetheless, these state-of-the-art analyses suffer from some deficiencies, from a statistical perspective, that could result in either erroneous or misleading conclusions. The goal of this work is to develop a statistically rigorous method for the analysis of unigenic evolution data, improving upon existing techniques by

1. relaxing the assumption that sample sizes are large enough such that asymptotic normality necessarily applies,

2. relaxing the assumption that selection-sensitive regions of a protein are contiguous,

3. clarifying the relationship between Fisher-style *p*-values and Neyman-Pearson Type-I and Type-II error probabilities with regard to testing hypotheses of functional selection,

4. relaxing the the assumption that the PCR amplification protocol does not meaningfully affect mutation probabilities, and

5. addressing the ability to of unigenic evolution to detect hyper-mutability.

We expand upon each of these points, in turn, below.

First, both Deminoff *et al*. and Behrsin *et al*. equate observed event *relative counts *with the respective event *probabilities*. This equivalence is effectively true when either sample sizes are asymptotically large or probabilities are non-extreme (not too close to either zero or one). However, experimentally-feasible sample sizes are typically limited to the order of 50-100 replicates (clones) and even the most mutagenic of PCR conditions result in low probabilities (≈ 0.001 to 0.01) of point mutation. Therefore it is unlikely that observed counts have a simple relationship with the event frequency, even accounting for the continuity correction of Yates [8]. The difficulty of estimating probability parameters from event-counts when the likelihood of the event is very small is a well-known problem from the inference of binomial and multinomial frequency parameters [9]. The most obvious consequence of assuming "counts ≈ probabilities" under these constraints is that the normal approximation, on which the *χ*^2 ^statistic is critically dependent, may be invalid enough to yield misleading results. At the very least, the sampling variance of the *χ*^2 ^statistic itself is necessarily quite large. The anticipated parameter ranges above, for example, yield a coefficient of variation for *χ*^2 ^to be on the order of 100-300%. An additional problem with equating counts and probabilities is that, in doing so, the analysis of Behrsin *et al*. implicitly conditions on the total number of mutations as given. We anticipate that the actual number of mutations would be roughly Poisson distributed, implying that the variance of the mutation count will be on the order of the expected count itself, further degrading the validity (or at least power) of the *χ*^2 ^statistic to correctly determine the effect of selection.

Second, the assumption that selection-sensitive regions of a protein are contiguous is incorrect because proteins are three-dimensional amino acid chains where secondary, tertiary, and quaternary folds bring distant-in-sequence residues to three-dimensional proximity. Perhaps the most widely known example of functionally-important yet non-contiguous sites is the catalytic triad of residues in serine proteases such as trypsin [10,11]. Trypsin proteins have three absolutely required residues that form a charge-relay system needed for activity; with respect to the human sequence these are H57, D102, and S195. These three residues are widely separated in sequence, but are adjacent in the 3 D fold of the protein. Furthermore, S195 is surrounded by a conserved set of residues, but H57 and D102 are not. The residues surrounding the H58 and D102 equivalents in other organisms are very different and are drawn from all classes of amino acids. Thus using a 'windowing' procedure to identify hypo-mutable regions would fail in the H57 and D102 instances since many different amino acids are tolerated adjacent to an absolutely conserved position in a protein family. If selection-sensitive residues *cannot *be presumed contiguous, it is unclear how sites can be partitioned into 'selected' and 'non-selected' groups while correcting for implicit and combinatorially-increasing number of multiple comparisons.

Third, the use of a Fisher-style hypothesis test to compute a *p*-value directly is *not *equivalent to the estimation of the Neyman-Pearson Type-I and Type-II error probabilities *α *and *β*, respectively [12]. Although often confused in the literature, *p *and *α *are not interchangeable. Specifically, Fisher's *p*-value expresses the probability of the observed and more extreme data given the null hypothesis, and can be considered a random variable whose distribution is uniform on (0, 1) under that null. In contrast, the Neyman-Pearson *α *and *β *values directly compare the probability of the observed data given either the null or alternate hypotheses as correct. The value of *α *must be fixed *before *the observations are made and is subject to the minimization of *β*. Confusion between *α *and *p *results in the systematic *exaggeration *of evidence against the null hypothesis [13-17].

Fourth, current unigenic evolution analyses treat the mutagenic PCR protocol as a 'black box' process that takes a wild-type sequence as input and produces a pool of mutagenized clones as output. However, the physical process of mutagenic PCR via non-proofreading *Taq *polymerase imposes significant constraints on the amplification process in turn constrains properties of the final clone population. We show herein that the ultimate probabilities of the different classes of codon mutation are complicated, nonlinear functions of the *Taq* misincorporation probabilities. Given the complicated relationship between *Taq* misincorporation frequencies and codon mutation frequencies, it is important to determine if the PCR protocol meaningfully constrains observed codon mutation frequencies.

Last, the claim that unigenic evolution can detect hyper-mutability follows the fact that critical values of the *χ*^2 ^statistic can be observed due to either too few *or too many *observed mutation events. However, mutagenic PCR of a nucleotide sequence is an anisotropic 'random drift' through sequence-space. Selection can *only *act on the drifting sequence by 'slowing' its progression along trajectories that realize less-functional mutants, since these less-functional sequences are preferentially discarded. Neither mutagenic PCR nor functional selection are capable of 'accelerating' the sequence drift, thus implying that a 'large' number of observed mutations at a given site, while improbable, are not unexpected. We therefore claim that unigenic evolution is fundamentally incapable of detecting hyper-mutability, positing that unexpectedly-large site-mutation counts stem from either ordinary sampling variance, or non-modelled systematic, procedural, or other experimental errors.

The statistical framework described herein provides estimates of both the evidence of selection, and the statistical power available to detect that selection, independently for *each *codon site. It provides explicit comparisons with internal positive and negative controls, thus reducing the impact of systematic or experimental errors. It identifies *individual sites *rather than *broad regions *for follow-up analysis, and can guide wild-type sequence optimization with regard to unigenic mutability. With its emphasis on analytical rigour and its availability as an easy-to-use software add-in package, this work helps to make the analysis of saturating-mutagenesis experiments both statistically sound and broadly accessible.

## Results

To implement the aforementioned improvements we have developed a new method for analyzing data from unigenic evolution experiments. The analytical framework we have developed is uniquely powerful because it has no assumption of normality or other asymptotic approximations; has no requirement that critical residues be contiguous; provides detailed estimates of Type-I and Type-II errors; explicitly models the relationship between polymerase misincorporation probabilities and PCR mutation probabilities; and easily adapts to different codon compositions, nucleotide biases, and genetic codes. Our method, including subroutines for data visualization and summarization, was implemented as a package called unigenic as part of the R statistical software system [18] and is available under an open-source license.

### Overall Procedure

Like previous methods, our procedure begins by estimating the 'background' nucleotide mutation frequencies given by the mutagenic PCR on a control population of clones that are *not *subject to functional selection. This population, termed the 'unselected' population, serves as a control group that describes expected mutation frequencies under the null hypotheses of 'no functional selection'. The *nucleotide *mutation frequencies of the unselected clones are used to compute synonymous and nonsynonymous mutation probabilities for the *codons *of the wild-type sequence under the null hypothesis. Finally, the observed number of synonymous and nonsynonymous mutations occurring at each codon of the functionally-selected clones are tested to see whether they are more concordant with the null hypothesis or a generalized alternative.

The overall procedure of using nucleotide mutation frequencies to estimate codon mutation frequencies was first suggested by Deminoff *et al*. who argued that adequate statistical power could not be realized if only codon-triplet mutations were analyzed. During the development of our method, we tested the hypothesis that synonymous and nonsynonymous mutation counts alone have sufficient power to resolve functional versus nonfunctional proteins. Our test used a site-by-site test of multinomial homogeneity as described by Wolpert [19]. A test of multinomial homogeneity in our context is a test that, given the count of synonymous and nonsynonymous mutations at a particular site in both the selected and unselected populations, asks if the the counts are compatible with the hypothesis that the mutation frequencies are equal. This test is unique in that it does not require inferring or comparing frequencies directly. Instead, *all *possible frequencies that are compatible with the data are considered simultaneously. We found, for the experimental system described below, that codon mutation frequencies alone are *insufficient *to discriminate between populations. Results of the homogeneity tests are shown in Additional File 1. This lack of ability to discriminate populations underlies the necessity of formulating a null hypothesis via nucleotide mutation frequencies, and confirms the supposition of Deminoff *et al*..

#### Experimental System

Complete details of the experimental system and data analyzed in conjunction with the development of our method are presented in Kleinstiver *et al*. [20]. Briey, our experimental system analyzed the 266 amino acid GIY-YIG homing endonuclease I-Bmol[21-23], a site-specific DNA endonuclease consisting of an N-terminal catalytic domain connected by a linker region to a C-terminal DNA-binding domain. The N-terminal domain of ≈ 90 amino acids contains the class-defining GIY-YIG motif that is highly conserved in almost all the members of this endonuclease family. I-Bmol binds to a ≈ 38 bp recognition sequence (the homing site) and introduces a double-stranded DNA break leaving a single-stranded 2-nucleotide 3'-overhang. We took advantage of the site-specific endonuclease activity of I-Bmol in the genetic selection to isolate functional variants after 30 cycles of mutagenic PCR. The genetic selection utilizes a chloramphenicol resistance plasmid (pExp) to express wild-type I-Bmol or mutant variants, and a second ampicillin resistant compatible plasmid (pTox) that contains the I-Bmol homing site. pTox also encodes a DNA gyrase toxin under the control of an inducible arabinose promoter. Cells that contain both plasmids only survive plating under selective conditions if I-Bmol is functional and can cleave pTox. Cleavage of pTox by I-Bmol generates a linear plasmid that is rapidly degraded, thus removing the gyrase toxin and promoting cell survival. If I-Bmol is non-functional and pTox is not linearized, cells will not survive due to the activity of the gyrase toxin. Under non-selective conditions, all cells will survive regardless of whether I-Bmol is functional because there is no requirement to cleave the toxic plasmid. Using this genetic selection, we introduced random point-mutations in the I-Bmol coding region by mutagenic PCR, and isolated and sequenced 87 functional 'selected' clones after plating on selective media. We also isolated and sequenced 87 clones isolated on non-selective media resulting in a pool of 'unselected' clones in order to establish base-line mutagenesis frequencies.

#### PCR Misincorporation Frequencies

The first step in estimating the nucleotide misincorporation frequencies requires tabulation of observed nucleotide mutations in the unselected population into the 4 × 4 matrix *C*, where *c_ij _*represents the number of observed misincorporations from nucleotide *j *in the wild-type sequence to clone-type nucleotide *i*. Experimental nucleotide mutation counts for our I-Bmol system are presented in Table 1. Note that under even highly-mutagenic conditions, *C *is strongly diagonally-dominant, indicating that even under highly-mutagenic conditions misincorporations are still relatively rare. The mutation frequency matrix *P *can be computed from *C *by normalizing each column of *C *to sum to one. Doing so, in effect, equates misincorporation counts with relative frequencies. This simple normalization suffers from two drawbacks, however. First, the equivalence of mutation frequencies with relative counts is only true when the product (Σ*_k _c_kj_*)*p_ij _*is sufficiently large for all *i *and *j*, due to the same reasoning which underlies the approximation of the binomial with a normal distribution [24]. Second, this normalization results in only a point-estimate of *P *and provides no information about the accuracy of the estimate. Given the low frequency of many mutation events, such as the single observed {C ← G} event per 11 223 total {any ← G} events as shown in Table 1, it is doubtful that the condition of 'sufficiently large' holds.

**Table 1:** Sample Misincorporation Counts Under *H*_0_

	Unselected Clone Counts				Selected Clone Counts			
-	**A**	**C**	**G**	**T**	**A**	**C**	**G**	**T**
A	23 656	18	27	182	24 024	11	12	80
C	65	18 045	1	184	24	18 058	1	72
G	282	4	11 176	29	154	0	11 203	9
T	270	29	19	15 439	71	27	7	15 673

**Total**	24 273	18 096	11 223	15 834	24 273	18 096	11 223	15 834

Sample mutagenic PCR counts *C *for 30 cycles of mutagenic PCR on the 798-nucleotide wild-type sequence for the I-Bmol homing endonuclease, as sequenced from 87 clones. Matrix entry *C_ij _*represents the number of observed misincorporations from wild-type nucleotide *j *to clone-type nucleotide *i *in the 'unselected' population (top). For comparative purposes, misincorporation counts from the 'selected' population are also presented (bottom) although these are not used to estimate parameter matrix *T*.

To remedy these difficulties, the columns of *P *were presumed to be multinomial probabilities of a 'black-box' mutagenic process. Given an 'input' wild-type nucleotide *j*, column *j *of *P *gives the multinomial probabilities of the resultant 'output' clonal nucleotide. Under the hypothesis that the output nucleotide depends *only *on the input nucleotide, each of the four columns of *P *describe four, independent multinomial distributions.

Estimating multinomial parameters from observed event counts is a well-studied subject. When any (Σ*_k _c_kj_*)*p_ij _*is small, as is typical in unigenic evolution data, techniques for multinomial estimation have been thoroughly investigated under various Bayesian frameworks. Following numerous recommendations in the literature [25], each column of *P *is assumed to be Dirichlet-distributed such that our null hypothesis asserts that

(1) $$H_{0}:P_{*j}\sim \text{Dirichlet(}C_{*j}+\alpha )\;\text{for each given wild-type nucleotide}\;j,$$

where *α *is a vector of hyperparameters with each component set to 1/2. The justification and derivation of (1) are detailed under the 'Methods' subsection 'Multinomial Estimation'. Equation (1) defines *P *as a standard linear Markov operator. Let the four-dimensional vector *x_j _*denote the frequencies of the wild-type nucleotides A, C, G, or T at site *j*. In general the wild-type sequence will not display polymorphism, implying that *x_j _*is equal to one of [1, 0, 0, 0], [0, 1, 0, 0], [0, 0, 1, 0], or [0, 0, 0, 1]. The action of *P *on *x_j _*, given by standard matrix-vector multiplication, results in *y_j _*= *P_xj _*representing the frequency of nucleotides expected in site *j *within the unselected-clone population.

#### Modelling the Polymerase

The main difficulty with accepting the hypothesis that the columns of *P *describe *independent *multinomial processes is that this hypothesis is not concordant with inspection of observed data. Deminoff *et al*. specifically noted correlated differences in mutation events that were attributed to differences between transition and transversion processes. Behrsin *et al*. observed ostensibly the same phenomenon, noting that complementary misincorporation events, such as {C ← A} and {G ← T}, always had similar counts [[7], Table 1]. Again, this similarity was attributed to differences between the mutagenic mechanisms leading to transition and transversion.

Our observed counts, shown in Table 1, showed a similar pattern. However the reduction to frequencies, as shown in Table 2, suggested the hypothesis that $p_{ij}≃p_{\tilde{i}\tilde{j}}$, where $\tilde{i}$ and $\tilde{j}$ denote the complements of nucleotides *i *and *j*, respectively. Of the sixteen misincorporations described by *P*, twelve describe mutations. Differential mutation between transition and transversion can only explain differences between the four types of transition-mutations and the eight types of transversion-mutation. By itself, such a mechanism is incapable of explaining similarities between complementary-base mutations *within *each of these two classes. Preliminary computational modelling suggested that the similarities between *p_ij _*and $p_{\tilde{i}\tilde{j}}$ could be explained by modelling the *overall *mutagenic PCR process as the culmination of multiple cycles of error-prone DNA synthesis. Errors in synthesis are presumed to occur via nucleotide misincorporation by *Taq* polymerase under conditions optimized for mutation [6].

**Table 2:** Estimates for the Expected Nucleotide Mutation Frequency.

	Relative Count				Natural Parameter				Maximum *a posteriori*			
-	**A**	**C**	**G**	**T**	**A**	**C**	**G**	**T**	**A**	**C**	**G**	**T**
A	97.46	0.10	0.24	1.15	97.46	0.10	0.24	1.15	97.47	0.13	0.20	1.14
C	0.27	99.72	0.01	1.16	0.27	99.72	0.01	1.16	0.24	99.66	0.02	1.17
G	1.16	0.02	99.58	0.18	1.16	0.02	99.58	0.18	1.16	0.02	99.65	0.23
T	1.11	0.16	0.17	97.51	1.11	0.16	0.17	97.51	1.12	0.19	0.13	97.46

Three estimates for the expected frequency of nucleotide mutation *P *as a function of the counts *C *in Table 1 given as **relative counts **with *p_ij _*= *C_ij_/*Σ*_i _C_ij _*, **natural parameter **means as per equation (13), and **maximum *a posteriori ***estimates when constrained by the polymerase misincorporation model with frequencies *T*. The relative-count and natural-parameter frequencies are virtually identical, while the polymerase model-constrained estimates has minor deviation. The significance of differences is difficult to discern via inspection. However, small variances between the modelled values are magnified geometrically by the relationship between nucleotide and codon mutation frequencies, as per Additional File 2, and exponentially by the action of PCR amplification. Note similarities between *p_ij _*and $p_{\tilde{i}\;\tilde{j}}$ for all estimates, where $\tilde{i}$ and $\;\tilde{j}$ denote the complements of nucleotides *i *and *j*, respectively.

Error-prone nucleotide incorporation by *Taq* polymerase can be modelled if we let *τ_ij _*denote the relative frequency that the polymerase incorporates nucleotide *i *against template-nucleotide *j*. Collecting these misincorporation frequencies into matrix *T*, we compute the PCR mutation frequencies *P *as a function of the polymerase misincorporation frequencies *T *resulting in the secondary null hypothesis

(2) $${H^{\prime }}_{0}:P=P(T).$$

We denote (2) as a 'secondary' null hypothesis since *T *is estimated using only the unselected clone counts *C*, thereby still representing the hypothesis of 'no functional selection'. A detailed description of the model underlying ${H^{\prime }}_{0}$ and the computational challenges associated with computing the posterior distribution of *P *as a function of *T *are discussed under the 'Methods' subsection 'Modelling PCR via Polymerase'. Again, *P *under ${H^{\prime }}_{0}$ is defined such that it too is a linear Markov operator. Frequencies *P *inferred under ${H^{\prime }}_{0}$ are subtly different than those derived under *H*_0 _or via relative counts and are shown in Table 2. Although appearing small, the significance of these differences is difficult to discern by inspection for two reasons. First, codon mutation frequencies under the null hypothesis are computed as the product of three nucleotide mutation frequencies. The effect of small differences among the *p_ij _*parameters is therefore geometrically amplified with respect to codons. Second, the PCR process is a nonlinear, exponential amplification of misincorporation rates *T*, implying that small changes in *T *can result in large changes in both nucleotide and codon mutation frequencies.

#### Codon Mutation Frequencies

Unigenic evolution presumes that selection operates on protein function and not during transcription or translation. Differences in protein function are caused by nonsynonymous amino acid substitution. Therefore the frequencies of nonsynonymous mutation need to be computed from the given frequencies of independent nucleotide mutation. Although Behrsin et al. provide a number of different sample formulas for deriving codon mutation frequencies given nucleotide frequencies, we describe a generalized method of deriving these frequencies for two reasons. First, our framework immediately accommodates different genetic codes, such as mitochondrial or chloroplast. Second, we require ready generalization, beyond the two classes of synonymous or nonsynonymous mutation currently used, for future work.

A codon comprises three contiguous nucleotides within a given reading frame. Since *P *is assumed to act *independently *on nucleotides, the mutagenic PCR process for codons is concisely represented by

(3) M=P⊗P⊗P,

where *M *is a 64 × 64 linear Markov operator that operates on the space of codon frequencies and '⊗' denotes the standard Kronecker matrix-product. An explicit depiction of the Kronecker product and how it relates nucleotide mutation to amino acid mutation is shown in Additional File 2. As with nucleotides, given wild-type codon frequencies *w_j _*at codon-site *j*, the quantity *z_j _*= *Mw_j _*represents the frequency of site-*j *clone codons after mutagenic PCR under the null hypothesis.

The columns of *M *describe the probabilities that a codon subject to mutagenic PCR will remain identical, mutate synonymously, or mutate nonsynonymously. For example, consider column AGA of *M*. The standard genetic code translates AGA to arginine, as do the five additional codons CGT, AGG, CGA, CGG, and CGC. Therefore, given AGA as the wild-type codon, *M *describes the probability of either no mutation (identity) or synonymous mutation as

(4) $$\begin{array}{cc} p_{\text{sn}}=\sum_{i}M_{i,AGA} & \\ & \text{for }i\in \{AGA,\text{ CGT},\text{ AGG},\text{ CGA},\text{ CGG},\text{ CGC}\}, \end{array}$$

and the probability of nonsynonymous mutation as *p*_ns _= 1 - *p*_sn_. Such matrix partitioning is simple to code in languages supporting named-index array-slicing, such as R [18]. It is also simple to adapt the required bookkeeping to any desired genetic code. For computational efficiency and ease of notation, we denote *p*_sn,*j *_and *p*_ns,*j *_to be the probabilities of synonymous and nonsynonymous mutation at codon *j*, using the subscripts to clearly differentiate them from the entries of nucleotide mutation matrix *P*, above.

#### Unit of Analysis

Rather than using only the two classes of synonymous and nonsynonymous mutation, it is *theoretically *possible to compare the observed counts for all 20 amino acids at each codon site. Such a comparison reduces *M *to a 20 × 64 matrix mapping codons to amino acids. Preliminary analyses of the ≈ 100 clones sequenced in each of our selected and unselected populations, however, showed insufficient power to make meaningful inferences at the amino acid level.

Amino acids or synonymous/nonsynonymous mutation are not the only possible units of analysis, however. For example, the assumption that selection operates at the protein level implies that the functional assay is independent of transcription or translation efficiency. Future work could easily test this hypothesis by reducing *M *to the three classes of 'identical', 'synonymous but not identical', and 'nonsynonymous'. Possibly, even different classes of codons could be defined. The main tradeoff with using more classes for analysis is the requirement for larger sample sizes. However, the basic hypothesis-testing framework described below could be used with only trivial modification.

### Alternate Hypothesis

The analysis of a unigenic evolution experiment compares observed site-specific mutation counts between two populations. The first population, the control group, is *not *subject to selection; these are the 'unselected' clones and they provide the values of *p*_sn,*j *_and *p*_ns,*j *_under the null hypothesis.

The second population *is *subject to functional selection and results in a set of 'selected' clones. The frequency of mutation at each codon of the selected population provides an alternate hypothesis that can be compared with expectations under the null.

For codon-site *j *we denote $n_{\text{sn},j}^{\text{us}}$ and $n_{\text{ns},j}^{\text{us}}$ to be the respective number of observed synonymous and nonsynonymous mutations in the unselected population, and $n_{\text{sn},j}^{\text{mx}}$ and $n_{\text{ns},j}^{\text{mx}}$ to be the respective number of observed synonymous and nonsynonymous mutations in the selected population. The total number of clones sequenced from each pool is therefore $n^{\text{us}}=n_{\text{sn},j}^{\text{us}}+n_{\text{ns},j}^{\text{us}}$ and $n^{\text{mx}}=n_{\text{sn},j}^{\text{mx}}+n_{\text{ns},j}^{\text{mx}}$, both of which are constant for all *j*.

#### Likelihood Model

A standard multinomial likelihood model is used to describe the probability of mutation given the total number of clones sequenced and the presumed frequency of mutation. For each codon-site *j*, this gives

(5) $$\begin{array}{cc} \mathrm{Pr}\left(n_{\text{sn},j}^{\text{us}},n_{\text{ns},j}^{\text{us}}|n^{\text{us}},H\right) & \\ & =\left(\frac{n^{\text{us}}!}{n_{\text{sn},j}^{\text{us}}!n_{\text{ns},j}^{\text{us}}!}\right){\left(p_{\text{sn},j}^{\text{us}}\right)}^{n_{\text{sn},j}^{\text{us}}}{\left(p_{\text{ns},j}^{\text{us}}\right)}^{n_{\text{ns},j}^{\text{us}}} \end{array}$$

and

(6) $$\begin{array}{cc} \mathrm{Pr}\left(n_{\text{sn},j}^{\text{mx}},n_{\text{ns},j}^{\text{mx}}|n^{\text{mx}},H\right) & \\ & =\left(\frac{n^{\text{mx}}!}{n_{\text{sn},j}^{\text{mx}}!n_{\text{ns},j}^{\text{mx}}!}\right){\left(p_{\text{sn},j}^{\text{mx}}\right)}^{n_{\text{sn},j}^{\text{mx}}}{\left(p_{\text{ns},j}^{\text{mx}}\right)}^{n_{\text{ns},j}^{\text{mx}}}, \end{array}$$

with codon mutations presumed to be mutually independent. The conditional hypothesis *H *dictates the origin of the probability parameters $p_{\text{sn},j}^{\text{us}}$, $p_{\text{ns},j}^{\text{us}}$, $p_{\text{sn},j}^{\text{mx}}$, and $p_{\text{ns},j}^{\text{mx}}$. Under the null *H *= *H*_0 _these parameters are computed via nucleotide counts *C *using (1). Under the secondary null $H={H^{\prime }}_{0}$ they are computed via polymerase misincorporation frequencies *T *using (2). Under the alternate hypothesis *H *= *H_A_*, they are inferred using only the site-specific counts $n_{\text{sn},j}^{\text{us}}$, $n_{\text{ns},j}^{\text{us}}$, $n_{\text{sn},j}^{\text{mx}}$ and $n_{\text{ns},j}^{\text{mx}}$, respectively, theoretically accommodating *any *type of selection mechanism.

Therefore under *H_A _*the distribution of parameter-pair $\left[p_{\text{sn},j}^{k},p_{\text{ns},j}^{k}\right]$, for *k *∈ {us, mx}, is

(7) $$\left[p_{\text{sn},j}^{k},p_{\text{ns},j}^{k}\right]∼\text{Dirichlet}\left(n_{\text{sn},j}^{k},n_{\text{ns},j}^{k}+\alpha \right),$$

as discussed previously. Again, *α *is a vector of hyperparameters with each component set to 1/2 and the justification and derivation of (7) are detailed under the 'Methods' subsection 'Multinomial Estimation'.

Note that since parameters inferred under *H_A _*encompass arbitrary types of selection, they necessarily overlap with parameters under the null. However, under *H_A _*parameters $p_{\text{sn},j}^{k}$ and $p_{\text{ns},j}^{k}$ are relatively diffuse since they are estimated from ≈100 sequenced clones. Under *H*_0 _or ${H^{\prime }}_{0}$ these same parameters are estimated by ≈ 20 000 nucleotide misincorporations and are thus more precisely determined.

Lastly, we test both selected and unselected populations for consistency between hypotheses since doing so treats the unselected population as a negative control. This helps identify possible systematic or experimental errors during cloning or sequencing, thereby reducing false signals of selection.

#### Evidence of Selection

Given prior probabilities Pr(*H_A_*) and Pr(*H*_0_) that either *H_A _*or *H*_0 _is true for site *j*, Bayes' Theorem in conjunction with standard likelihood ratios can be used to compute

(8) $$\begin{array}{cc} \frac{\mathrm{Pr}\left(n_{\text{sn},j}^{k},n_{\text{ns},j}^{k}|n^{k},p_{\text{sn},j}^{k},p_{\text{ns},j}^{k},H_{A}\right)\mathrm{Pr}\left(H_{A}\right)}{\mathrm{Pr}\left(n_{\text{sn},j}^{k},n_{\text{ns},j}^{k}|n^{k},p_{\text{sn},j}^{k},p_{\text{ns},j}^{k},H_{0}\right)\mathrm{Pr}\left(H_{0}\right)} & \\ & =\frac{\mathrm{Pr}\left(\;H_{A}|n_{\text{sn},j}^{k},\;n_{\text{ns},j}^{k}|n^{k},\;p_{\text{sn},j}^{k},\;p_{\text{ns},j}^{k}\right)}{\mathrm{Pr}\left(\;H_{0}|n_{\text{sn},j}^{k},\;n_{\text{ns},j}^{k}|n^{k},\;p_{\text{sn},j}^{k},\;p_{\text{ns},j}^{k}\right)}, \end{array}$$

the relative probabilities of the hypotheses at codon *j*, given the data *and *parameter values. UsuallyPr(*H_A_*) and Pr(*H*_0_) are set equal to each other in the absence of other information. However, parameter values are not known precisely but have posterior distributions. Taking logarithms of (8) and integrating over these posteriors results in

$$R_{j}^{k}=\iint \log\left(\tilde{R}\right)d{\left[p_{\text{sn},j}^{k},\;p_{\text{ns},j}^{k}\right]}_{H_{A}}d{\left[p_{\text{sn},j}^{k},\;p_{\text{ns},j}^{k}\right]}_{H_{0}}$$

where

$$\tilde{R}=\frac{\mathrm{Pr}\left(n_{\text{sn},j}^{k},n_{\text{ns},j}^{k}|n^{k},p_{\text{sn},j}^{k},p_{\text{ns},j}^{k},H_{A}\right)\mathrm{Pr}(H_{A})}{\mathrm{Pr}\left(n_{\text{sn},j}^{k},n_{\text{ns},j}^{k}|n^{k},p_{\text{sn},j}^{k},p_{\text{ns},j}^{k},H_{0}\right)\mathrm{Pr}(H_{0})}$$

giving

(9) $$R_{j}^{k}=\log\left[\frac{\mathrm{Pr}\left(H_{A}|n_{\text{sn},j}^{k},n_{\text{ns},j}^{k}\right)}{\mathrm{Pr}\left(H_{0}|n_{\text{sn},j}^{k},n_{\text{ns},j}^{k}\right)}\right],$$

the expected log-odds-ratio of *H_A _*versus *H*_0 _given the number of observed mutations for codon *j *in clone-population *k*. We call $R_{j}^{k}$ the Evidence of Selection (EoS) for codon *j *in clone-population *k*.

**Figure 1:**
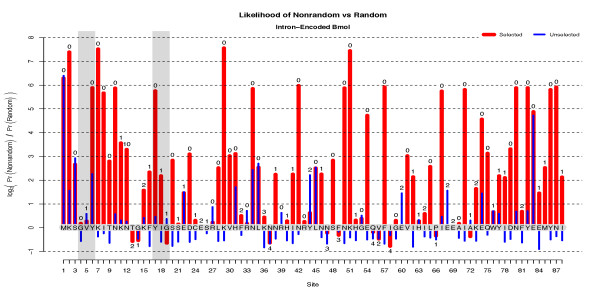
**The Evidence of Selection for ****I-Bmol**. Posterior log_2_-odds-ratio EoS score $R_{j}^{\text{mx}}$ for the first 88 amino acid sites of I-Bmol for both selected (red) and unselected (blue) clone populations. The wild-type amino acid sequence is given along the abscissa. The total number of nonsynonymous mutations for that site in the selected population are indicated over the red bars. In this context 'nonrandom' indicates the alternate hypothesis while 'random' indicates expectations under the null. Note that the M1 start-codon is always preserved as an artifact of ligation during cloning. Thus M1 is a positive control for both unselected and selected pools. Some codon sites, such as E83, exhibited evidence of selection in the unselected pool thereby indicating that the evidence of selection may be attributed to putative cloning artifacts [20]. The well-known GIY-YIG domain has been highlighted in grey and is discussed in detail under 'Evidence of Selection'.

The EoS score is the primary criterion used by Kleinstiver *et al*. [20] to identify functionally-relevant residues in I-Bmol via unigenic evolution. The EoS scores for the first 88 sites of I-Bmol are shown in Figure 1.

Although (9) is derived using similar principles to Kullback's and Leibler's information divergence [26], a similarity exploited below, it is not obvious why integrating the logarithm of (8) over the posterior parameter estimates is a statistically valid procedure. Briey, this integration is justified because log-odds-ratios are isomorphic to standard Euclidean vector spaces [27-29] and can be 'added' together in a meaningful manner that is consistent with the fundamental laws of probability. For example, consider two different point-values for ${\left[p_{\text{sn},j}^{k},p_{\text{ns},j}^{k}\right]}_{H_{A}}$ and ${\left[p_{\text{sn},j}^{k},p_{\text{ns},j}^{k}\right]}_{H_{0}}$ for the integrand of (9). The first point-value yields an odds-ratio of 10:1, indicating a preference for *H_A_*, while the second point-value yields 1:10, indicating a preference for *H*_0_. Intuitively, if these two point-estimates were the only parameter values available, we expect the 'total' odds-ratio to be 1:1. In other words, the data supports both hypotheses equally. This 'total' is represented exactly by summing the log-odds-ratios, where

$$\left(\log_{10}(1)+\log_{10}(10)\right):\left(\log_{1}(1)+\log_{10}(10)\right)=1:1,$$

as expected. Such 'addition' holds over very general conditions, and underlies not only the validity of (9) for computing $R_{j}^{k}$, but the foundations of information theory and the Kullback-Leibler divergence [30].

The interpretation of log-odds-ratios in the literature is traditionally taken as the 'strength of evidence' favoring one hypothesis over another [31,32]. However, since *H_A _*describes a general case that embeds *H*_0 _as an alternative, this traditional interpretation is inappropriate for $R_{j}^{k}$. Instead, $R_{j}^{k}$ is interpreted in the Neyman-Pearson sense, where it describes the relative probability of correctly classifying a specific observation to be due to either *H_A _*or *H*_0_. Interpretation of $R_{j}^{k}$ requires considering three cases:

• $R_{j}^{k}>0$: *H_A _*is more probable than *H*_0_, with $R_{j}^{k}$ being the expected logarithm of the true-positive to false-negative ratio for determining whether or not selection operated on the site.

• $R_{j}^{k}≃0$: Both *H_A _*and *H*_0 _are supported equally by the data, implying that the data are unable to differentiate between whether or not functional selection has occurred.

• $R_{j}^{k}<0$: Although technically implying that *H*_0 _is more probable than *H_A_*, the embedding of *H*_0 _within *H_A _*implies that negative values of $R_{j}^{k}$ will likely have small magnitudes and can be interpreted as if they are zero.

The interpretation of $R_{j}^{k}<0$ follows from Gibbs' inequality which guarantees the probability-weighted average of $R_{j}^{k}\left(n_{\text{sn},j}^{k},n_{\text{ns},j}^{k}\right)$ over all possible mutation counts to be non-negative. Therefore observations for which $R_{j}^{k}<0$ are likely due to sampling variance and are best interpreted, unless large in magnitude, as if they were zero. In practice, as shown both in Figure 1 and the data of Kleinstiver *et al*. [20], large negative values for $R_{j}^{k}$ have not been observed.

A concrete example of interpreting $R_{j}^{k}$ is given by examining the GIY-YIG motif of I-Bmol, shown grey-highlighted in Figure 1. Precise values of $R_{j}^{k}$ along with the expected and observed number of nonsynonymous mutations are given in Additional File 3. Although all six motif-residues are well conserved within this homing endonuclease family, Figure 1 shows that unigenic selection is only detectable for the tyrosine residues which show posterior odds-ratios of 2^5.8 ^≈ 59.7-to-one in favour of selection. The four-to-one odds ratio or less shown by the other residues is by general convention considered to be negligible.

**This example highlights how the lack of evidence of selection does *not *imply a lack of functional importance.** Lack of evidence is precisely that: there is not enough data to classify a given site as either 'important' or 'unimportant'. Often, as can be seen with the GIY-YIG motif, many codons are intrinsically resistant to nonsynonymous changes under mutagenic PCR. The glycine residues, for example, can be seen to have had five mutations at site 4 in the selected population but less than one of them is expected to be nonsynonymous. Much of the reason that selection is detectable at the tyrosine residues is the large number of expected nonsynonymous mutations, a number principally dependent on the tyrosine codon's nucleotide composition. The number of nonsynonymous mutations expected for different clone population sizes is shown in Additional File 4 and is described more fully later.

We note an advantage of our method over previous work is shown by examining $R_{j}^{k}$ values for the *unselected *population. There, the negligible values of $R_{j}^{k}$ act as a negative control indicating 'no evidence of selection' when no selection is actually present. Large values of $R_{j}^{k}$ in the unselected clone population would indicate the presence of systematic bias or experimental errors.

### Power and Reliability

The similarity of (9) to a Kullback-Leibler divergence can be exploited to estimate the statistical power for inferring selection at a given site. The integrand of (9), for given parameter values and where Pr(*H_A_*) = Pr(*H*_0_), is the log-ratio of multinomial likelihoods. Taking the expectation of this log-ratio over the space of all possible data given *H_A _*yields a point-estimate of the Kullback-Leibler divergence

$${D^{\prime }}_{H_{A}}=\sum_{\left[n_{\text{sn},j}^{k},n_{\text{ns},j}^{k}\right]}\mathrm{Pr}\left(n_{\text{sn},j}^{k},n_{\text{ns},j}^{k}|n^{k},H_{A}\right)\log\left(\tilde{L}\right)$$

where

$$\tilde{L}=\left[\frac{\mathrm{Pr}\left(n_{\text{sn},j}^{k},n_{\text{ns},j}^{k}|n^{k},p_{\text{sn},j}^{k},p_{\text{ns},j}^{k},H_{A}\right)}{\mathrm{Pr}\left(n_{\text{sn},j}^{k},n_{\text{ns},j}^{k}|n^{k},p_{\text{sn},j}^{k},p_{\text{ns},j}^{k},H_{0}\right)}\right],$$

which can itself be integrated over the multinomial parameter posteriors, as before, to yield the overall expected divergence $D_{H_{A}}$. As interpreted by Kullback [26,30], $D_{H_{A}}$ measures how distinguishable two random variables are in terms of the expected true-positive versus false-negative rate. Conditioning on *H*_0_, the complementary $D_{H_{0}}$ provides the expected true-negative versus false-positive rate. Together, $D_{H_{A}}$ and $D_{H_{0}}$ specify the confusion matrix between hypotheses, thus providing a detailed power estimate of hypothesis distinguishability.

**Figure 2:**
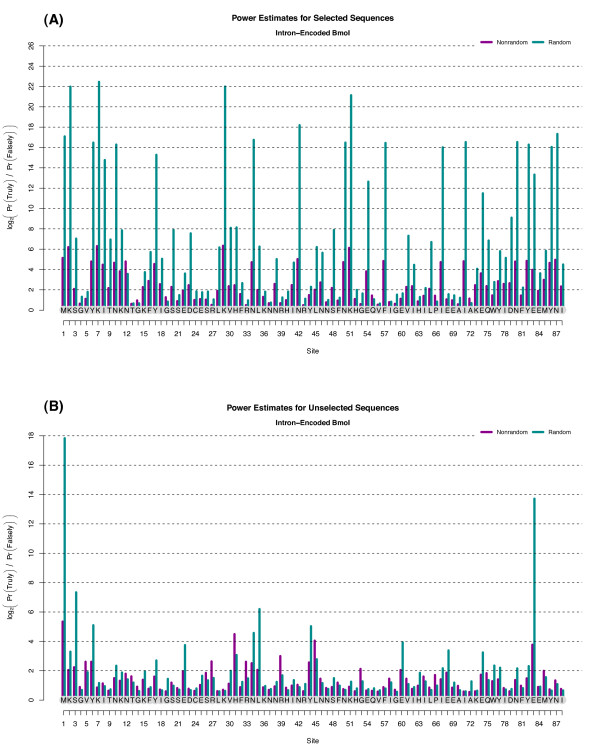
**The Reliability of Inference for I-BmoI**. Estimates of statistical power (reliability of inference) for I-Bmol, for both selected and unselected clone populations. 'Nonrandom' associations are presumed due to the alternate hypothesis while 'random' associations are presumed due to the null. Power is computed as the expected log_2_-odds-ratio for either true-positive versus false-negative (mauve) or true-negative versus false-positive (teal). **(A) **For the selected sequences, the true-positive ratio is strongly correlated with the posterior log_2_-odds-ratio shown in Figure 1. **(B) **Approximate inferential power of sites in the unselected population, each of which is expected *a priori *to be near zero. Note that both M1 and E83, identified as putative outliers in Figure 1, are shown to clearly (and unexpectedly) violate the null hypothesis. Such violations hallmark systematic errors or experimental artifacts.

Another way of interpreting the per-site values of $D_{H_{A}}$ and $D_{H_{0}}$ is through the idea of 'reliability'. If we assume that the mutation frequencies estimated via *H_A _*and *H*_0 _are even approximately correct, $D_{H_{A}}$ and $D_{H_{0}}$ quantify the estimated robustness of $R_{j}^{k}$ by averaging it over the range of expected nonsynonymous mutation counts. The estimated reliability of the EoS score for I-Bmol is shown in Figure 2. For the selected population, the true-positive reliability scores are highly correlated with their respective $R_{j}^{k}$ values, agreeing with the intuitive notion that the greater the evidence of selection, the more likely that that evidence is reliable.

### Effect of Sample Size

To help elucidate the *practical *effects of sample size on EoS values with respect to both the unselected and selected clone population, subsampled clone populations were analyzed with results displayed in Additional File 5. In brief, even as few as 10 unselected clones (yielding 100-300 nucleotide misincorporation counts) were capable of giving reasonable estimates of parameter matrix *T *and sensitivity. Reasonable specificity however, as judged by the ability to correctly detect selection of the methionine start signal, was not achieved with fewer than all 87 of the unselected clones. With respect to the misincorporation frequencies estimated from the counts in Table 1, percentiles of the likely number of nonsynonymous mutations observed for given clone population sizes under the null hypothesis of 'no selection' are shown in Additional File 4. This table shows considerable non-normality that is particularly pronounced for mutation-resistant codons, highlighting the requirement (and opportunity) to 'tune' the effective selection pressure on individual residues by adjusting codon composition. Again, since normality is a requirement for the validity of *χ*^2^-based statistics, the non-normality displayed by many residues even under very large sample sizes (> 500 clones) calls the validity of such analyses into question.

## Global Insights from Local

The derivation of $R_{j}^{k}$ (EoS), although a primary result of this work, is alone insufficient to analyze unigenic evolution data. The following subsections provide additional computational details required for a complete analysis.

### Selecting Selected Sites

One of the major shortcomings of previous work was the difficulty of discerning which *groups *of sites were under functional selection given statistical procedures that were designed under the assumption of site-independence. Given *n *codons with 'sufficiently-high' EoS score, there are *n*! different ways to partition those *n *codons into functionally-important and functionally-unimportant categories and thereby estimate the false-discovery rate. This huge number of partitions implies that traditional techniques for multiple-comparison correction reduce statistical power to impractically low levels.

The 'windowing' analyses of Deminoff *et al*. and Behrsin *et al*. were used to constrain the number of required multiple-test corrections to a reasonable level.

The principal benefit of using $R_{j}^{k}$ as the evidence of selection is that the additive nature of log-odds-ratios imply that $R_{j}^{k}$ values can simply be summed across all sites *j *of interest without unnecessary loss of inferential power. If *J *denotes the set of sites-of-interest, then the combined log-odds-ratio

(10) $$R_{J}^{k}=\sum_{j\in J}R_{j}^{k}$$

can be interpreted as the log of relative probability that *all *sites in *J *were observed due to the action of selection as hypothesized by *H_A_*. Unlike traditional multiple-test corrections such as Bonferroni's, $R_{J}^{k}$ intrinsically 'self-corrects' for the number of sites considered.

The benefit of using $R_{J}^{k}$ as evidence of selection in unigenic evolution is clearly demonstrated by Figure eight of Kleinstiver *et al*. [20] via the functional analysis of I-Bmol. There, assays of N12 D, S20Q, H31A, I67N, and I71N mutants clearly implicate these residues, as identified by their EoS scores, as functionally important. This importance is seen experimentally as the generation of a phenotype distinct from wild-type for each respective mutant.

Note that $R_{J}^{k}$ systematically underestimates the posterior odds that a set of sites are subject to selection since it predicates on all sites of *J *being selected. If only one or two of these sites were false-positives, $R_{J}^{k}$ behaves as if all sites *J *were false-positives, artificially reducing the actual true-positive rate. It is straightforward, though tedious, to compute precise overall true-positive rate given $R_{j}^{k}$ for *j *∈ *J*. However, in practice the individual values $R_{j}^{k}$ generally display a sharp boundary between 'large' and 'small' values, making the choice of putative functional sites straightforward via simple inspection (see Figure 1).

We further note that neither $R_{j}^{k}$ or $R_{J}^{k}$ are directly comparable to either the *H*-scores or *χ*^2^-values of Deminoff *et al*. or Behrsin *et al*. since the former condition explicitly on observed data while the latter condition on unobserved hypothetical data. Restated, the former is congruent to a Type-I error probability (*α*), while the latter is a Fisher-type significance *p*-value. Although correlated, these two values have no simple relationship.

### Protein Mutation Count

For the experimental conditions used herein, nonsynonymous mutation probabilities varied between ≈ 0-10%, depending on the given codon. These mutation probabilities in turn determine *κ*, the total number of nonsynonymous mutations expected in the overall protein. The overall mutation count is an important experimental diagnostic since too few mutations lead to inefficient mutagenesis and high sequencing costs, while too many mutations result in nearly-certain functional knockout. The simplest method of computing the distribution of *κ *uses standard Monte Carlo techniques to perform *in silico *mutagenesis of the wild-type sequence given nucleotide mutation parameters under *H*_0_.

**Figure 3:**
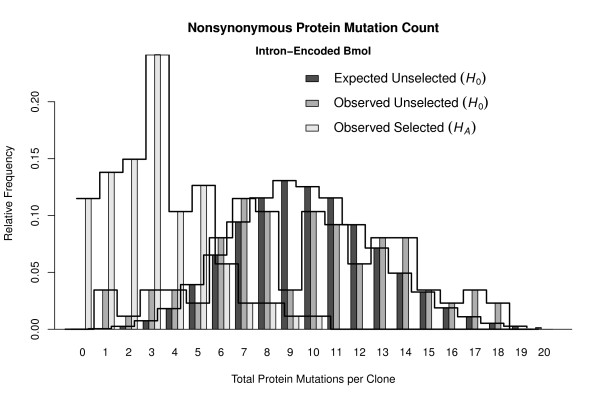
**The Expected Mutation Count Distribution for ****I-Bmol**. The expected distribution of total nonsynonymous mutations per clone (*κ*) for I-Bmol under the null hypothesis of 'no selection', the observed mutation count frequencies for the unselected clones, and the observed mutation count frequencies for the selected clones under the alternate hypothesis. Over 96.7% of clones are expected to have between 4-16 nonsynonymous mutations, inclusive, within the 266 amino acid sites of I-Bmol. Expected and observed distributions under *H*_0 _were very similar, considering the sample size of 87 clones. The selected population, under *H_A_*, displayed a marked decrease in the number of mutations, with > 95% of clones having seven or fewer mutations.

The distributions of total protein mutations per clone, both expected and observed for the unselected clones under *H*_0 _and observed for the selected clones under *H_A_*, are shown in Figure 3. Under *H*_0 _> 96.7% of clones are expected to have between 4-16 nonsynonymous mutations, inclusive, within the 266 amino acid sites of I-Bmol. Expected and observed distributions under *H*_0 _were very similar, especially considering the small sample-size of 87 clones that was available to estimate the distribution. The selected population, under *H_A_*, displayed a marked decrease in the number of mutations, with > 95% of clones having seven or fewer mutations.

Note that like previous analyses, both our null and alternate hypotheses assume site-independence among mutations. Second-site suppression or other modes of site-interaction are not taken into account. Under rare conditions for I-Bmol, it is possible that up to 20 mutations could be expected for this 266-site protein, making it likely that interaction effects are non-negligible factors of selection.

### Comparison with Previous Work

**Figure 4:**
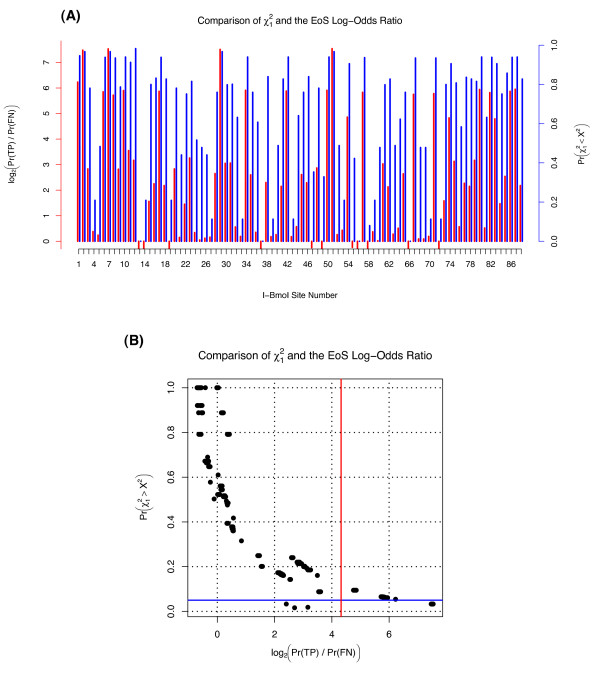
**A Comparison of EoS values with Prior Work**. Comparison of the EoS value with the $\chi_{1}^{2}$ statistics of prior work. **(A) **A per-site comparison of EoS values (left, red) with $\chi_{1}^{2}$ statistics (blue, right). Both values are plotted such that larger values roughly indicate 'greater significance'. **(B) **EoS and $\chi_{1}^{2}$ values are highly though non-linearly correlated, but are *not *comparable in terms of 'significance'. Specifically, while only 10/266 sites exceed the $\chi_{1}^{2}$ critical value of *p *< 0.05 as shown by the blue horizontal line, 41/266 sites showed a posterior odds ratio of 20:1 or greater as shown by the red vertical line. We emphasize that binning adjacent sites would increase the specificity of the *χ*^2 ^statistic at the cost of considerable sensitivity as per Behrsin *et al*. [7], and that 20:1 odds ratios are only roughly comparable to *p *values of 0.05.

A comparison of our EoS values with the *χ*^2^-based statistics of previous work is shown in Figure 4. In a broad sense, non-binned site-specific *χ*^2 ^statistics with one degree of freedom and EoS values appear to be highly predictive of one another. However, their interpretation and use are very different, especially with respect to sensitivity-*vs*.-specificity and multiple-comparison correction. For example, the single degree of freedom used in Figure 4 dictates a wide sampling variance for the $\chi_{1}^{2}$ statistic, translating to a very high predicted false-positive rate. Binning adjacent sites such as done by Behrsin *et al*. [7] could reduce the false positive rate but only at the expense of a concomitantly-lower true positive rate -- the classic sensitivity versus specificity tradeoff.

Nonetheless we note that even using the highly-sensitive $\chi_{1}^{2}$ values, only 10 of the 266 sites exceeded the critical *p*-value of *p *< 0.05, whereas using EoS, 41 of 266 sites exceeded the critical log-odds ratio of 20:1 (see Figure 4). Thus previous methods identify less than 25% of sites identified with EoS. Most importantly, the additional sensitivity of the EoS value appears *without *detriment to specificity, as shown by the estimated false-positive to true-negative ratios in Figure 1, ratios that are impractical to estimate via previous methods. Thus our method appears both significantly more sensitive *and *selective than previous work for the unigenic analysis of I-Bmol.

Another advantage of EoS values over *χ*^2^-based statistics is illustrated by the nontrivial comparison of the *p *< 0.05 and 20:1 odds-ratio critical values shown in Figure 4. Although detailed interpretations and the differences thereof have already been discussed, in the particular case of the I-Bmol data shown in Figure 4 it is important to realize that Bonferroni correction of the $\chi_{1}^{2}$ values would render none of the sites significant by conventional measures. In contrast, the log-odds ratio shows comparatively little power-loss due to multiple-comparison correction.

From a theoretical viewpoint, another advantage of the EoS value is that it deals with a very direct statistical question: how likely are the observed data given the model? Unrealistic EoS values are necessary and sufficient to diagnose unrealistic assumptions in the mathematical description of unigenic evolution. In contrast, summary statistics such as *χ*^2 ^values similarly condition on model accuracy, but necessarily *further *condition on the statistic being a good indicator of the phenomenon under investigation -- in this case, selection. Thus unrealistic values of *χ*^2 ^can be similarly be attributed to model mis-specification, but could also be due to the inadequacy of the chosen test statistic.

## Additional Results

Although the main result of this work is the EoS score $R_{j}^{k}$ and its associated reliability estimate, two additional results were discovered during the analysis of the I-Bmol system. The first result concerned differences between *H*_0 _and ${H^{\prime }}_{0}$, the second concerned the treatment of stop codons.

### Polymerase Versus PCR Modelling

A surprising result was that $R_{j}^{k}$ and power estimates computed under *H*_0_, modelling only the overall PCR process, and ${H^{\prime }}_{0}$, modelling the misincorporation of nucleotides by *Taq* polymerase, were effectively indistinguishable. Observed log-odds differences were all on the order of the Monte Carlo sampling-variance cutoff used to estimate each and were therefore effectively zero. The inability of the data to discriminate between *H*_0 _and ${H^{\prime }}_{0}$ implies that there is no effective difference between these models. In effect, there is no meaningful difference in the $R_{j}^{k}$ scores computed by either (a) treating mutagenic PCR as a 'black-box' process or (b) modelling mutagenic PCR to be be consistent with the action of error-prone polymerase.

This finding is surprising because modelling the effect of the polymerase over multiple PCR cycles appears to be required in order to produce mutation frequencies, such as those in Table 2, where complementary-base frequencies are always almost equal. The similarity of respective $R_{j}^{k}$ values imply that differences between estimates of *P *under each hypothesis are negligible compared to the magnitude of uncertainty inherent in estimating *P *given *C*.

A consequence of this finding is that it implies that the polymerase may not be the major mechanism responsible for nucleotide mutation. For example, cytosine deamination via thermal decomposition during PCR cycling [33-35] provides a credible, alternative mutation mechanism. In this case the non-proofreading property of *Taq* would be a more important factor than its misincorporation characteristics.

Optimizing the experimental conditions under which mutagenesis occurs can have important consequences in the efficiency and expected outcome of unigenic evolution. For example, note the ≈ 100-fold difference between {C ← G} and {G ← A} mutation frequencies shown in Table 2. Small changes in mutation frequencies due to different mutagenic protocols could effect large changes in the distribution of mutant codons, suggesting that future work should investigate mechanisms to elucidate the factors affecting the precise characteristics of mutagenic PCR.

### Stop Codon Assumption

One of the more significant assumptions of this work is the presumption that the effect of stop codons can be ignored. Unlike other mutations, the appearance of a premature stop codon affects every subsequent codon, negates our assumption of site-independence, and likely results in a complete loss of function. For the system examined herein and in Kleinstiver *et al*. [20], we found that the frequency of stop codon production was sufficiently small as to not significantly affect results or interpretation. Given this putatively small effect, a more exact treatment of stop codons and their functional effects are left for future work since a correct and rigorous treatment would likely add considerable algebraic and computational complexity.

## Conclusions

From an experimental point of view, the evidence of selection at a given site represents only part of the required information; in general, the reliability of that evidence must also be assessed. Quantifying reliability is important since small sample sizes, mutation events that are too rare to be reliably estimated, and the effects of multiple comparisons can complicate the interpretation of unigenic evolution experiments. Our method, which computes both evidence and reliability, represents a significant advance over previous work since it simultaneously assesses both the evidence of selection and the reliability of inference.

Experimental validation of our methods was provided via analysis of the poorly-characterized homing endonuclease I-Bmol, where previously-described methods from the literature were *unable *to elucidate functionally-critical residues. With the ability to guide the selection of precise experimental mutagenesis conditions, our method makes unigenic analysis a more broadly applicable technique with which to probe protein function.

## Methods

Herein we provide technical and implementation details for our analytical framework, the most important of which are (a) the estimation of multinomial frequencies from counts, (b) our model of mutagenic PCR via the action of an error-prone polymerase, and (c) selecting the prior and sampling the posterior of the polymerase misincorporation frequencies.

### Multinomial Estimation

The estimation of multinomial frequencies from counts is one of the oldest subjects in statistics. When asymptotically-many observations are available, both Bayesian and frequentist methods infer nearly identical parameter values, where frequencies are simply proportional to counts. However, when observations are rare, prior beliefs will always, necessarily significantly affect inferred results. These effects, thoroughly described by Jaynes [36], can be understood though a simple example. Suppose that 10 000 clones were sequenced of which 5000 were found to have nonsynonymous mutations at a given site. Using the normal approximation to the binomial distribution both the mean frequency of nonsynonymous mutation and its standard error are easily computed with high accuracy. However, if only *one *nonsynonymous mutation had been observed, the actual frequency of mutation is not clear since mutation frequencies of 0.5, 1, or 2 mutations per 10000 clones, a range of 400%, are all realistic and compatible with the given data. Prior belief that the mutation rate should be 'somewhat high' will favor the higher rate, surprise at seeing *any *mutation would imply the lower rate is more believable.

The consensus in the statistical community is now that there is no 'best' notion of 'prior ignorance' that can be universally considered correct [37,38]. Instead, research has focused on developing methods with precise and well-characterized assumptions in order to minimize, in some sense, the influence of prior assumptions on the inference. These well-charactered assumptions are called 'objective' or 'reference' priors and are the type of prior we choose as a basis for inferring both multinomial nucleotide mutation frequencies and nonsynonymous codon mutation frequencies.

If *p *represents a set of multinomial frequencies and *n *a set of observed counts, then Bayes' Theorem tells us that

(11) Pr(p|n) ∝ Pr(n|p)⋅Pr(p),

where Pr(*n*|*p*) is the likelihood of the counts given parameters and Pr(*p*) is our assumed prior distribution of the parameters *before *any data is observed. Detailed information-theoretic studies by Berger and Bernardo [25] found that *p *~ Dirichlet(*α*) with all components of vector *α *set to 1/2 was a prior that formally minimized the inuence of the prior on the posterior Pr(*p|n*). This specific prior was found to be invariant to reparameterization and is identical to the one derived by Jeffreys [39].

From an experimental viewpoint, invariance to reparameterization is an critical requirement for inferring frequencies from counts since the property implies that the *same *inference would be made if, for example, relative mutation *rates *had been estimated rather than *frequencies*. Any other choice of prior would yield different posterior values of *p *even when given identical data.

For the multinomial distribution with the objective reference prior above, the posterior has the simple form

(12) p|n~ Dirichlet(n+α).

Again, only *α *= 1/2 formally maximizes the information 'extracted' from the counts *n*. The expected value of these posterior relative frequencies is

(13) $$E\left[\log\left(p_{i}\right)\right]=\sum_{i}\psi \left(n_{i}+\alpha_{i}\right)+\psi \left(\sum_{i}\left(n_{i}+\alpha_{i}\right)\right)$$

for each frequency-component *i*, where *ψ *denotes the digamma function. Equation (13) is termed the 'natural' parameter mean for *p*, and when *n_i _*is sufficiently large it is approximately equal to *n_i_*/Σ*_i _n_i_*. This similarity is evident when comparing the 'Relative Count' and 'Natural Parameter' estimates for nucleotide mutation frequencies in Table 2 where all four multinomial parameter set estimates agree to within 1%. However, for codon mutation counts on the order of 0-2 observations per 100 clones, differences between estimates can be considerable.

### Modelling PCR via Polymerase

Modelling the full mutagenic PCR process requires formally describing both the process of nucleotide misincorporation via polymerase and the action of multiple cycles of denaturation, synthesis, and reassociation that are the basis of PCR. Our model uses a Bayesian framework that explicitly accounts for the rarity of nonsynonymous mutations to infer parameter values of this model.

#### Polymerase Misincorporation

Mutagenic PCR is composed of of multiple cycles of low-fidelity *Taq*-based amplification. The model we adopt assumes that under mutagenic conditions [6]*Taq* polymerase

• induces errors *only *by nucleotide misincorporation,

• has negligible slippage, stutter, or other errors due to repeats,

• and has site-independent misincorporation probabilities.

The assumption that slippage and stutter are negligible is substantiated by previous studies of *Taq* errors [6,34] and visual inspection of our data. The assumption that nucleotide misincorporation events are independent is somewhat stronger since it may be argued that spatial distortions induced by template-adduct mispairing affect subsequent DNA synthesis. However, inspection shows misincorporation events to be sufficiently rare that this effect, if it exists, is of sufficiently small magnitude as to be negligible.

A single nucleotide is represented as the four-dimensional probability column-vector *p *that comprises the probability of that nucleotide being one of the four nucleotides A, C, G, or T. The action of *Taq* polymerase on template *p *is modelled as a linear Markov operator *T *that pairs adduct *q *= *Tp *against the template during synthesis. Polymerase operator *T *has the explicit representation

(14) $$T=\left[\begin{array}{cccc} \tau_{\text{AA}} & \tau_{\text{AC}} & \tau_{\text{AG}} & \tau_{\text{AT}}\\ \tau_{\text{CA}} & \tau_{\text{CC}} & \tau_{\text{CG}} & \tau_{\text{CT}}\\ \tau_{\text{GA}} & \tau_{\text{GC}} & \tau_{\text{GG}} & \tau_{\text{GT}}\\ \tau_{\text{TA}} & \tau_{\text{TC}} & \tau_{\text{TG}} & \tau_{\text{TT}} \end{array}\right],$$

where *τ_ij _*denotes the probability that adduct-nucleotide *i *is base-paired to template nucleotide *j*.

With this notation, the columns of *T *sum to one. Further, since misincorporation events are rare, the counter-diagonal components *τ*_AT_, *τ*_CG_, *τ*_GC_, and *τ*_TA_ are assumed to be ≈ 1, while all other parameters are ≪ 1. We emphasize that a full specification of *T *requires sixteen parameters and four constraints, yielding twelve independent degrees of freedom. These constraints imply that both matrix *T *and an an implicit twelve-parameter model describing the *relative **Taq* misincorporation frequencies are equivalent.

#### The Mutagenic PCR Process

**Figure 5:**
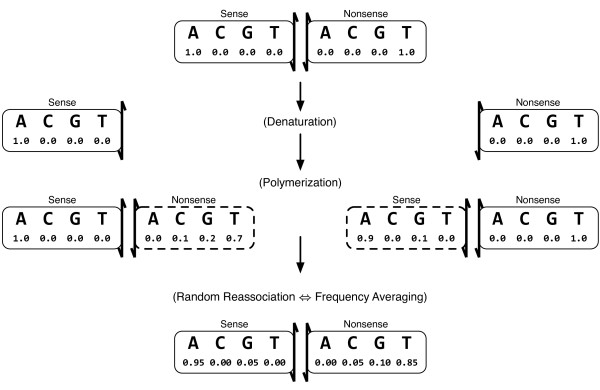
**A Model of Mutagenic PCR**. A model of a single cycle of mutagenic PCR. Each nucleotide of both sense and nonsense strands are treated as probability four-vectors. The 'state' of a nucleotide is the relative frequency we expect to observe it as either A, C, G, or T. The initial wild-type sequence is presumed to be well-defined. An example PCR cycle begins with A and T on the sense and nonsense strand, respectively, that are subsequently separated via denaturation. Error-prone polymerization of new nonsense and sense strands by *Taq* polymerase have, for this example, probabilities of 0.3 and 0.1 of nucleotide misincorporation. Random reassociation at the end of the cycle effectively averages the frequency of mutation at each site and implies that nucleotide frequencies are statistically independent despite being physically contiguous on the same strand. After the final PCR cycle completes a random sample of the DNA strands are selected for cloning, a fraction of which are subjected to selection based on the nucleotide sequence of the sense strand alone.

A single DNA fragment to be amplified consists of two base-paired strands, the sense strand encoding the protein of interest and its complement the nonsense strand. Let four-vectors *s *and *n *represent the nucleotide *pair *at a single site. Four of the sixteen combinations of *s *and *n *represent proper Watson-Crick base pairs while the remaining twelve represent (presumably rare) mispairings. An individual cycle of PCR amplification can then be modelled as the process shown in Figure 5. Although initially base-paired, the two strands are separated and become independent when the DNA is denatured. *Taq* polymerase proceeds to add stepwise adducts onto each strand in a template-dependent manner. The attachment of a subsequent adduct is independent of the adduct at the previous position. In this way, a newly polymerized nonsense strand is built up using the sense strand as a template. Any mistakes during polymerization result in nucleotide mismatches between the strands which are not repaired because *Taq* polymerase does not contain a proofreading activity nor are other DNA repair enzymes present in the *in vitro *reaction. The individual nucleotide site-pairs can thus be considered independently of all other site-pairs, even though they are physically contiguous with other site-pairs on the oligonucleotide.

Mathematically, this single PCR cycle can be described by an operator Φ that acts on the sense-nonsense pair (*s*, *n*) such that

(15) $$\Phi :(s,n)\mapsto \left(\frac{1}{2}(s+Tn),\frac{1}{2}(n+Ts)\right),$$

where *T *denotes the *Taq* polymerase operator (14). Starting from the wild-type sense-nonsense pair (*s*_0, _*n*_0_) the probabilistic base-pair mixture after *k *rounds of mutagenic PCR can be computed via the iterative formula

(16) $$\left(s_{k},n_{k}\right)=\Phi \left(s_{k-1},n_{k-1}\right).$$

The resultant sense-strand probability vector *s_k _*is therefore a highly-nonlinear function of the *Taq* error probabilities. For illustrative purposes we show the elegant Pascal-triangle-like hierarchy resulting from explicit representations of {2*^k^s_k_*} for *k *= 0 ... 7 as follows:

$$\begin{array}{c} \left\{s_{0}\right\}\\ \left\{Tn_{0}+s_{0}\right\}\\ \left\{T^{2}s_{0}+2Tn_{0}+s_{0}\right\}\\ \left\{T^{3}n_{0}+3T^{2}s_{0}+3Tn_{0}+s_{0}\right\}\\ \left\{T^{4}s_{0}+4T^{3}n_{0}+6T^{2}s_{0}+4Tn_{0}+s_{0}\right\}\\ \left\{T^{5}n_{0}+5T^{4}s_{0}+10T^{3}n_{0}+10T^{2}s_{0}+5Tn_{0}+s_{0}\right\}\\ \left\{T^{6}s_{0}+6T^{5}n_{0}+15T^{4}s_{0}+20T^{3}n_{0}+15T^{2}s_{0}+6Tn_{0}+s_{0}\right\}\\ \left\{T^{7}n_{0}+7T^{6}s_{0}+21T^{5}n_{0}+35T^{4}s_{0}+35T^{3}n_{0}+21T^{2}s_{0}+7Tn_{0}+s_{0}\right\} \end{array}$$

For practical computation, however, equation (16) should be used to avoid excessive underflow and truncation errors.

Given a polymerase matrix *T*, the overall multi-cycle mutagenic PCR process can be described by computing *s_k _*under four different conditions, namely the condition that *s*_0 _was precisely one of A, C, G, or T. Let $s_{k}^{\ell }$ denote the probability vector *s_k _*given *s*_0 _was precisely nucleotide ℓ. Then we can describe the action of the multi-cycle mutagenic PCR process on the wild-type sequence by the linear 4 × 4 Markov operator *P *as the column-concatenation of the $s_{k}^{\ell }$ such that

(17) $$P=[\;\begin{array}{cccc} s_{k}^{\text{A}} & s_{k}^{\text{C}} & s_{k}^{\text{G}} & s_{k}^{\text{T}} \end{array}]$$

where, again, each column sums to one. Given a wild-type nucleotide sense-strand state-vector *w_s _*and *Taq* error probabilities *T*, operator *P *transforms *w_s _*into *m_s _*= *Pw_s_*, where *m_s _*is the probability state-vector for a mutant sense-strand after *k *cycles of mutagenic PCR.

Under the null hypothesis of 'no selection', the likelihood of misincorporation counts *C *given mutation probabilities *P*(*T*) is given by the product of the four multinomial distributions

$$\begin{array}{cc} \mathrm{Pr}\left(C|P(T)\right) & \\ & =\tilde{M}\prod_{j\in \{\text{A,C,G,T}\}}{\left(p_{\text{A},j}\right)}^{c_{A,j}}{\left(p_{\text{C},j}\right)}^{c_{C,j}}{\left(p_{\text{G},j}\right)}^{c_{G,j}}{\left(p_{\text{T},j}\right)}^{c_{T,j}}, \end{array}$$

where

$$\tilde{M}=\left(\frac{\left(\sum_{k}c_{kj}\right)!}{\;(c_{\text{A},j})!(c_{\text{C},j})!(c_{\text{G},j})!(c_{\text{T},j})!}\right)$$

and each frequency *p_ij _*is complicated, nonlinear function of the misincorporation frequencies *T*. Since the entries of *T*, not *P*, are the fundamental parameters of likelihood (18), the standard Dirichlet prior described above is inappropriate. Instead, a corresponding noninformative 'objective' prior is derived for it, below.

**Figure 6:**
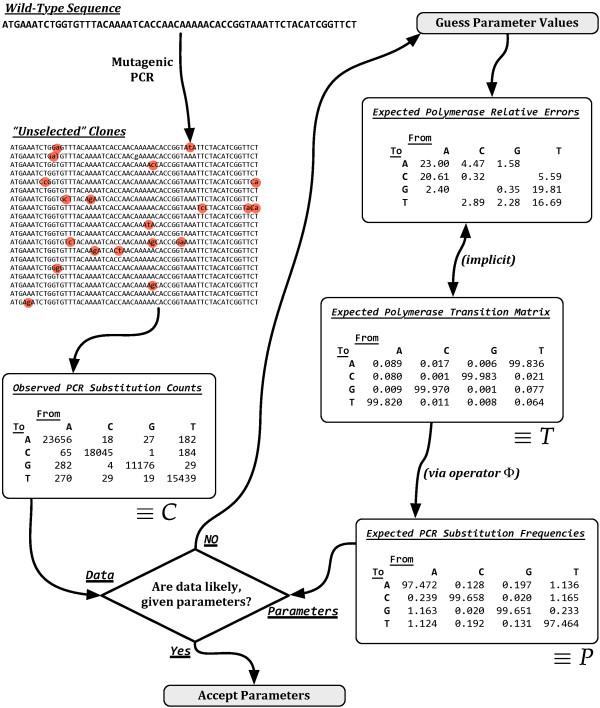
**Inferring Polymerase Errors from Misincorporation Counts**. The conceptual relationship between mutagenic PCR, the unselected clone population, and characteristics of the *Taq* polymerase. The top-left depicts a wild-type sequence that, when subject to mutagenic PCR, results in a clonal population that has not been selected for functional integrity. The number of misincorporations from a wild-type nucleotide to a clone-type nucleotide is summarized as 'Observed PCR Misincorporation Counts' matrix *C*. The top-right depicts how relative *Taq* misincorporation rates are implicitly assumed from the 'Expected Polymerase Transition Matrix' *T *that describes how *Taq* incorporates nucleotides onto the nascent strand. These misincorporation probabilities are converted to 'Expected PCR Misincorporation Frequencies' *P *via nonlinear 'PCR Operator' Φ, described in the text, that models the multi-cycle PCR process. A Bayesian procedure is used to determine the compatibility of the given parameters with the observed data, as described under 'Methods'.

A conceptual flowchart of how parameters *T *are chosen with respect to counts *C *is shown in Figure 6. The figure describes in essence how samples of the posterior Pr(*T|C*) are realized. It is worth emphasizing that the sixteen parameters of *T *possess only twelve degrees of freedom, as previously discussed and shown explicitly in Figure 6, because statistical algorithms must be carefully designed to be correct with regard to such constraints.

### Polymerase Priors and Posteriors

Choosing a prior distribution for likelihood (18) is not trivial, especially since there is no universal notion of "complete prior ignorance" [9,37,40]. Choice of prior is therefore governed by specific criteria assumed by the investigator to be important. One nearly universally-accepted criterion is that of *reparameterization-invariance*, a criterion requiring that the inference should not depend on the units of either the parameters or observations. The importance of such invariance has been detailed by Jeffreys [39], Wallace and Freeman [41], Jermyn [42], and many others. In our context, such invariance ensures that parameterizing the likelihood by, for example, either "the expected number of observed misincorporations per PCR cycle" or its reciprocal "the expected number of PCR cycles before a misincorporation is observed" yield equivalent inferences. Since there is no meaningful physical difference between these two parameterizations it is essential that each yield equivalent results.

Under even highly-mutagenic conditions, the error probabilities given by the columns of *T *are known *a priori *to be very close to the extremes of either zero or one. *Taq* polymerase is one of the best-studied polymerases in molecular biology and its error rate is known to be heavily influenced by the precise experimental conditions under which it is used [6,43]. Therefore, since the precise *relative scales *of the different types of polymerase errors are not known, we derived a prior for *T *that is 'objective' in the sense of Berger *et al*. [38]. An 'objective' prior is one that formally minimizes, in an information-theoretic sense, the influence of the prior on the posterior and is constructed to always be invariant to reparameterization.

#### An Objective Polymerase Prior

Fortunately, there is a common case where selection of the prior is almost universally accepted, and that is when the model parameters form an Abelian Lie group [9], or in other words, when the parameter space is a commutative group on a differentiable manifold. To see that the 16 parameters of *T *conform to this special structure, consider the 16 dimensional parameter column-vector

(19) $$\tilde{T}=\ln\left\{\text{vec}T\right\},$$

where 'vec' is the standard matrix-vectorization operator. Each column-constraint of *T*, namely that it sum to one, is equivalent to removing a particular one-dimensional subspace from span $\left\{\tilde{T}\right\}$.

Removing the specific groups of interest results in the quotient-space

(20) $$\text{span}\left\{\tilde{T}\right\}/\text{span}\left\{e_{1},e_{2},e_{3},e_{4}\right\},$$

where

$$\begin{array}{l} e_{\text{1}}=\text{vec }\left[1_{\text{4}},\;0_{\text{4}},\;0_{\text{4}},\;0_{\text{4}}\right]\;,\\ e_{\text{2}}=\text{vec }\left[0_{\text{4}},\;1_{\text{4}},\;0_{\text{4}},\;0_{\text{4}}\right]\;,\\ e_{\text{3}}=\text{vec }\left[0_{\text{4}},\;0_{\text{4}},\;1_{\text{4}},\;0_{\text{4}}\right]\;,\text{ and}\\ e_{\text{4}}=\text{vec }\left[0_{\text{4}},\;0_{\text{4}},\;0_{\text{4}},\;1_{\text{4}}\right]\;. \end{array}$$

The quotient-space (20) which fully describes the 16-parameter *T *is therefore isomorphic to the 12-dimensional additive Lie group of ℝ^12 ^which in itself is a Hilbert space [27].

Further justification for assuming that ℝ^12 ^is the 'correct' structure with which to understand *T *comes from realizing that although the columns of *T *are parameters in our likelihood function, they are also discrete and finite probability densities in their own right. Each column of *T *represents the *relative *frequency of concrete events, namely *Taq* misincorporations. When normalized, they represent multinomial probabilities; when non-normalized they represent relative Poisson frequencies.

Our model is only interested in relative relative rates as given by the multinomial model, and it is well-known that the natural parameter space of the multinomial distribution, as a member of the exponential family, is the logarithm of the multinomial parameters.

Given the special Lie group structure of *T*, the appropriate prior is generally considered to be that of Jeffreys [39]. The Jeffreys prior *J *is defined as

(21) $$J\left(T\right)=\sqrt{\left|\det F\left(T\right)\right|},$$

where *F*(*T*) denotes the Fisher Information Matrix (FIM) of the given likelihood model, in this case given by (18). However, it is not entirely obvious *which *likelihood model should be utilized. The likelihoods of *codon *mutation, as given by (5) and (6), condition on the the codon-mutation probabilities. However, our null-hypothesis is computed at the *nucleotide *level because the null-hypothesis states that the selected-clone mutation probabilities are consistent with the unselected-clone mutation probabilities.

Incidentally, we note that although it is widely reported in the literature that Jeffreys' prior fails for 'simple' distributions such as the univariate normal with unknown mean and variance. In fact, Jeffreys' prior results in compatible with frequentist methods if a parameterization with *Abelian *Lie group structure is enforced.

The parameters of *T *are therefore estimated by the 4 × 4 matrix of observed nucleotide point-mutations *n_ij _*. These counts enumerate the number of times a wild-type nucleotide of type *j *was observed to be of type *i *in a sequenced clone. Since mutations are relatively rare, the diagonal *n_ii _*elements are expected to be several orders of magnitude greater than the off-diagonals. The likelihood function (18) can be more compactly written as

$$\ln L\propto \sum_{ij}c_{ij}\ln\left(p_{ij}(T)\right),$$

which, again, is the log-sum of four independent multinomial processes. The parameters *p_ij _*are the probabilities that wild-type nucleotide *j *will be mutated to *i *after *k *cycles of mutagenic PCR and *implicit *conditioning that the wild-type sequence is given to be *j*. For (22) the corresponding 16 × 16 matrix *F *is defined entry-wise as

(23) $${\left[F(T)\right]}_{kl,mn}=-E\left[\frac{\partial^{2}\ln L}{\partial \tau_{kl}\partial \tau_{mn}}|T\right],$$

with the expectation being taken over all possible observations.

Explicit computation of the FIM is straightforward and can be accomplished by noting that

(24) $$\frac{\partial \ln L}{\partial \tau_{kl}}=\sum_{ij}\frac{c_{ij}}{p_{ij}}\frac{\partial p_{ij}}{\partial \tau_{kl}}$$

which implies that

(25) $$\begin{array}{cc} \frac{\partial^{2}\ln L}{\partial \tau_{kl}\partial \tau_{mn}}=\sum_{ij}\frac{\partial }{\partial \tau_{mn}}\left[\frac{c_{ij}}{p_{ij}}\frac{\partial p_{ij}}{\partial \tau_{kl}}\right] & \\ & =\sum_{ij}\;c_{ij}\left[\frac{\partial^{2}p_{ij}}{\partial \tau_{kl}\partial \tau_{mn}}\frac{1}{p_{ij}}-\frac{\partial p_{ij}}{\partial \tau_{kl}}\frac{\partial p_{ij}}{\partial \tau_{kl}}\frac{1}{p_{ij}^{2}}\right]. \end{array}$$

Taking the expectation of (25) as per (23) requires only evaluating E[*c_ij_*]. Since (22) is the log-sum of four multinomials, we can evaluate

(26) $$E\;[c_{ij}]=c_{++}p_{ij}p_{j},$$

where *c*_++ _is the total number of observed wild-type-to-mutant-clone nucleotide pairs and *p_j _*is the probability of that the wild-type nucleotide is of type *j*. Estimation of *p_j _*is equivalent to determining the ratio of (G+C)/(A+T) content for the wild-type sequence.

The nature of the genetic code, be it standard or otherwise, dictates it be extremely unusual for an organism to code for a protein using a nucleotide sequence with (G+C)-content approaching either 0% or 100%. The fraction (G+C)/(A+T) should then be very well approximated by the ratio of the (G+C) to (A+T) counts. Such approximation allows us to assume that the expected (G+C)-content is equal to the observed (G+C)-content, with the result that (26) can be simplified to

(27) $$E\;[c_{ij}]≃\;c_{+j}p_{ij},$$

where *c_+j _*is the row-vector of column-totals of *c_ij _*and can be taken as a given value. Note that in the pedagogical or extremely rare case that (27) is not accurate, the (G+C)-content can always be estimated via standard Bayesian methods at the expense of the simplified computation that we utilize. It is also worth noting that mutagenic PCR never incorporates enough nucleotide changes to appreciably change (G+C)-content.

The final expression for each entry of the Fisher Information Matrix [*F*(*T*)]*_kl,mn _*is therefore proportional to the negative of

(28) $$\sum_{ij}c_{+j}p_{ij}\left[\frac{\partial^{2}p_{ij}}{\partial \tau_{kl}\partial \tau_{mn}}\frac{1}{p_{ij}}-\frac{\partial p_{ij}}{\partial \tau_{kl}}\frac{\partial p_{ij}}{\partial \tau_{kl}}\frac{1}{p_{ij}^{2}}\right],$$

where each parameter *p_ij _*is a function of *T*, and Jeffreys' prior easily computed via the 12-dimensional pseudo-determinant. All that remains is to compute the first- and second-order derivatives of *p_ij _*with respect to the 16 entries of *T*.

These derivatives could be computed analytically. However, since typical experiments use on the order of *k *≈ 30 PCR cycles, analytic derivatives of *P *with respect to *T *result in unwieldily and numerically-unstable expressions. Instead, second-order differentiation arithmetic [44,45] is used via operator overloading in Fortran to compute all required derivatives of *p_ij _*with respect to *T*. Briey, differentiation arithmetic computes a function and its gradient and Hessian simultaneously by utilizing the algebra of differential operators. The simple structure of all relevant equations make them particularly amenable toward straightforward implementation.

#### Sampling from the Posterior

Computing *F*(*T*) has the secondary benefit of simplifying the procedure of realizing samples from the posterior distribution of *T *as well. This simplification arises from two sources. First, under Jeffreys' prior the maximum *a posteriori *(MAP) parameter estimate of *T *can be used as central point-estimate of *T *that is invariant to reparameterization [41,42]. The MAP point-estimate is similar, conceptually, to the point-estimate given by maximum-likelihood methods. Second, given the central MAP estimate of $\hat{T}(C)$, it is well-known that, asymptotically with the sample-size,

(29) $$\hat{T}\left(C\right)\to N\left(T,{\tilde{F}}^{-1}\left(T\right)\right),$$

where N denotes the multivariate normal and ${\tilde{F}}^{-1}\left(T\right)$ denotes the inverse of the FIM divided by the sample-size used to estimate $\hat{T}$. Frequencies *P *computed from the MAP estimate of *T *are shown in Table 2 and appear very similar to those estimated via relative frequencies and natural parameters. Even though (29) describes an asymptotic relationship, it can be exploited to sample the *exact *posterior of *T *via the Metropolis algorithm [9]. Specifically, the mean expected value of *T*, computed via (13), can be combined with our estimate of *F *via (29) to yield a viable proposal function for Metropolis sampling. With Metropolis sampling, differences between the true and approximate posterior of *T *are eliminated due to the use of rejection sampling. The approximation only affects sampling efficiency, not accuracy: the poorer the approximation, the larger the proportion of rejected samples. In practice, we find that acceptance ratios even as low as 1-10% yield posterior samples more than rapidly enough for practical analysis of large proteins.

### Contrasting Methods

The classical molecular evolution literature suggests two contrasting approaches to the analytical model and method we have described which deserve particular recognition. The first contrast is between *models *of sequence evolution and the second is between established statistical *methods*.

#### Codon Evolution Models

Codon-specific models for classical molecular evolution have been previously described by Goldman *et al*. [46] and Mayrose *et al*. [47]. These models seek to describe the combined effects of codon mutation *and *selection through a continuous-time Markov process by prescribing a constrained form of the infinitesimal Markov generator *Q *for the hypothesized 64-by-64 codon substitution matrix *M*. The principal constraints used enforce the ideas that (a) only single-nucleotide changes may have nonzero rates, and (b) the nonzero substitution rates have a biologically-relevant parameterization. In contrast, unigenic evolution proceeds through multiple rounds of mutation from a *single *ancestor before concluding with a single selective sweep. It therefore describes a process fundamentally different from the combined mutation-plus-selection process approximated by classical molecular evolution models. Toward this end, we describe unigenic evolution by a *discrete-time *Markov transition model *M*. Note that since classical molecular evolution must account for hypothetical unobserved states throughout *continuous *time, it is *necessarily *parameterized by the instantaneous Markov generator *Q*. An immediate consequence of this differing parameterization is that two nucleotide misincorporations per codon per "instantaneous mutation step" necessarily have zero probability under the classical molecular evolution model but *non-negligible *probability under the unigenic evolution model.

Furthermore, even approximate comparisons between the two models are difficult to describe because the combined mutation-plus-selection model of classical molecular evolution is necessarily constrained to satisfy the detail-balance relationship *Q_ij_p_j _*= *Q_ji_p_i_*, where *p *denotes the unique stationary vector of *Q*. In contrast, the stepwise-mutation mechanism of *Taq* misincorporation that drives unigenic evolution implies that detail balance is *not *required nor even desirable.

Thus we conclude that the models and processes describing classical molecular evolution and unigenic evolution are sufficiently different to preclude straightforward comparison.

#### Maximum Likelihood Methods

Both likelihood methods [48] and Bayesian methods have a rich history of use in classical molecular evolution, and the general differences between these approaches have been discussed extensively in both the evolutionary and statistical literature [9]. To a first approximation, likelihood methods are well known to be equivalent to their Bayesian counterparts under the conditions of (a) an asymptotically uniform prior and (b) asymptotically large sample sizes. Under these conditions, the task of parameter estimation is essentially equivalent to the task of hypothesis testing, and different statistical frameworks yield essentially identical inferences.

In unigenic evolution experiments we know *a priori *that nucleotide misincorporation probabilities are very small. Since our hypothesis tests rely on indirectly estimating the probability of very rare events, it is therefore difficult to justify the assumption of "asymptotically large sample size" required by likelihood techniques. Furthermore, it is well known for these types of multinomial-inference problems that uniform priors are generally inappropriate [25,38]. Thus the two key requirements of maximum likelihood theory are not met by our model of unigenic evolution, motivating our decision to use Bayesian methods.

Furthermore, our methods have been developed specifically to test hypotheses about site-specific selection and *not *estimate the strength of selection. Unlike likelihood methods, for Bayesian methods the tasks of "model selection" and "parameter estimation" are often *not *equivalent and can give seemingly-inconsistent inferences without careful analysis (see Kass *et al*. [31,49] for details). We believe that the most direct comparison with previous work can be done in the context of Neyman-Pearson testing [12]. For traditional Neyman-Pearson testing, a critical value of observed nonsynonymous substitutions would be computed for each site based on estimated *Taq* misincorporation frequencies. If fewer nonsynonymous substitutions than that critical value are observed, that site is classified as being under selection. In some sense this procedure "double-dips" the data; on one hand using observations to infer misincorporation frequencies, and on the other using observations to actually classify the site.

In contrast, our Kullback-Leibler-based approach uses the data *only *to test hypotheses at each site, integrating over all possible hypothetical data sets that could have been observed given plausible misincorporation rates. More specific and extensive comparisons between the Kullback-Leibler approach and the Neyman-Pearson approach have been extensively studied in the statistical literature, although they are not especially well known [[26], especially pp. 4-5]. More important than their differences, however, are their similarities. Both compute true-positive/false-negative and true-negative/false-positive classification ratios for the null and alternate hypotheses; they simply use different methodological approaches to do so.

## Software Availability

Software and sample input and output are available from the authors and are also online as Additional Files 6 and 7.

## Competing interests

The authors declare that they have no competing interests.

## Authors' contributions

AF conceived of, designed and tested the statistical methods for this study, and drafted the manuscript. DE designed and BK carried out the molecular studies in the laboratory. DE, LW, and GG participated in the design and validation of the study, its subsequent iterative refinement, and editing the manuscript. All authors read and approved the final manuscript.

## Supplementary Material

Additional file 1 **Homogeneity Tests are Insufficient to Detect Selection**. The necessity of computing the codon mutation frequencies *M *via nucleotide frequencies *P *is shown by the lack of statistical power for determining selection purely by codon-by-codon comparison of unselected and selected clones. **(A) **Using the test for such multinomial homogeneity as given by Wolpert [19], the posterior log_2_-odds-ratio between hypotheses, ≈ -0.4, implies that they are virtually indistinguishable. **(B) **The estimated power of such analysis has a posterior log_2_-odds-ratio of ≈ 0.05 thereby showing the unsuitability of tests for functional selection that rely only on codon-based mutation counts. Of particular significance is that the the M1 start-codon is not discerned in either selected or unselected population, even though it is absolutely required for protein function in the selected clones and absolutely conserved due to the cloning technique in the unselected population. The complete absence of power at M1 and other sites shows the unsuitability of codon homogeneity to serve as evidence of selection. Note that the additive property of log_2_-odds-ratios implies that combining counts for identical codon classes increases the log_2_-odds-ratio only linearly, thereby implying that reasonable power cannot be achieved by codon-class analysis either, for the given sample size. Click here for file

Additional file 2 **The Kronecker Product, Illustrated**. An explicit representation of the Kronecker product *P *⊗ *P*. Since mutations in nucleotide sites are assumed independent, the frequency that nucleotide *j *is mutated to *i *is *p_ij _*. For a second nucleotide, again the frequency that nucleotide *l *is mutated to *k *is *p_kl_*. Therefore, the joint frequency that both mutations occur is *p_ij_p_kl_*. A third Kronecker-multiplication would result in the 64 × 64 matrix *M *= *P *⊗ *P *⊗ *P*. Being given a third mutation of frequency of *p_mn _*yields a final codon mutation frequency of *p_ij_p_kl_p_mn_*. Click here for file

Additional file 3 **Details of the ****GIY-YIG****Domain**. Numerical details of the GIY-YIG motif grey-highlighted in Figure 1. 'EoS' refers to the $R_{j}^{k}$ log-odds ratio, 'Total' is the total (synonymous plus nonsynonymous) number of observed codon mutations, 'NSO' is the observed number of nonsynonymous mutations, and 'NSE' is the expected number of nonsynonymous mutations. All 'expected' values are conditioned on the null hypothesis of 'no selection'. Additional expected nonsynonymous counts for different codons are shown in Additional File 4. Click here for file

Additional file 4 **The Expected Number of Nonsynonymous Misincorporations **Percentiles for the expected number of nonsynonymous mutations under the null hypothesis of 'no selection' for different clone population sample size, given misincorporation frequencies estimated by the unselected population counts shown in Table 1. Of particular importance is the wide range of 'Pr(NS)', the estimated probability of nonsynonymous mutation. This probability ranges from 0.0056 to 0.0633 per codon, an 11.2-fold difference. 'Q02', 'Q50', and 'Q98' represent the 2%, 50%, and 98% binomial percentiles, respectively, indicating that the observed number of nonsynonymous mutations under *H*_0 _is 96% likely to be within the indicated range. Codons resistant to nonsynonymous mutation, such as alanine and glycine, show obvious non-normality for even between 200-500 sequenced clones. Click here for file

Additional file 5 **The Effect of Sample Size for ****I-Bmol**. The effect of differing selected and unselected clone population sample sizes on the power of inference. Subsamples of 5, 10, 20, 40, and 87 (all) clone populations were analyzed as per Figure 1 and shown using identical axis scales, with the 87-87 plot therefore identical to Figure 1. All populations are *subset inclusive*, meaning that the 10-sample subset contained all sequences of the 5-sample subset, and so on. Approximate nucleotide misincorporation frequencies can be estimated by dividing the counts shown in Table 1 as appropriate. We note that even using only 5/87 unselected clones to estimate parameter matrix *T *resulted in qualitatively similar EoS values (red) for all 87-clone selected populations. Unselected clones were critical, however, in estimating false-positive (blue) rates, with all 87 unselected clones being required to detect the methionine start-signal. Click here for file

Additional file 6 **Software Package**. Source code for an R-Project software package that we call 'unigenic'. The code has been tested on Mac OS 10.5 and recent versions of Linux-based operating systems, and requires that R ≥ 2.9.1 and a modern Fortran95 compiler be available. For help installing R packages, see http://cran.r-project.org/doc/manuals/R-admin.html#Installing-packages. Click here for file

Additional file 7 **Sample Input and Output**. Sample input, output, and driver files for the given software package. Click here for file
