# Supplementary material for: Estimating the evidence of selection and the reliability of inference in unigenic evolution
Source: Algorithms Mol Biol. 2010 Nov 8;5:35. doi: 10.1186/1748-7188-5-35 (PMC2994857; doi:10.1186/1748-7188-5-35)
Supplement: Additional file 1 — Homogeneity Tests are Insufficient to Detect Selection. The necessity of computing the codon mutation frequencies M via nucleotide frequencies P is shown by the lack of statistical power for determining selection purely by codon-by-codon comparison of unselected and selected clones. (A) Using the test for such multinomial homogeneity as given by Wolpert [19], the posterior log2-odds-ratio between hypotheses, ≈ -0.4, implies that they are virtually indistinguishable. (B) The estimated power of such analysis has a posterior log2-odds-ratio of ≈ 0.05 thereby showing the unsuitability of tests for functional selection that rely only on codon-based mutation counts. Of particular significance is that the the M1 start-codon is not discerned in either selected or unselected population, even though it is absolutely required for protein function in the selected clones and absolutely conserved due to the cloning technique in the unselected population. The complete absence of power at M1 and other sites shows the unsuitability of codon homogeneity to serve as evidence of selection. Note that the additive property of log2-odds-ratios implies that combining counts for identical codon classes increases the log2-odds-ratio only linearly, thereby implying that reasonable power cannot be achieved by codon-class analysis either, for the given sample size. [file 1748-7188-5-35-S1.PDF]

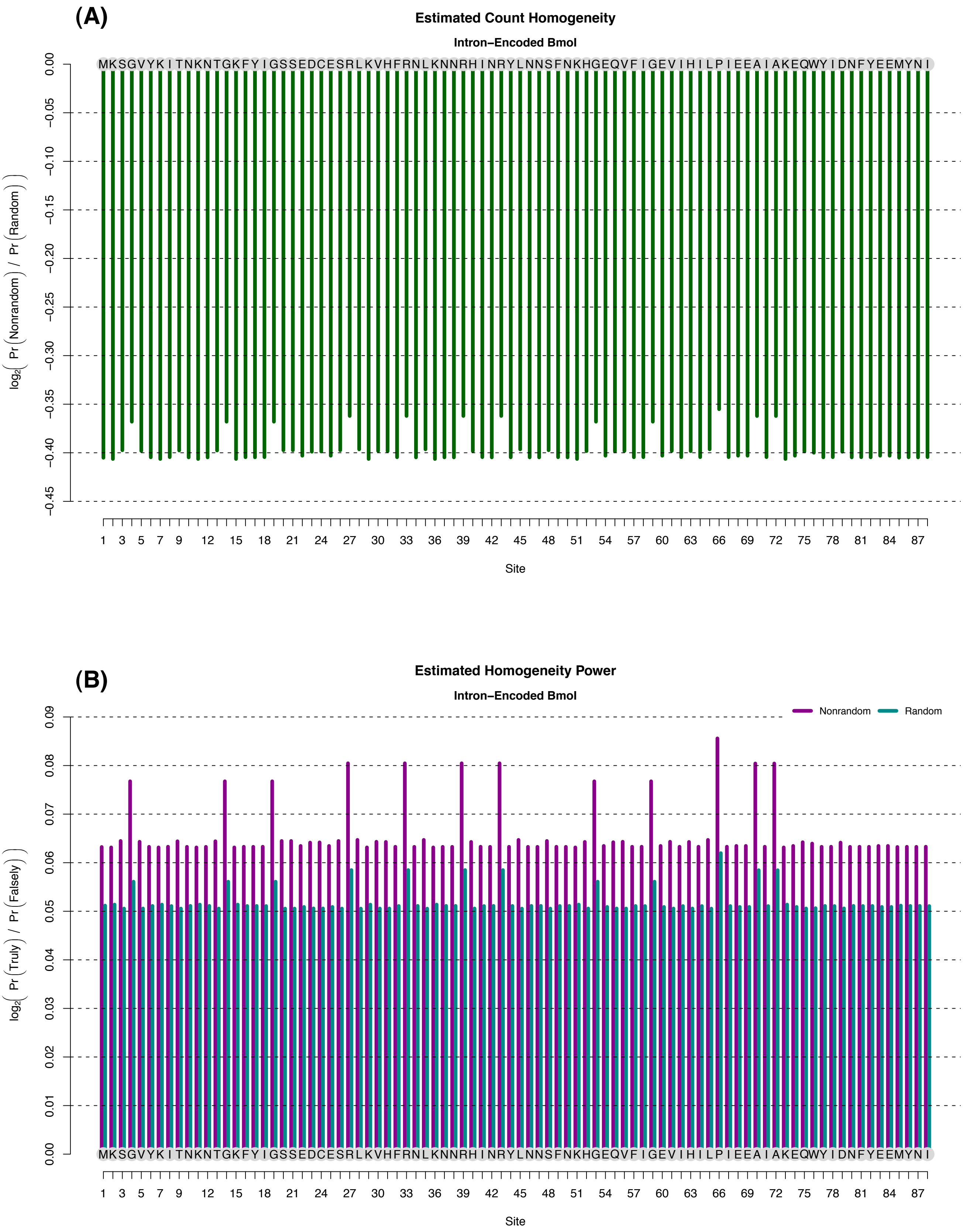

(A)
Estimated Count Homogeneity
Intron–Encoded BmoI
log2(Pr(Nonrandom) / Pr(Random))
0.00 −0.05 −0.10 −0.15 −0.20 −0.25 −0.30 −0.35 −0.40 −0.45
MKSGVYKITNKNTGKFYIGSSEDCESRLKVHFRNLKNNRHINRYLNNSFNKHGEQVFIGEVIHILPIEEAIAKEQWYIDNFYEEMYNI
1 3 5 7 9 12 15 18 21 24 27 30 33 36 39 42 45 48 51 54 57 60 63 66 69 72 75 78 81 84 87
Site
(B)
Estimated Homogeneity Power
Intron–Encoded BmoI
Nonrandom
Random
log2(Pr(Truly) / Pr(Falsely))
0.09 0.08 0.07 0.06 0.05 0.04 0.03 0.02 0.01 0.00
MKSGVYKITNKNTGKFYIGSSEDCESRLKVHFRNLKNNRHINRYLNNSFNKHGEQVFIGEVIHILPIEEAIAKEQWYIDNFYEEMYNI
1 3 5 7 9 12 15 18 21 24 27 30 33 36 39 42 45 48 51 54 57 60 63 66 69 72 75 78 81 84 87
Site
