# Supplementary material for: Estimating the evidence of selection and the reliability of inference in unigenic evolution
Source: Algorithms Mol Biol. 2010 Nov 8;5:35. doi: 10.1186/1748-7188-5-35 (PMC2994857; doi:10.1186/1748-7188-5-35)
Supplement: Additional file 4 — The Expected Number of Nonsynonymous Misincorporations Percentiles for the expected number of nonsynonymous mutations under the null hypothesis of 'no selection' for different clone population sample size, given misincorporation frequencies estimated by the unselected population counts shown in Table 1. Of particular importance is the wide range of 'Pr(NS)', the estimated probability of nonsynonymous mutation. This probability ranges from 0.0056 to 0.0633 per codon, an 11.2-fold difference. 'Q02', 'Q50', and 'Q98' represent the 2%, 50%, and 98% binomial percentiles, respectively, indicating that the observed number of nonsynonymous mutations under H0 is 96% likely to be within the indicated range. Codons resistant to nonsynonymous mutation, such as alanine and glycine, show obvious non-normality for even between 200-500 sequenced clones. [file 1748-7188-5-35-S4.PDF]

			10 Clones			20 Clones			50 Clones			100 Clones			200 Clones			500 Clones		
Amino	Codon	Pr(NS)	Q02	Q50	Q98	Q02	Q50	Q98	Q02	Q50	Q98	Q02	Q50	Q98	Q02	Q50	Q98	Q02	Q50	Q98
*	TAA	0.0518	0	0	2	0	1	3	0	2	6	1	5	10	4	10	17	16	26	37
*	TAG	0.0514	0	0	2	0	1	3	0	2	6	1	5	10	4	10	17	16	26	36
*	TGA	0.0514	0	0	2	0	1	3	0	2	6	1	5	10	4	10	17	16	26	36
A	GCA	0.0070	0	0	1	0	0	1	0	0	2	0	1	3	0	1	4	0	3	8
A	GCC	0.0070	0	0	1	0	0	1	0	0	2	0	1	3	0	1	4	0	3	8
A	GCG	0.0070	0	0	1	0	0	1	0	0	2	0	1	3	0	1	4	0	3	8
A	GCT	0.0070	0	0	1	0	0	1	0	0	2	0	1	3	0	1	4	0	3	8
C	TGC	0.0302	0	0	2	0	0	3	0	1	4	0	3	7	2	6	11	8	15	23
C	TGT	0.0420	0	0	2	0	1	3	0	2	5	1	4	9	3	8	15	12	21	31
D	GAC	0.0307	0	0	2	0	0	3	0	1	4	0	3	7	2	6	12	8	15	24
D	GAT	0.0424	0	0	2	0	1	3	0	2	5	1	4	9	3	8	15	12	21	31
E	GAA	0.0429	0	0	2	0	1	3	0	2	5	1	4	9	3	8	15	13	21	31
E	GAG	0.0312	0	0	2	0	0	3	0	1	4	0	3	7	2	6	12	8	15	24
F	TTC	0.0504	0	0	2	0	1	3	0	2	6	1	5	10	4	10	17	16	25	36
F	TTT	0.0619	0	0	3	0	1	4	0	3	7	2	6	12	6	12	20	20	31	42
G	GGA	0.0084	0	0	1	0	0	1	0	0	2	0	1	3	0	1	5	1	4	9
G	GGC	0.0084	0	0	1	0	0	1	0	0	2	0	1	3	0	1	5	1	4	9
G	GGG	0.0084	0	0	1	0	0	1	0	0	2	0	1	3	0	1	5	1	4	9
G	GGT	0.0084	0	0	1	0	0	1	0	0	2	0	1	3	0	1	5	1	4	9
H	CAC	0.0293	0	0	2	0	0	2	0	1	4	0	3	7	2	6	11	7	15	23
H	CAT	0.0411	0	0	2	0	1	3	0	2	5	1	4	9	3	8	14	12	20	30
I	ATA	0.0608	0	0	2	0	1	4	0	3	7	2	6	11	6	12	20	20	30	42
I	ATC	0.0499	0	0	2	0	1	3	0	2	6	1	5	10	4	10	17	15	25	35
I	ATT	0.0515	0	0	2	0	1	3	0	2	6	1	5	10	4	10	17	16	26	36
K	AAA	0.0633	0	0	3	0	1	4	0	3	7	2	6	12	6	13	20	21	32	43
K	AAG	0.0519	0	0	2	0	1	3	0	2	6	1	5	10	4	10	17	16	26	37
L	CTA	0.0262	0	0	2	0	0	2	0	1	4	0	2	6	1	5	10	6	13	21
L	CTC	0.0277	0	0	2	0	0	2	0	1	4	0	3	7	1	5	11	7	14	22
L	CTG	0.0261	0	0	2	0	0	2	0	1	4	0	2	6	1	5	10	6	13	21
L	CTT	0.0277	0	0	2	0	0	2	0	1	4	0	3	7	1	5	11	7	14	22
L	TTA	0.0511	0	0	2	0	1	3	0	2	6	1	5	10	4	10	17	16	25	36
L	TTG	0.0396	0	0	2	0	1	3	0	2	5	1	4	8	3	8	14	11	20	29
M	ATG	0.0537	0	0	2	0	1	4	0	3	6	1	5	10	5	11	18	17	27	38
N	AAC	0.0513	0	0	2	0	1	3	0	2	6	1	5	10	4	10	17	16	26	36
N	AAT	0.0628	0	0	3	0	1	4	0	3	7	2	6	12	6	12	20	21	31	43
P	CCA	0.0056	0	0	1	0	0	1	0	0	2	0	0	2	0	1	4	0	3	7
P	CCC	0.0056	0	0	1	0	0	1	0	0	2	0	0	2	0	1	4	0	3	7
P	CCG	0.0056	0	0	1	0	0	1	0	0	2	0	0	2	0	1	4	0	3	7
P	CCT	0.0056	0	0	1	0	0	1	0	0	2	0	0	2	0	1	4	0	3	7
Q	CAA	0.0416	0	0	2	0	1	3	0	2	5	1	4	9	3	8	15	12	21	30
Q	CAG	0.0299	0	0	2	0	0	3	0	1	4	0	3	7	2	6	11	8	15	23
R	AGA	0.0402	0	0	2	0	1	3	0	2	5	1	4	8	3	8	14	12	20	30
R	AGG	0.0286	0	0	2	0	0	2	0	1	4	0	3	7	1	6	11	7	14	22
R	CGA	0.0060	0	0	1	0	0	1	0	0	2	0	0	3	0	1	4	0	3	7
R	CGC	0.0070	0	0	1	0	0	1	0	0	2	0	1	3	0	1	4	0	3	8
R	CGG	0.0060	0	0	1	0	0	1	0	0	2	0	0	3	0	1	4	0	3	7
R	CGT	0.0070	0	0	1	0	0	1	0	0	2	0	1	3	0	1	4	0	3	8
S	AGC	0.0307	0	0	2	0	0	3	0	1	4	0	3	7	2	6	12	8	15	24
S	AGT	0.0424	0	0	2	0	1	3	0	2	5	1	4	9	3	8	15	12	21	31
S	TCA	0.0277	0	0	2	0	0	2	0	1	4	0	3	7	1	5	11	7	14	22
S	TCC	0.0277	0	0	2	0	0	2	0	1	4	0	3	7	1	5	11	7	14	22
S	TCG	0.0277	0	0	2	0	0	2	0	1	4	0	3	7	1	5	11	7	14	22
S	TCT	0.0277	0	0	2	0	0	2	0	1	4	0	3	7	1	5	11	7	14	22
T	ACA	0.0282	0	0	2	0	0	2	0	1	4	0	3	7	1	5	11	7	14	22
T	ACC	0.0282	0	0	2	0	0	2	0	1	4	0	3	7	1	5	11	7	14	22
T	ACG	0.0282	0	0	2	0	0	2	0	1	4	0	3	7	1	5	11	7	14	22
T	ACT	0.0282	0	0	2	0	0	2	0	1	4	0	3	7	1	5	11	7	14	22
V	GTA	0.0290	0	0	2	0	0	2	0	1	4	0	3	7	2	6	11	7	14	23
V	GTC	0.0290	0	0	2	0	0	2	0	1	4	0	3	7	2	6	11	7	14	23
V	GTG	0.0290	0	0	2	0	0	2	0	1	4	0	3	7	2	6	11	7	14	23
V	GTT	0.0290	0	0	2	0	0	2	0	1	4	0	3	7	2	6	11	7	14	23
W	TGG	0.0331	0	0	2	0	0	3	0	1	5	0	3	7	2	6	12	9	16	25
Y	TAC	0.0509	0	0	2	0	1	3	0	2	6	1	5	10	4	10	17	16	25	36
Y	TAT	0.0624	0	0	3	0	1	4	0	3	7	2	6	12	6	12	20	21	31	43
