# Supplementary figures and images for: Estimating the evidence of selection and the reliability of inference in unigenic evolution
Source: Algorithms Mol Biol. 2010 Nov 8;5:35. doi: 10.1186/1748-7188-5-35 (PMC2994857; doi:10.1186/1748-7188-5-35)

Number of *Unselected* Clones

Number of *Selected* Clones

5

10

20

40

87

5

10

20

40

87

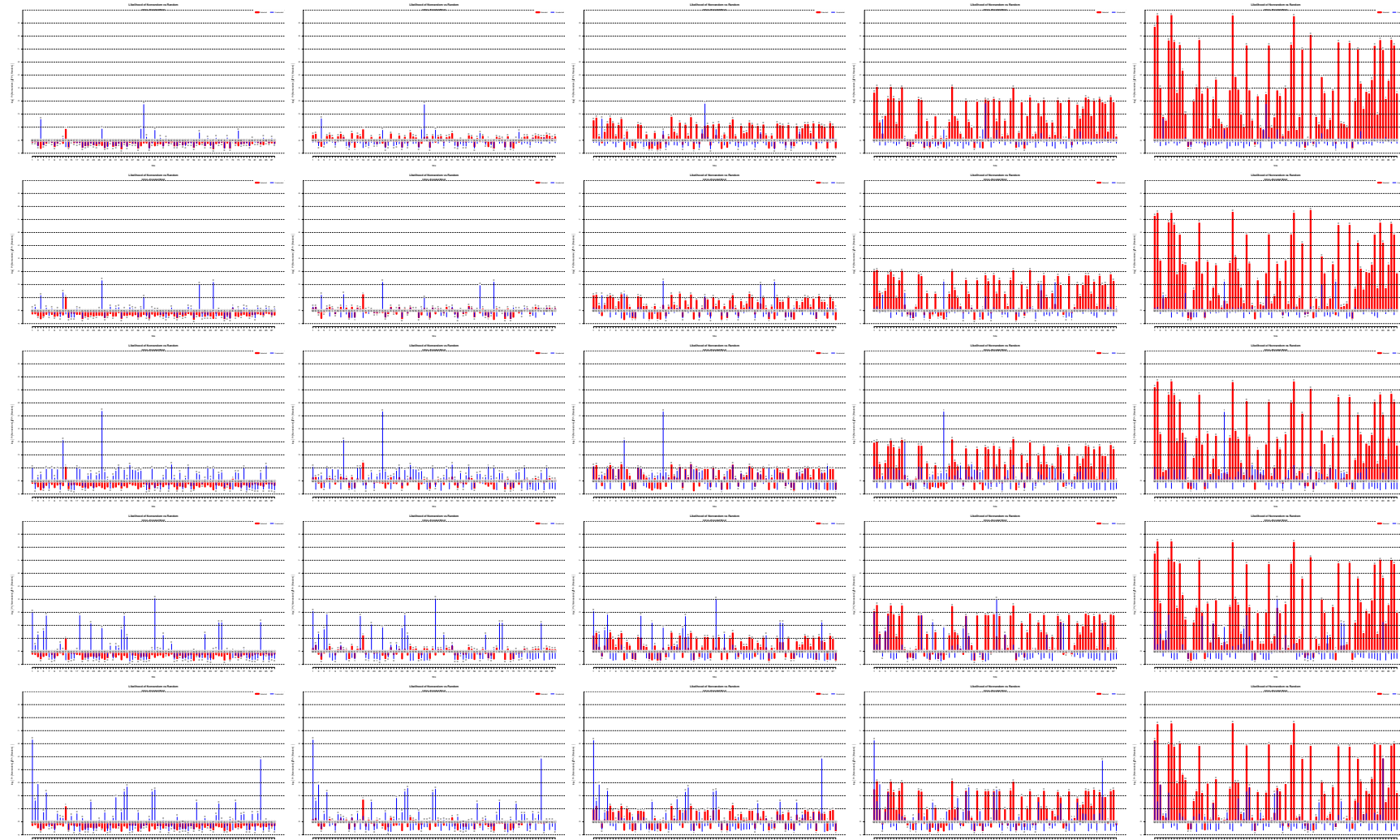

Supplement: Additional file 5 — The Effect of Sample Size for I-Bmol. The effect of differing selected and unselected clone population sample sizes on the power of inference. Subsamples of 5, 10, 20, 40, and 87 (all) clone populations were analyzed as per Figure 1 and shown using identical axis scales, with the 87-87 plot therefore identical to Figure 1. All populations are subset inclusive, meaning that the 10-sample subset contained all sequences of the 5-sample subset, and so on. Approximate nucleotide misincorporation frequencies can be estimated by dividing the counts shown in Table 1 as appropriate. We note that even using only 5/87 unselected clones to estimate parameter matrix T resulted in qualitatively similar EoS values (red) for all 87-clone selected populations. Unselected clones were critical, however, in estimating false-positive (blue) rates, with all 87 unselected clones being required to detect the methionine start-signal. [file 1748-7188-5-35-S5.PDF]

# Frequency of Multiple Substitutions

Intron-Encoded Bmol

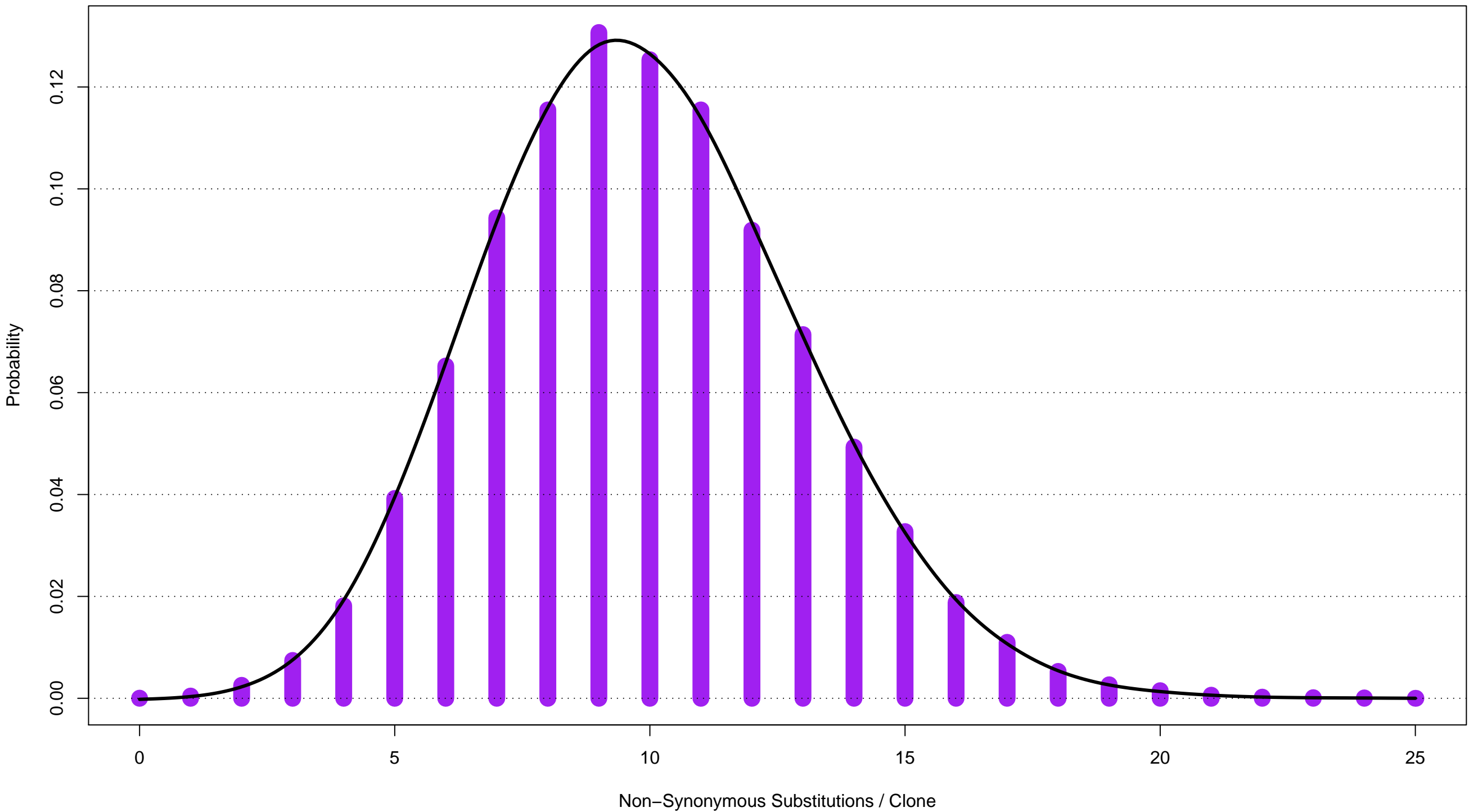

Supplement: Additional file 7 — Sample Input and Output. Sample input, output, and driver files for the given software package. [file 1748-7188-5-35-S7.ZIP › unigenic_example/edgell.pcr/by_sfreq.pdf]

# Frequency of Multiple Substitutions

Intron-Encoded Bmol

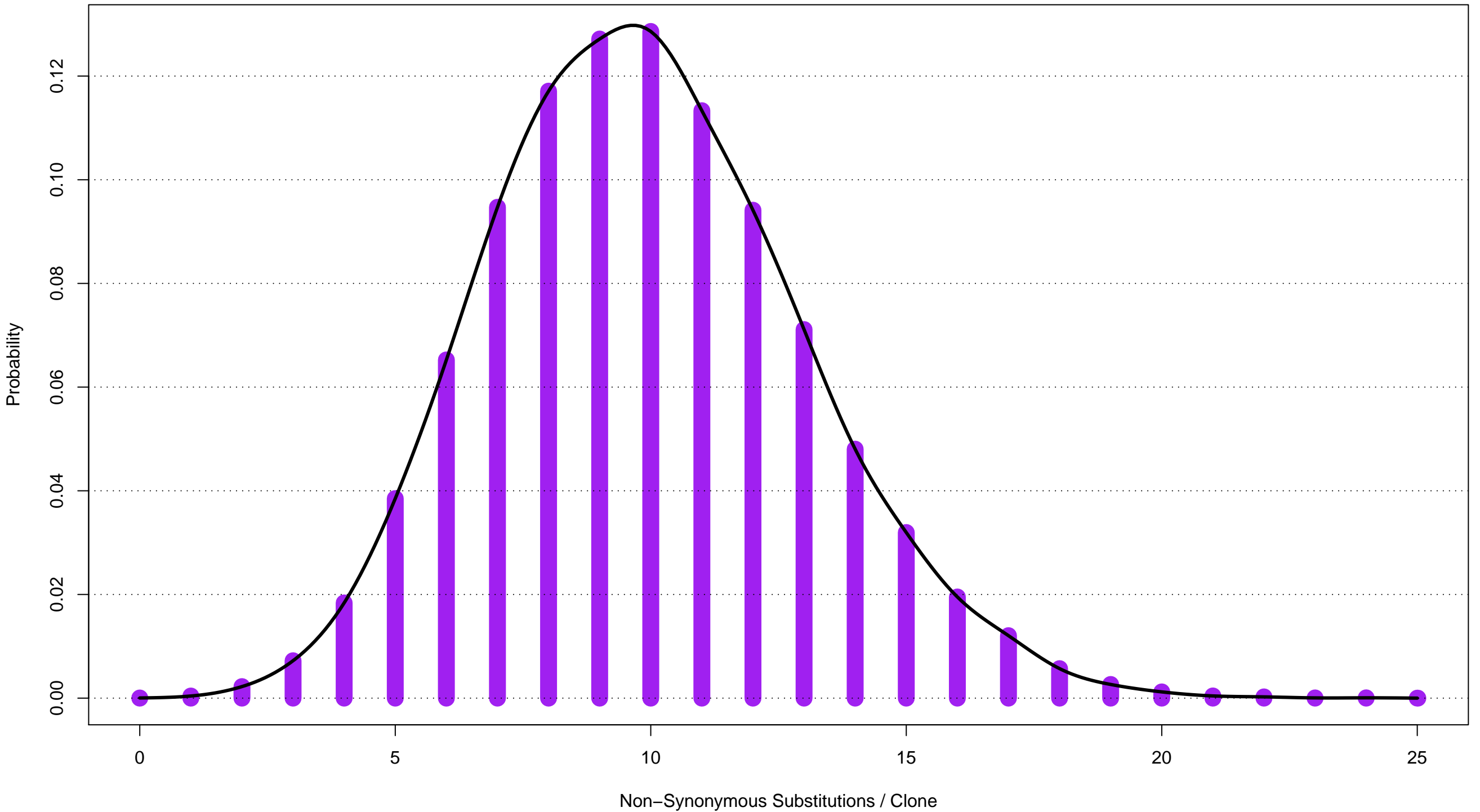

Supplement: Additional file 7 — Sample Input and Output. Sample input, output, and driver files for the given software package. [file 1748-7188-5-35-S7.ZIP › unigenic_example/edgell.pol/by_sfreq.pdf]
