# Supplementary material for: Estimating the evidence of selection and the reliability of inference in unigenic evolution
Source: Algorithms Mol Biol. 2010 Nov 8;5:35. doi: 10.1186/1748-7188-5-35 (PMC2994857; doi:10.1186/1748-7188-5-35)
Supplement: Additional file 7 — Sample Input and Output. Sample input, output, and driver files for the given software package. [file 1748-7188-5-35-S7.ZIP › unigenic_example/edgell.pcr/by_site_llr.pdf]

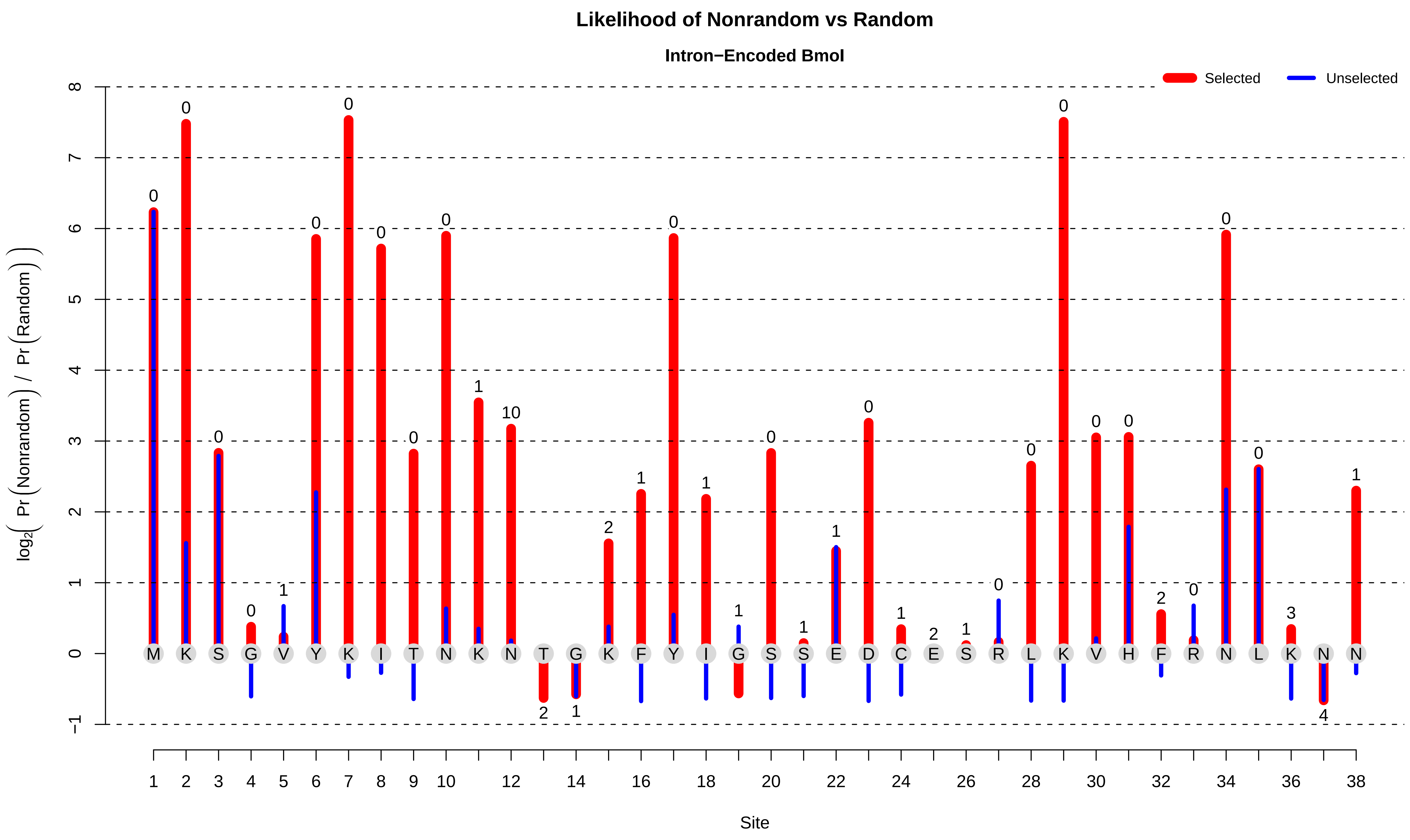

Likelihood of Nonrandom vs Random
Intron−Encoded BmoI
Selected
Unselected
log2(Pr(Nonrandom) / Pr(Random))
Site
M K S G V Y K I T N K N T G K F Y I G S S E D C E S R L K V H F R N L K N N
0 0 0 0 1 0 0 0 0 0 1 10 2 1 2 1 0 1 1 0 1 1 0 1 2 1 0 0 0 0 0 2 0 0 0 3 4 1

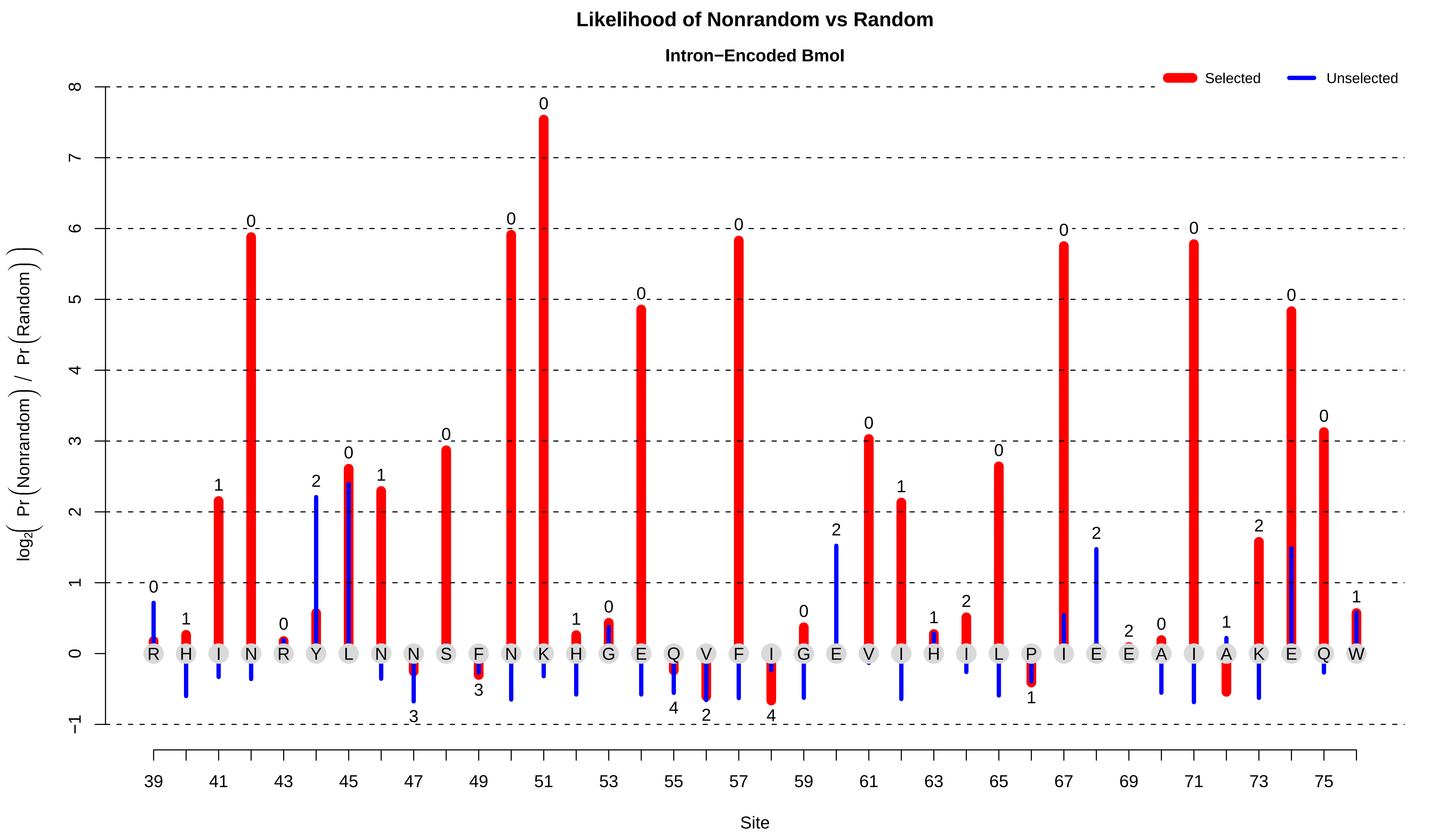

Likelihood of Nonrandom vs Random
Intron−Encoded BmoI
Selected
Unselected
log2(Pr(Nonrandom) / Pr(Random))
Site

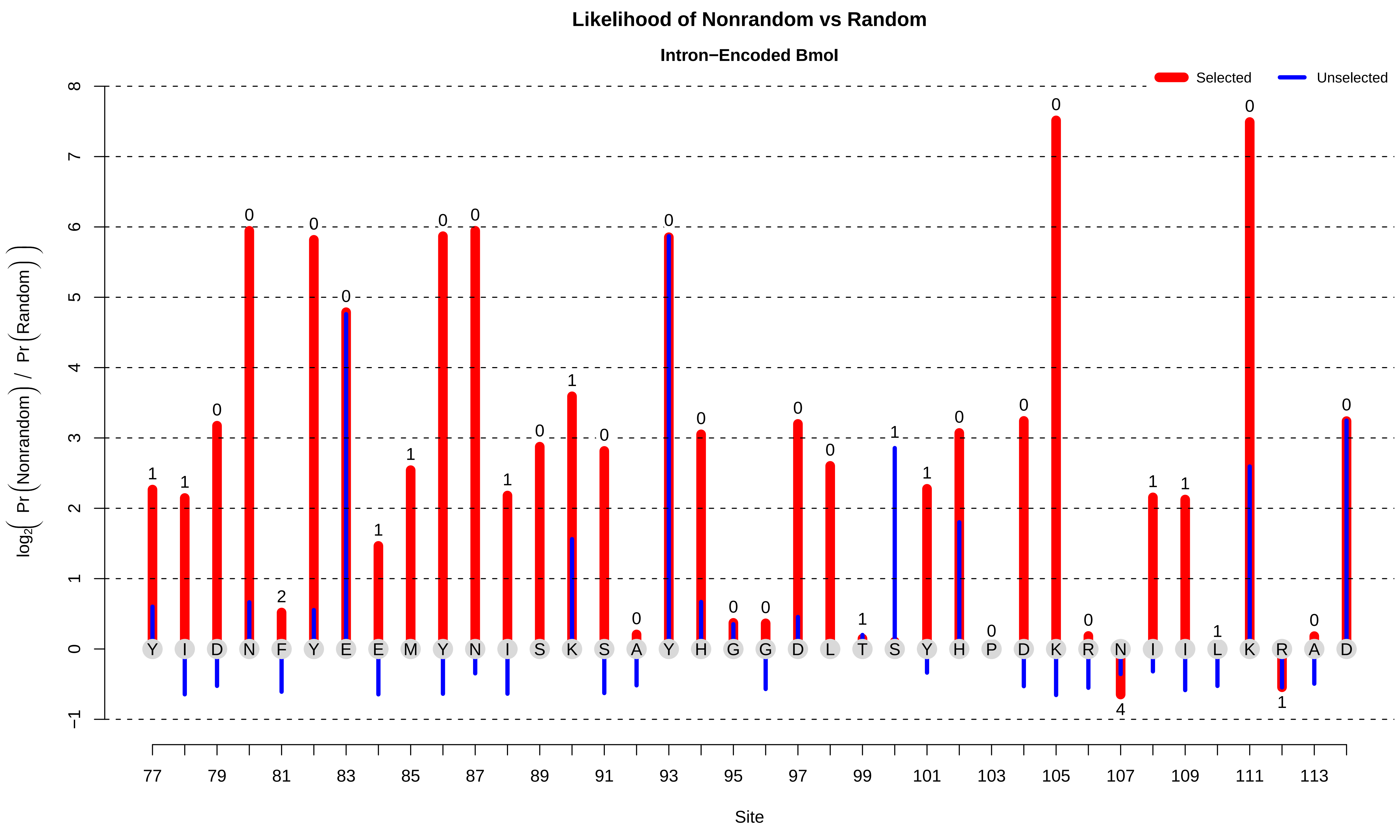

Likelihood of Nonrandom vs Random
Intron−Encoded BmoI
Selected
Unselected
log2(Pr(Nonrandom) / Pr(Random))
Site
Y I D N F Y E E M Y N I S K S A Y H G G D L T S Y H P D K R N I I L K R A D
77 79 81 83 85 87 89 91 93 95 97 99 101 103 105 107 109 111 113

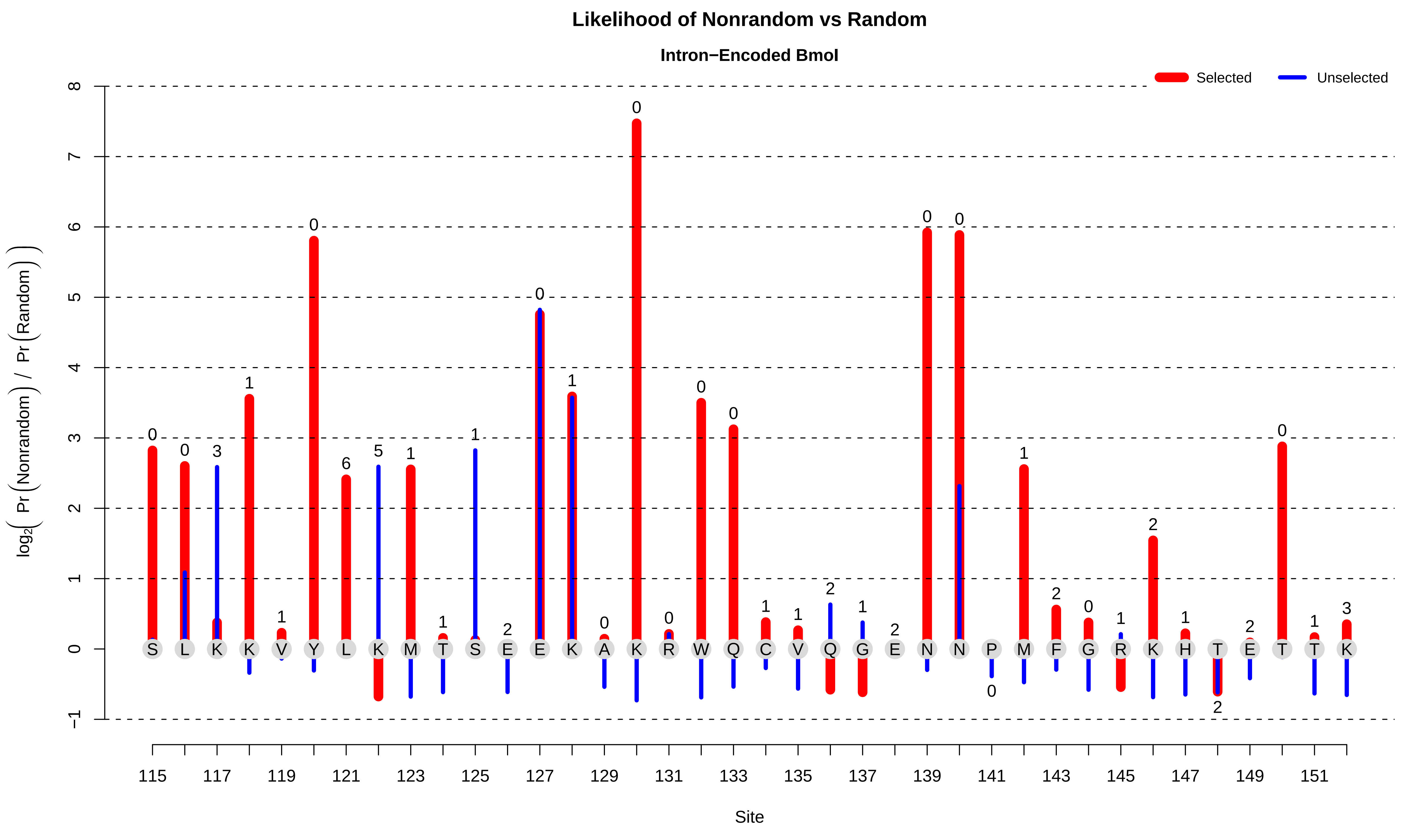

Likelihood of Nonrandom vs Random
Intron−Encoded BmoI
Selected
Unselected
log2(Pr(Nonrandom) / Pr(Random))
Site
S L K K V Y L K M T S E E K A K R W Q C V Q G E N N P M F G R K H T E T T K
115 117 119 121 123 125 127 129 131 133 135 137 139 141 143 145 147 149 151

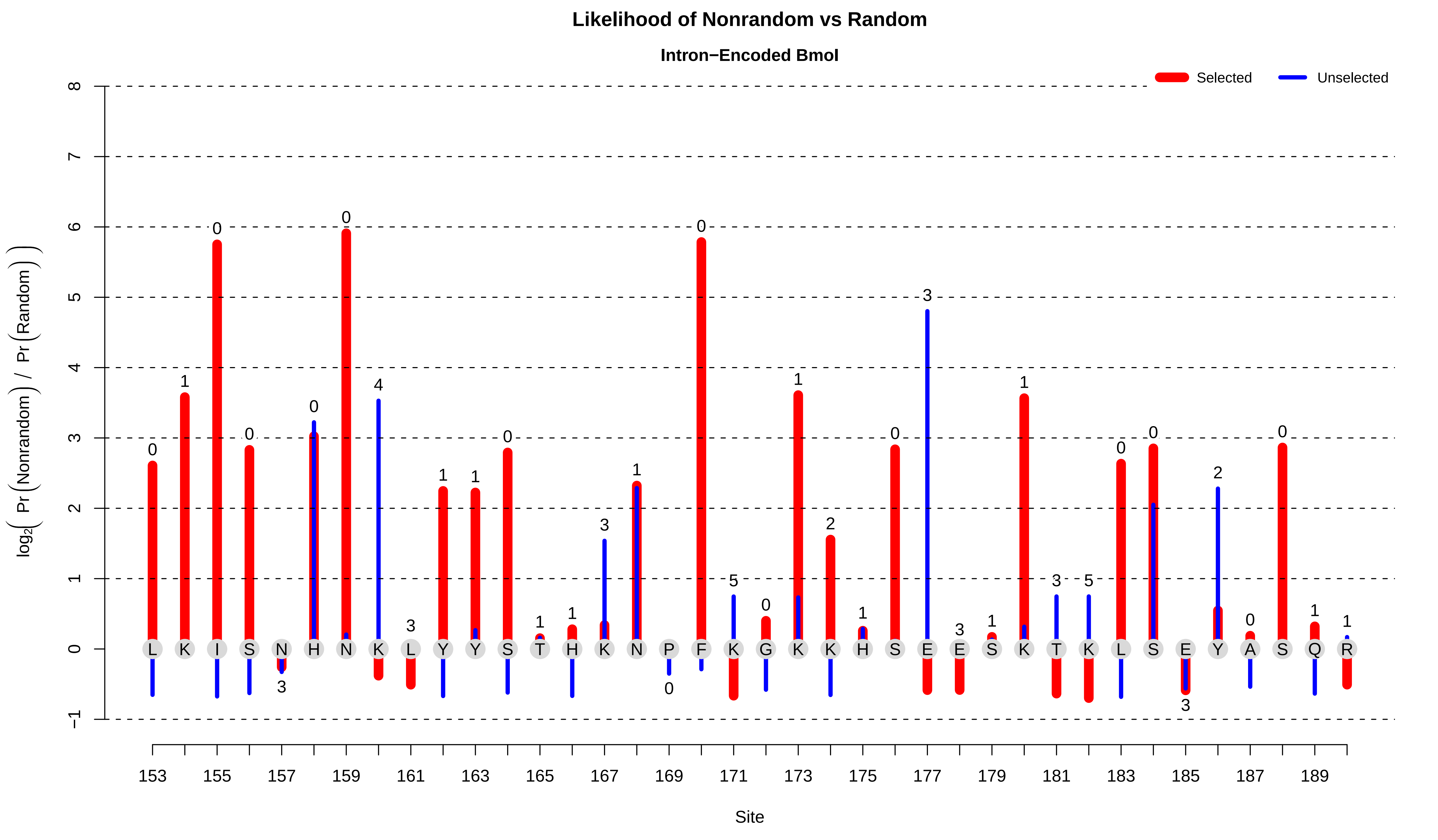

Likelihood of Nonrandom vs Random
Intron−Encoded BmoI
Selected
Unselected
log2(Pr(Nonrandom) / Pr(Random))
Site

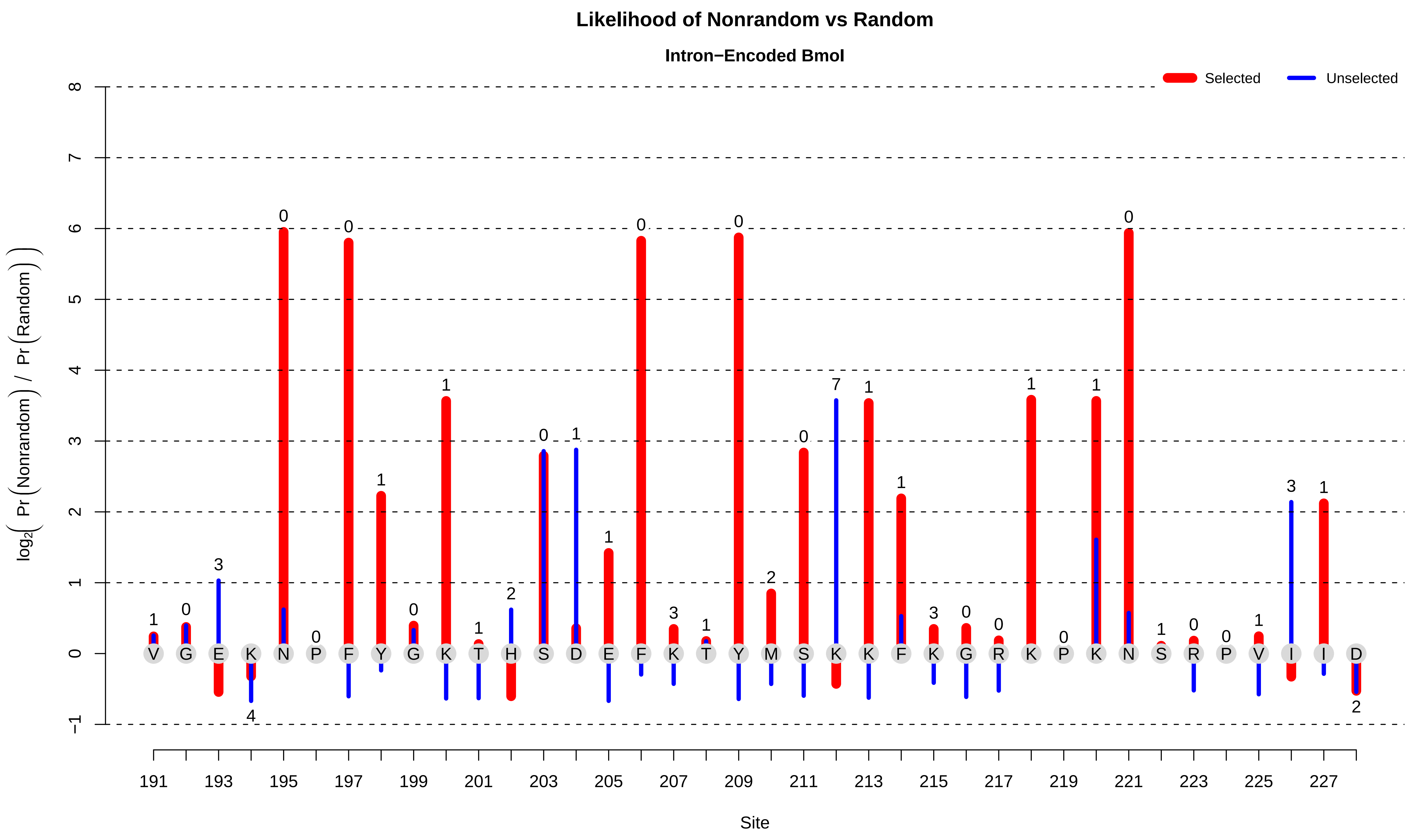

Likelihood of Nonrandom vs Random
Intron−Encoded BmoI
Selected
Unselected
log2(Pr(Nonrandom) / Pr(Random))
Site
V G E K N P F Y G K T H S D E F K T Y M S K K F K G R K P K N S R P V I I D
191 193 195 197 199 201 203 205 207 209 211 213 215 217 219 221 223 225 227

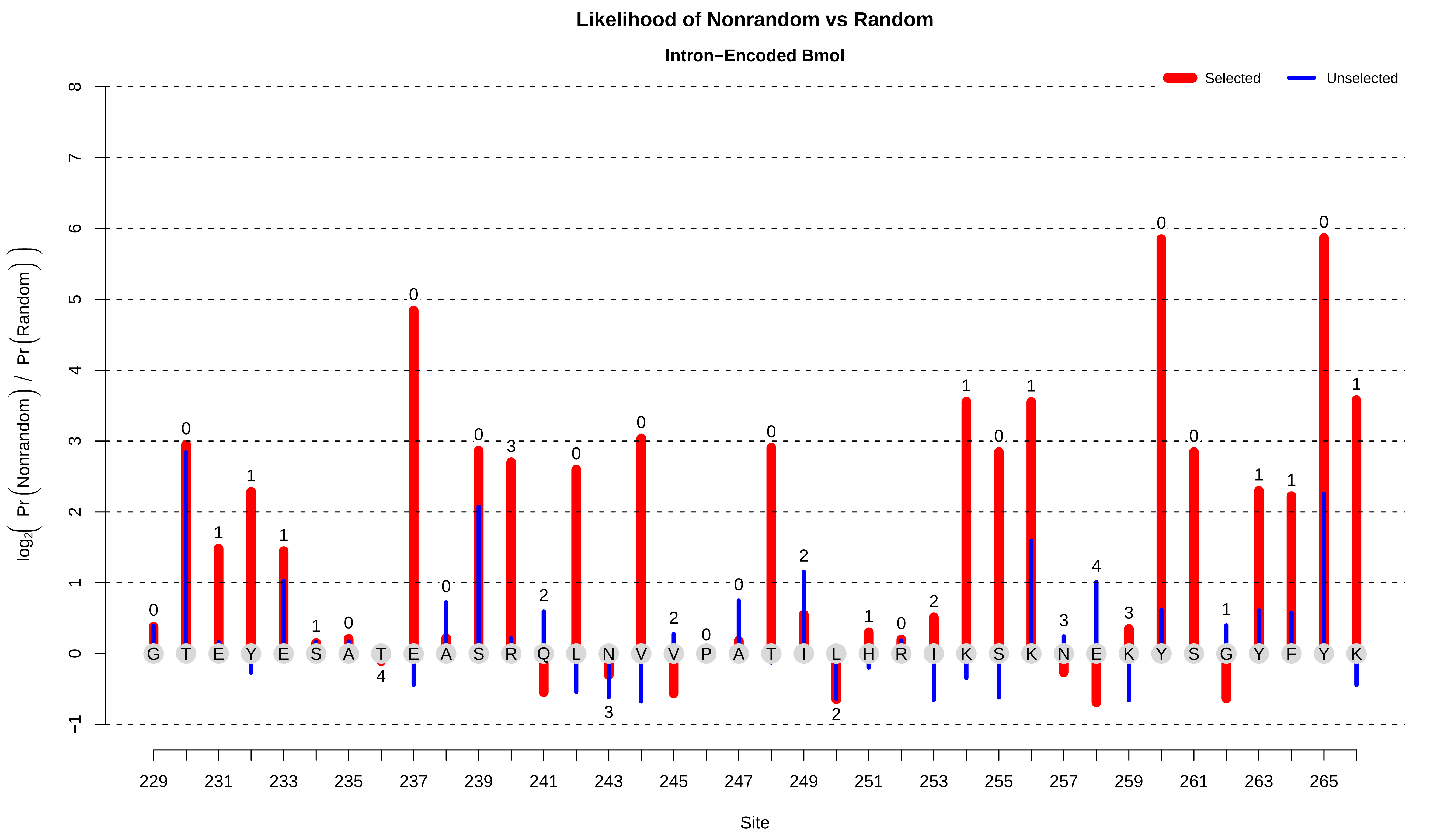

Likelihood of Nonrandom vs Random
Intron−Encoded BmoI
Selected
Unselected
log2(Pr(Nonrandom) / Pr(Random))
Site
G T E Y E S A T E A S R Q L N V V P A T I L H R I K S K N E K Y S G Y F Y K
229 231 233 235 237 239 241 243 245 247 249 251 253 255 257 259 261 263 265
