# Supplementary material for: Estimating the evidence of selection and the reliability of inference in unigenic evolution
Source: Algorithms Mol Biol. 2010 Nov 8;5:35. doi: 10.1186/1748-7188-5-35 (PMC2994857; doi:10.1186/1748-7188-5-35)
Supplement: Additional file 7 — Sample Input and Output. Sample input, output, and driver files for the given software package. [file 1748-7188-5-35-S7.ZIP › unigenic_example/edgell.pcr/by_struct.pdf]

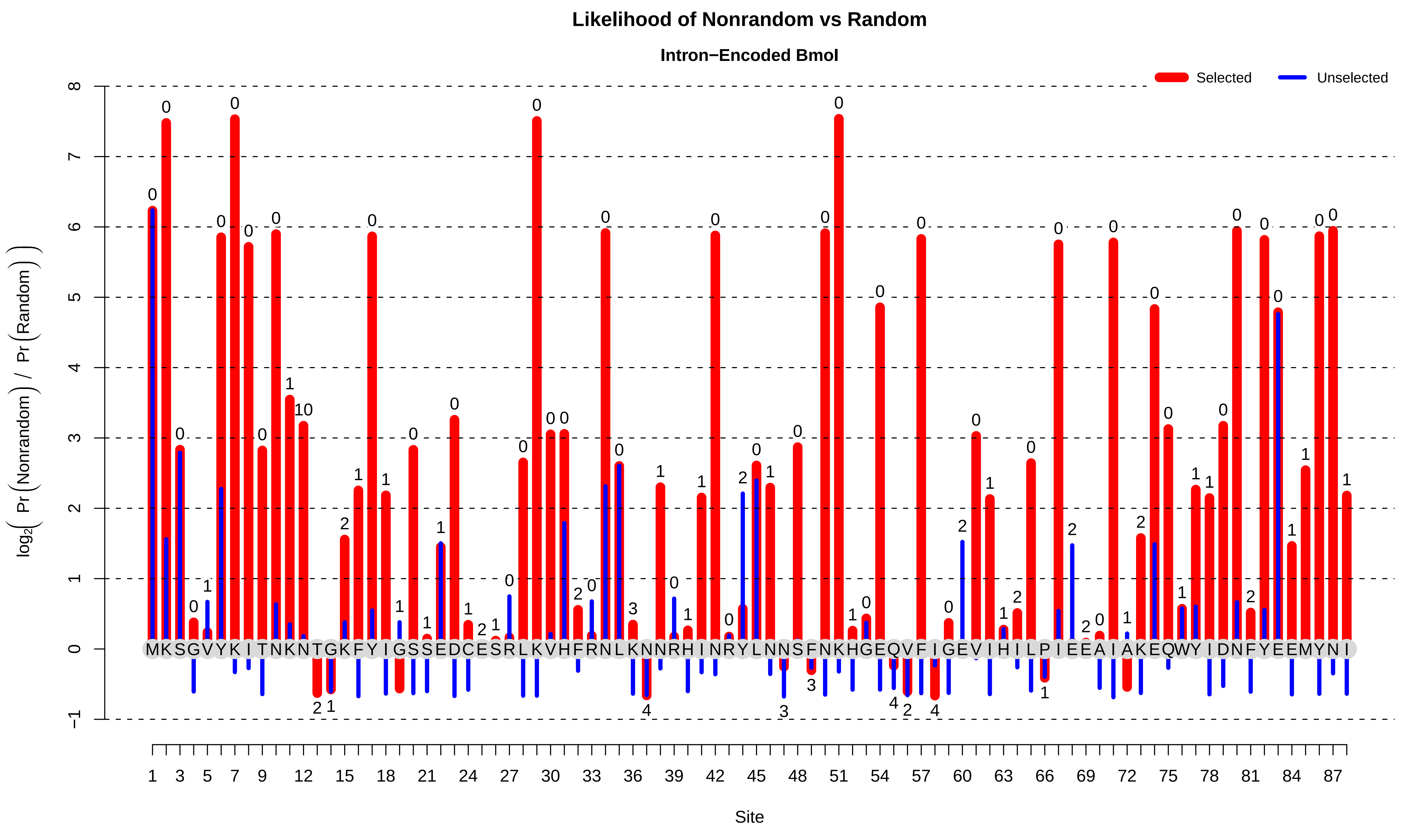

Likelihood of Nonrandom vs Random
Intron−Encoded BmoI
Selected
Unselected
$\log_2\left(\Pr\left(\text{Nonrandom}\right) / \Pr\left(\text{Random}\right)\right)$
MKSGVYKITNKNTGKFYIGSSEDCESRLKVHFRNLKNNRHINRYLNNSFNKHGEQVFIGEVIHILPIEEAIAKEQWYIDNFYEEMYNI
Site

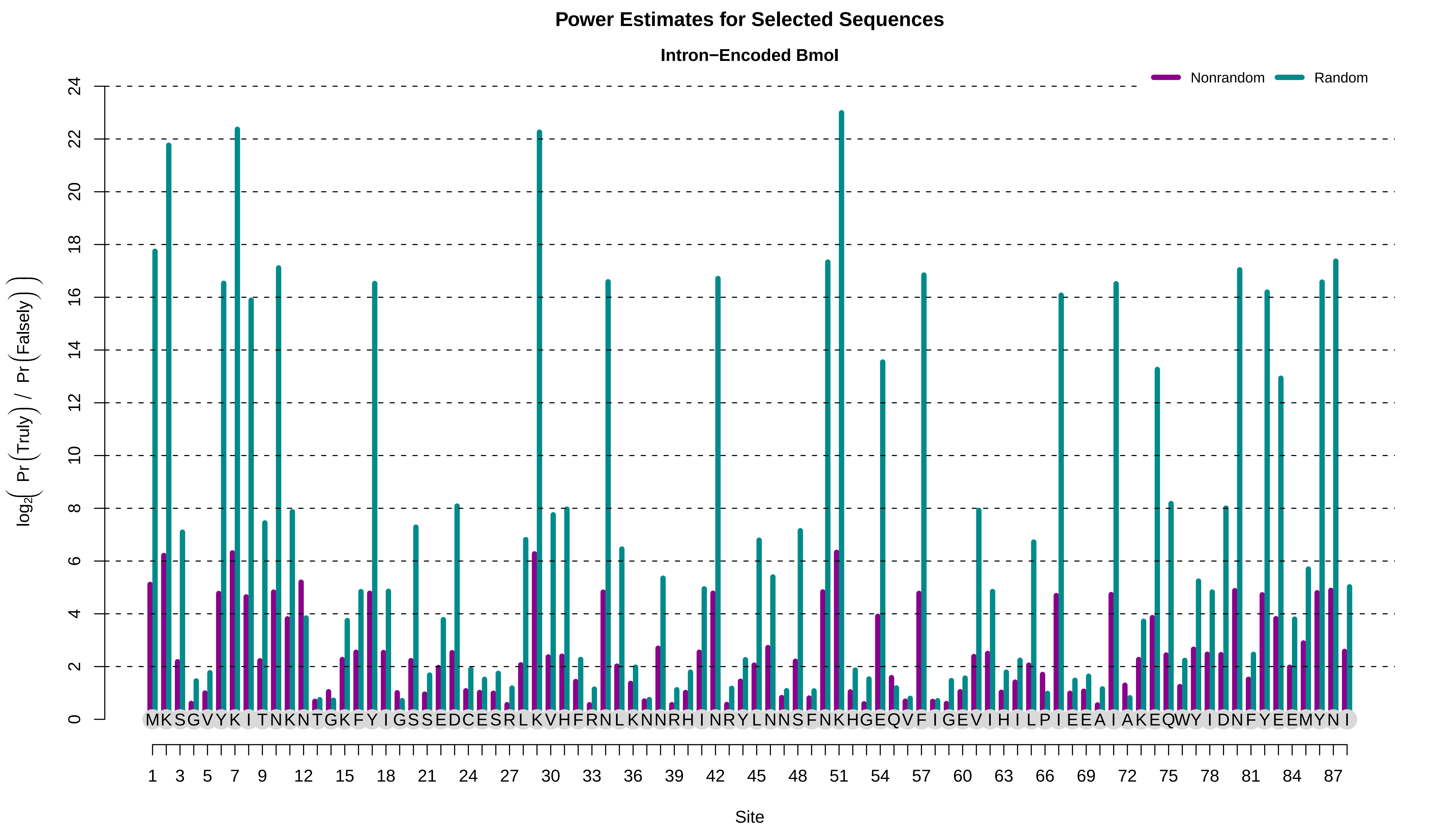
Power Estimates for Selected Sequences
Intron−Encoded BmoI
Nonrandom
Random
$\log_2\left(\Pr\left(\text{Truly}\right) / \Pr\left(\text{Falsely}\right)\right)$
0 2 4 6 8 10 12 14 16 18 20 22 24
M K S G V Y K I T N K N T G K F Y I G S S E D C E S R L K V H F R N L K N N R H I N R Y L N N S F N K H G E Q V F I G E V I H I L P I E E A I A K E Q W Y I D N F Y E E M Y N I
1 3 5 7 9 12 15 18 21 24 27 30 33 36 39 42 45 48 51 54 57 60 63 66 69 72 75 78 81 84 87
Site

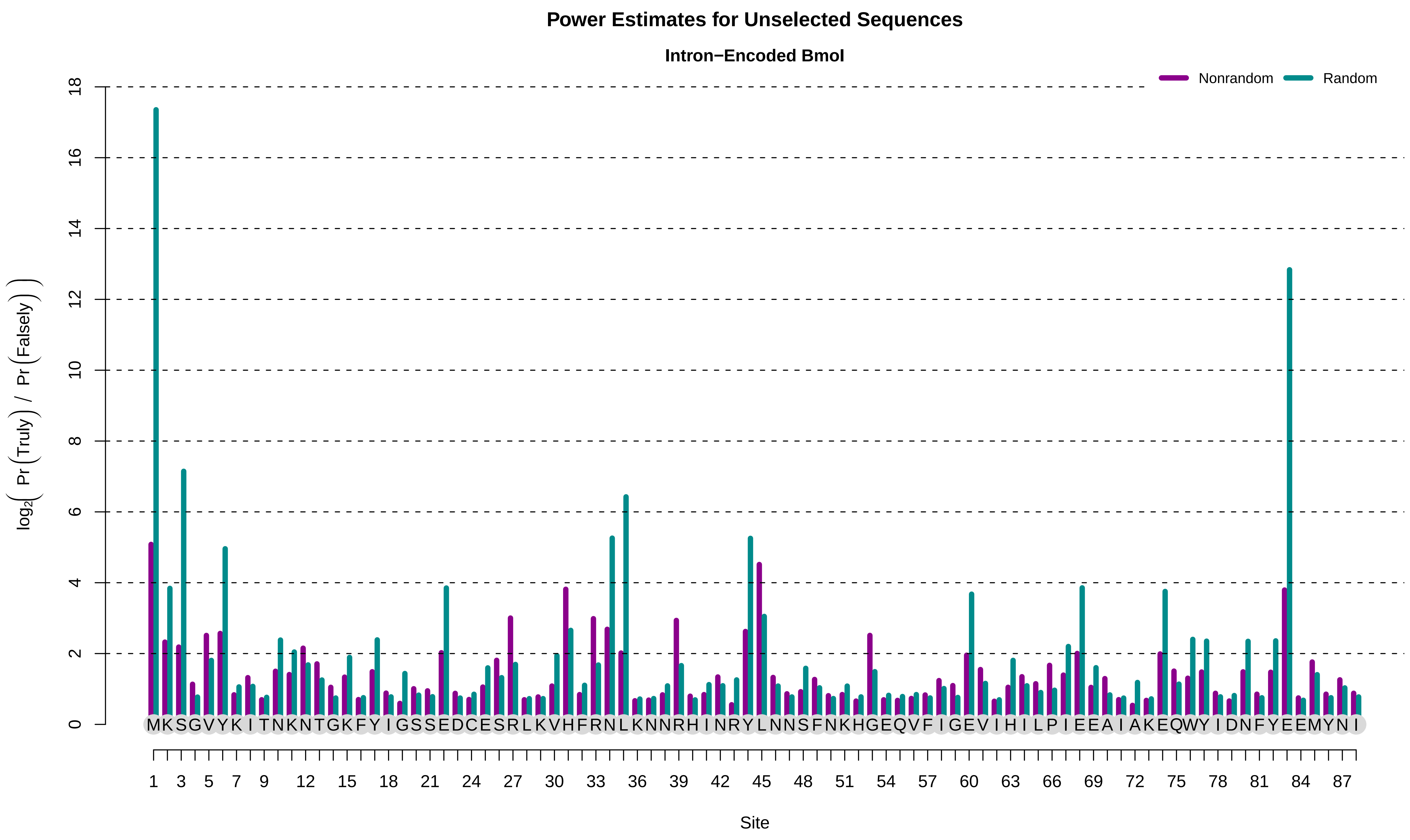

Power Estimates for Unselected Sequences
Intron−Encoded BmoI
Nonrandom
Random
$\log_2\left(\Pr\left(\text{Truly}\right) / \Pr\left(\text{Falsely}\right)\right)$
0
2
4
6
8
10
12
14
16
18
MKSGVYKITNKNTGKFYIGSSEDCESRLKVHFRNLKNNRHINRYLNNSFNKHGEQVFIGEVIHILPIEEAIAKEQWYIDNFYEEMYNI
1
3
5
7
9
12
15
18
21
24
27
30
33
36
39
42
45
48
51
54
57
60
63
66
69
72
75
78
81
84
87
Site

Estimated Count Homogeneity

Intron−Encoded BmoI

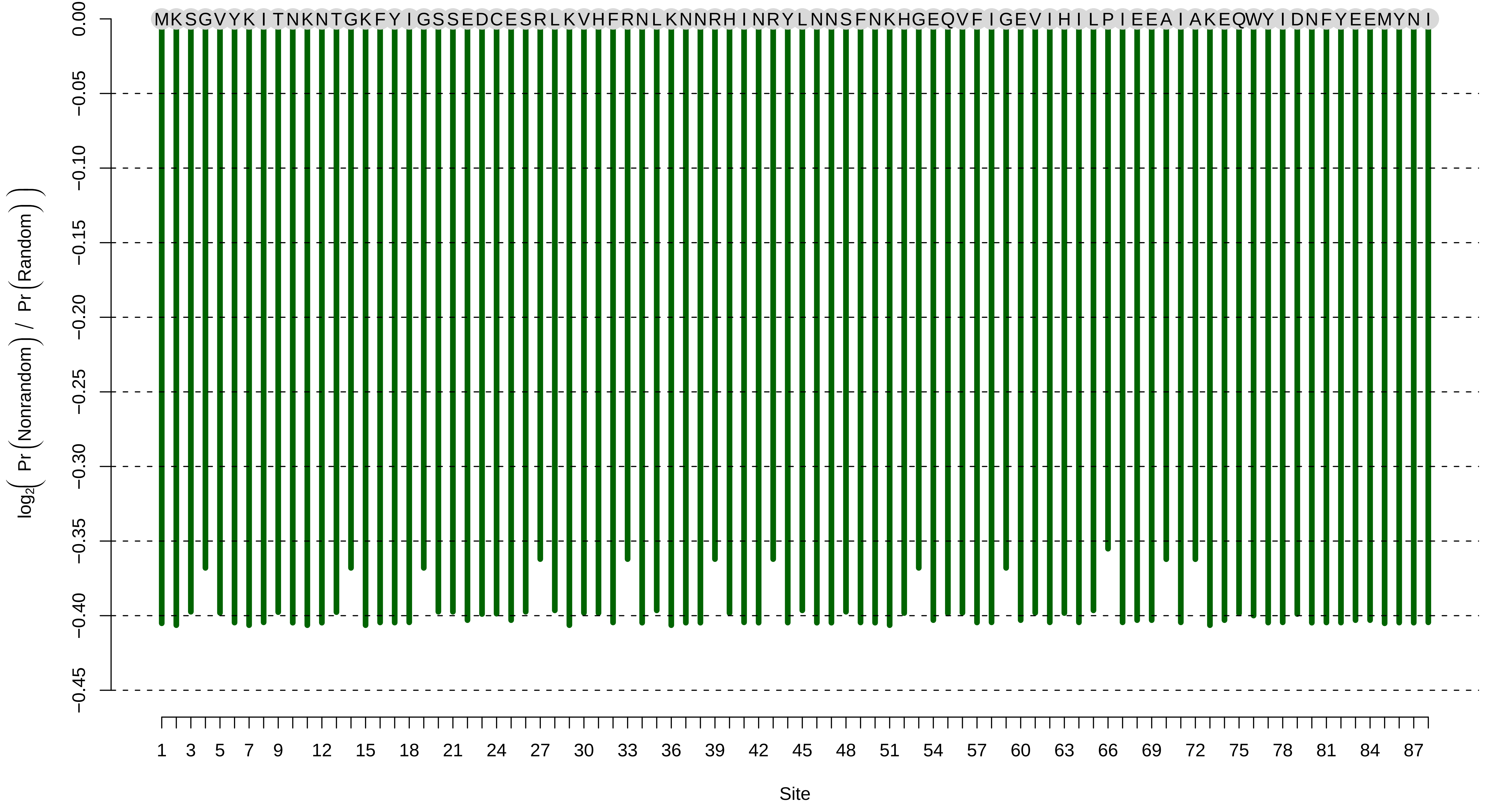

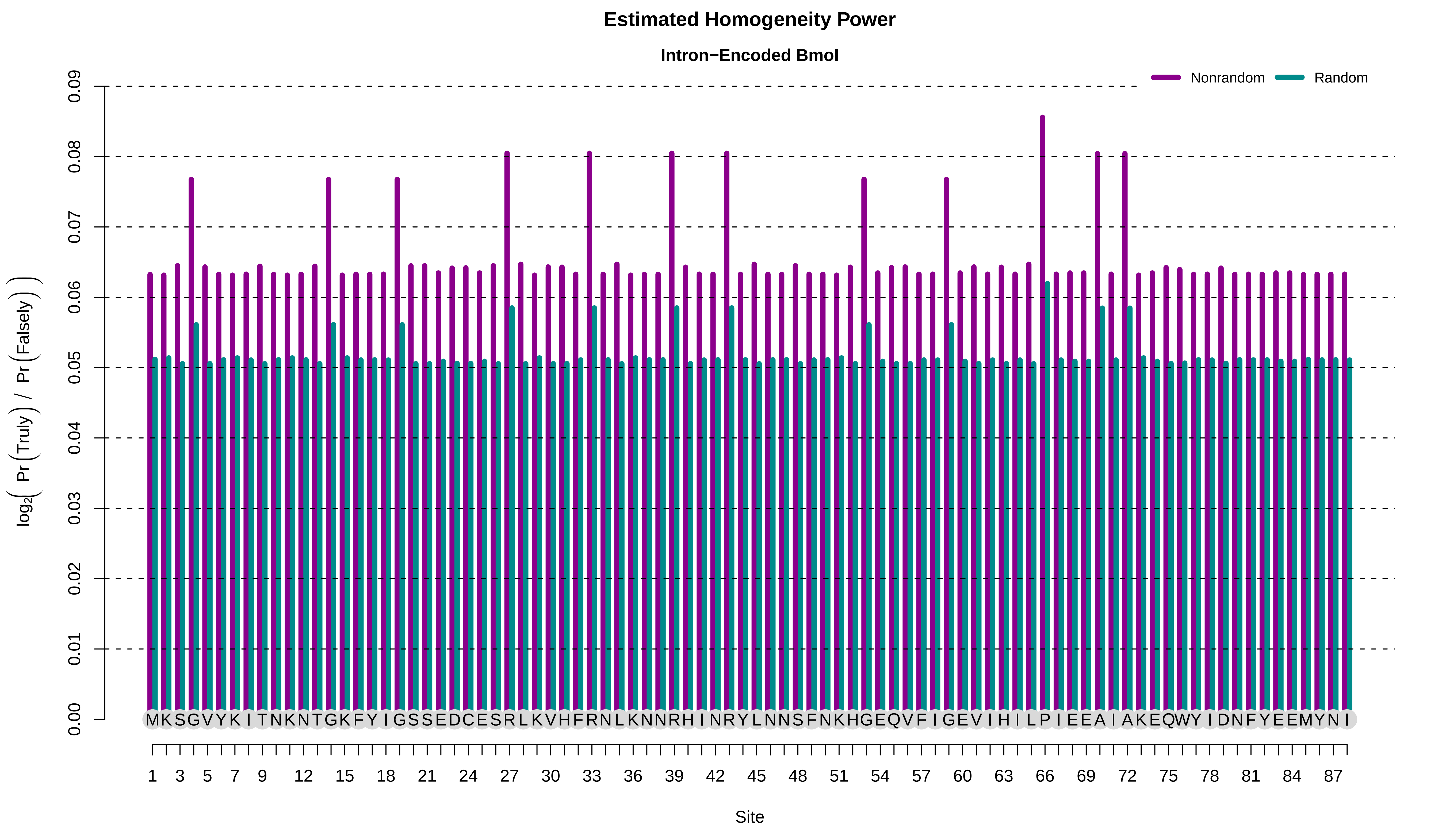

Estimated Homogeneity Power
Intron−Encoded BmoI
Nonrandom
Random
$\log_2\left(\Pr(\text{Truly}) / \Pr(\text{Falsely})\right)$
Site
M K S G V Y K I T N K N T G K F Y I G S S E D C E S R L K V H F R N L K N N R H I N R Y L N N S F N K H G E Q V F I G E V I H I L P I E E A I A K E Q W Y I D N F Y E E M Y N I
