# Supplementary material for: Estimating the evidence of selection and the reliability of inference in unigenic evolution
Source: Algorithms Mol Biol. 2010 Nov 8;5:35. doi: 10.1186/1748-7188-5-35 (PMC2994857; doi:10.1186/1748-7188-5-35)
Supplement: Additional file 7 — Sample Input and Output. Sample input, output, and driver files for the given software package. [file 1748-7188-5-35-S7.ZIP › unigenic_example/edgell.pol/by_codon_ech.pdf]

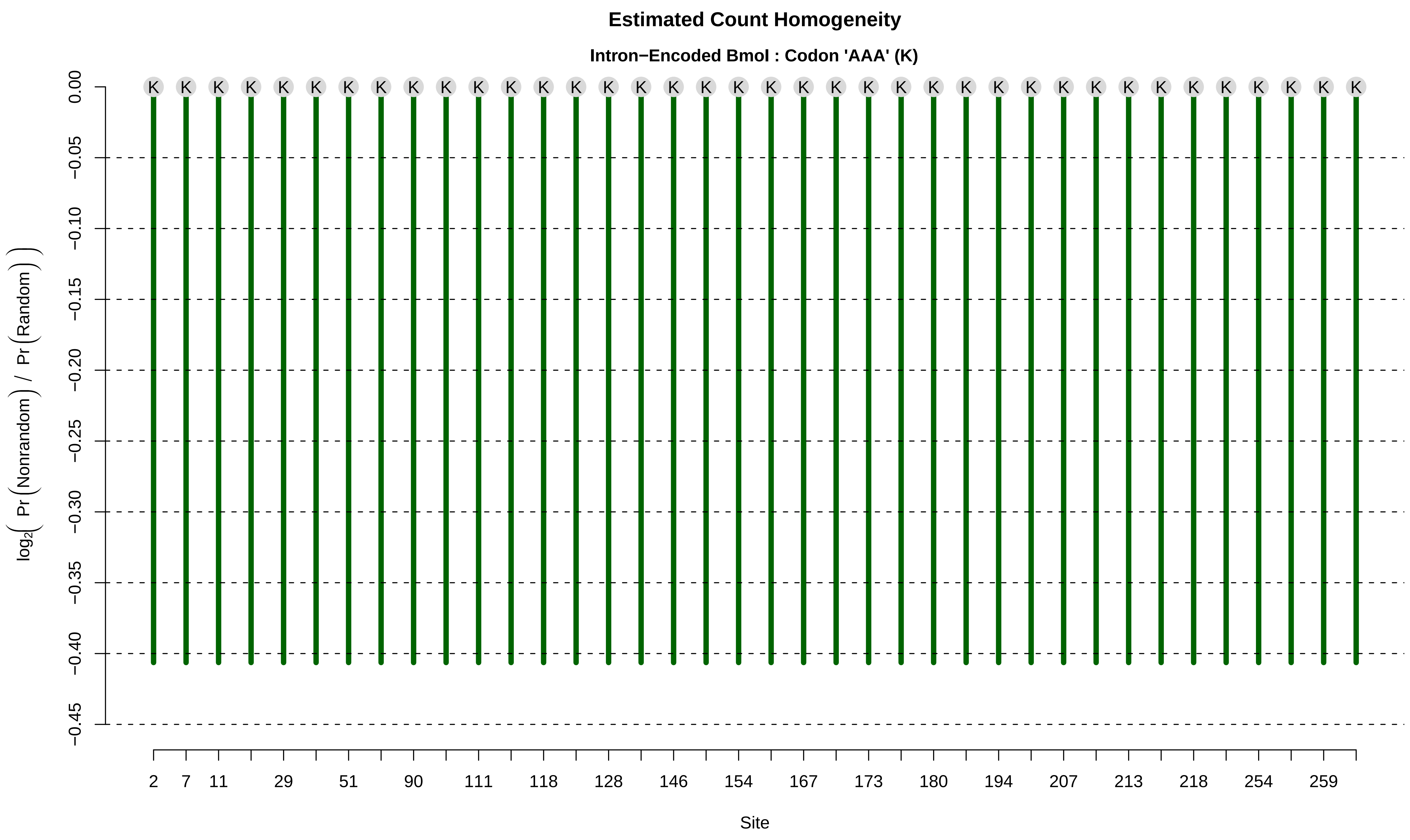
Estimated Count Homogeneity
Intron−Encoded BmoI : Codon 'AAA' (K)
$\log_2\left(\Pr\left(\text{Nonrandom}\right) / \Pr\left(\text{Random}\right) \right)$
0.00
−0.05
−0.10
−0.15
−0.20
−0.25
−0.30
−0.35
−0.40
−0.45
Site
2
7
11
29
51
90
111
118
128
146
154
167
173
180
194
207
213
218
254
259

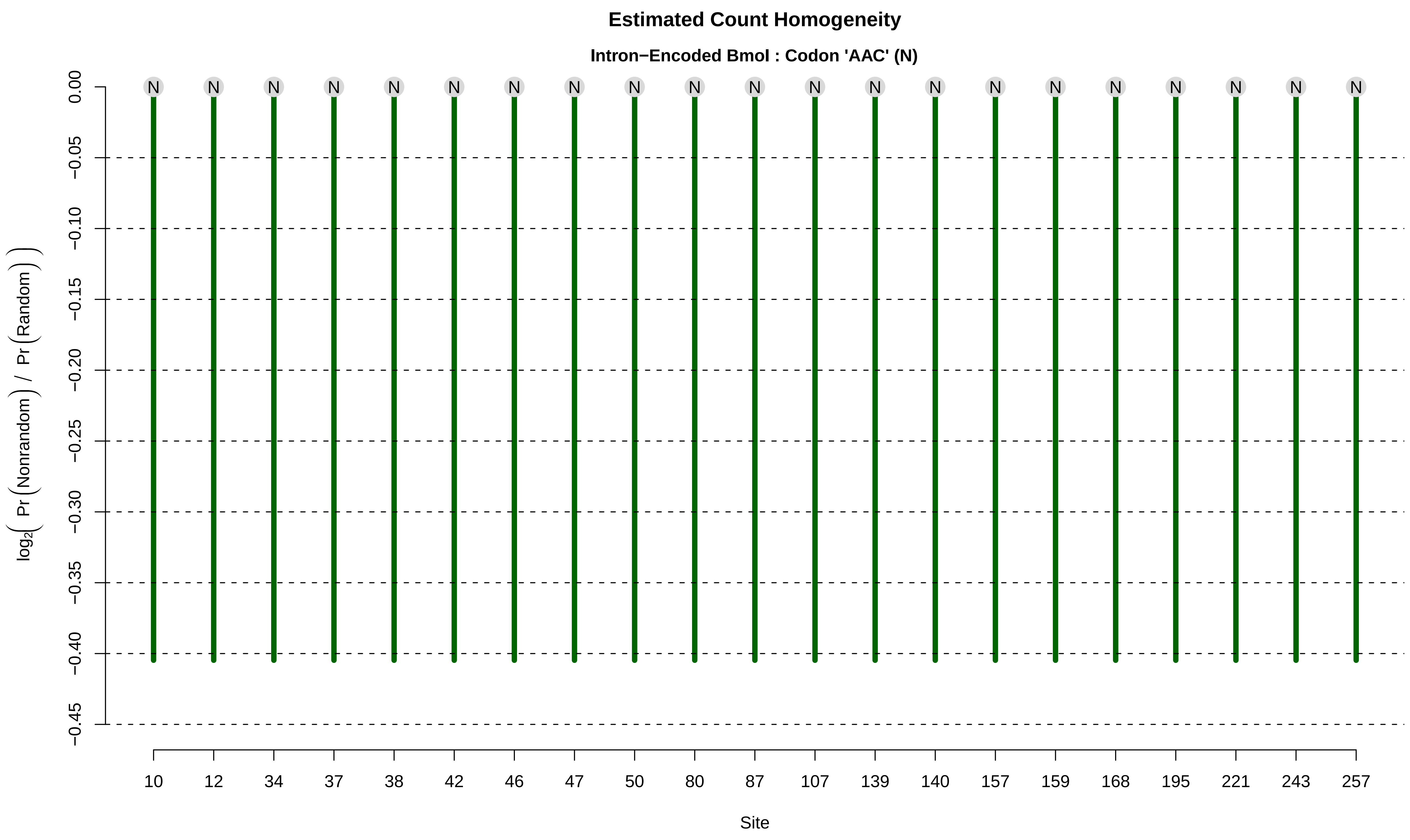

Estimated Count Homogeneity
Intron−Encoded BmoI : Codon 'AAC' (N)
log2(Pr(Nonrandom) / Pr(Random))
0.00
−0.05
−0.10
−0.15
−0.20
−0.25
−0.30
−0.35
−0.40
−0.45
N
10
12
34
37
38
42
46
47
50
80
87
107
139
140
157
159
168
195
221
243
257
Site

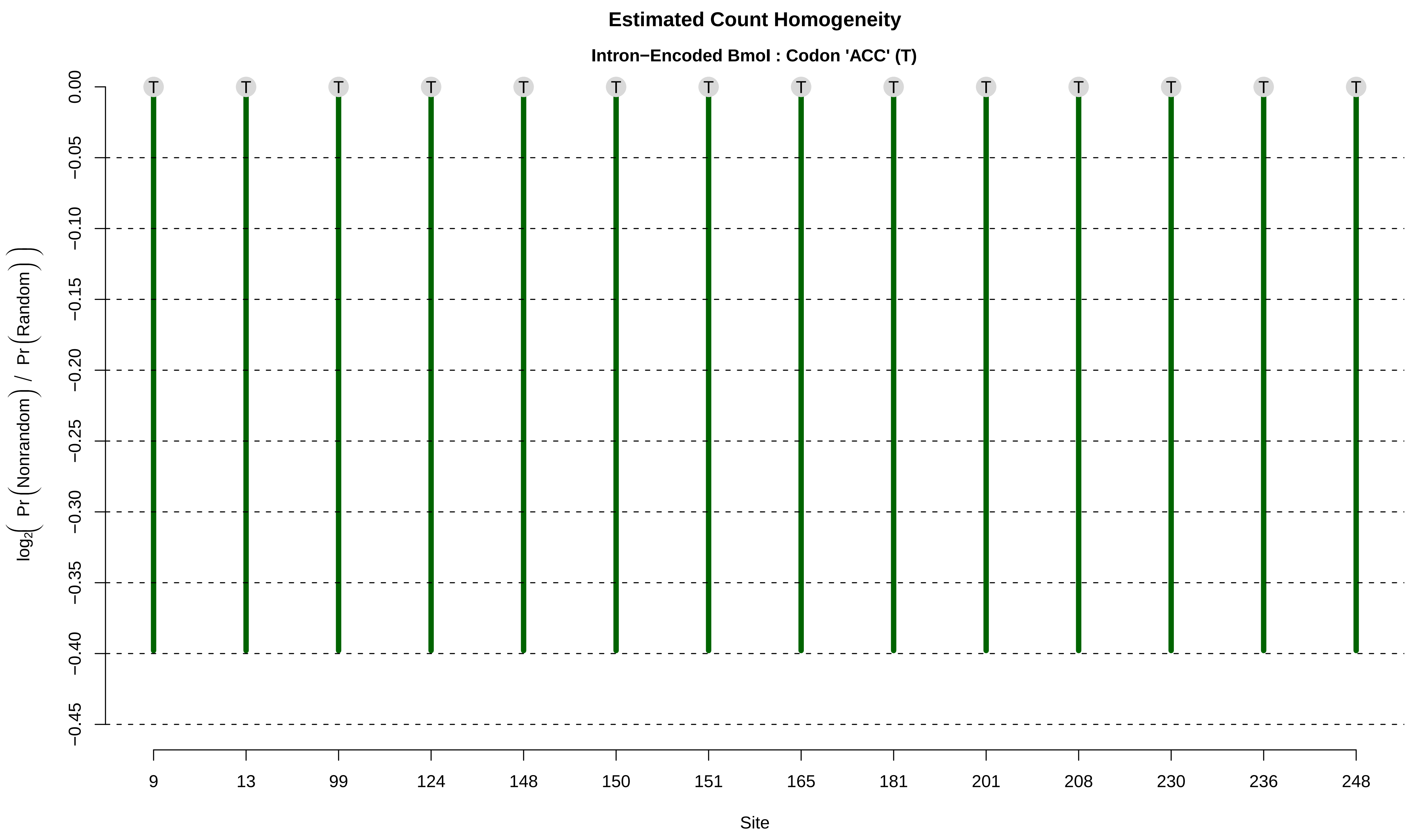

Estimated Count Homogeneity
Intron−Encoded BmoI : Codon 'ACC' (T)
log2(Pr(Nonrandom) / Pr(Random))
0.00
−0.05
−0.10
−0.15
−0.20
−0.25
−0.30
−0.35
−0.40
−0.45
T
9
13
99
124
148
150
151
165
181
201
208
230
236
248
Site

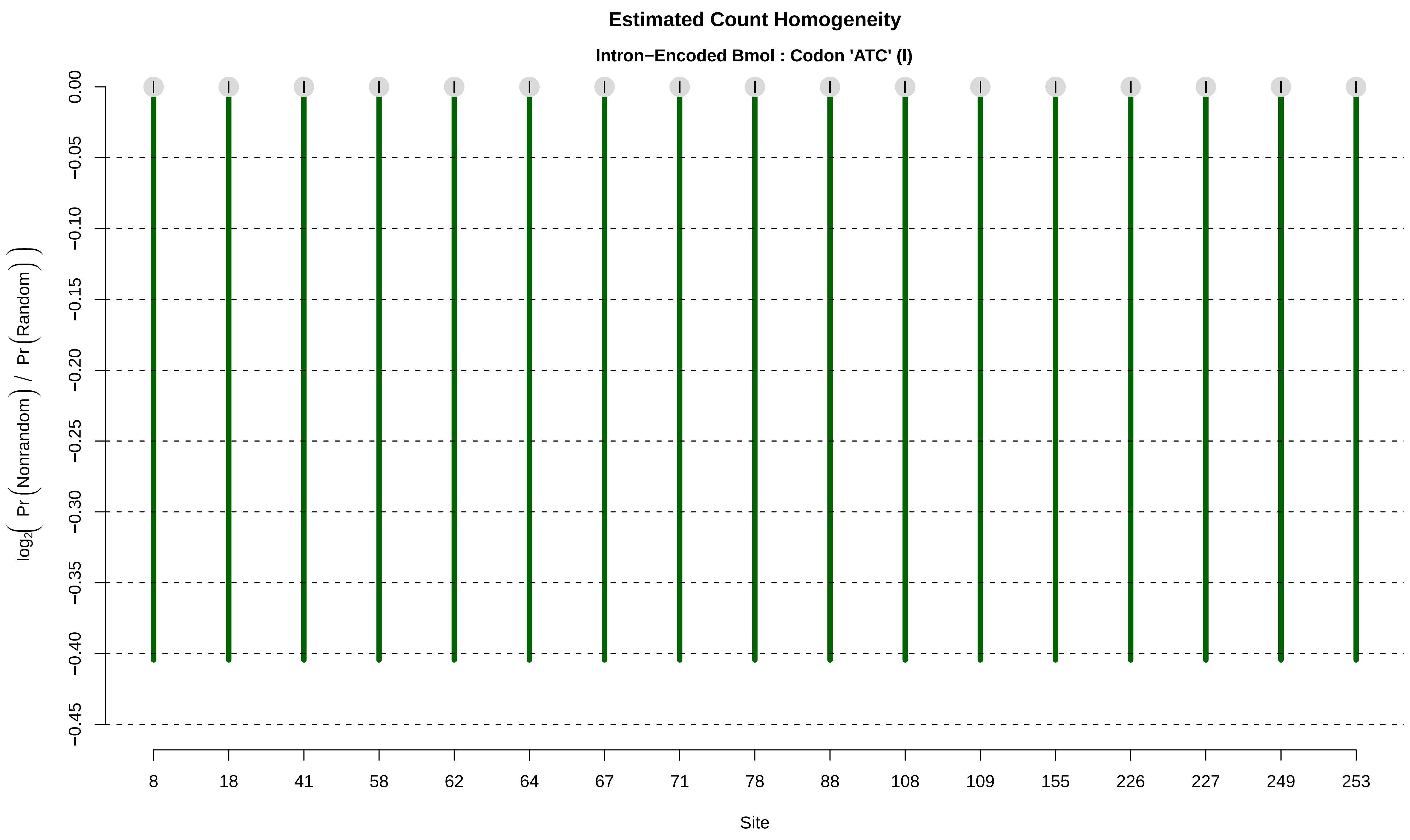

Estimated Count Homogeneity
Intron−Encoded BmoI : Codon 'ATC' (I)
log2(Pr(Nonrandom) / Pr(Random))
0.00
−0.05
−0.10
−0.15
−0.20
−0.25
−0.30
−0.35
−0.40
−0.45
8
18
41
58
62
64
67
71
78
88
108
109
155
226
227
249
253
Site

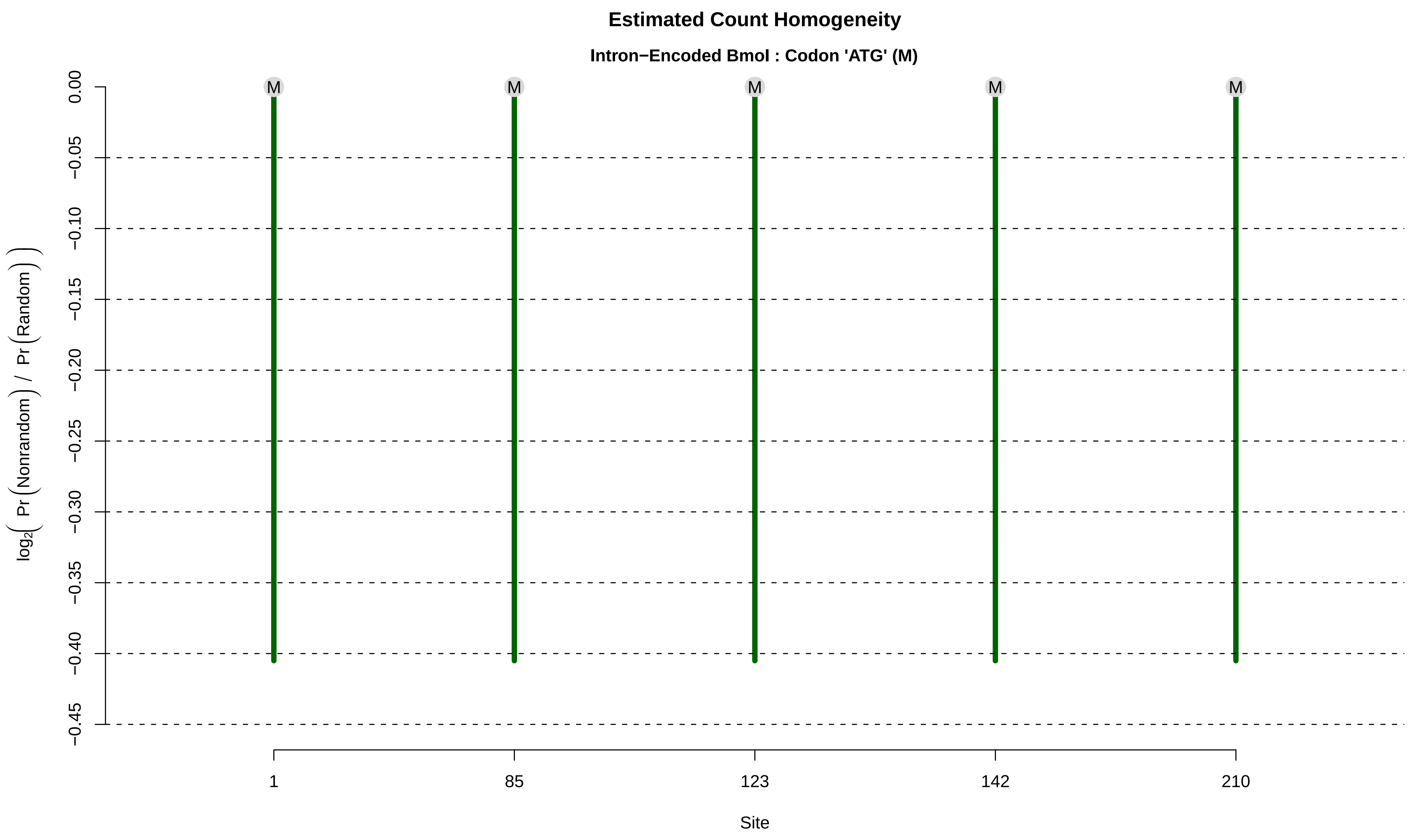
Estimated Count Homogeneity
Intron−Encoded BmoI : Codon 'ATG' (M)
log2(Pr(Nonrandom) / Pr(Random))
0.00
−0.05
−0.10
−0.15
−0.20
−0.25
−0.30
−0.35
−0.40
−0.45
M
M
M
M
M
1
85
123
142
210
Site

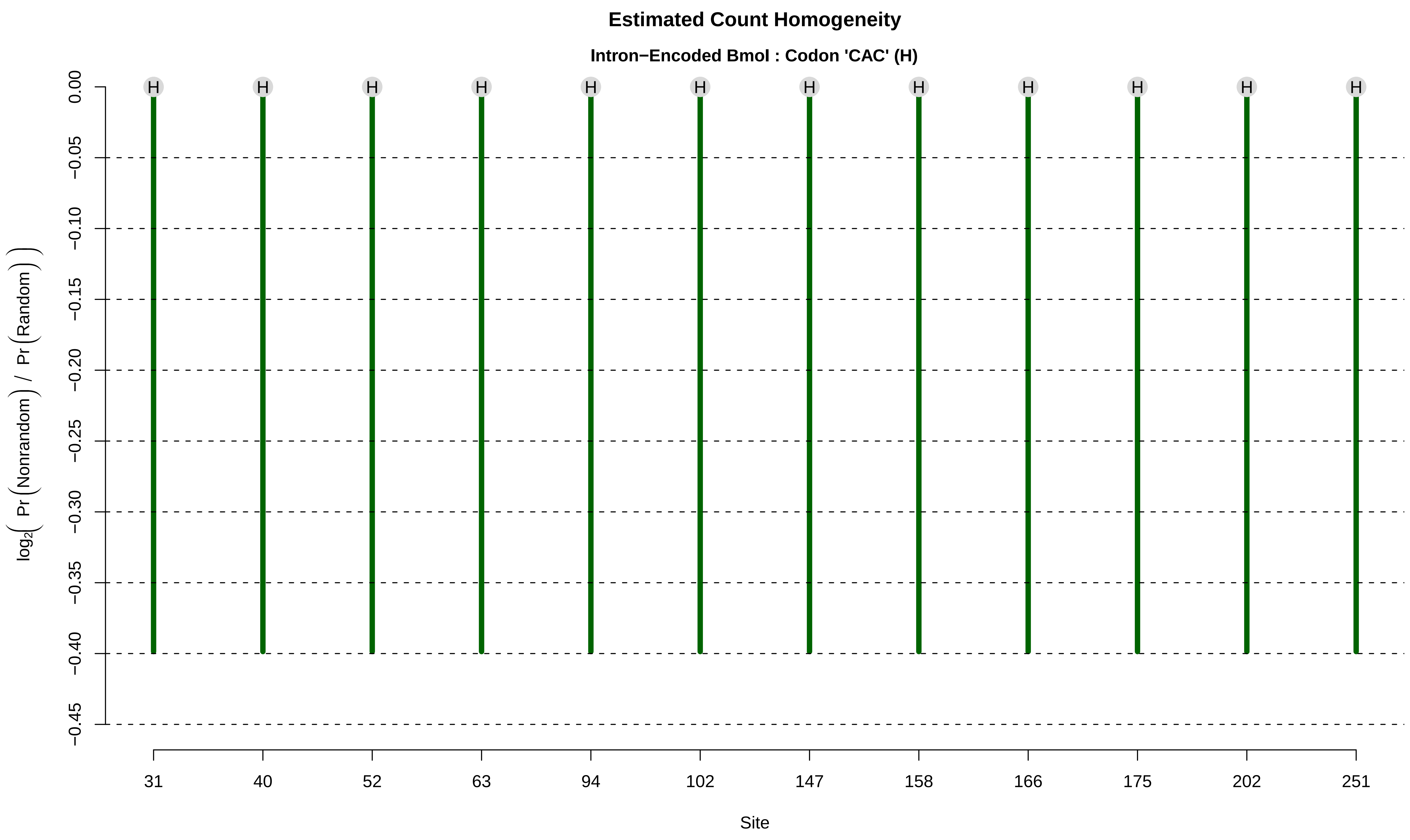
Estimated Count Homogeneity
Intron−Encoded BmoI : Codon 'CAC' (H)
log2(Pr(Nonrandom) / Pr(Random))
0.00
−0.05
−0.10
−0.15
−0.20
−0.25
−0.30
−0.35
−0.40
−0.45
H
31
40
52
63
94
102
147
158
166
175
202
251
Site

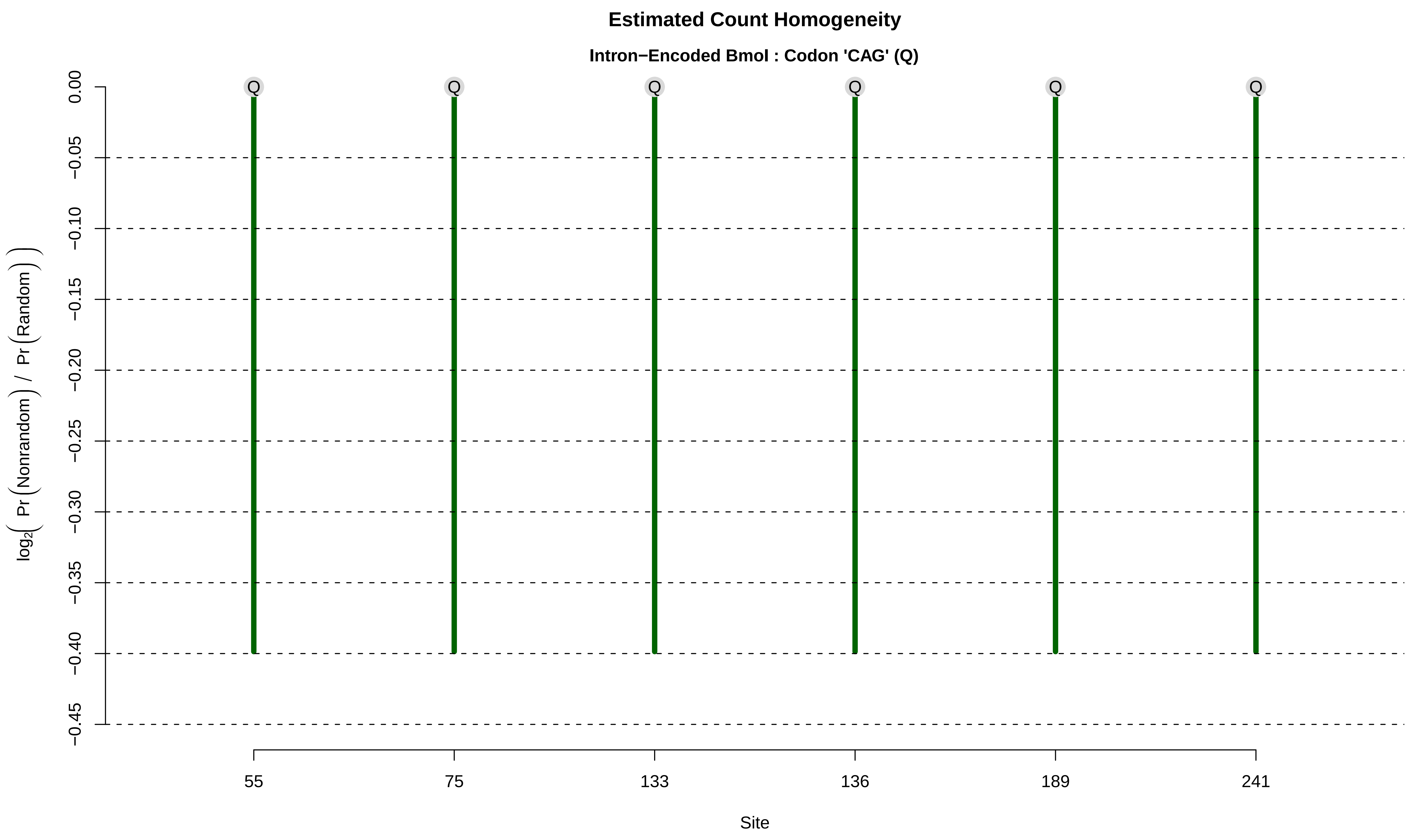

Estimated Count Homogeneity
Intron−Encoded BmoI : Codon 'CAG' (Q)
0.00
−0.05
−0.10
−0.15
−0.20
−0.25
−0.30
−0.35
−0.40
−0.45
log2(Pr(Nonrandom) / Pr(Random))
Q
Q
Q
Q
Q
Q
55
75
133
136
189
241
Site

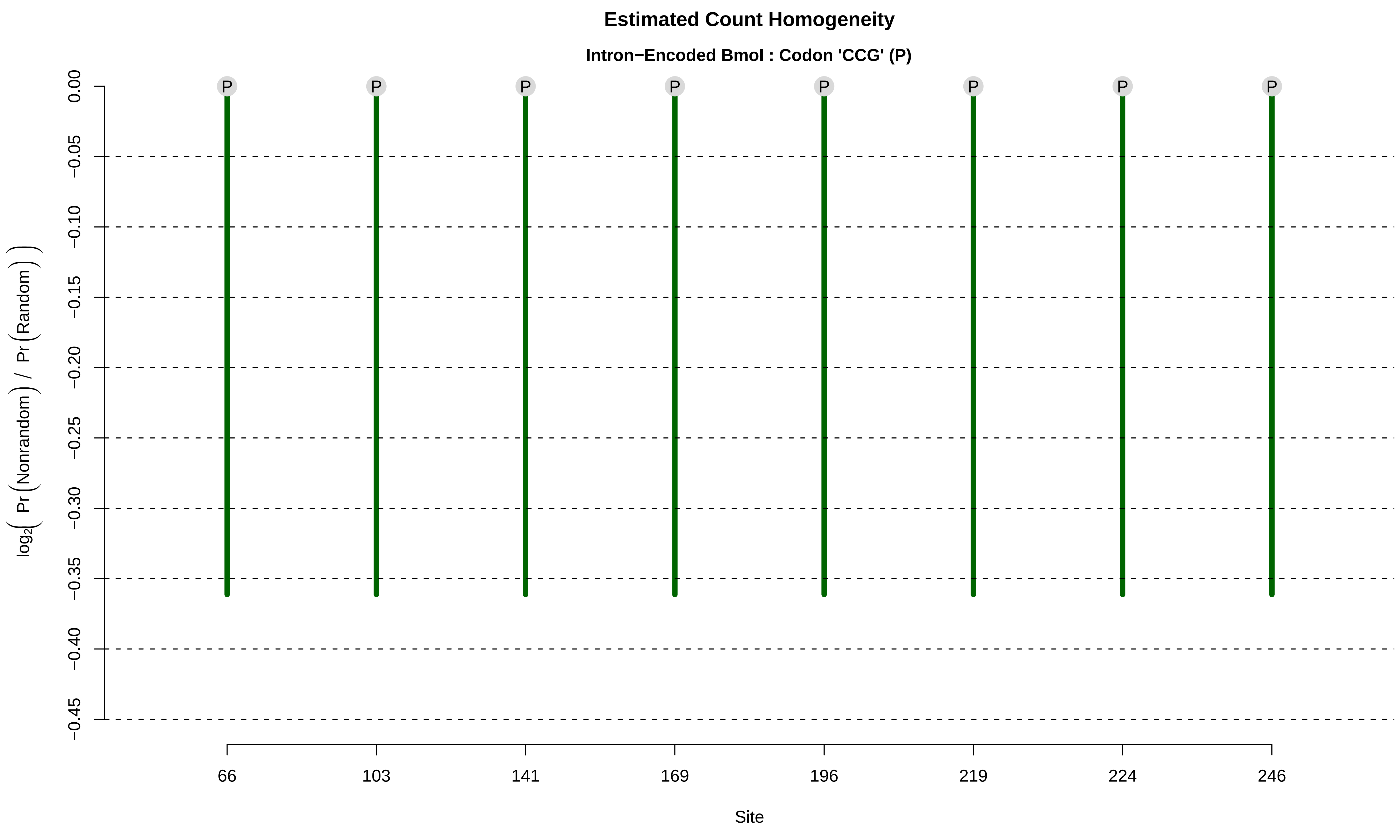
Estimated Count Homogeneity
Intron−Encoded BmoI : Codon 'CCG' (P)
log2(Pr(Nonrandom) / Pr(Random))
0.00
−0.05
−0.10
−0.15
−0.20
−0.25
−0.30
−0.35
−0.40
−0.45
P
P
P
P
P
P
P
P
66
103
141
169
196
219
224
246
Site

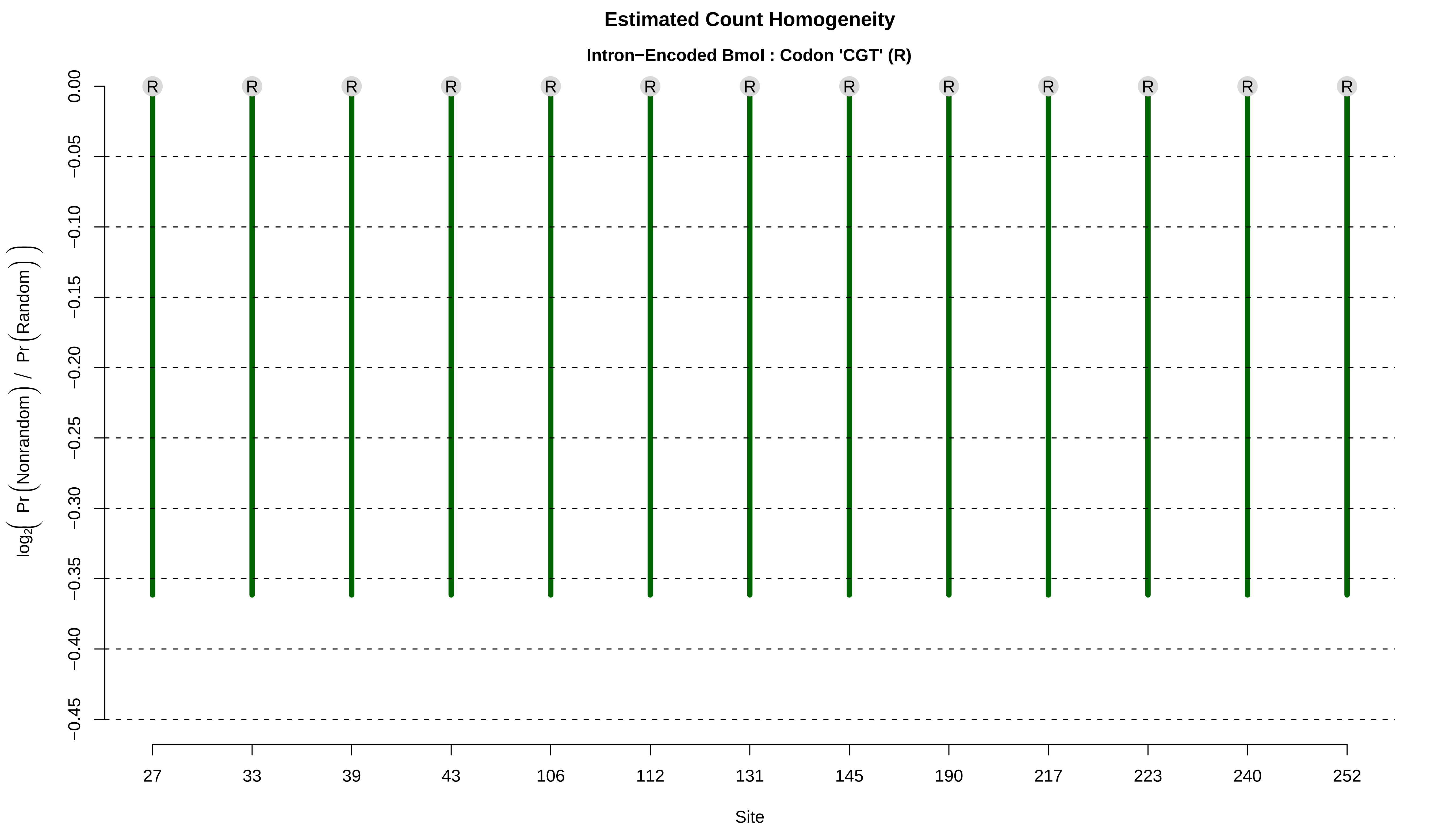

Estimated Count Homogeneity
Intron−Encoded BmoI : Codon 'CGT' (R)
log2(Pr(Nonrandom) / Pr(Random))
0.00
−0.05
−0.10
−0.15
−0.20
−0.25
−0.30
−0.35
−0.40
−0.45
R
27
33
39
43
106
112
131
145
190
217
223
240
252
Site

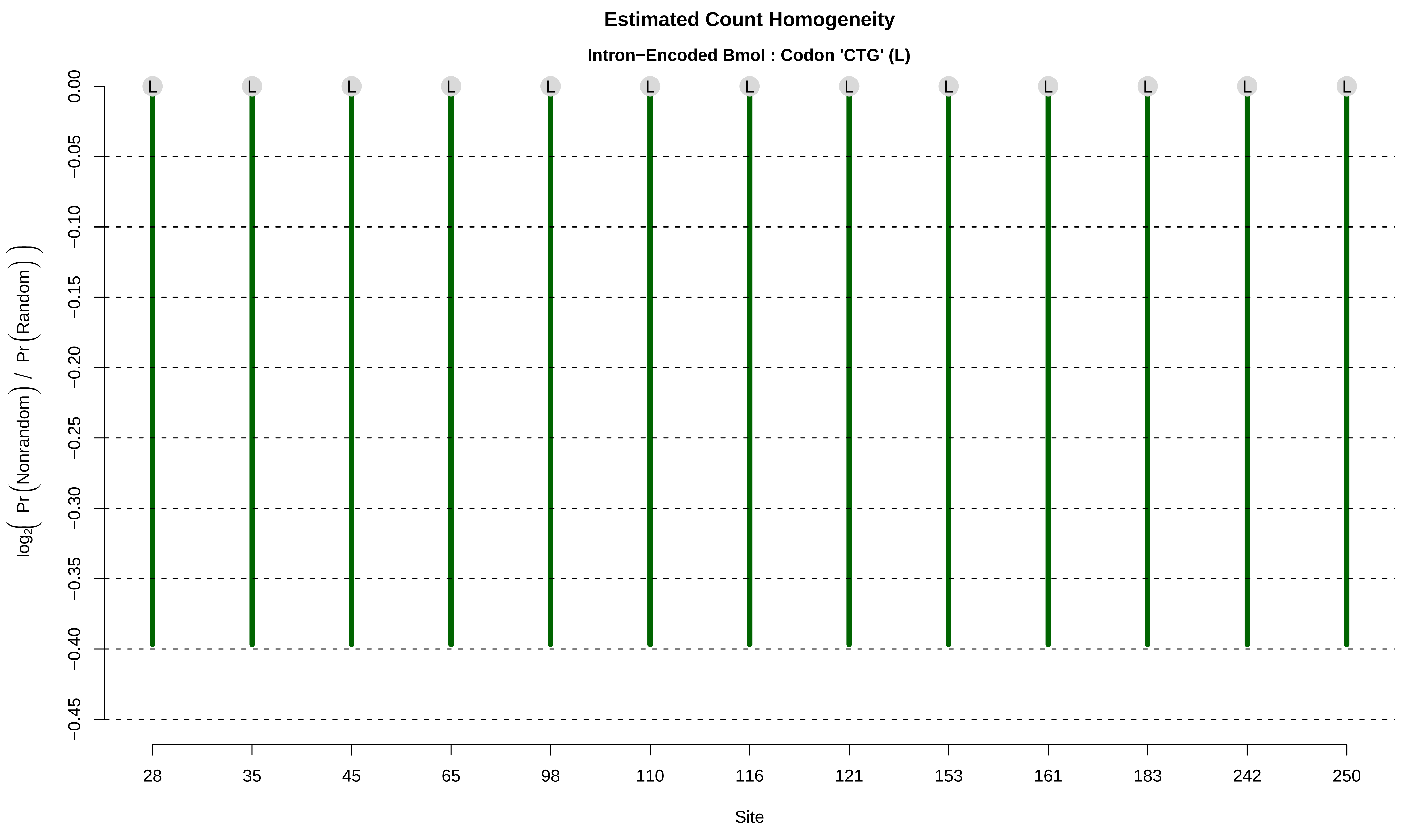

Estimated Count Homogeneity
Intron−Encoded BmoI : Codon 'CTG' (L)
log2(Pr(Nonrandom) / Pr(Random))
0.00
−0.05
−0.10
−0.15
−0.20
−0.25
−0.30
−0.35
−0.40
−0.45
L
28
35
45
65
98
110
116
121
153
161
183
242
250
Site

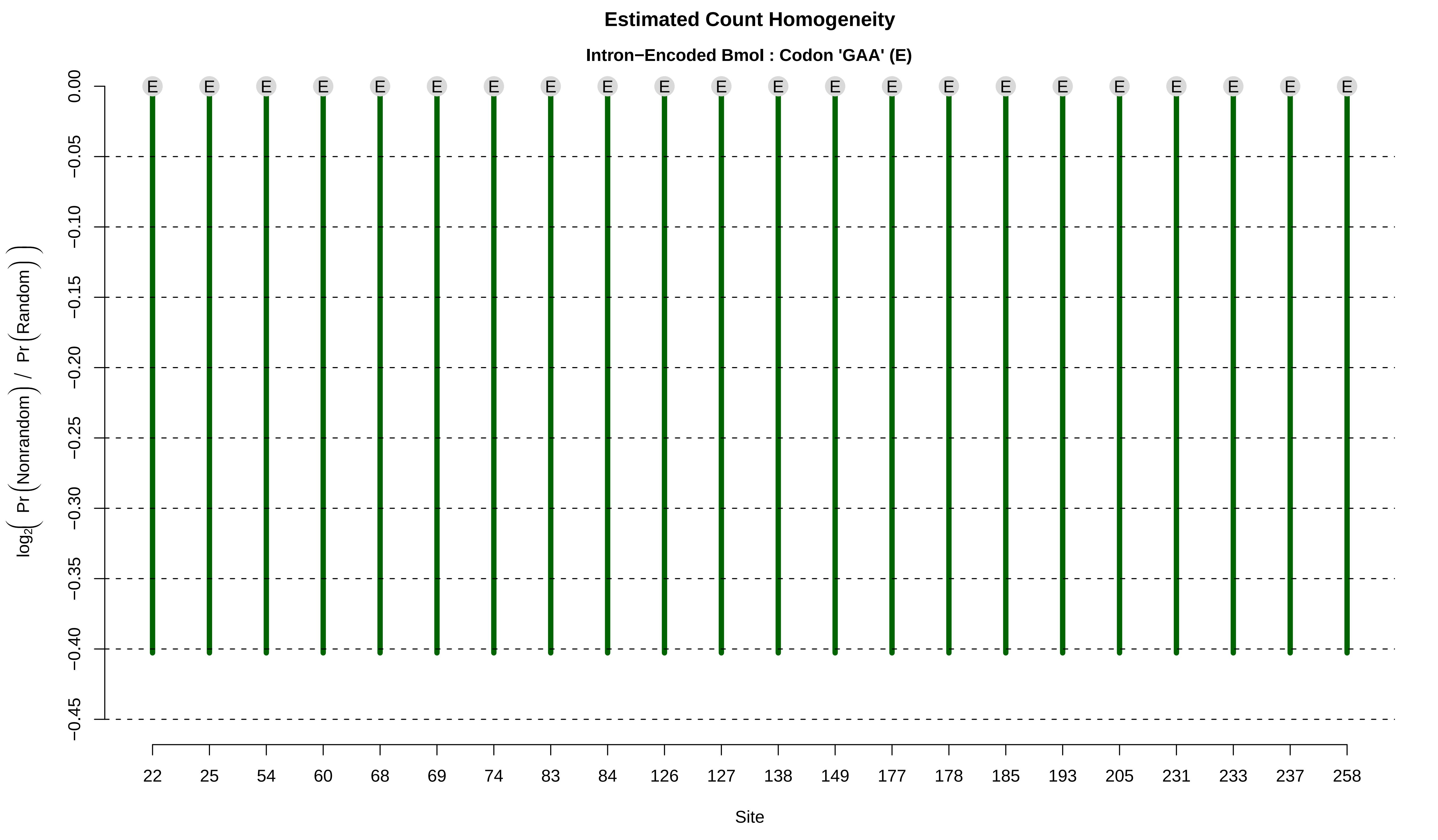
Estimated Count Homogeneity
Intron−Encoded BmoI : Codon 'GAA' (E)
log2(Pr(Nonrandom) / Pr(Random))
0.00
−0.05
−0.10
−0.15
−0.20
−0.25
−0.30
−0.35
−0.40
−0.45
E
22
25
54
60
68
69
74
83
84
126
127
138
149
177
178
185
193
205
231
233
237
258
Site

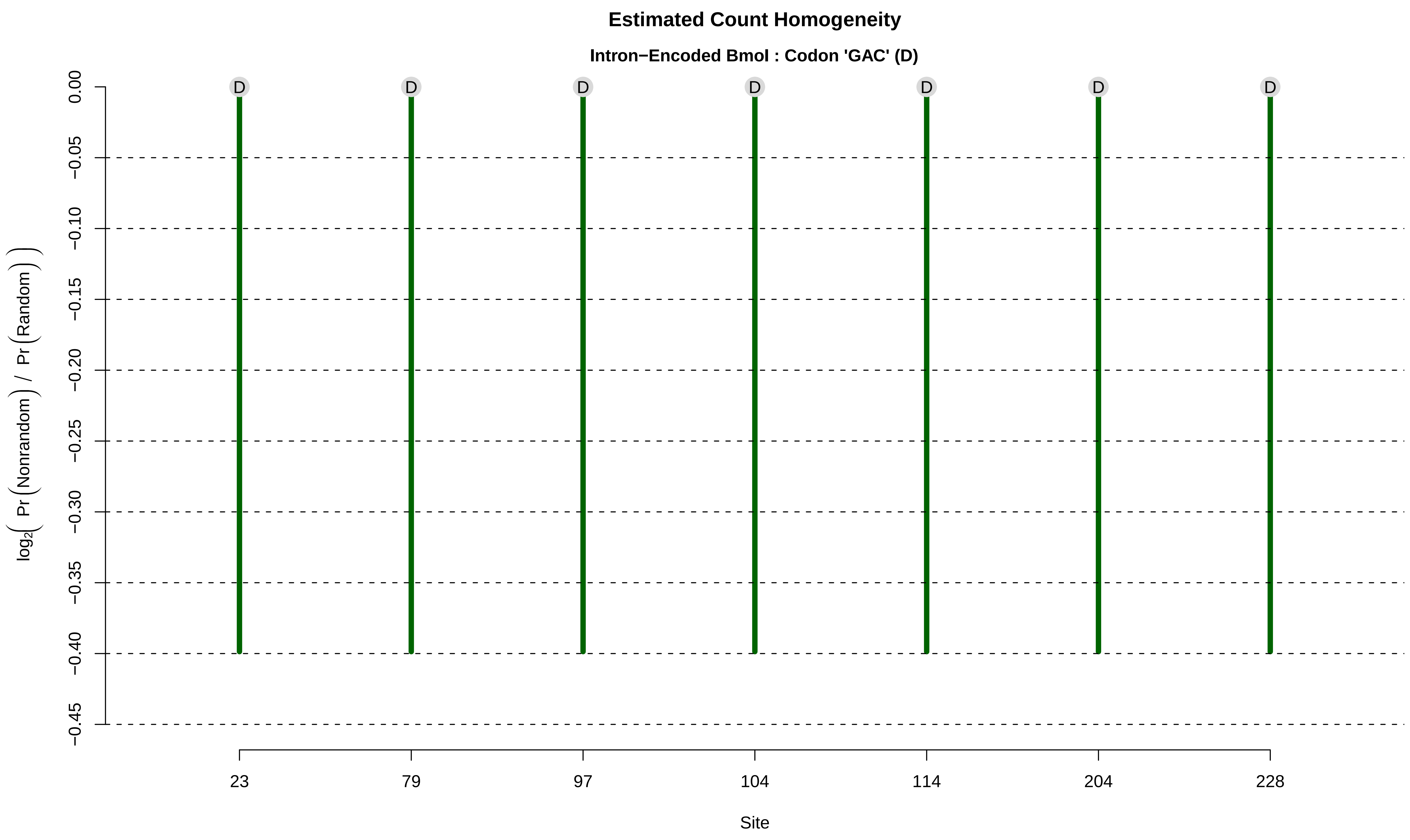

Estimated Count Homogeneity
Intron−Encoded BmoI : Codon 'GAC' (D)
0.00
−0.05
−0.10
−0.15
−0.20
−0.25
−0.30
−0.35
−0.40
−0.45
log2(Pr(Nonrandom) / Pr(Random))
D
23
79
97
104
114
204
228
Site

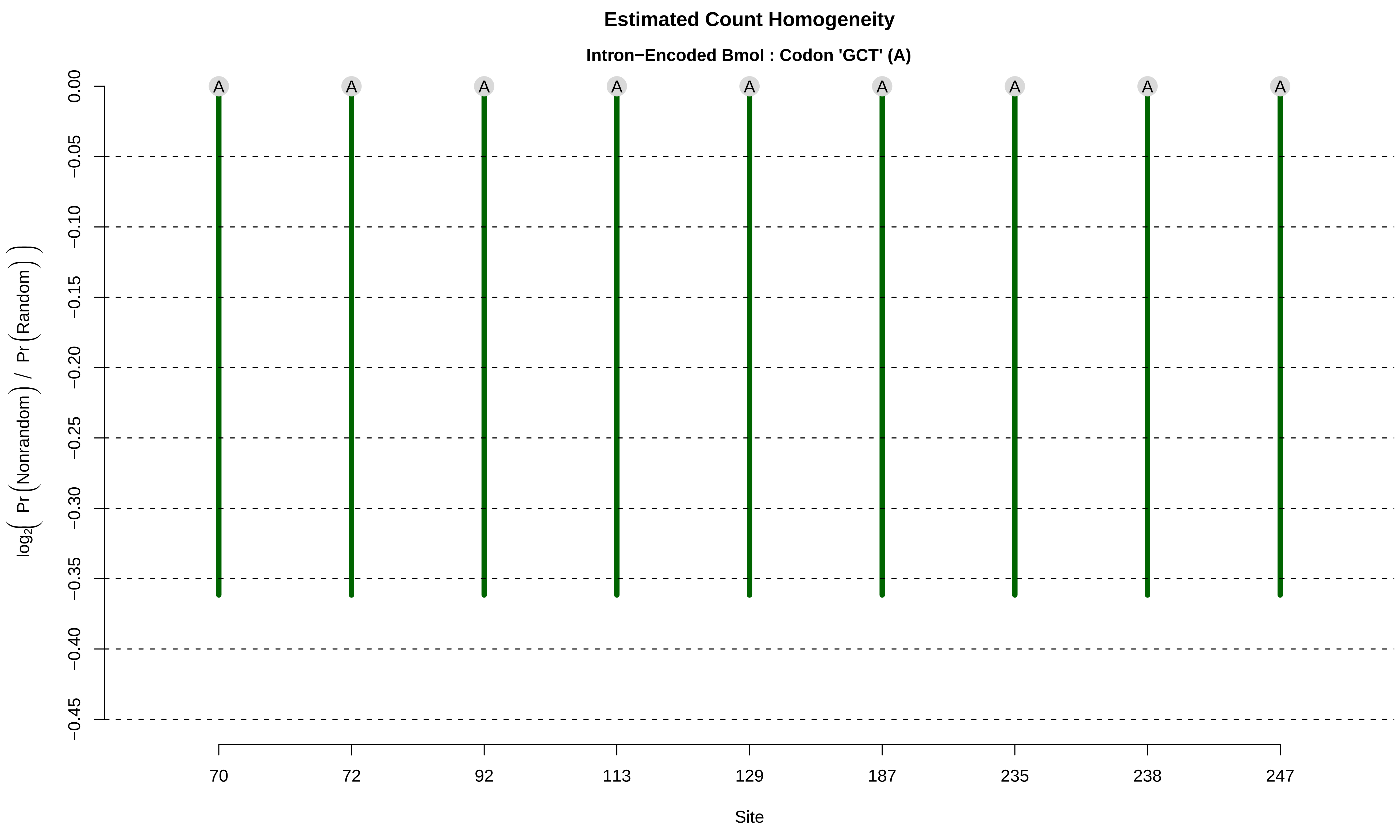

Estimated Count Homogeneity
Intron−Encoded BmoI : Codon 'GCT' (A)
log2(Pr(Nonrandom) / Pr(Random))
0.00
−0.05
−0.10
−0.15
−0.20
−0.25
−0.30
−0.35
−0.40
−0.45
A
A
A
A
A
A
A
A
A
70
72
92
113
129
187
235
238
247
Site

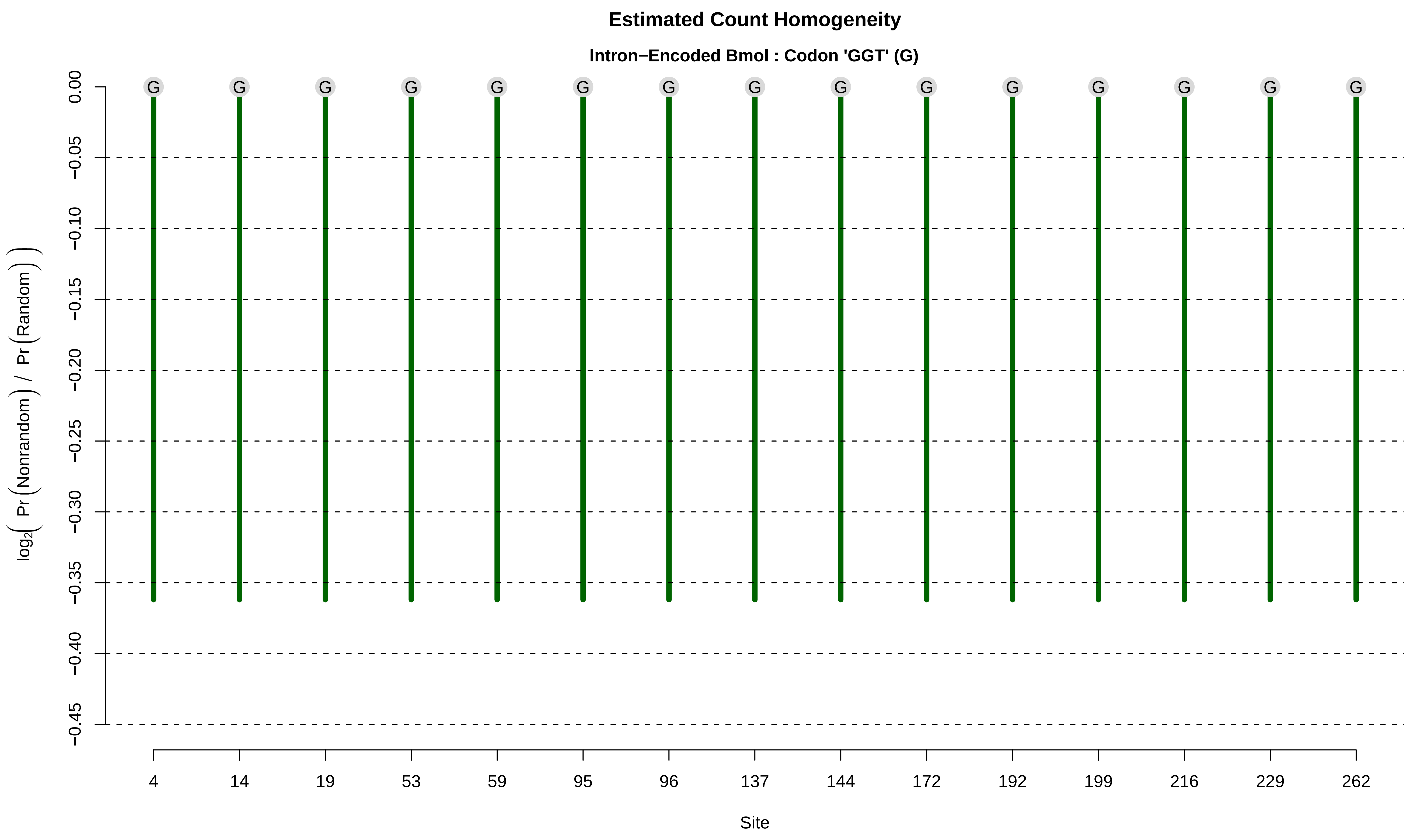
Estimated Count Homogeneity
Intron−Encoded BmoI : Codon 'GGT' (G)
log2(Pr(Nonrandom) / Pr(Random))
0.00
−0.05
−0.10
−0.15
−0.20
−0.25
−0.30
−0.35
−0.40
−0.45
G
4
14
19
53
59
95
96
137
144
172
192
199
216
229
262
Site

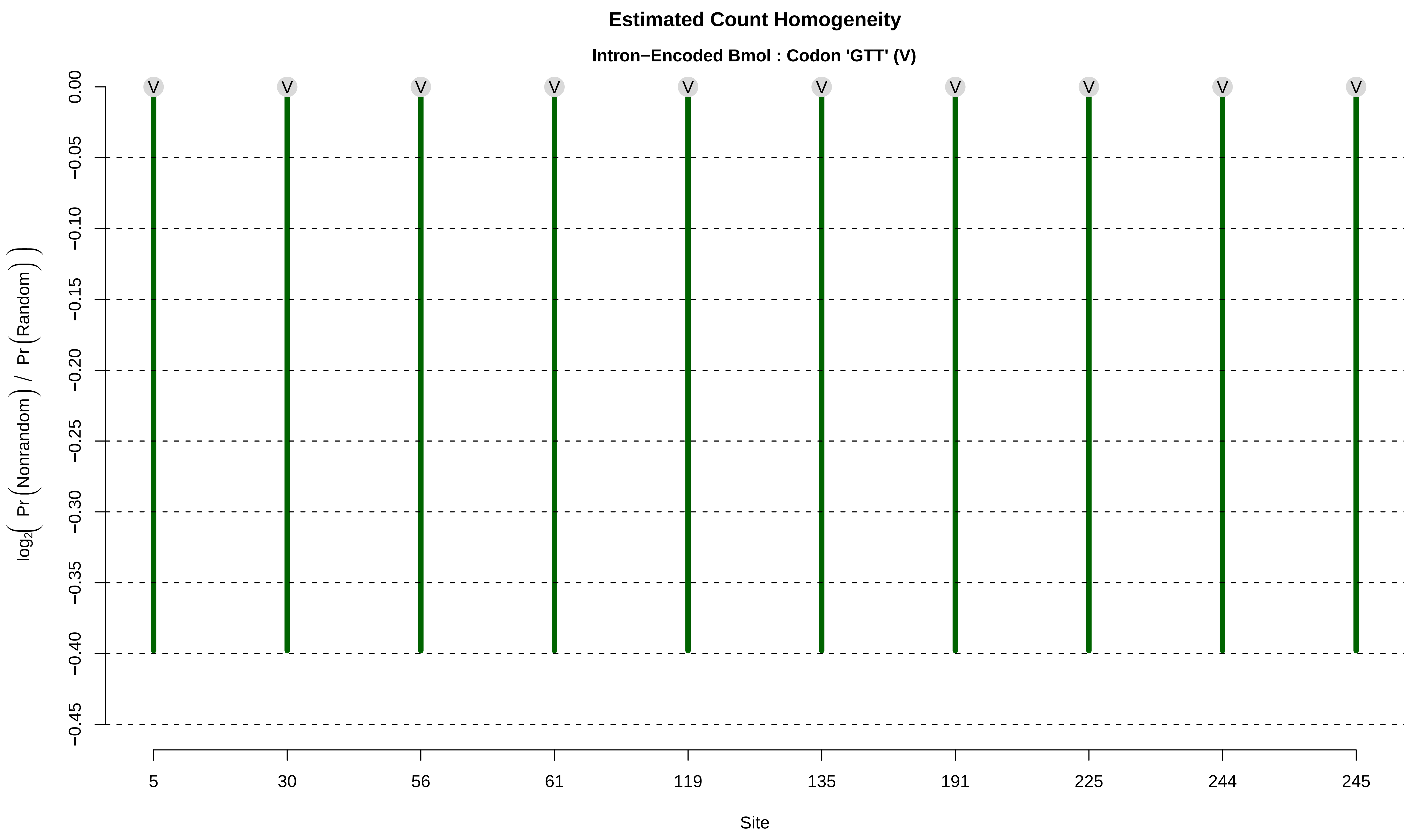

Estimated Count Homogeneity
Intron−Encoded BmoI : Codon 'GTT' (V)
log2(Pr(Nonrandom) / Pr(Random))
0.00
−0.05
−0.10
−0.15
−0.20
−0.25
−0.30
−0.35
−0.40
−0.45
V
5
30
56
61
119
135
191
225
244
245
Site

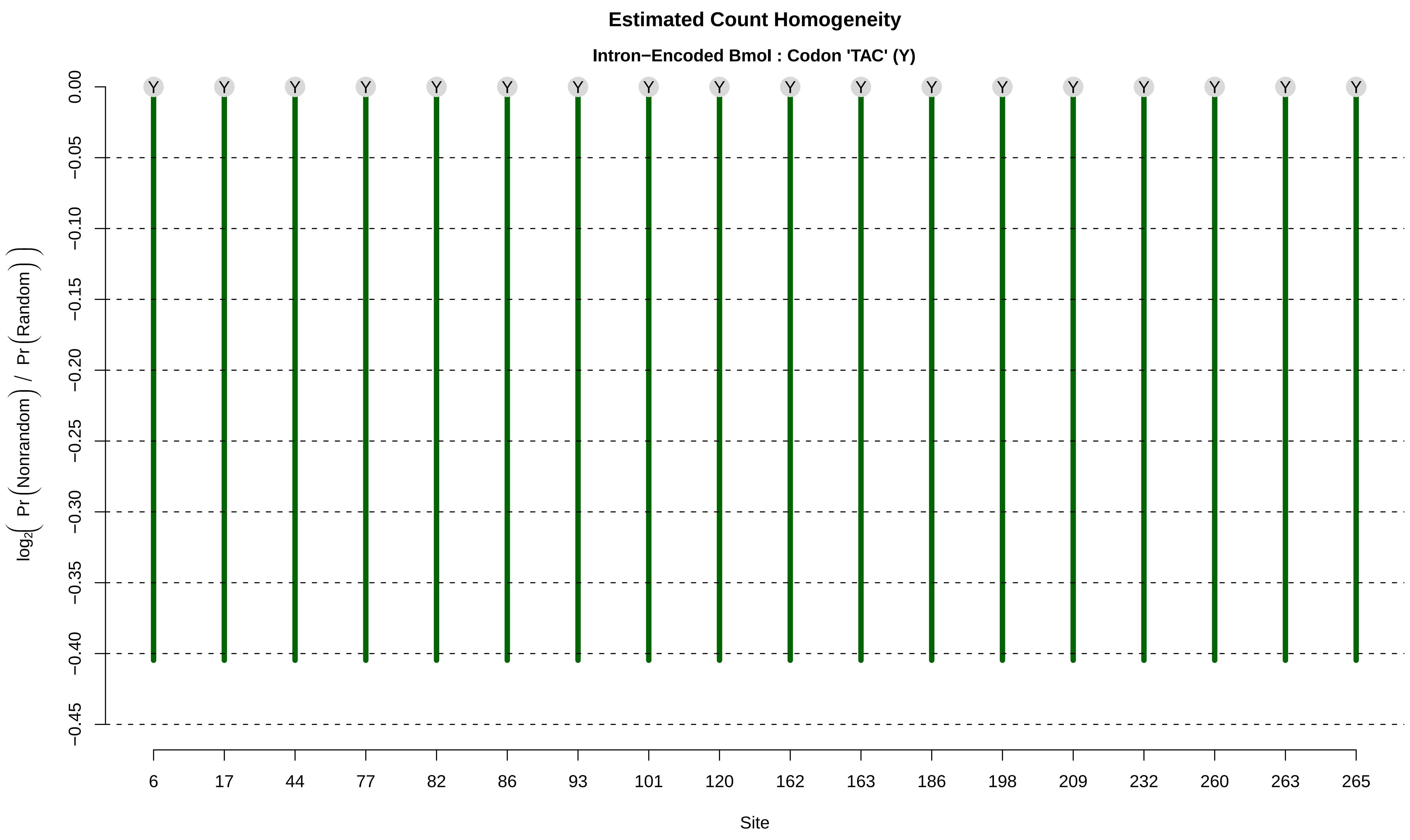
Estimated Count Homogeneity
Intron−Encoded BmoI : Codon 'TAC' (Y)
log2(Pr(Nonrandom) / Pr(Random))
0.00
−0.05
−0.10
−0.15
−0.20
−0.25
−0.30
−0.35
−0.40
−0.45
Y
6
17
44
77
82
86
93
101
120
162
163
186
198
209
232
260
263
265
Site

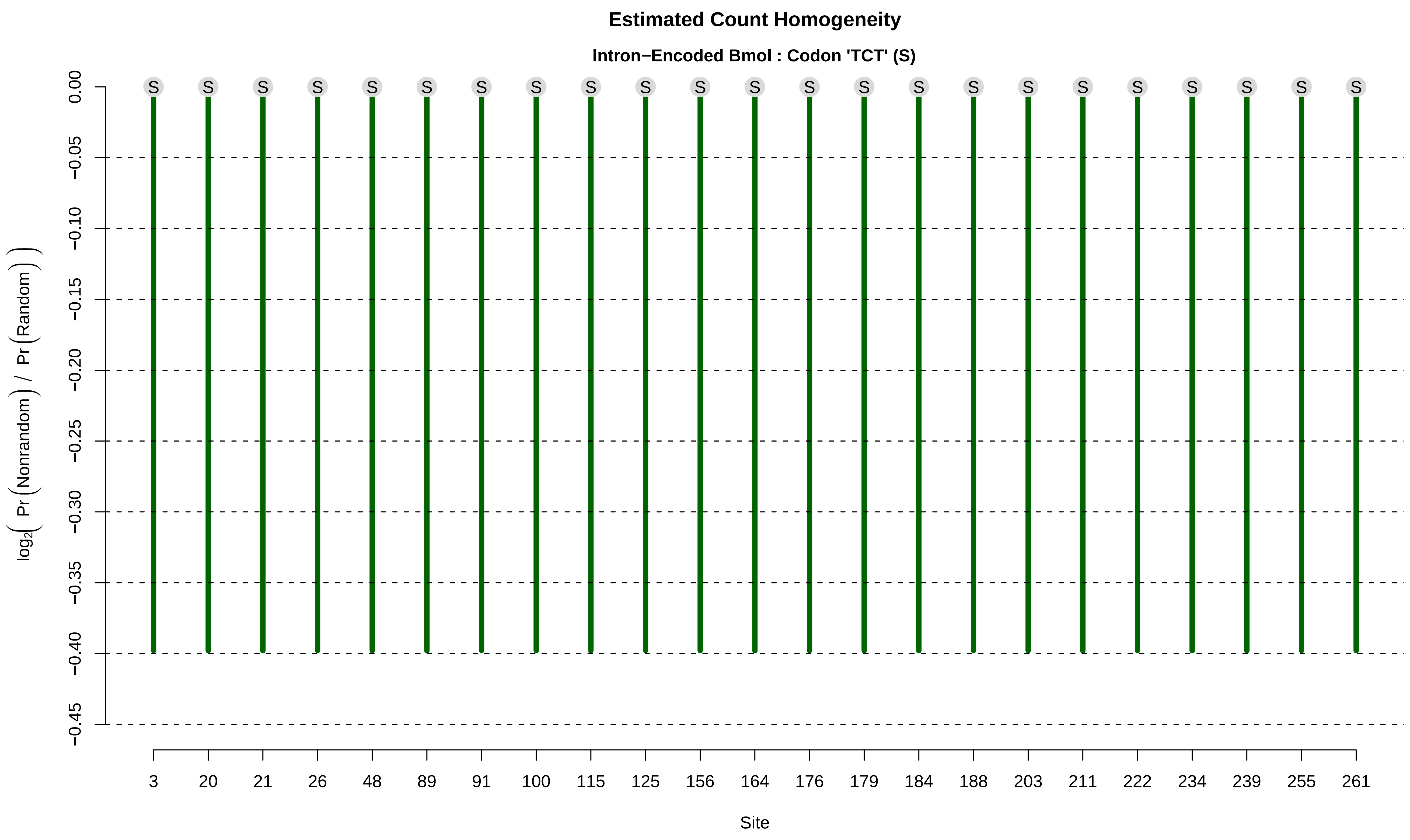

Estimated Count Homogeneity
Intron−Encoded BmoI : Codon 'TCT' (S)
log2(Pr(Nonrandom) / Pr(Random))
0.00
−0.05
−0.10
−0.15
−0.20
−0.25
−0.30
−0.35
−0.40
−0.45
S
3
20
21
26
48
89
91
100
115
125
156
164
176
179
184
188
203
211
222
234
239
255
261
Site

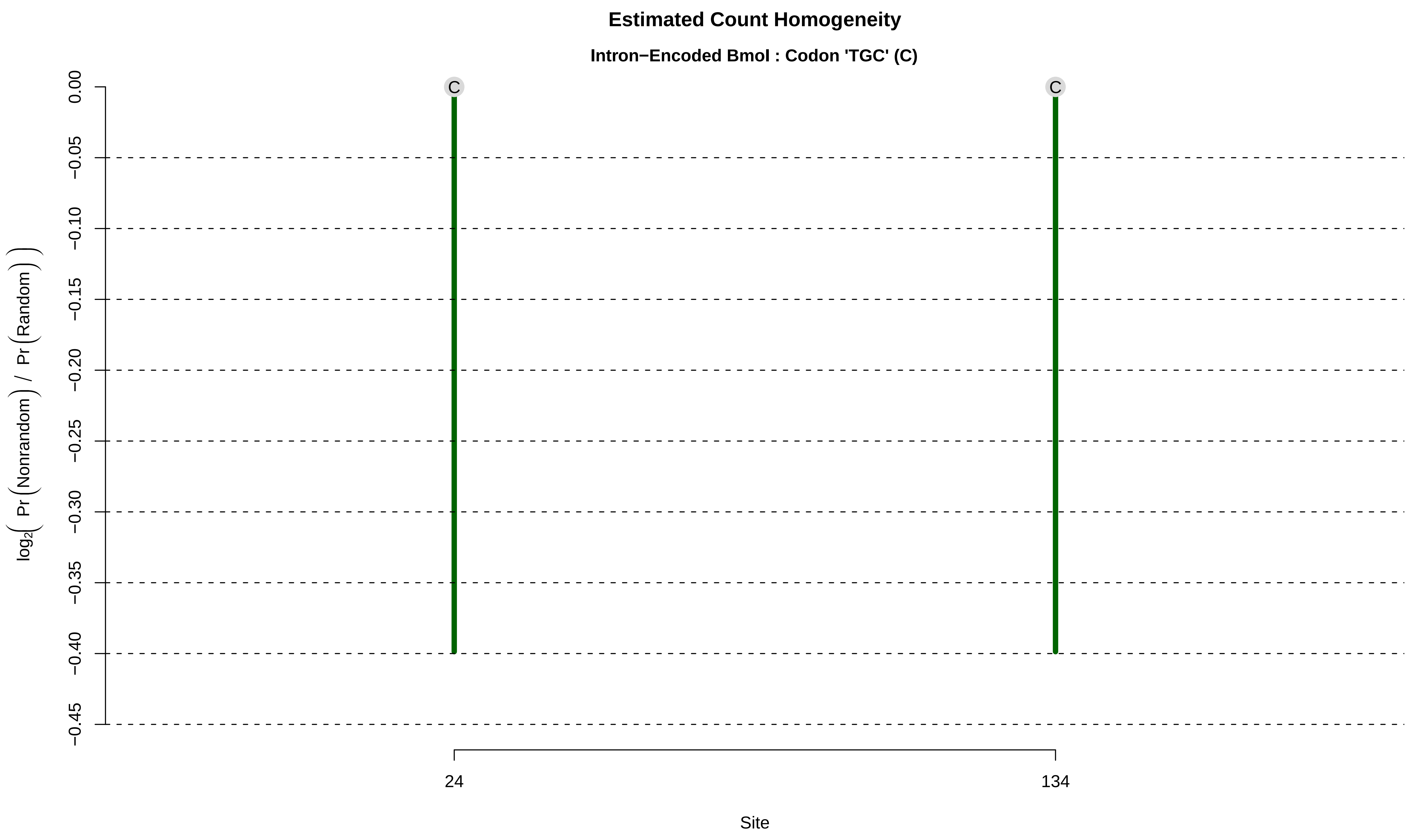
Estimated Count Homogeneity
Intron−Encoded BmoI : Codon 'TGC' (C)
0.00
−0.05
−0.10
−0.15
−0.20
−0.25
−0.30
−0.35
−0.40
−0.45
log2(Pr(Nonrandom) / Pr(Random))
C
C
24
134
Site

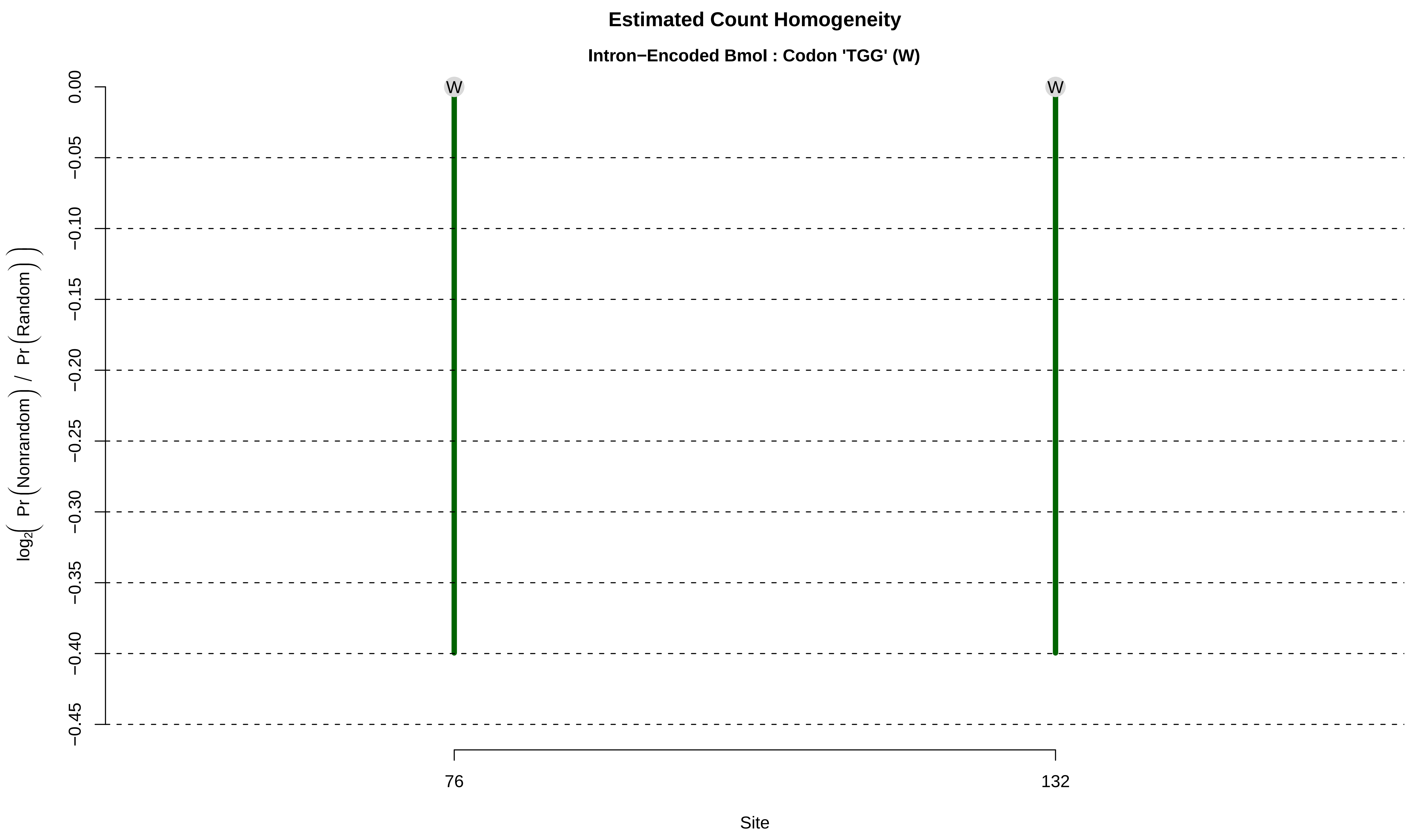
Estimated Count Homogeneity
Intron−Encoded BmoI : Codon 'TGG' (W)
log2(Pr(Nonrandom) / Pr(Random))
0.00
−0.05
−0.10
−0.15
−0.20
−0.25
−0.30
−0.35
−0.40
−0.45
W
W
76
132
Site

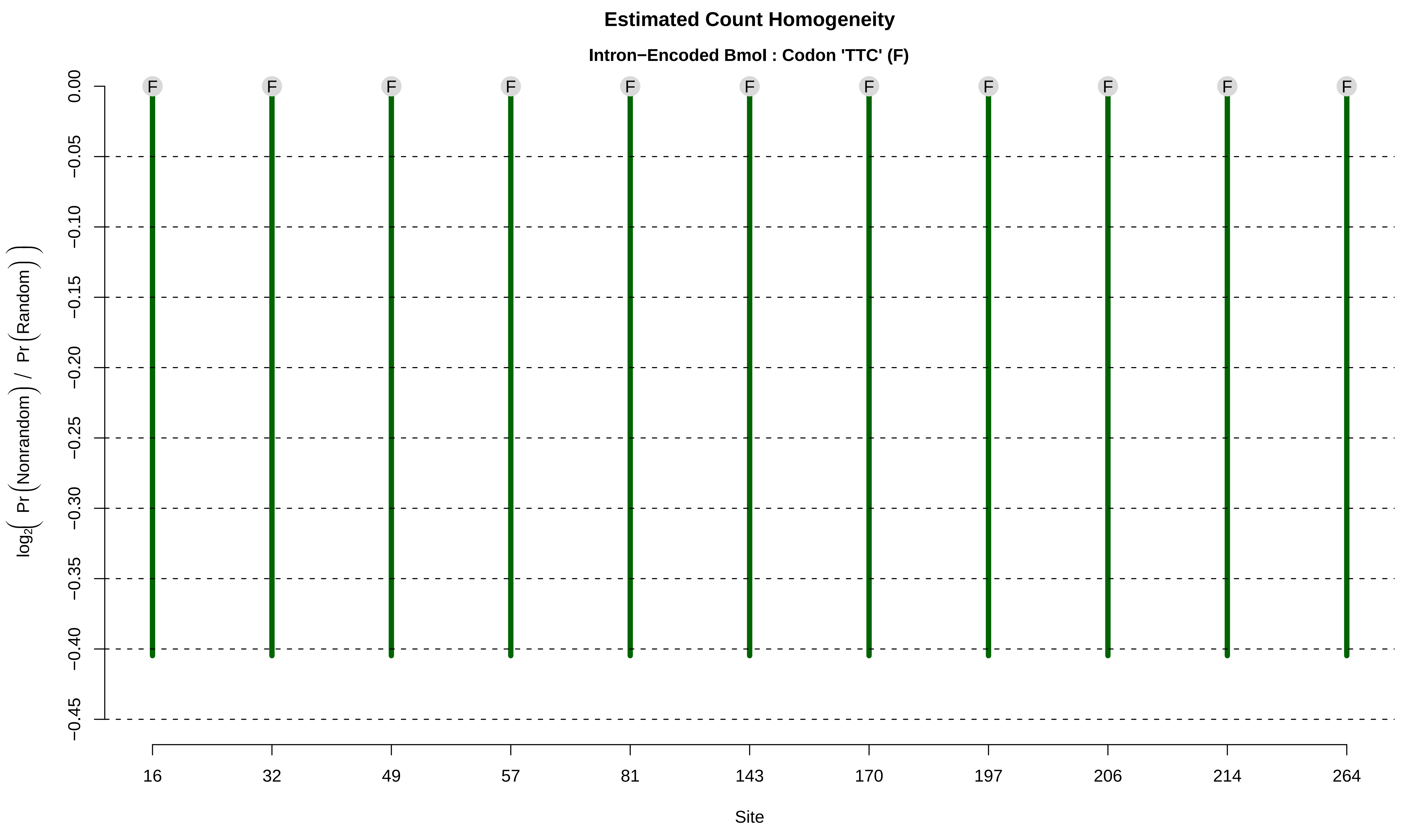

Estimated Count Homogeneity
Intron−Encoded BmoI : Codon 'TTC' (F)
0.00
−0.05
−0.10
−0.15
−0.20
−0.25
−0.30
−0.35
−0.40
−0.45
log2(Pr(Nonrandom) / Pr(Random))
F
16
32
49
57
81
143
170
197
206
214
264
Site
