# Supplementary material for: Estimating the evidence of selection and the reliability of inference in unigenic evolution
Source: Algorithms Mol Biol. 2010 Nov 8;5:35. doi: 10.1186/1748-7188-5-35 (PMC2994857; doi:10.1186/1748-7188-5-35)
Supplement: Additional file 7 — Sample Input and Output. Sample input, output, and driver files for the given software package. [file 1748-7188-5-35-S7.ZIP › unigenic_example/edgell.pol/by_codon_llr.pdf]

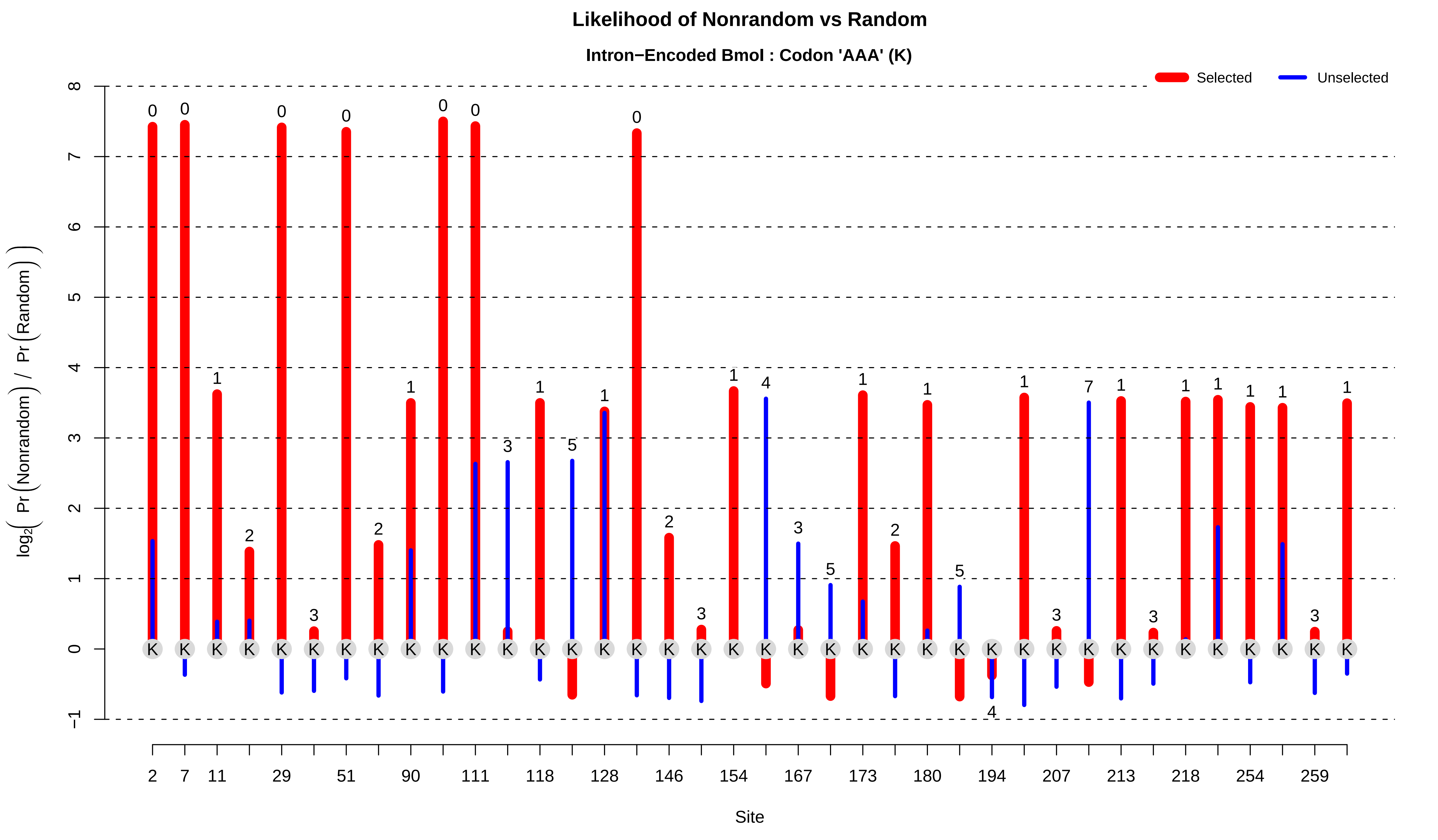
Likelihood of Nonrandom vs Random
Intron−Encoded BmoI : Codon 'AAA' (K)
Selected
Unselected
log2(Pr(Nonrandom) / Pr(Random))
Site
2 7 11 29 51 90 111 118 128 146 154 167 173 180 194 207 213 218 254 259

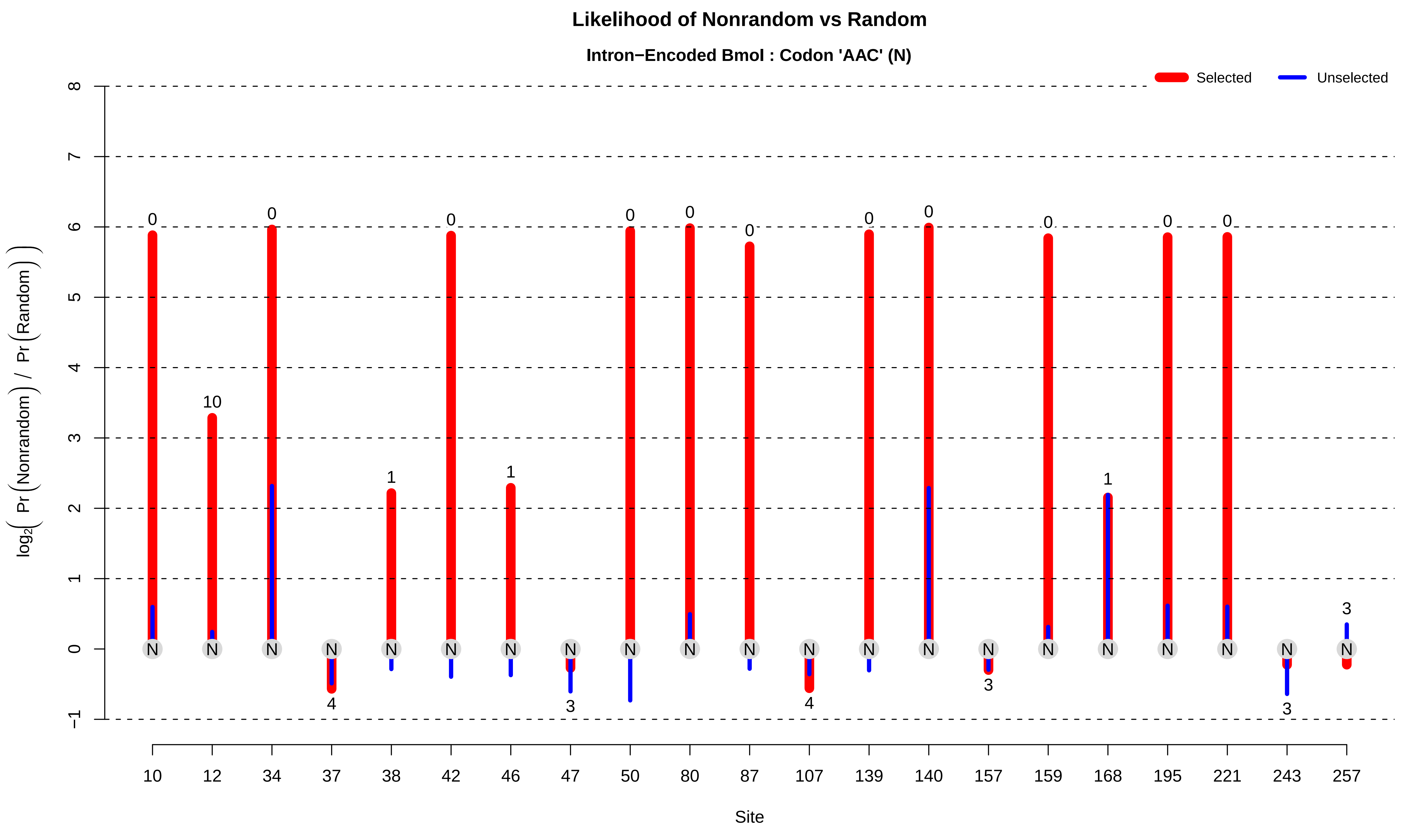

Likelihood of Nonrandom vs Random
Intron−Encoded BmoI : Codon 'AAC' (N)
Selected
Unselected
$\log_2\left(\Pr(\text{Nonrandom}) / \Pr(\text{Random})\right)$
Site
10 12 34 37 38 42 46 47 50 80 87 107 139 140 157 159 168 195 221 243 257
−1 0 1 2 3 4 5 6 7 8

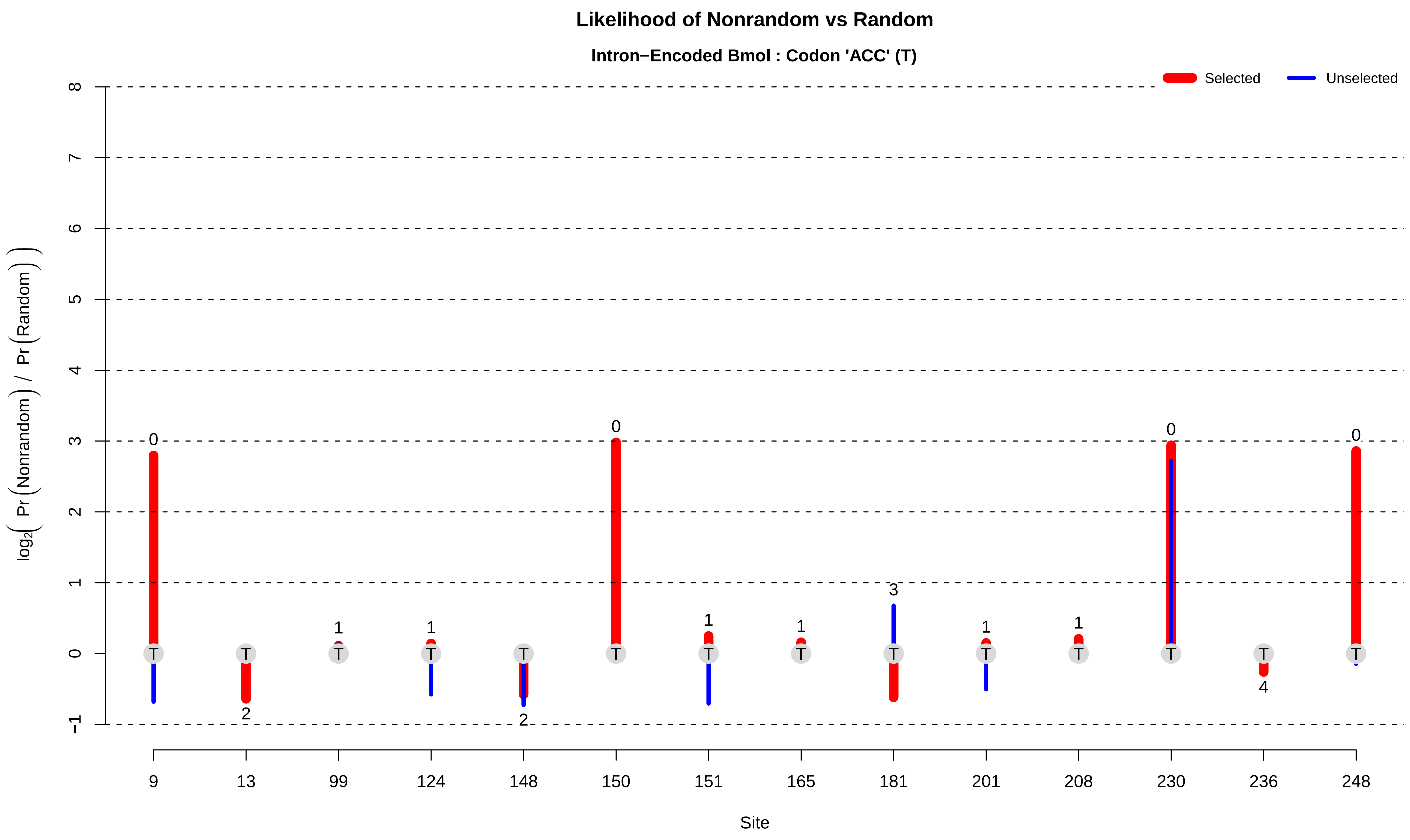

Likelihood of Nonrandom vs Random
Intron−Encoded BmoI : Codon 'ACC' (T)
Selected
Unselected
log2(Pr(Nonrandom) / Pr(Random))
Site
9
13
99
124
148
150
151
165
181
201
208
230
236
248

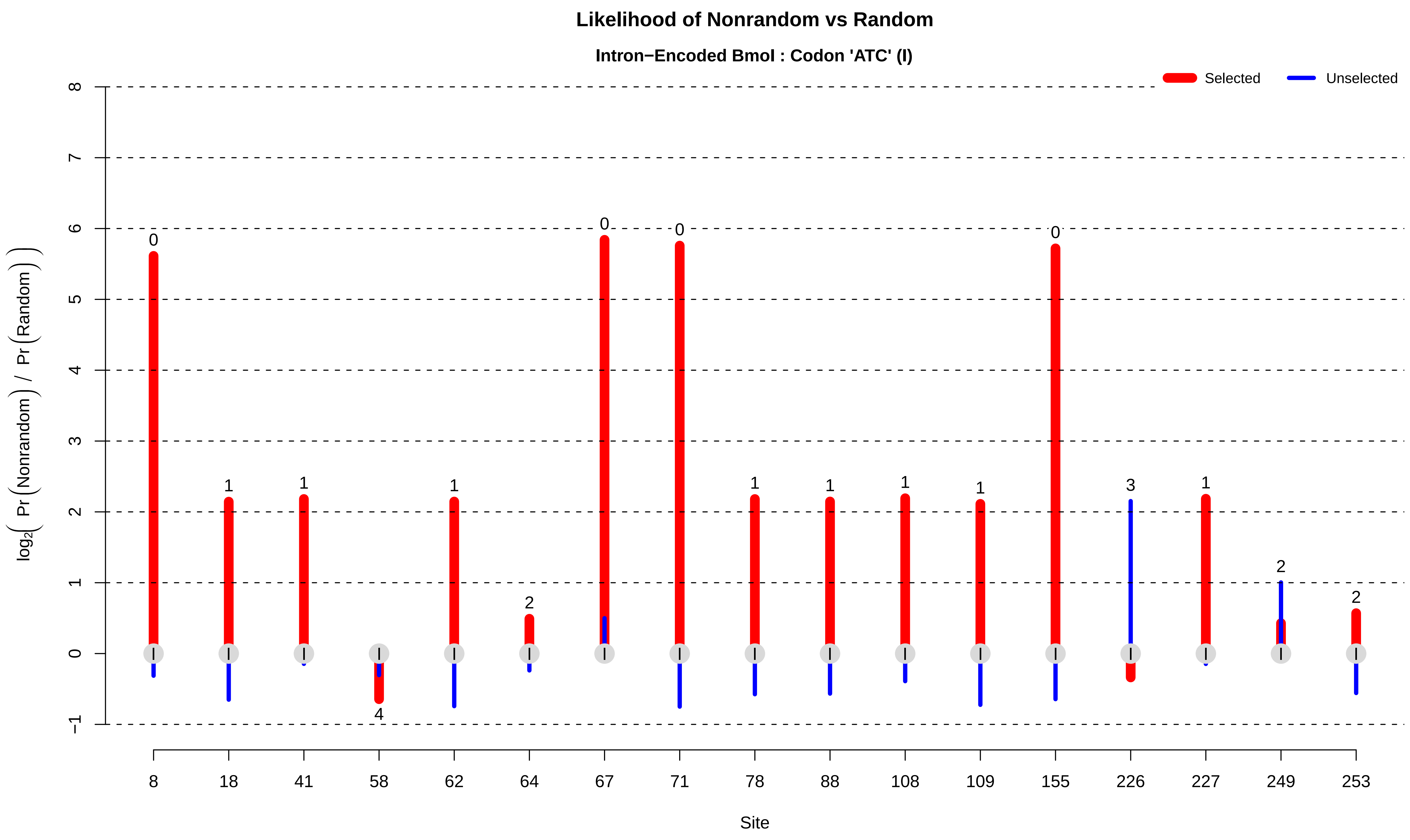

Likelihood of Nonrandom vs Random
Intron−Encoded BmoI : Codon 'ATC' (I)
Selected
Unselected
log2(Pr(Nonrandom) / Pr(Random))
Site
8
18
41
58
62
64
67
71
78
88
108
109
155
226
227
249
253

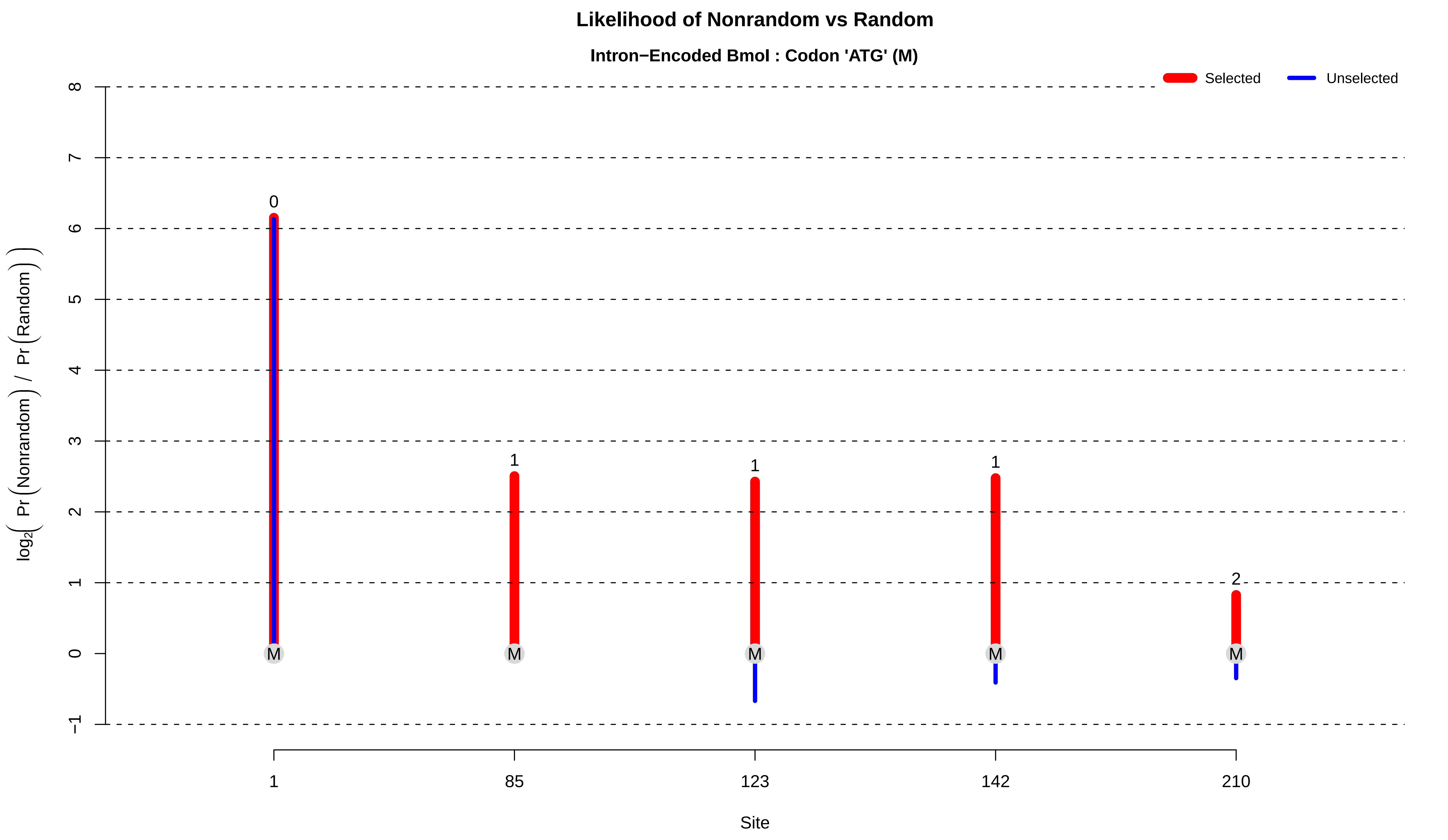
Likelihood of Nonrandom vs Random
Intron−Encoded BmoI : Codon 'ATG' (M)
Selected
Unselected
log2(Pr(Nonrandom) / Pr(Random))
8
7
6
5
4
3
2
1
0
−1
0
1
1
1
2
M
M
M
M
M
1
85
123
142
210
Site

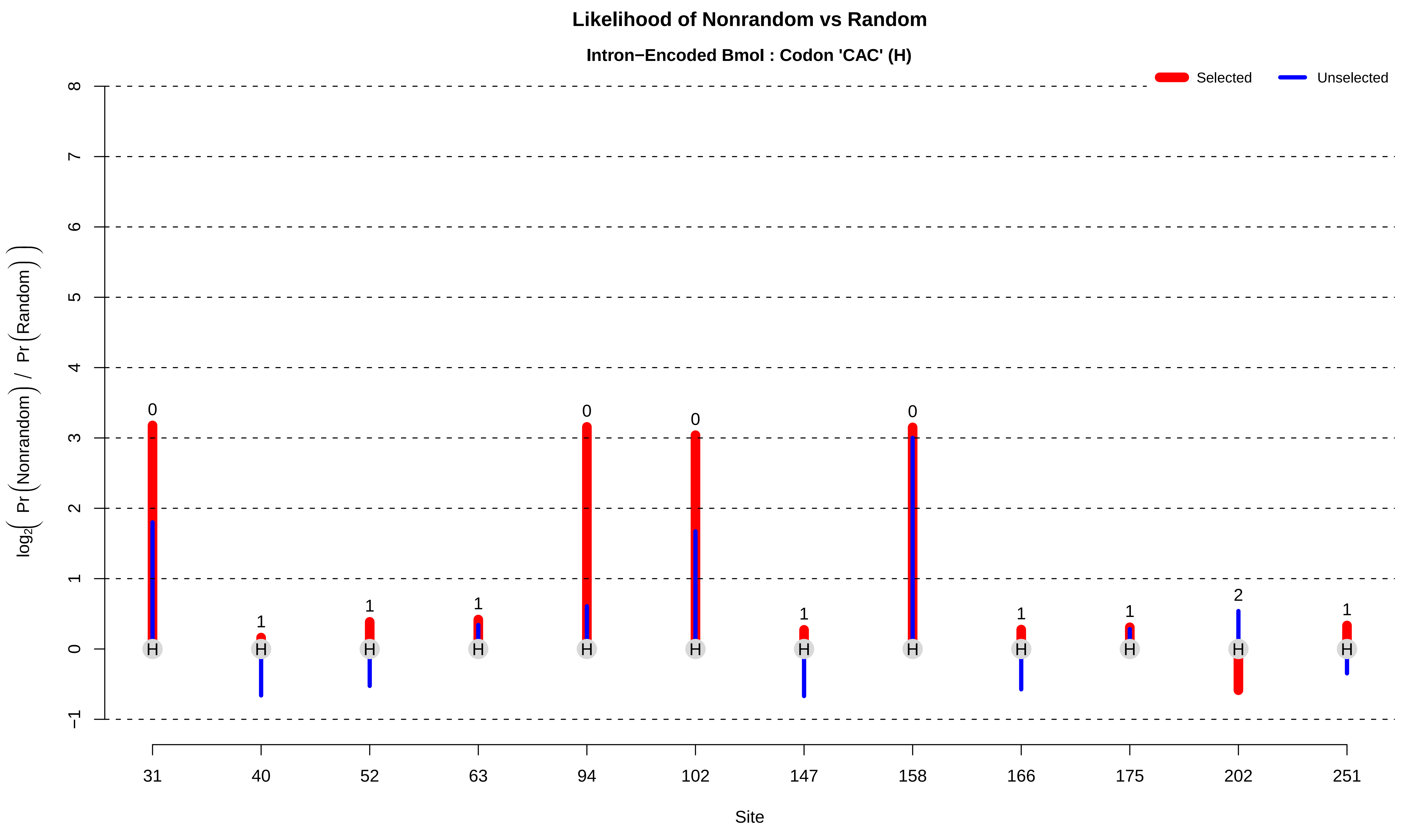

Likelihood of Nonrandom vs Random
Intron−Encoded BmoI : Codon 'CAC' (H)
Selected
Unselected
log2(Pr(Nonrandom) / Pr(Random))
Site
31
40
52
63
94
102
147
158
166
175
202
251

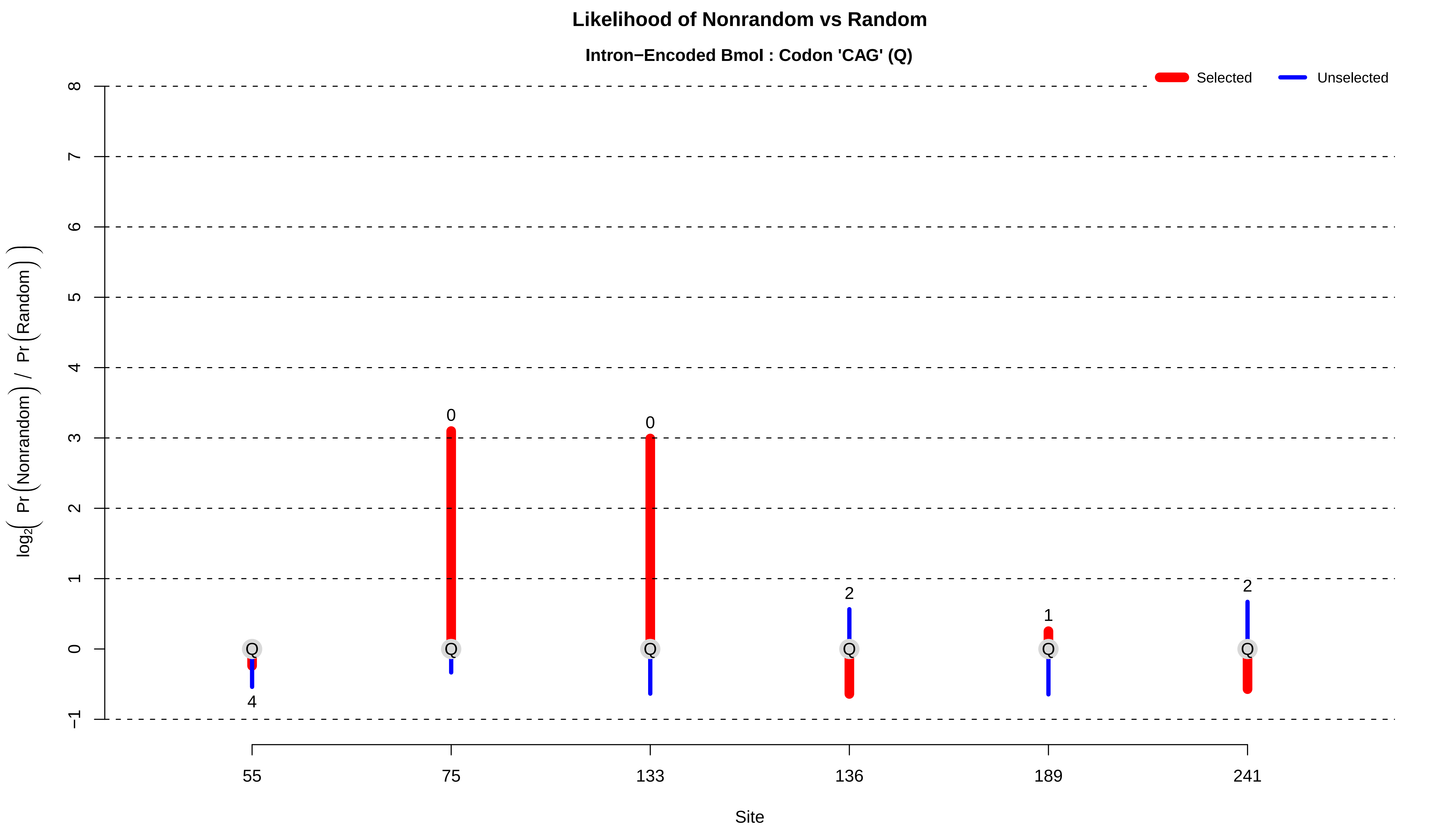

Likelihood of Nonrandom vs Random
Intron−Encoded BmoI : Codon 'CAG' (Q)
Selected
Unselected
log2(Pr(Nonrandom) / Pr(Random))
8
7
6
5
4
3
2
1
0
−1
0
0
2
1
2
4
Q
55
75
133
136
189
241
Site

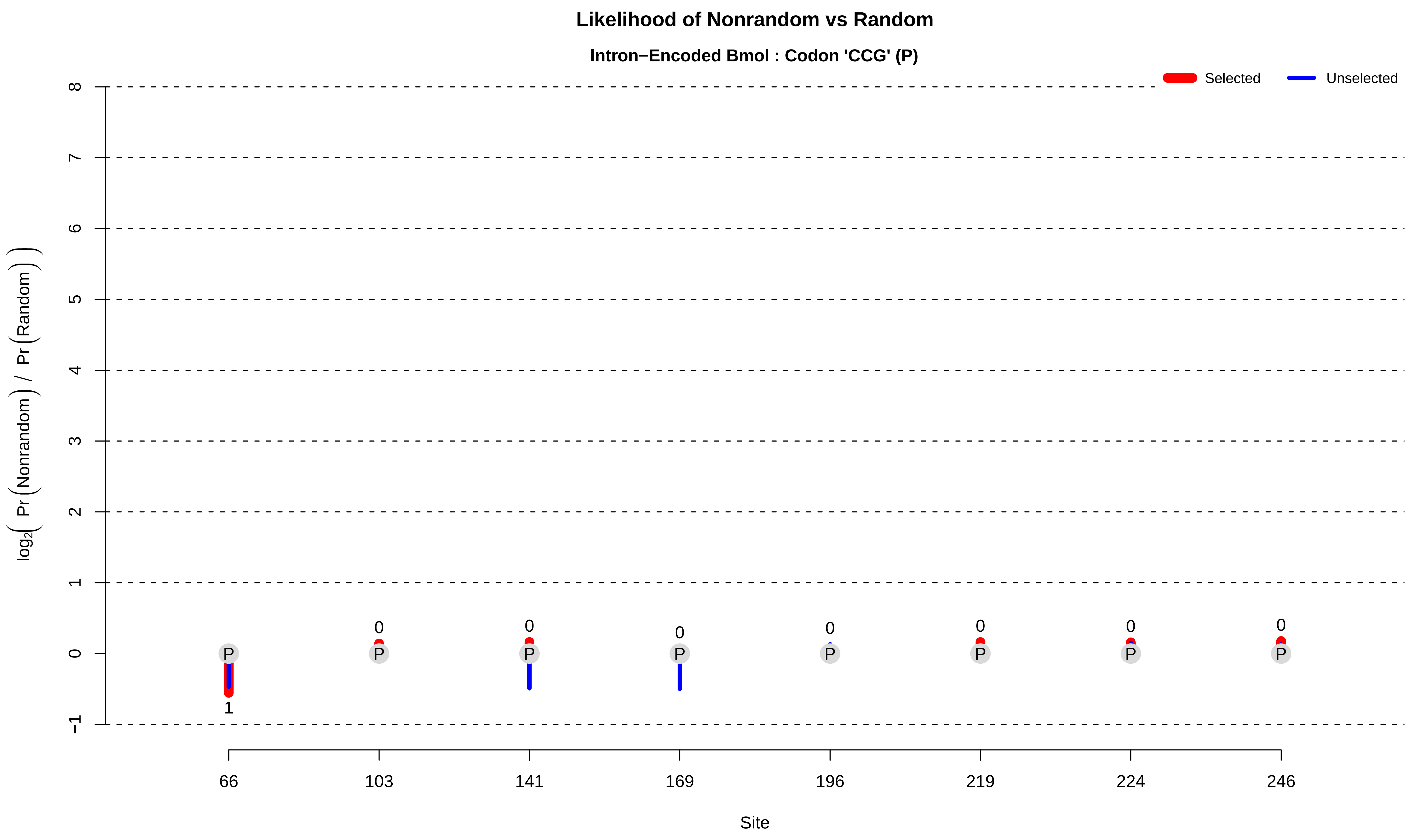
Likelihood of Nonrandom vs Random
Intron−Encoded BmoI : Codon 'CCG' (P)
Selected
Unselected
log2(Pr(Nonrandom) / Pr(Random))
8
7
6
5
4
3
2
1
0
−1
P
1
0
66
103
141
169
196
219
224
246
Site

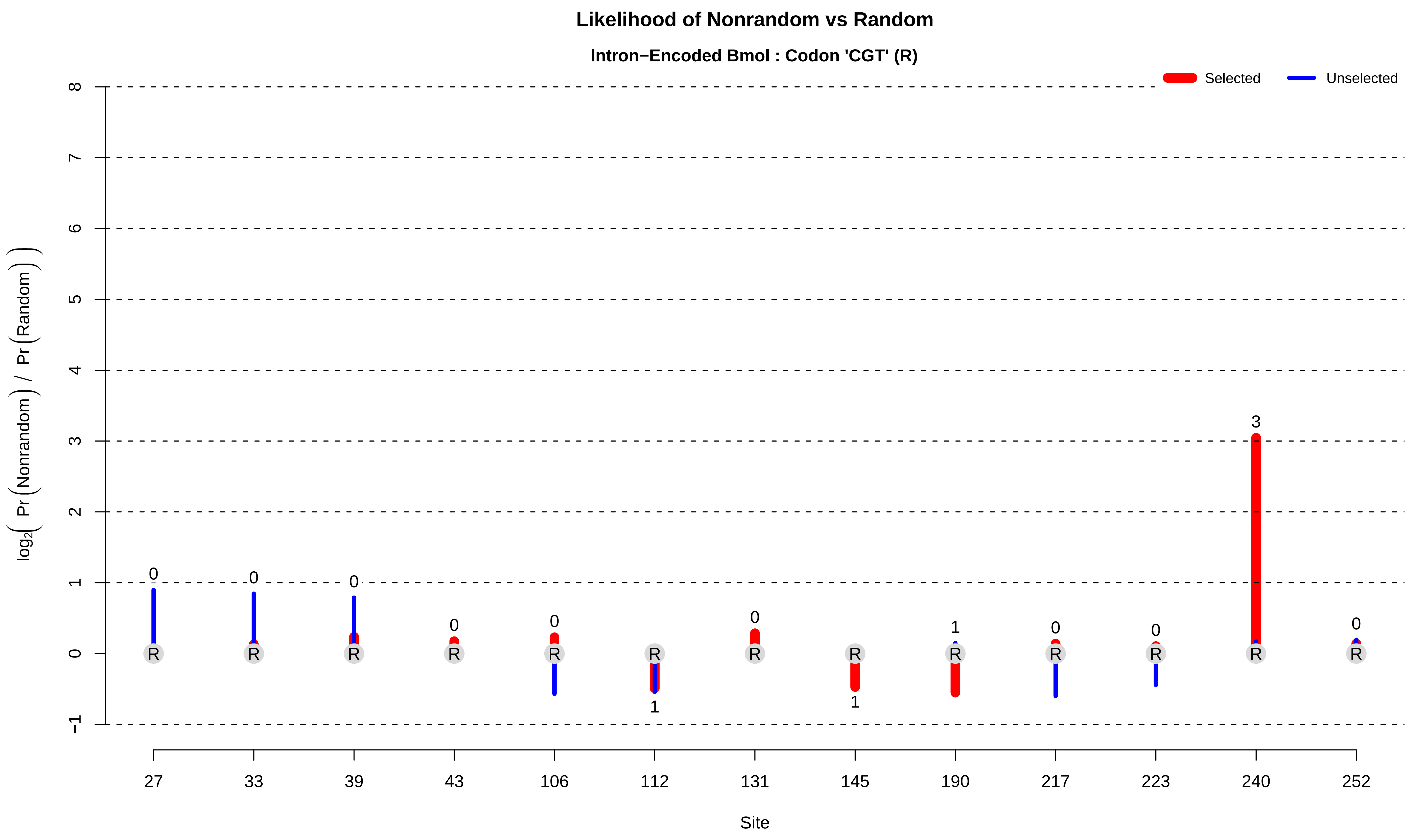

Likelihood of Nonrandom vs Random
Intron−Encoded BmoI : Codon 'CGT' (R)
Selected
Unselected
log2(Pr(Nonrandom) / Pr(Random))
8
7
6
5
4
3
2
1
0
−1
0
0
0
0
0
1
0
1
1
0
0
3
0
R
27
33
39
43
106
112
131
145
190
217
223
240
252
Site

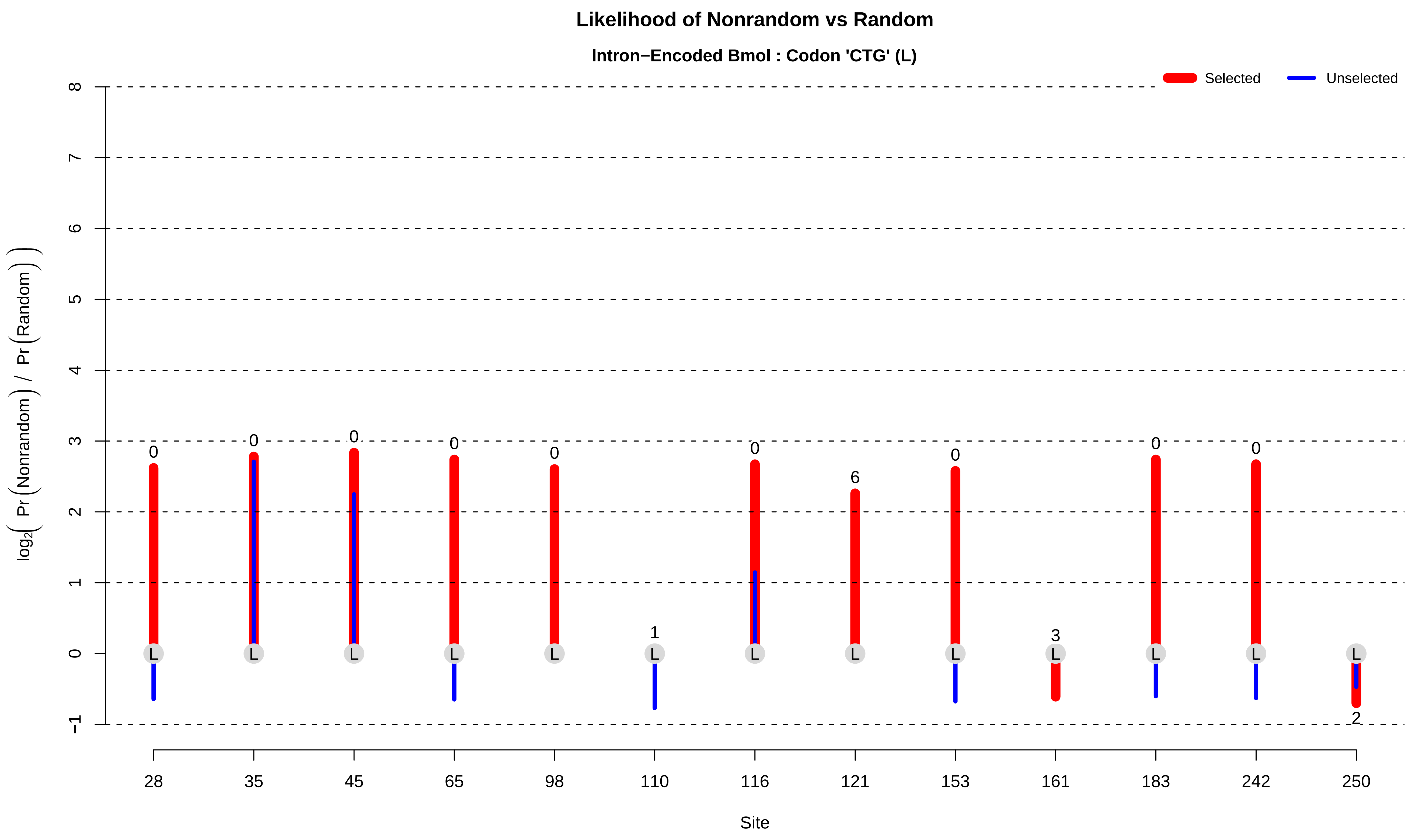
Likelihood of Nonrandom vs Random
Intron−Encoded BmoI : Codon 'CTG' (L)
Selected
Unselected
$\log_2\left(\Pr(\text{Nonrandom}) / \Pr(\text{Random})\right)$
8
7
6
5
4
3
2
1
0
−1
0
0
0
0
0
1
0
6
0
3
0
0
2
L
28
35
45
65
98
110
116
121
153
161
183
242
250
Site

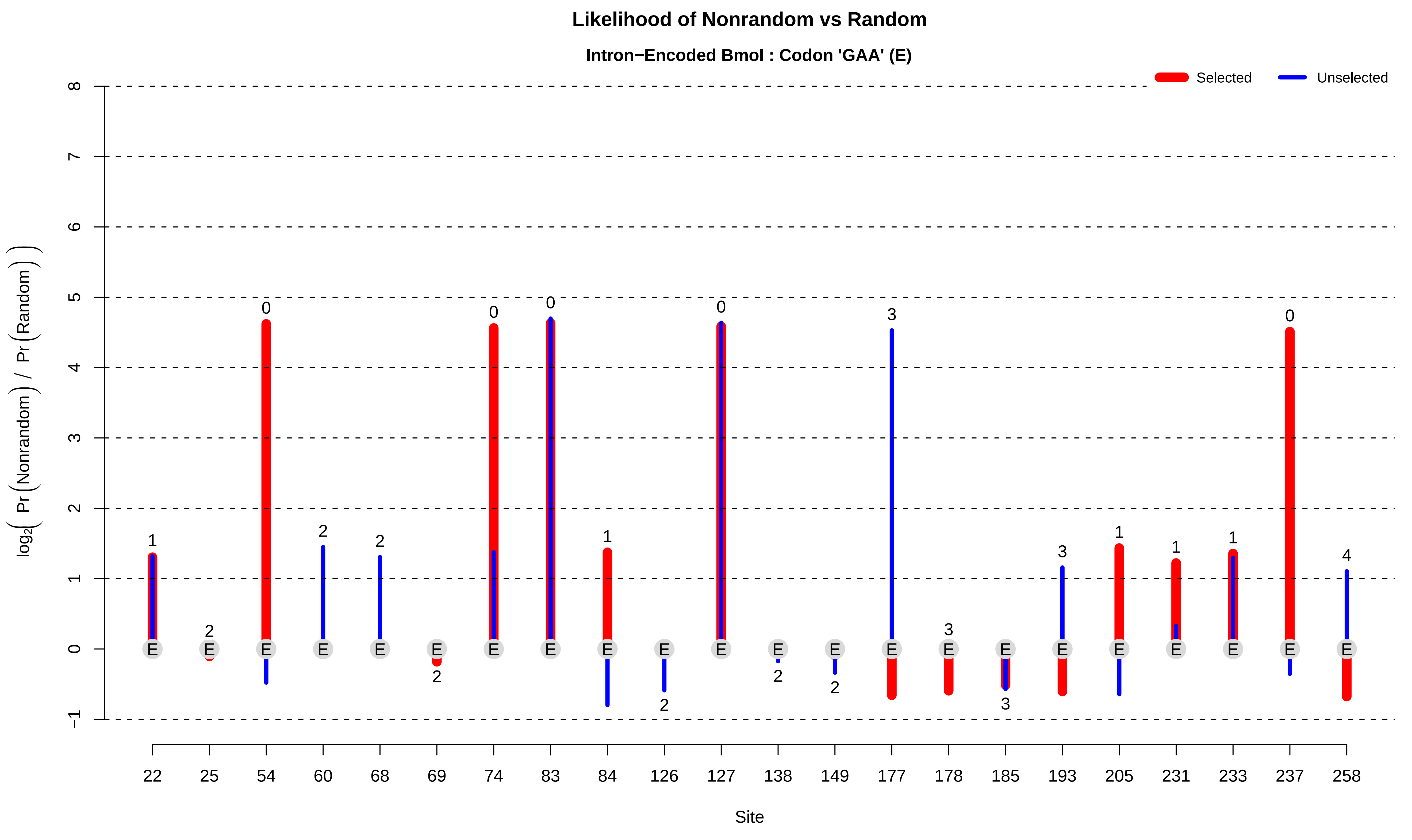
Likelihood of Nonrandom vs Random
Intron−Encoded BmoI : Codon 'GAA' (E)
Selected
Unselected
log2(Pr(Nonrandom) / Pr(Random))
Site
22 25 54 60 68 69 74 83 84 126 127 138 149 177 178 185 193 205 231 233 237 258

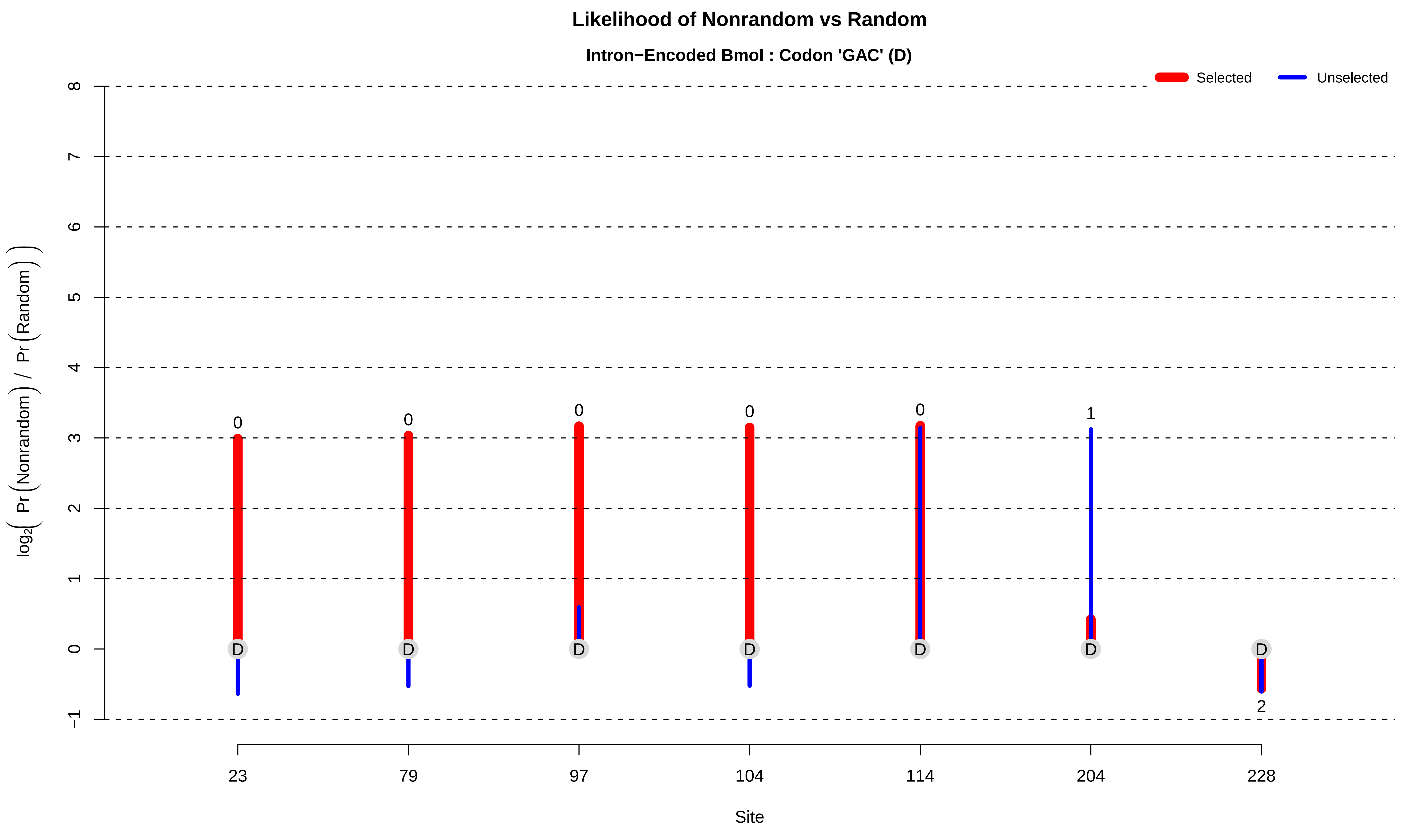
Likelihood of Nonrandom vs Random
Intron−Encoded BmoI : Codon 'GAC' (D)
Selected
Unselected
log2(Pr(Nonrandom) / Pr(Random))
8
7
6
5
4
3
2
1
0
−1
0
0
0
0
0
1
2
D
D
D
D
D
D
D
23
79
97
104
114
204
228
Site

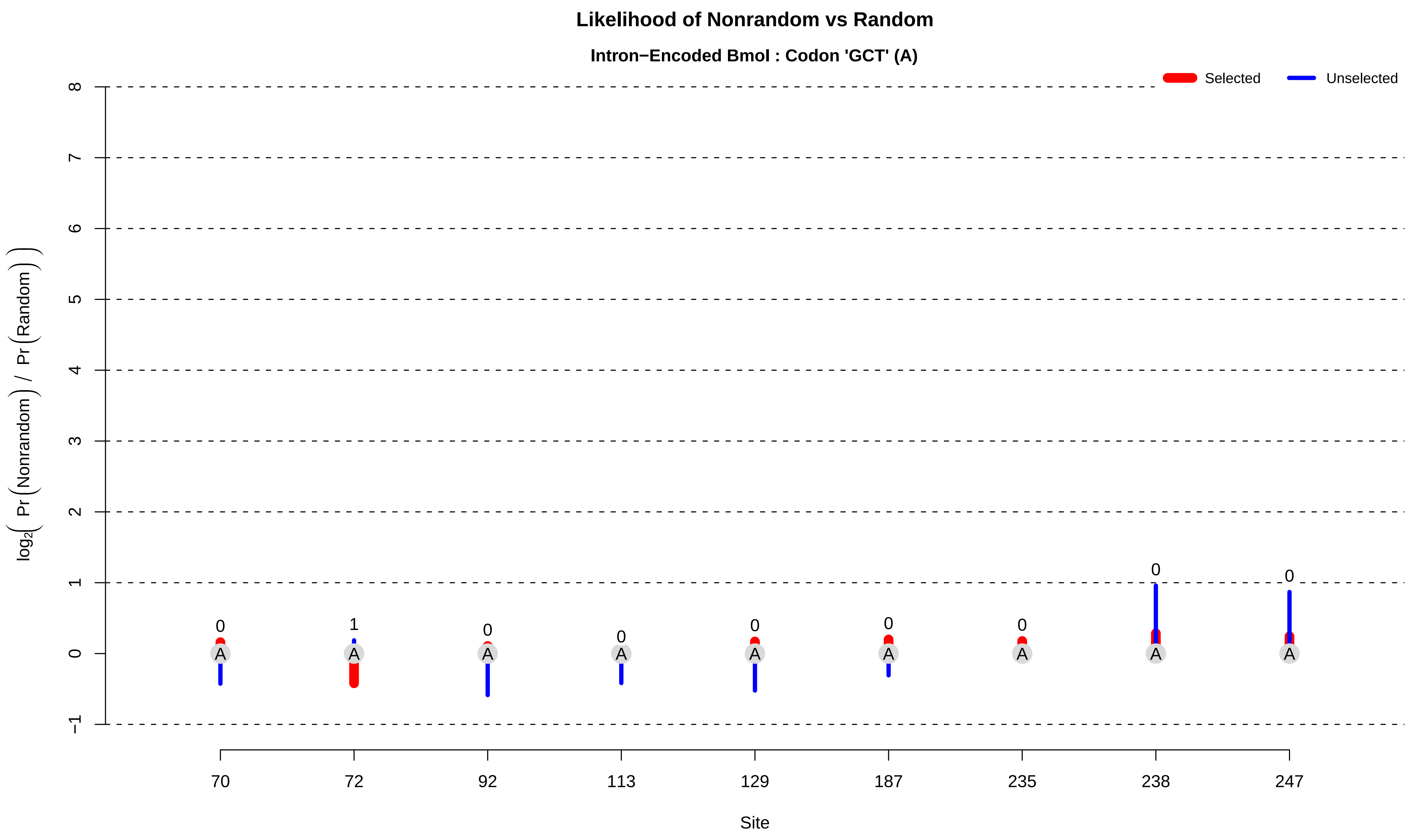

Likelihood of Nonrandom vs Random
Intron−Encoded BmoI : Codon 'GCT' (A)
Selected
Unselected
log2(Pr(Nonrandom) / Pr(Random))
8
7
6
5
4
3
2
1
0
−1
0
1
0
0
0
0
0
0
0
A
70
72
92
113
129
187
235
238
247
Site

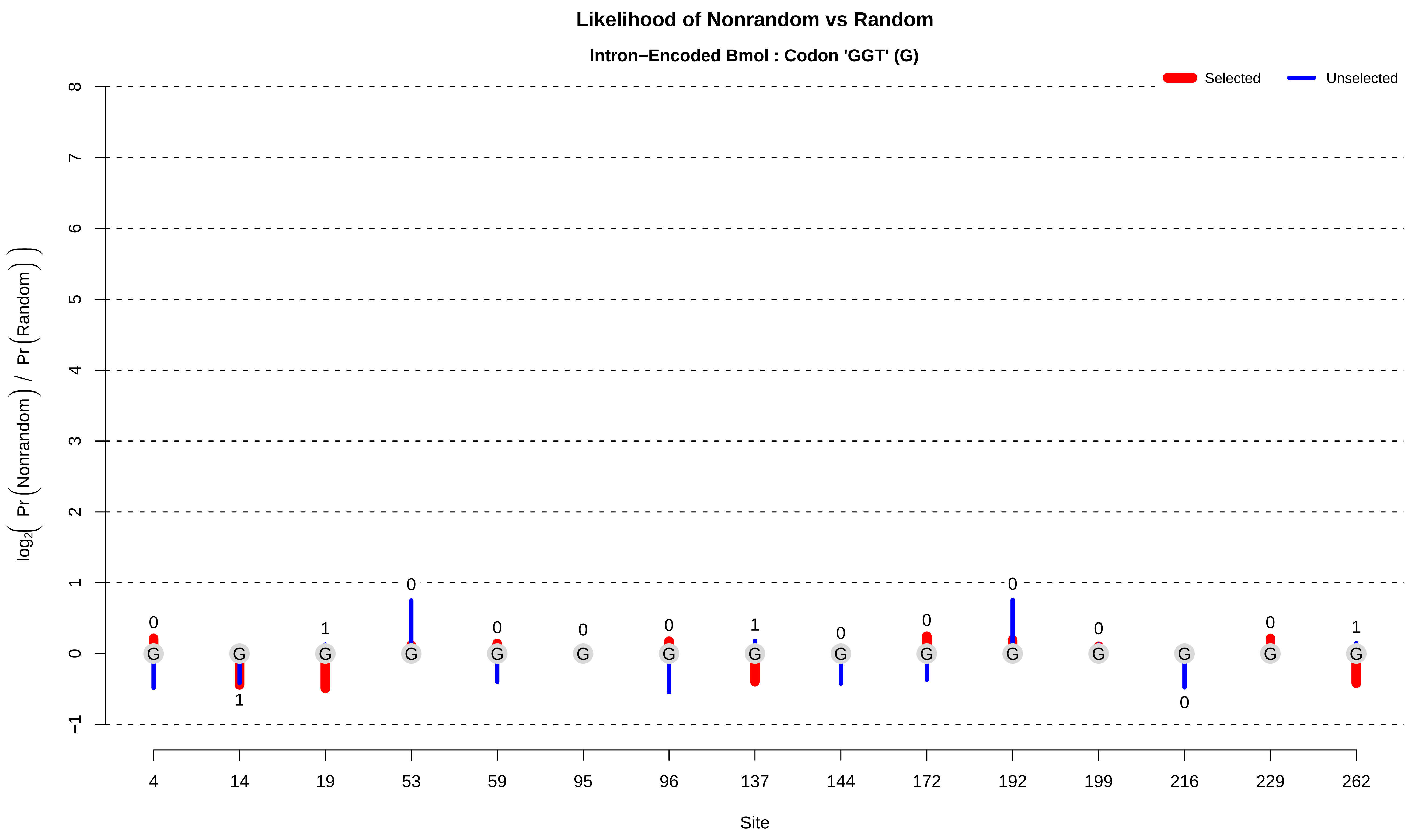

Likelihood of Nonrandom vs Random
Intron−Encoded BmoI : Codon 'GGT' (G)
Selected
Unselected
$\log_2\left(\Pr\left(\text{Nonrandom}\right) / \Pr\left(\text{Random}\right)\right)$
8
7
6
5
4
3
2
1
0
−1
0
1
1
0
0
0
0
1
0
0
0
0
0
0
1
G
4
14
19
53
59
95
96
137
144
172
192
199
216
229
262
Site

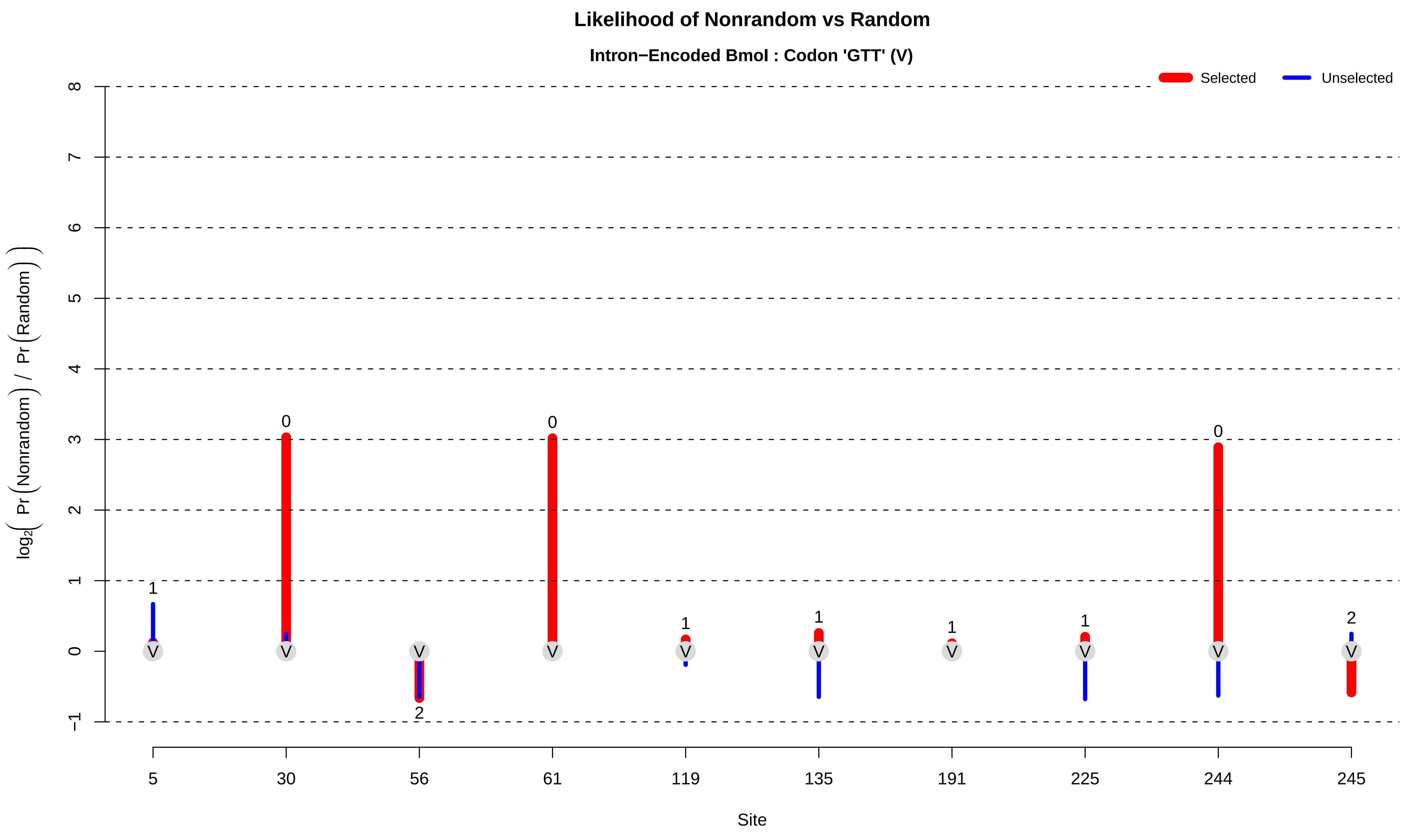
Likelihood of Nonrandom vs Random
Intron−Encoded BmoI : Codon 'GTT' (V)
Selected
Unselected
log2(Pr(Nonrandom) / Pr(Random))
−1
0
1
2
3
4
5
6
7
8
Site
5
30
56
61
119
135
191
225
244
245

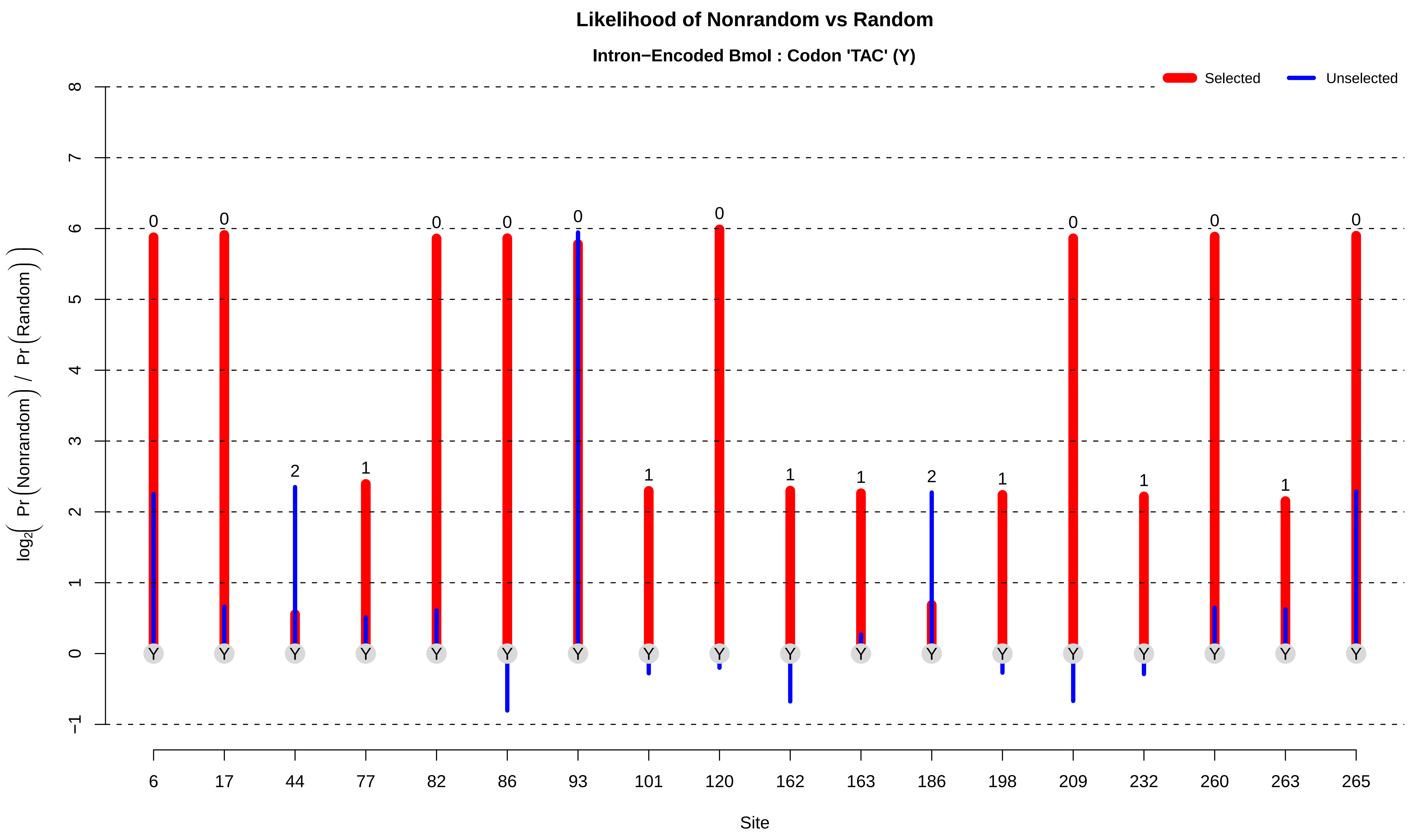

Likelihood of Nonrandom vs Random
Intron−Encoded BmoI : Codon 'TAC' (Y)
Selected
Unselected
log2(Pr(Nonrandom) / Pr(Random))
Site
6
17
44
77
82
86
93
101
120
162
163
186
198
209
232
260
263
265

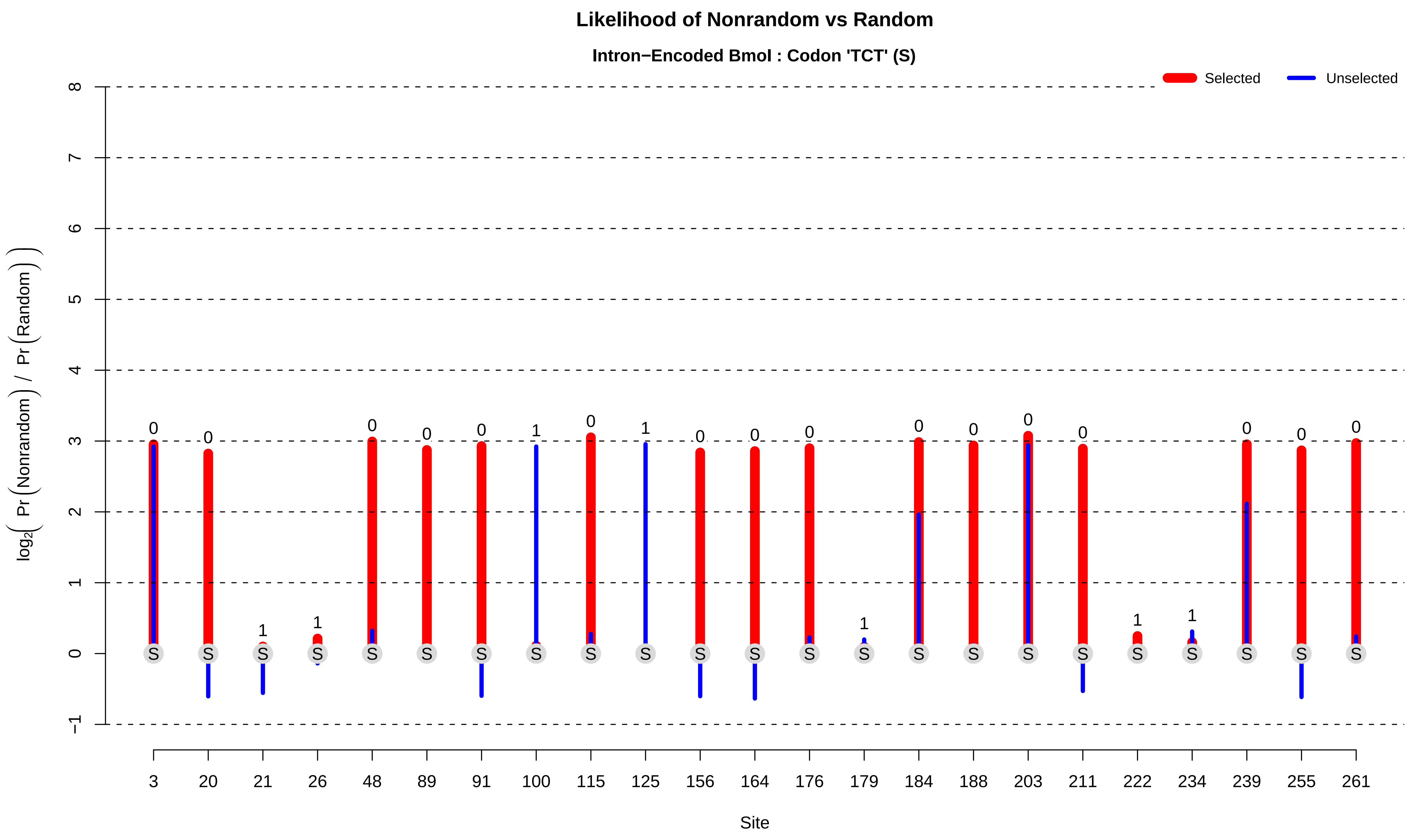

Likelihood of Nonrandom vs Random
Intron−Encoded BmoI : Codon 'TCT' (S)
Selected
Unselected
log2(Pr(Nonrandom) / Pr(Random))
Site
3 20 21 26 48 89 91 100 115 125 156 164 176 179 184 188 203 211 222 234 239 255 261

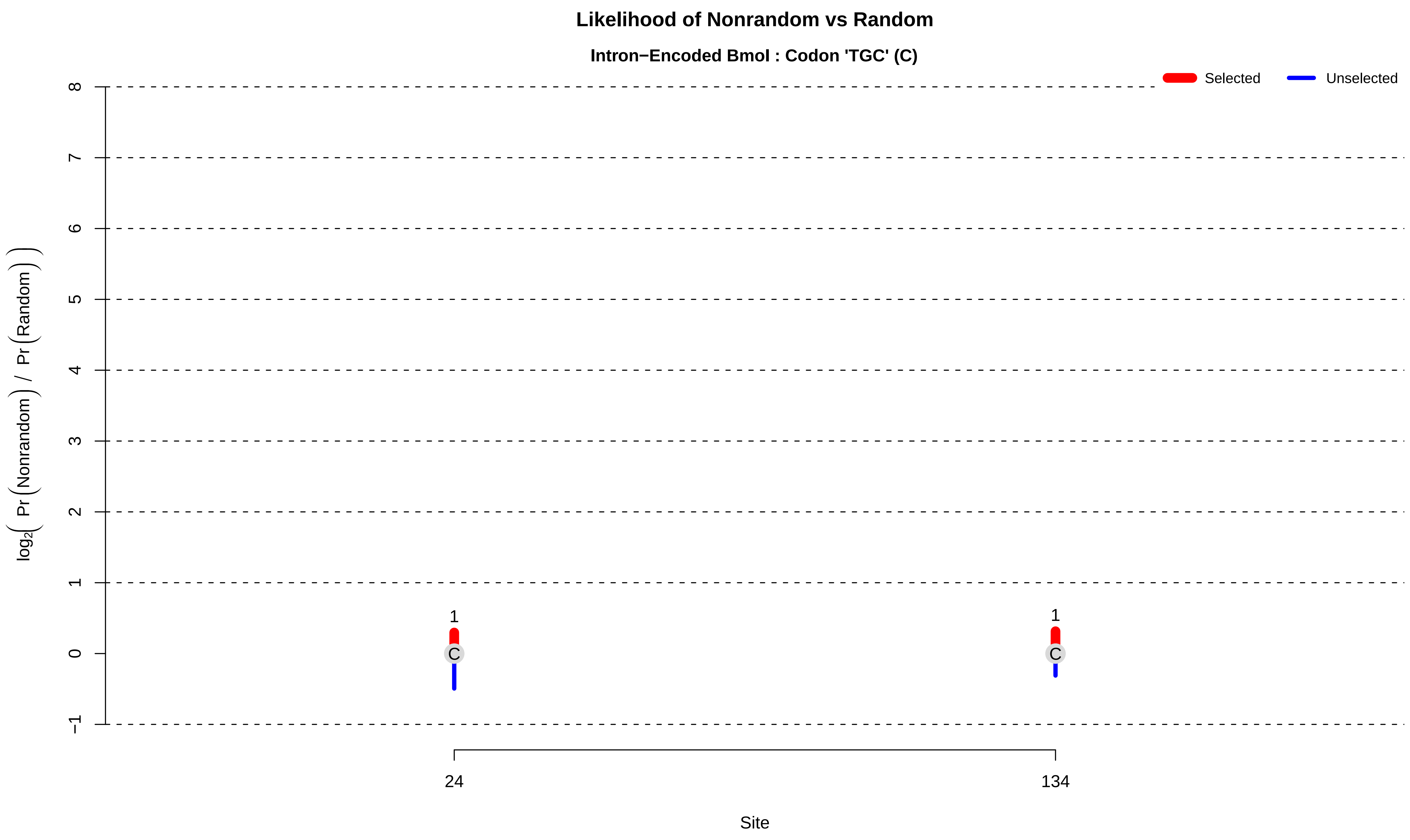

Likelihood of Nonrandom vs Random
Intron−Encoded BmoI : Codon 'TGC' (C)
Selected
Unselected
log2(Pr(Nonrandom) / Pr(Random))
8
7
6
5
4
3
2
1
0
−1
1
C
1
C
24
134
Site

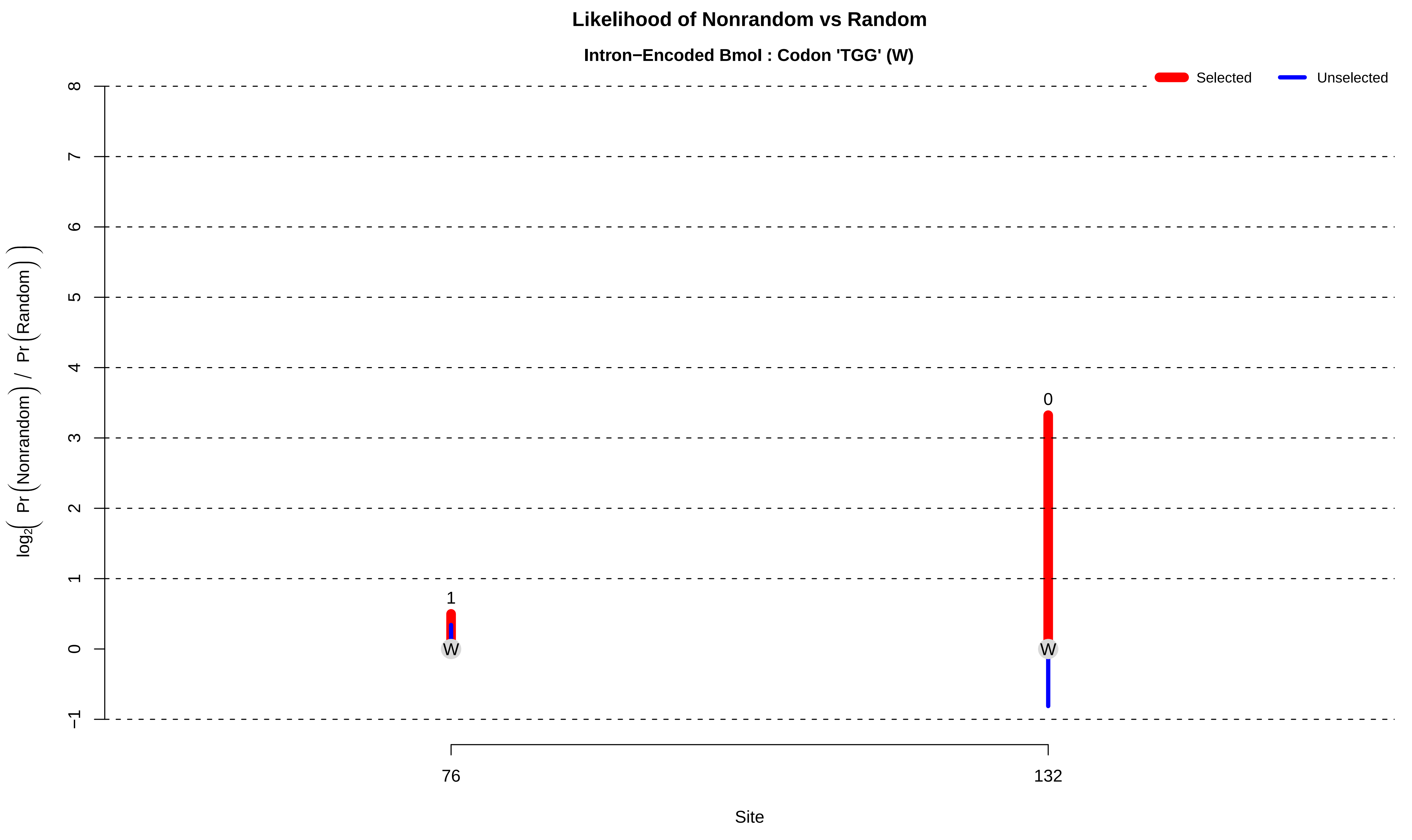
Likelihood of Nonrandom vs Random
Intron−Encoded BmoI : Codon 'TGG' (W)
Selected
Unselected
$\log_2\left(\Pr\left(\text{Nonrandom}\right) / \Pr\left(\text{Random}\right)\right)$
8
7
6
5
4
3
2
1
0
−1
1
0
W
W
76
132
Site

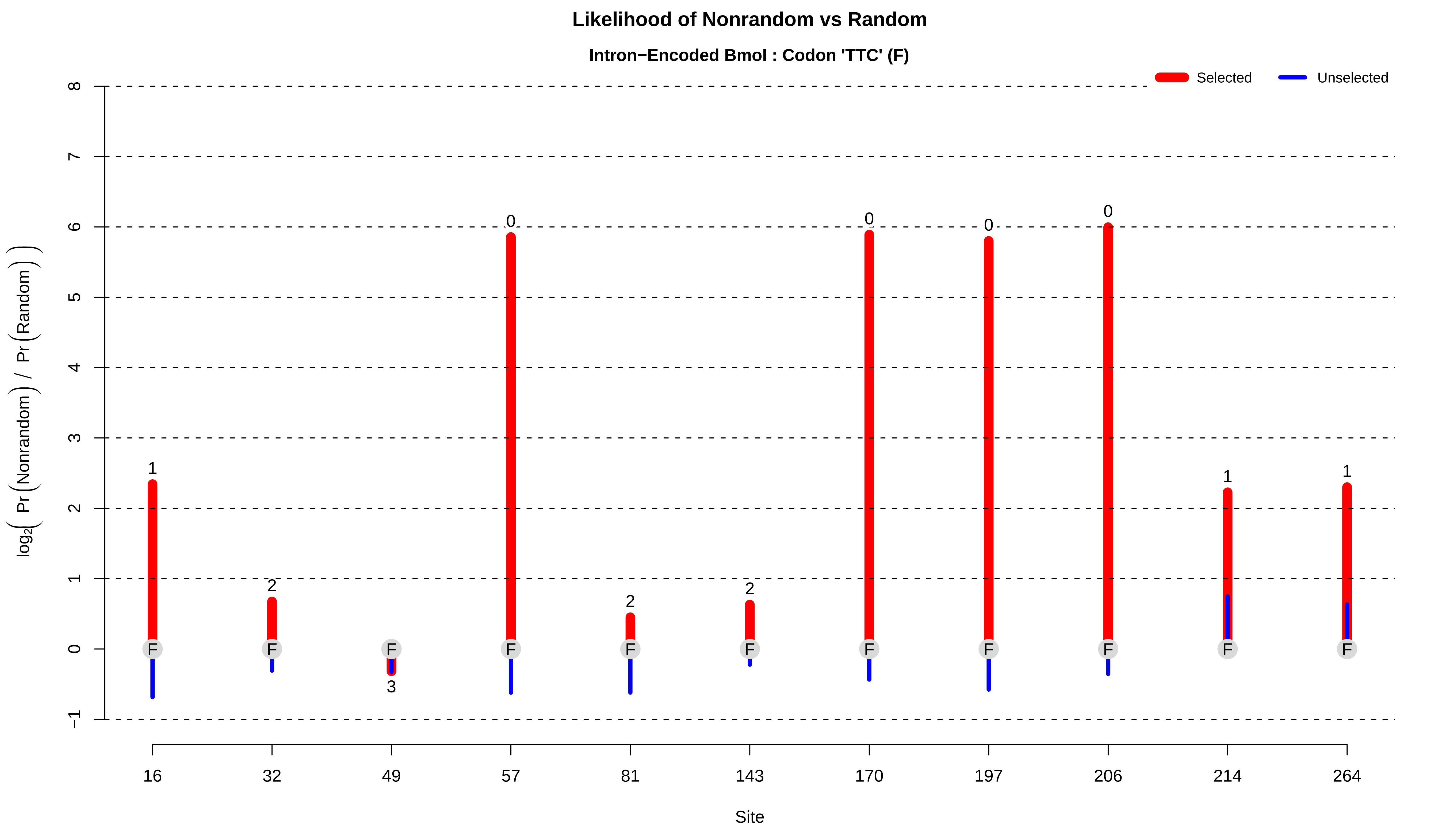

Likelihood of Nonrandom vs Random
Intron−Encoded BmoI : Codon 'TTC' (F)
Selected
Unselected
log2(Pr(Nonrandom) / Pr(Random))
−1
0
1
2
3
4
5
6
7
8
Site
16
32
49
57
81
143
170
197
206
214
264
1
2
3
0
2
2
0
0
0
1
1
F
