# Supplementary material for: Estimating the evidence of selection and the reliability of inference in unigenic evolution
Source: Algorithms Mol Biol. 2010 Nov 8;5:35. doi: 10.1186/1748-7188-5-35 (PMC2994857; doi:10.1186/1748-7188-5-35)
Supplement: Additional file 7 — Sample Input and Output. Sample input, output, and driver files for the given software package. [file 1748-7188-5-35-S7.ZIP › unigenic_example/edgell.pol/by_codon_pus.pdf]

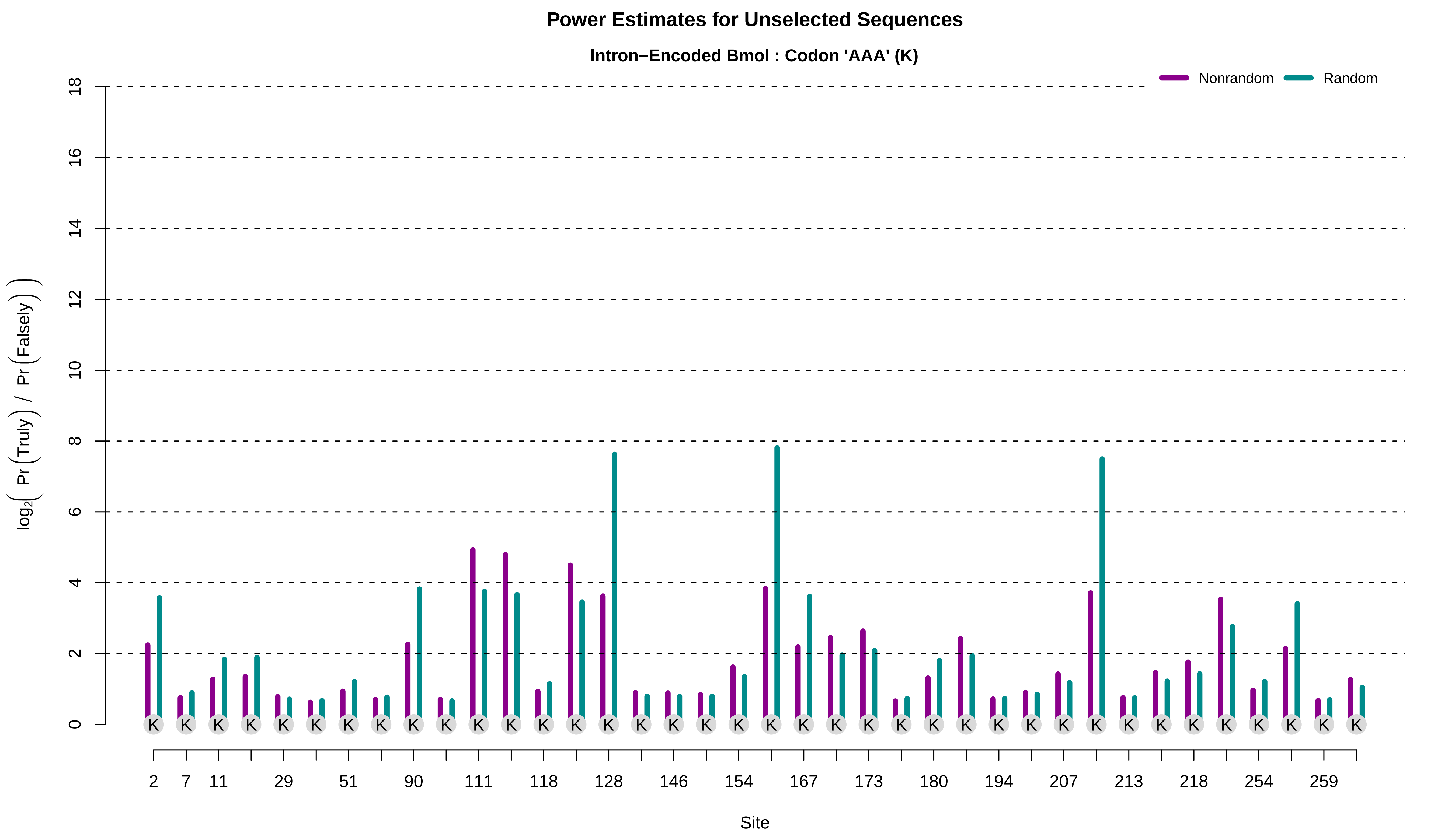

Power Estimates for Unselected Sequences
Intron−Encoded BmoI : Codon 'AAA' (K)
Nonrandom
Random
log2(Pr(Truly) / Pr(Falsely))
0
2
4
6
8
10
12
14
16
18
K
2
7
11
29
51
90
111
118
128
146
154
167
173
180
194
207
213
218
254
259
Site

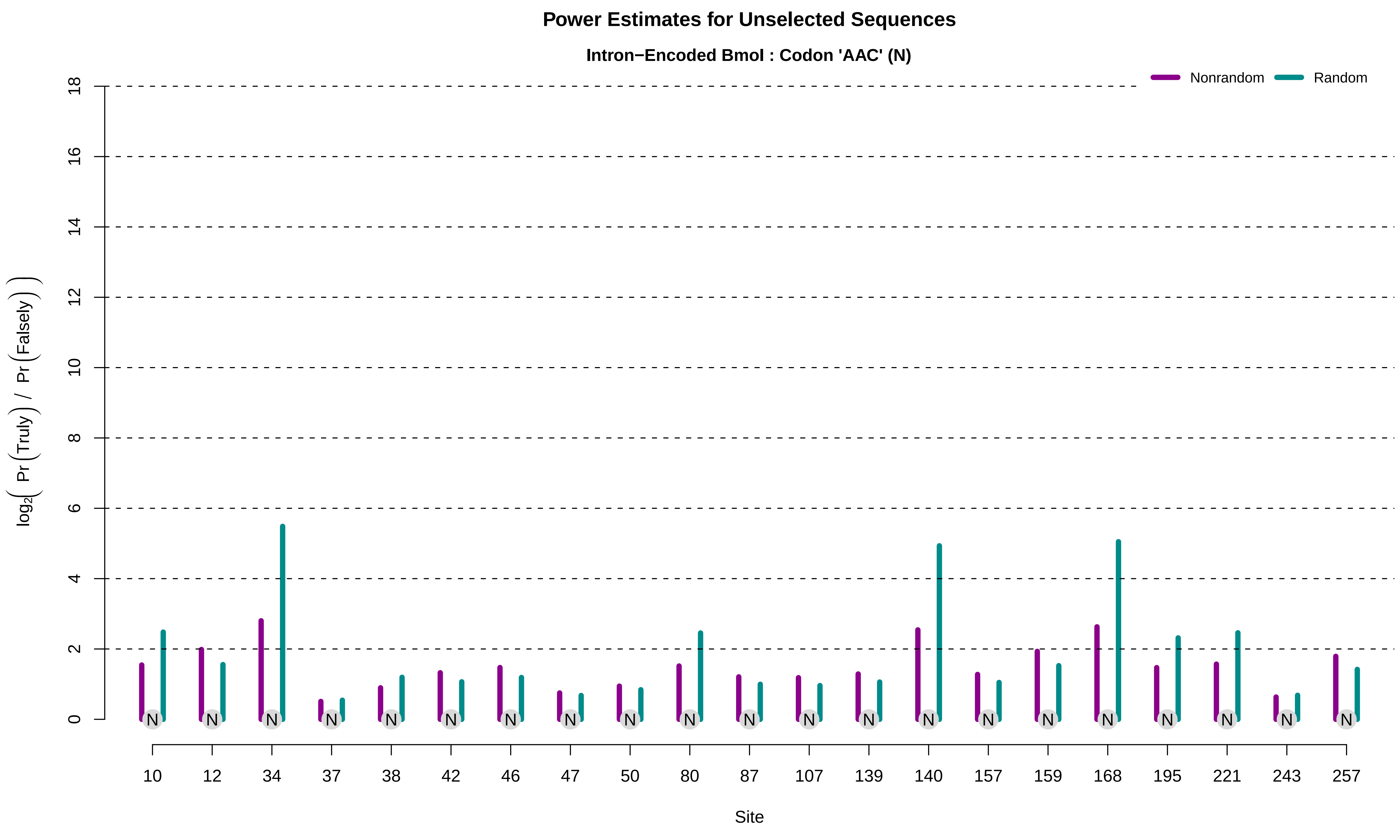
Power Estimates for Unselected Sequences
Intron−Encoded BmoI : Codon 'AAC' (N)
Nonrandom
Random
log2(Pr(Truly) / Pr(Falsely))
0
2
4
6
8
10
12
14
16
18
N
10
12
34
37
38
42
46
47
50
80
87
107
139
140
157
159
168
195
221
243
257
Site

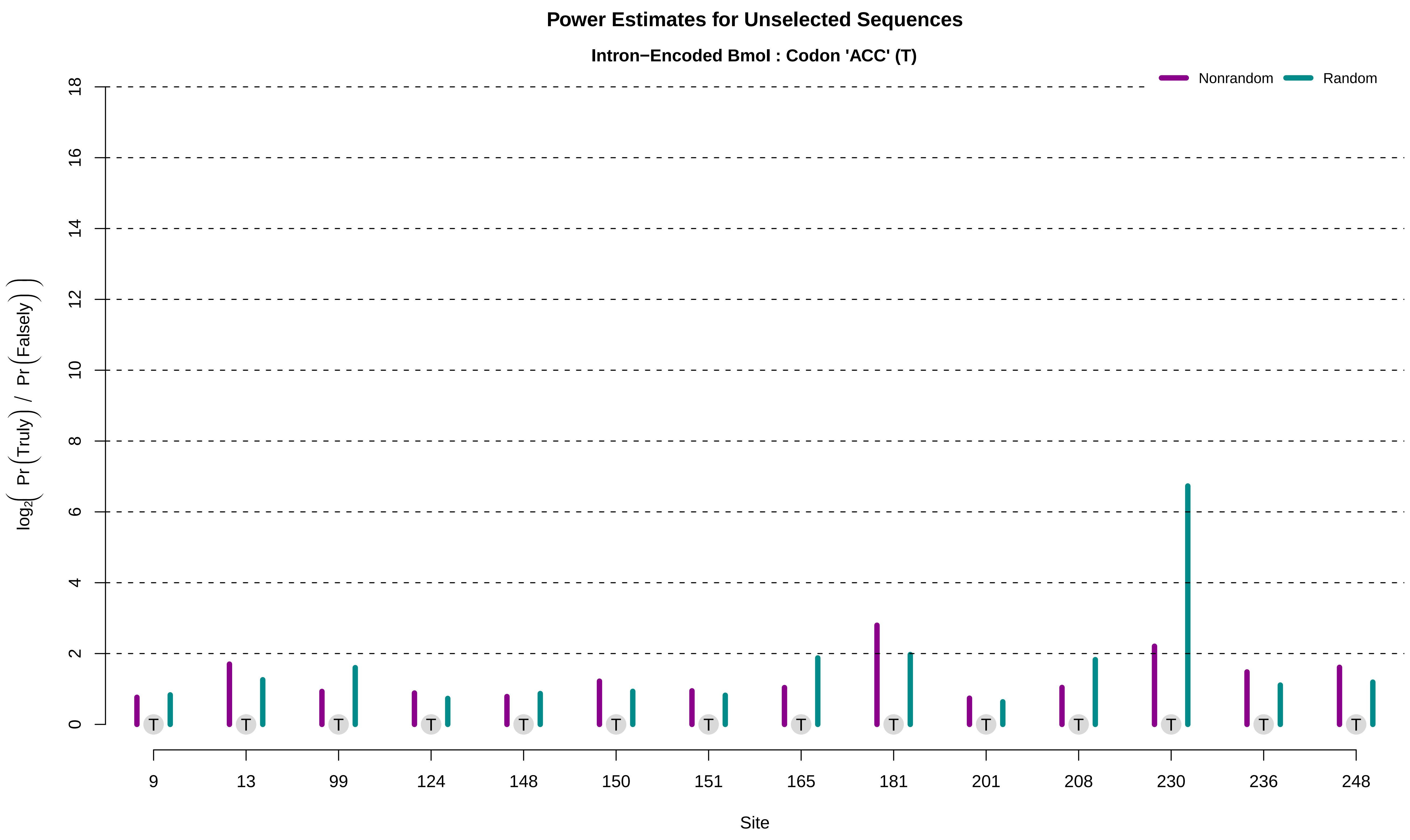
Power Estimates for Unselected Sequences
Intron−Encoded BmoI : Codon 'ACC' (T)
Nonrandom
Random
log2(Pr(Truly) / Pr(Falsely))
0
2
4
6
8
10
12
14
16
18
T
9
13
99
124
148
150
151
165
181
201
208
230
236
248
Site

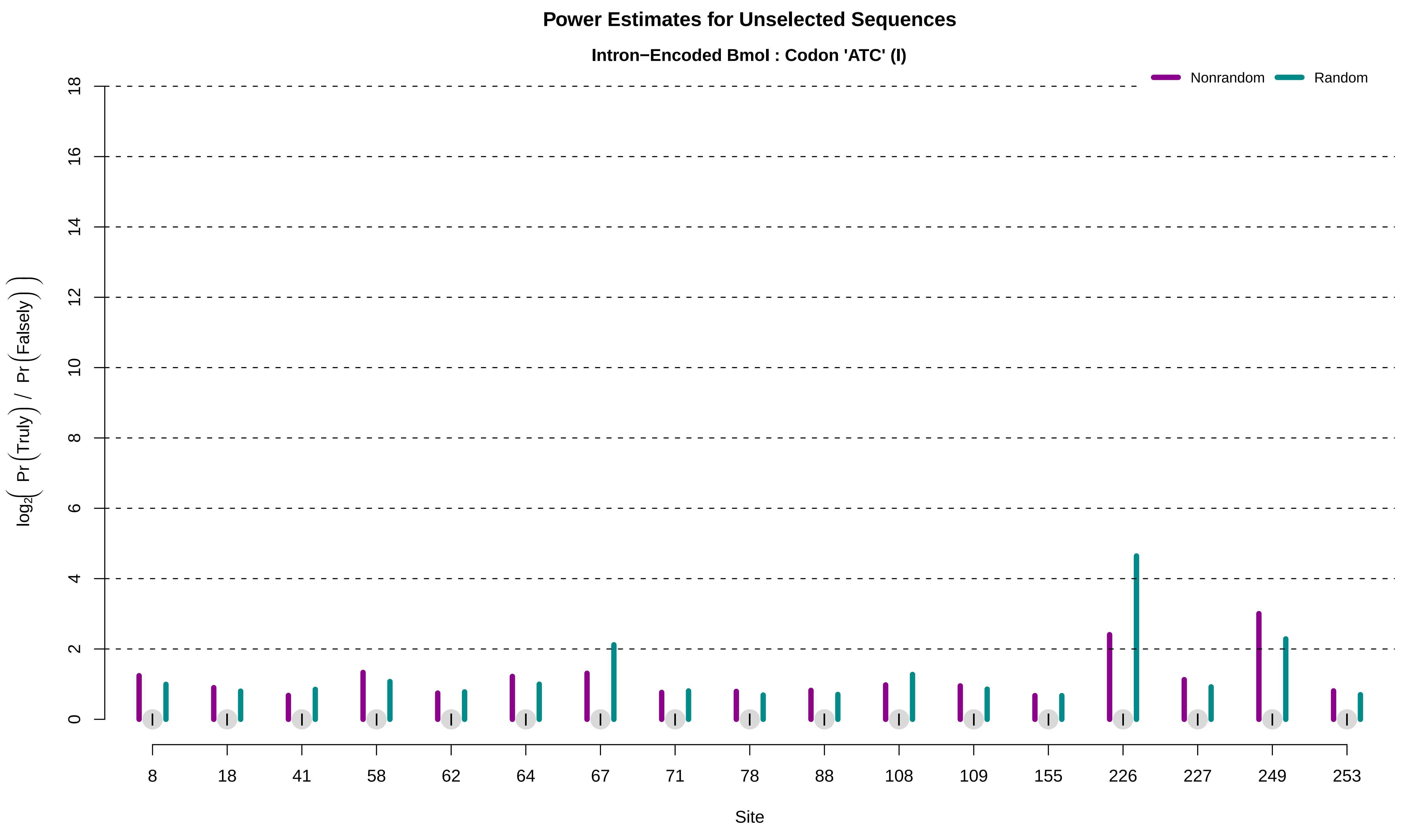

Power Estimates for Unselected Sequences
Intron−Encoded BmoI : Codon 'ATC' (I)
Nonrandom
Random
$\log_2\left(Pr\left(Truly\right) / Pr\left(Falsely\right) \right)$
0
2
4
6
8
10
12
14
16
18
8
18
41
58
62
64
67
71
78
88
108
109
155
226
227
249
253
Site

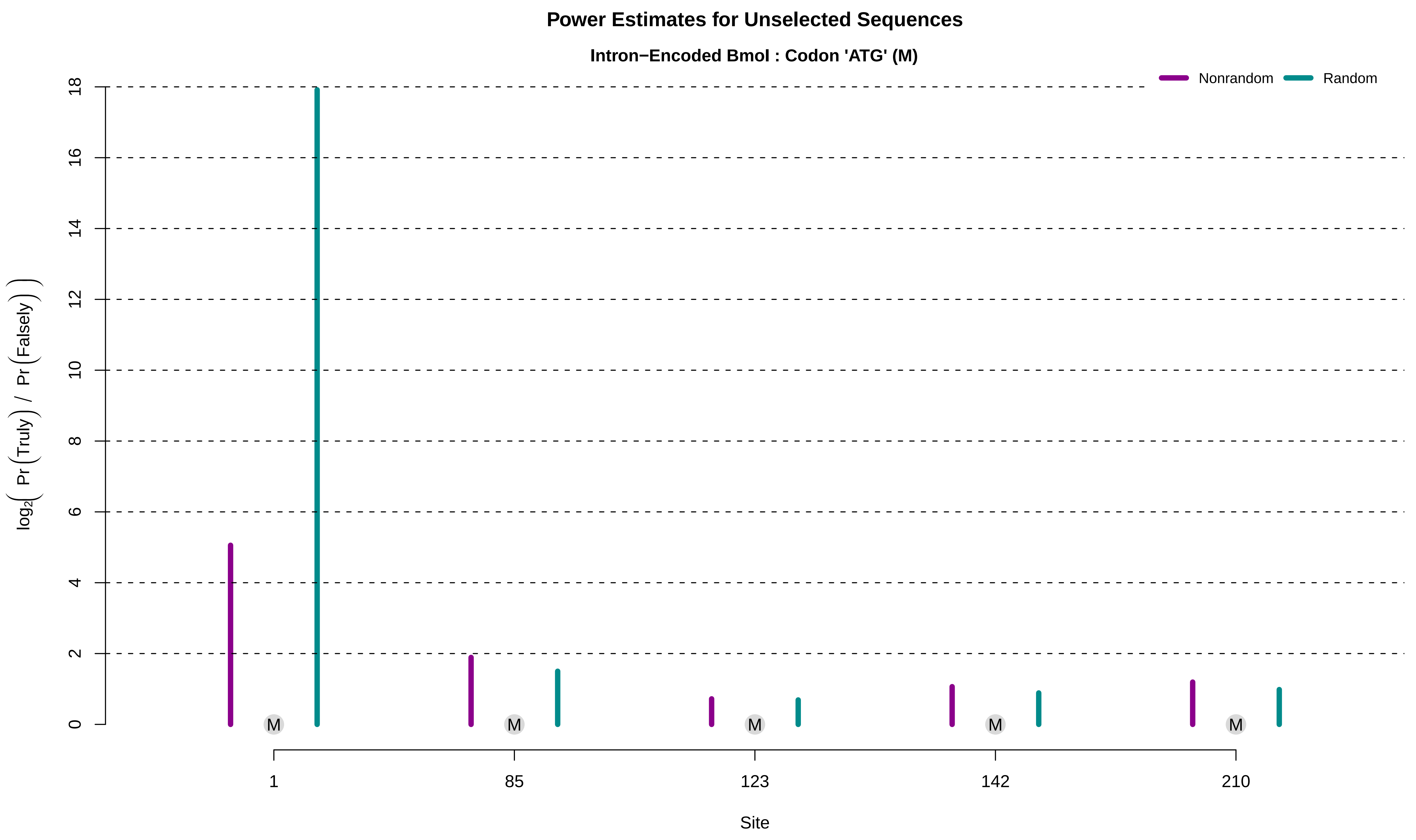
Power Estimates for Unselected Sequences
Intron−Encoded BmoI : Codon 'ATG' (M)
Nonrandom
Random
0
2
4
6
8
10
12
14
16
18
log2(Pr(Truly) / Pr(Falsely))
M
M
M
M
M
1
85
123
142
210
Site

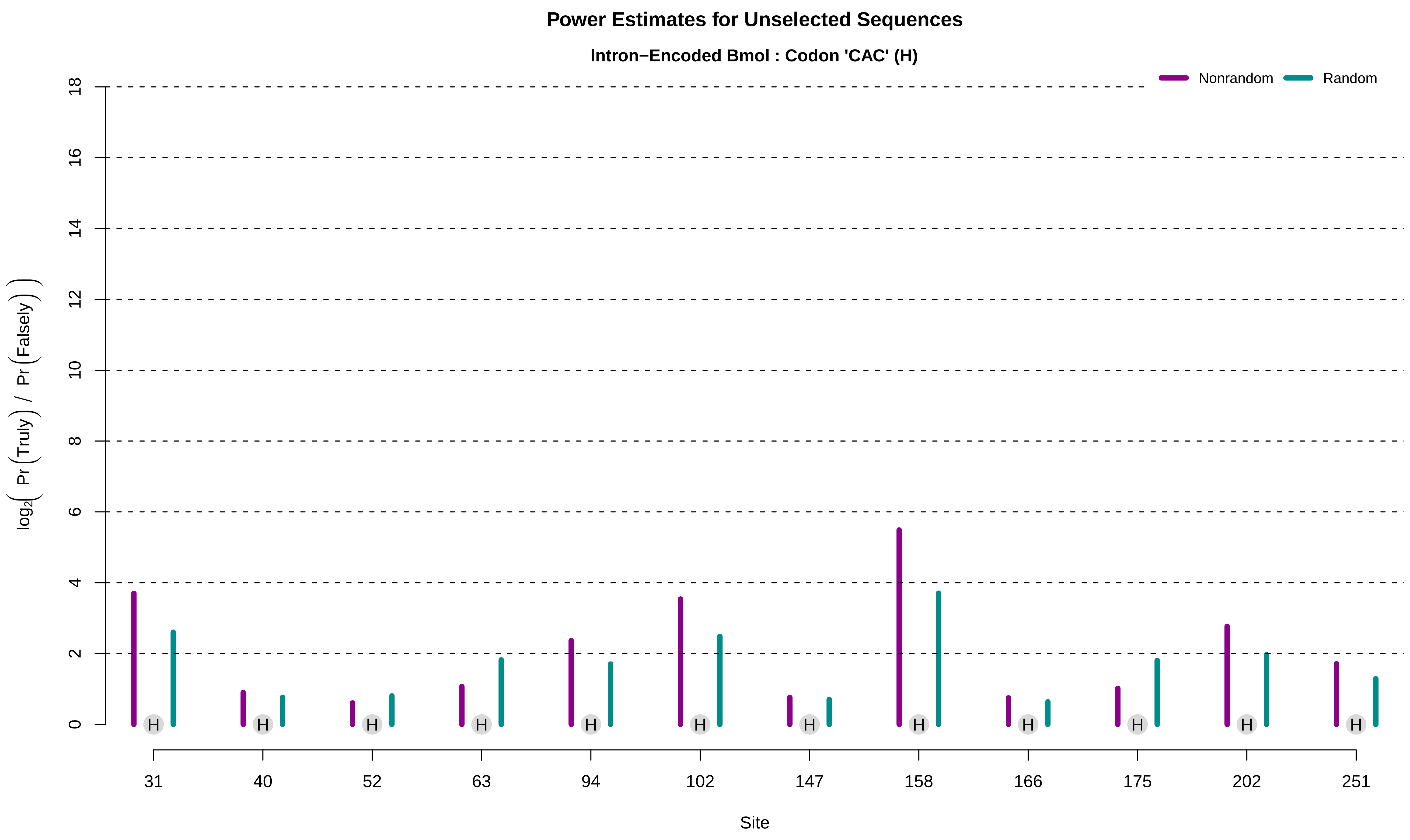

Power Estimates for Unselected Sequences
Intron−Encoded BmoI : Codon 'CAC' (H)
Nonrandom
Random
log2(Pr(Truly) / Pr(Falsely))
0
2
4
6
8
10
12
14
16
18
H
31
40
52
63
94
102
147
158
166
175
202
251
Site

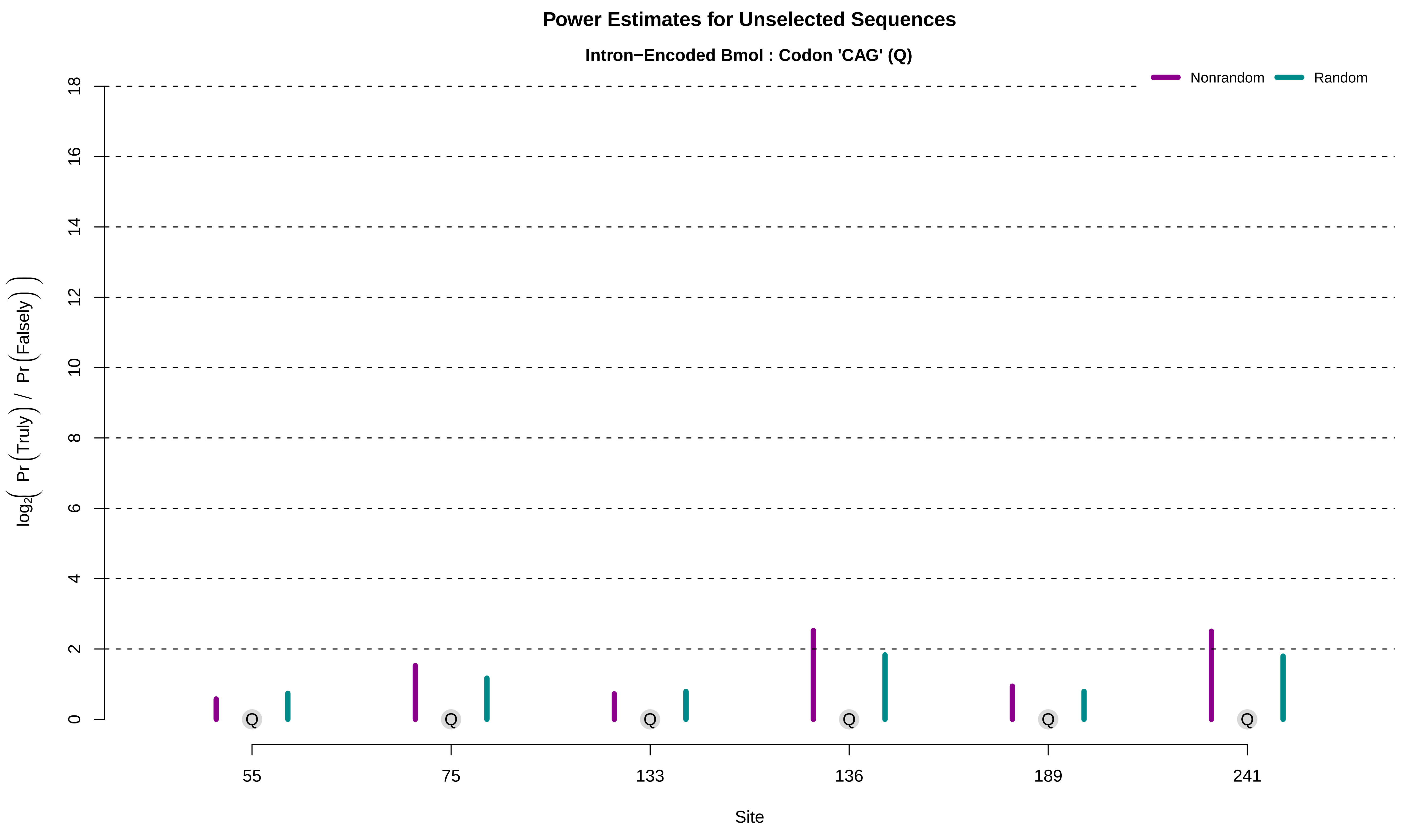

Power Estimates for Unselected Sequences
Intron−Encoded BmoI : Codon 'CAG' (Q)
Nonrandom
Random
0
2
4
6
8
10
12
14
16
18
log2(Pr(Truly) / Pr(Falsely))
Q
Q
Q
Q
Q
Q
55
75
133
136
189
241
Site

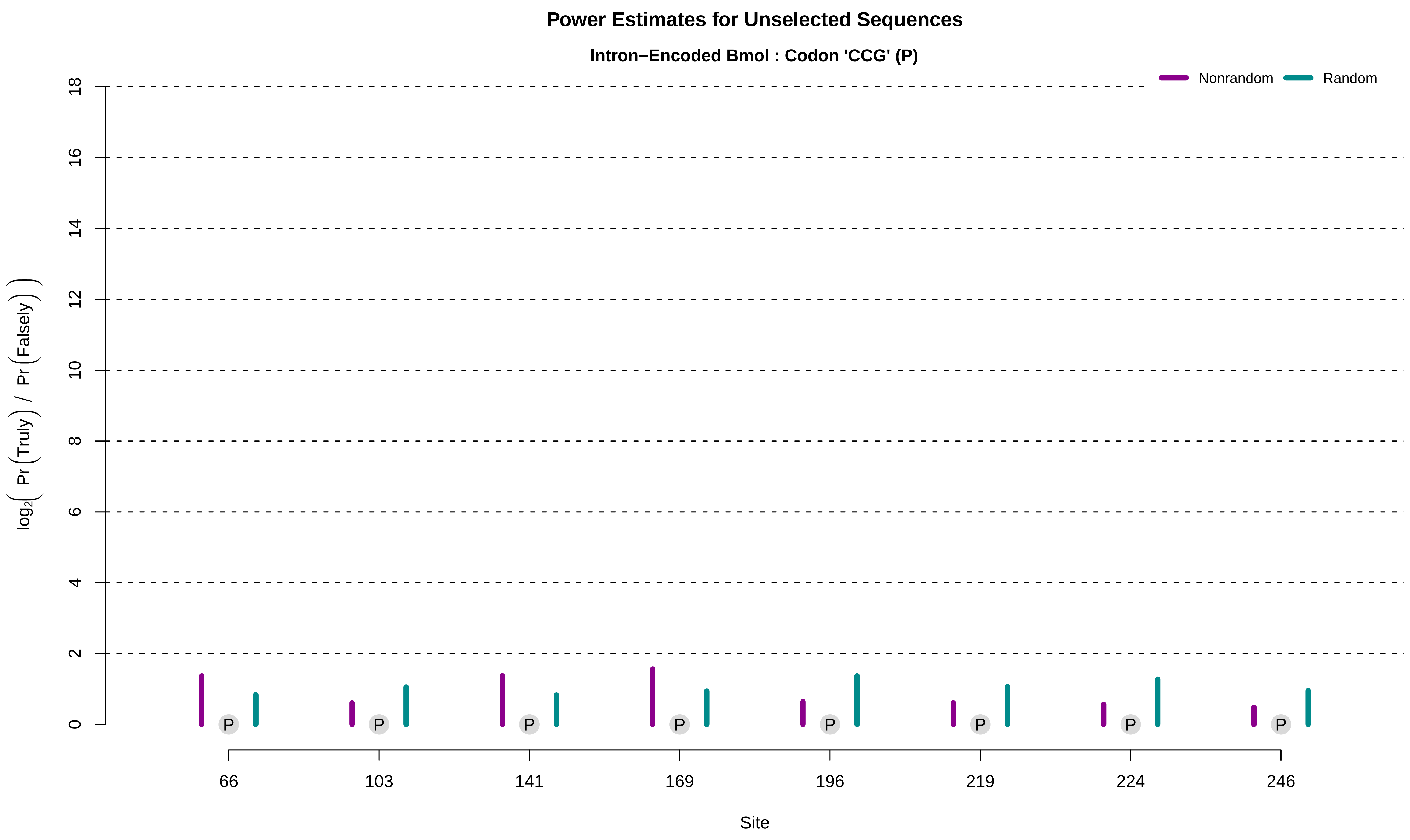
Power Estimates for Unselected Sequences
Intron−Encoded BmoI : Codon 'CCG' (P)
Nonrandom
Random
log2(Pr(Truly) / Pr(Falsely))
0
2
4
6
8
10
12
14
16
18
P
66
103
141
169
196
219
224
246
Site

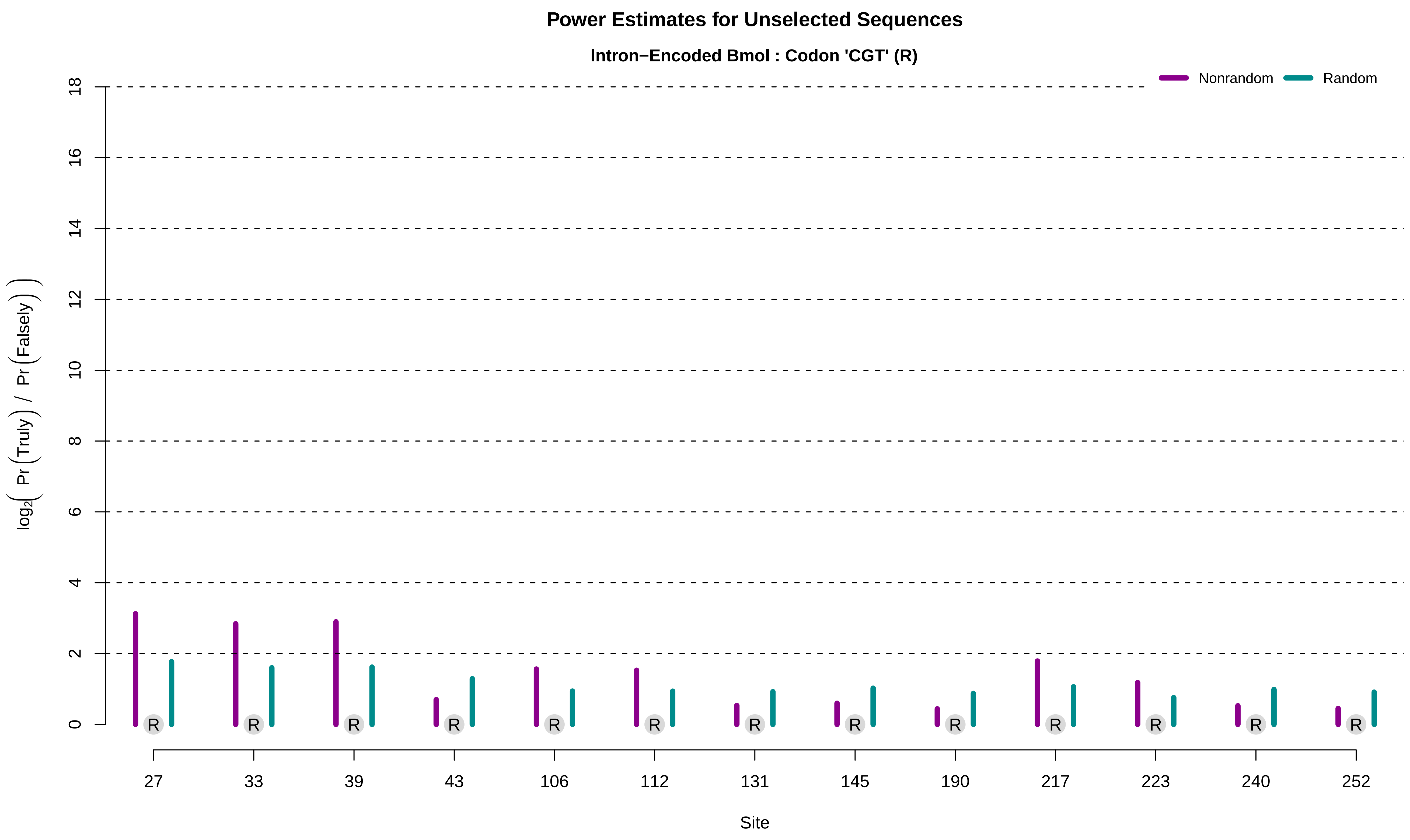

Power Estimates for Unselected Sequences
Intron−Encoded BmoI : Codon 'CGT' (R)
Nonrandom
Random
log2(Pr(Truly) / Pr(Falsely))
0
2
4
6
8
10
12
14
16
18
R
27
33
39
43
106
112
131
145
190
217
223
240
252
Site

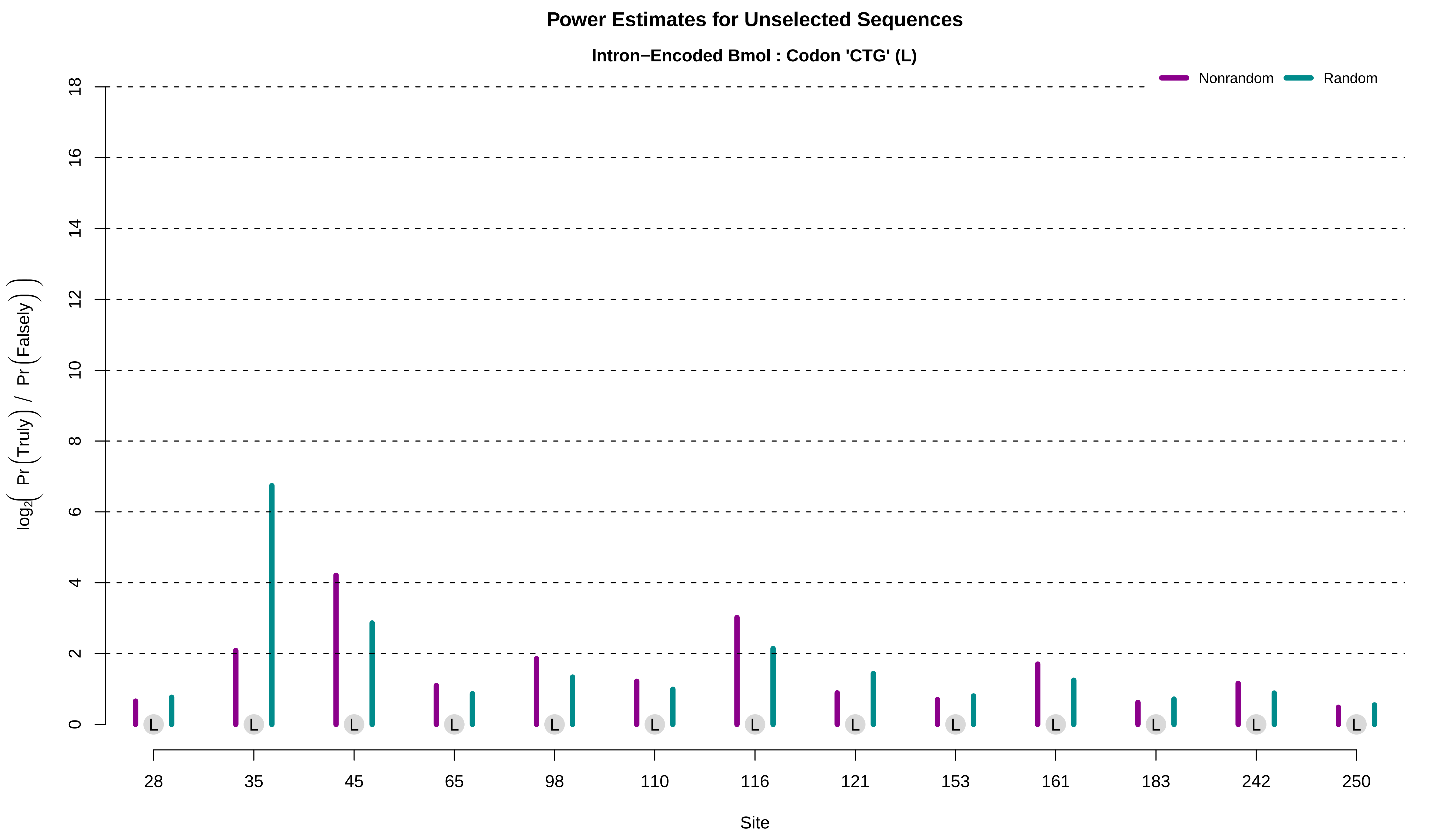

Power Estimates for Unselected Sequences
Intron−Encoded BmoI : Codon 'CTG' (L)
Nonrandom
Random
log2(Pr(Truly) / Pr(Falsely))
0
2
4
6
8
10
12
14
16
18
L
28
35
45
65
98
110
116
121
153
161
183
242
250
Site

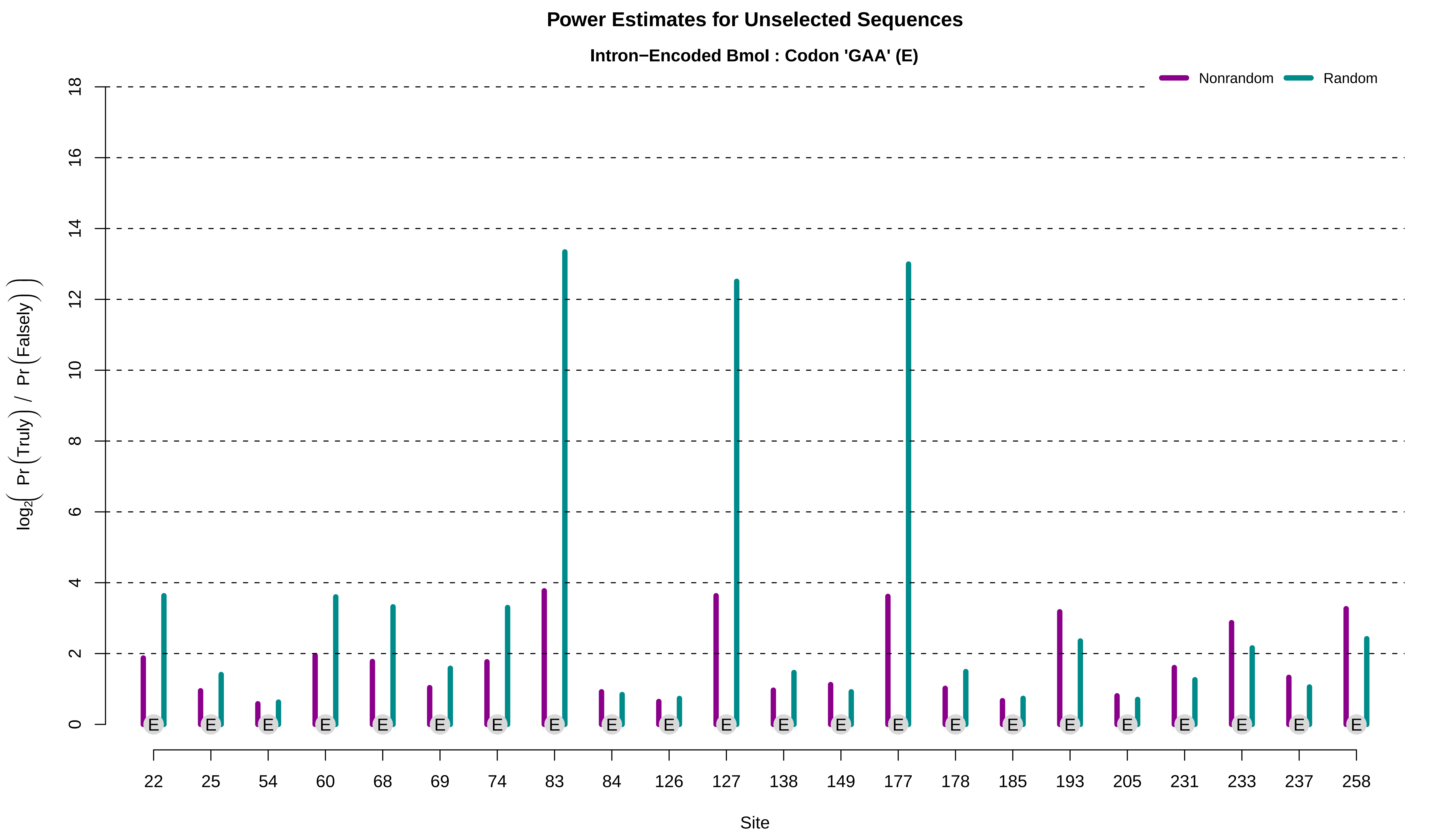

Power Estimates for Unselected Sequences
Intron−Encoded BmoI : Codon 'GAA' (E)
Nonrandom
Random
0
2
4
6
8
10
12
14
16
18
log2(Pr(Truly) / Pr(Falsely))
E
22
25
54
60
68
69
74
83
84
126
127
138
149
177
178
185
193
205
231
233
237
258
Site

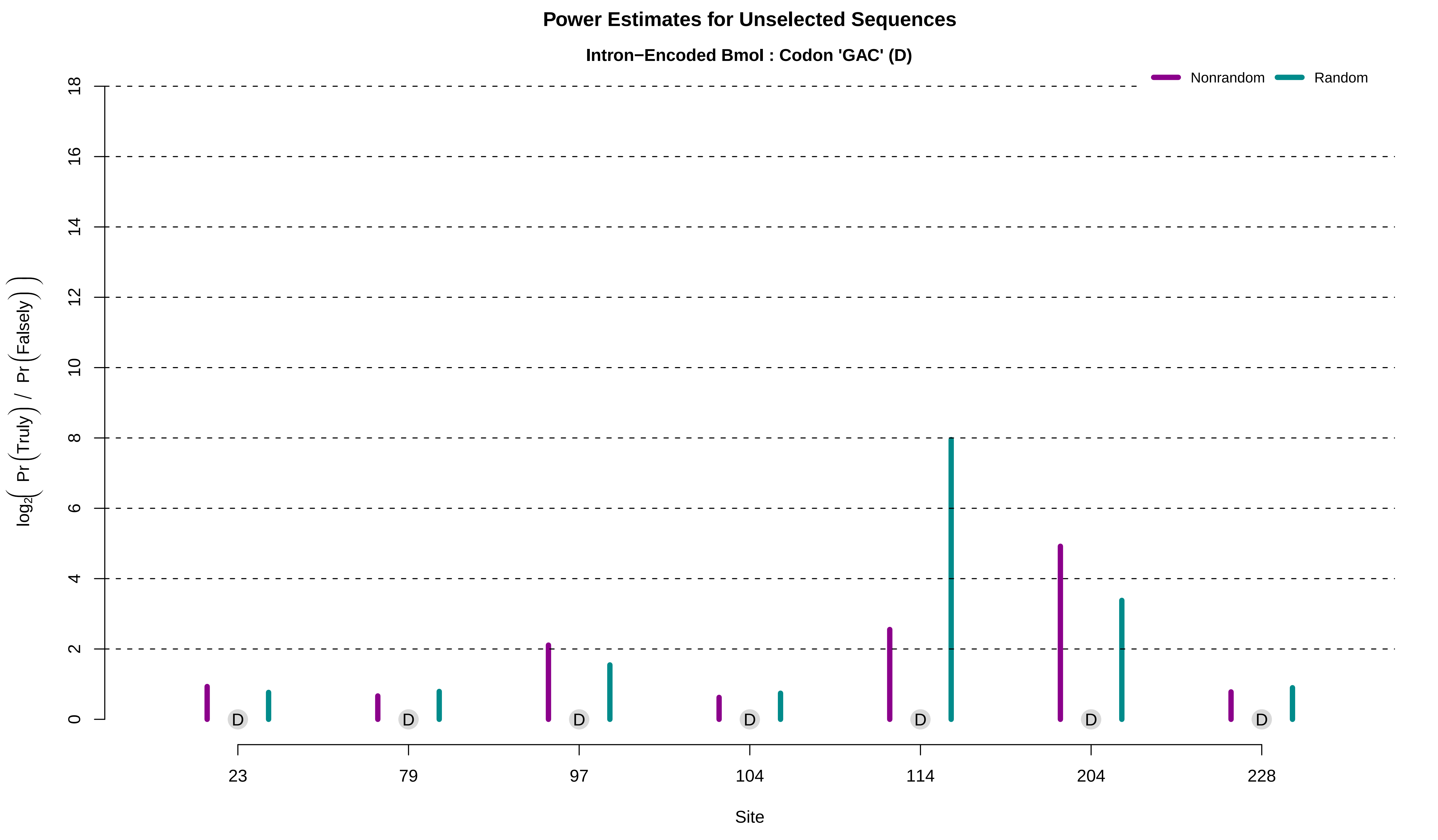

Power Estimates for Unselected Sequences
Intron−Encoded BmoI : Codon 'GAC' (D)
Nonrandom
Random
log2(Pr(Truly) / Pr(Falsely))
0
2
4
6
8
10
12
14
16
18
D
23
79
97
104
114
204
228
Site

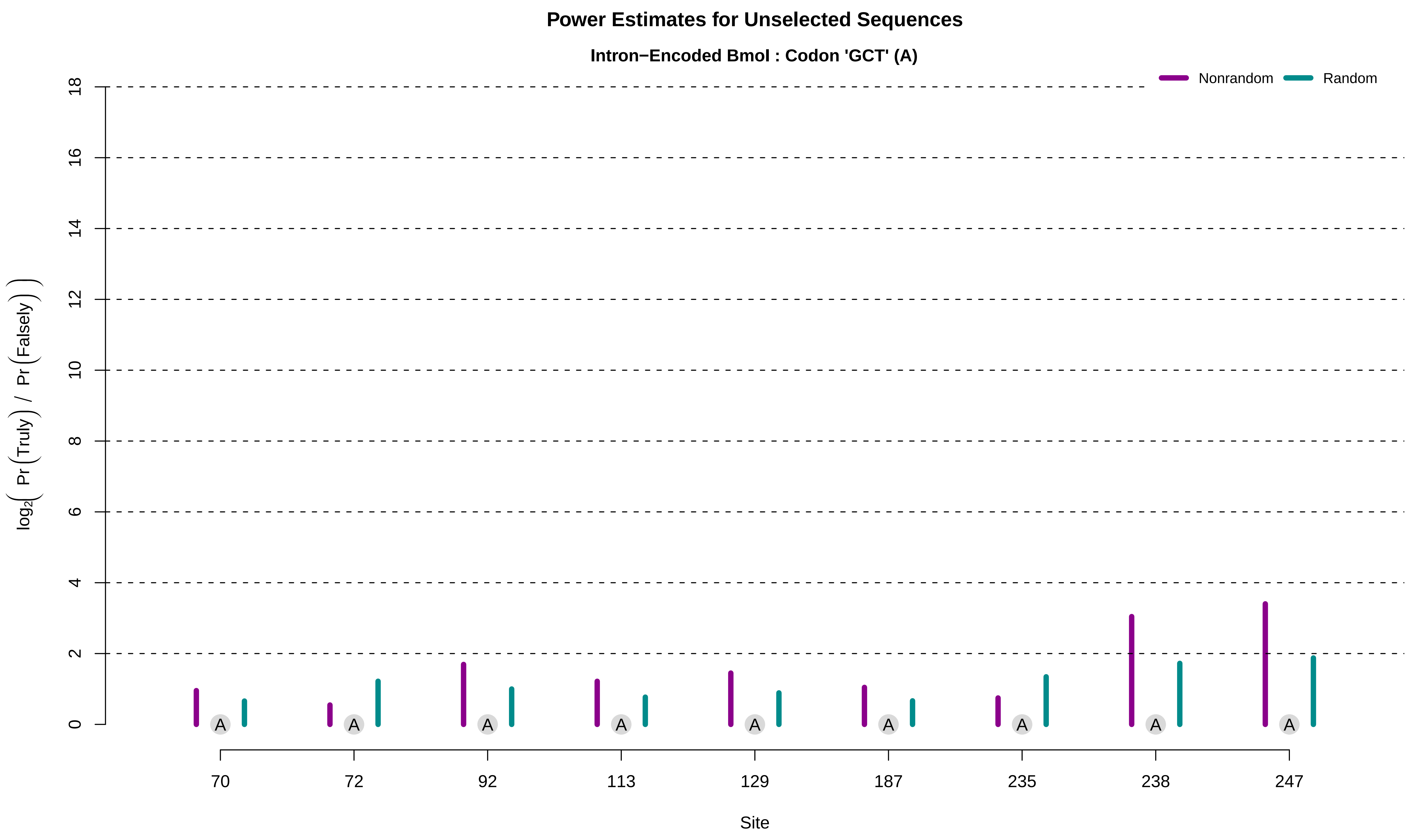

Power Estimates for Unselected Sequences
Intron−Encoded BmoI : Codon 'GCT' (A)
Nonrandom
Random
log2(Pr(Truly) / Pr(Falsely))
0
2
4
6
8
10
12
14
16
18
A
70
72
92
113
129
187
235
238
247
Site

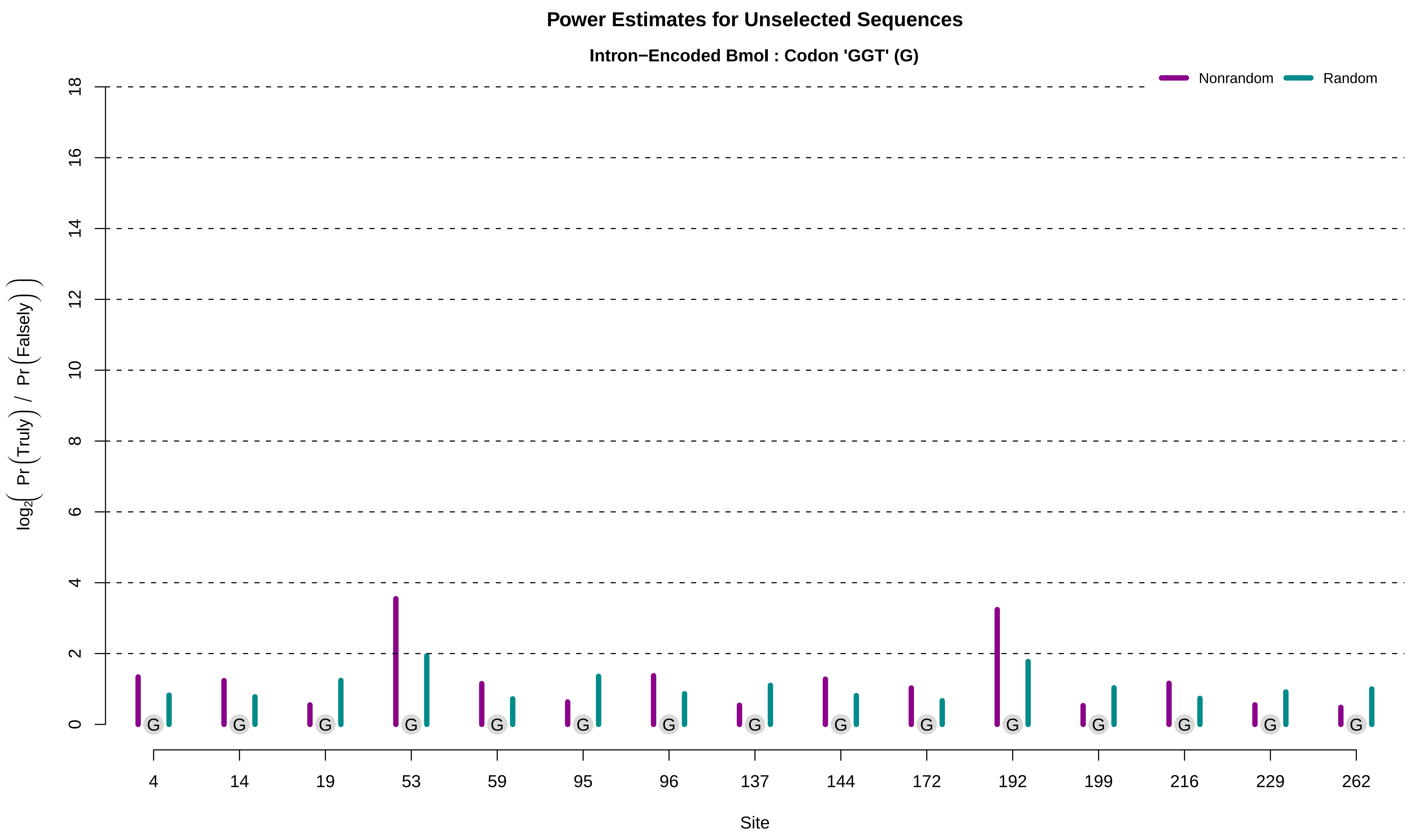

Power Estimates for Unselected Sequences
Intron−Encoded BmoI : Codon 'GGT' (G)
Nonrandom
Random
log2(Pr(Truly) / Pr(Falsely))
0
2
4
6
8
10
12
14
16
18
G
4
14
19
53
59
95
96
137
144
172
192
199
216
229
262
Site

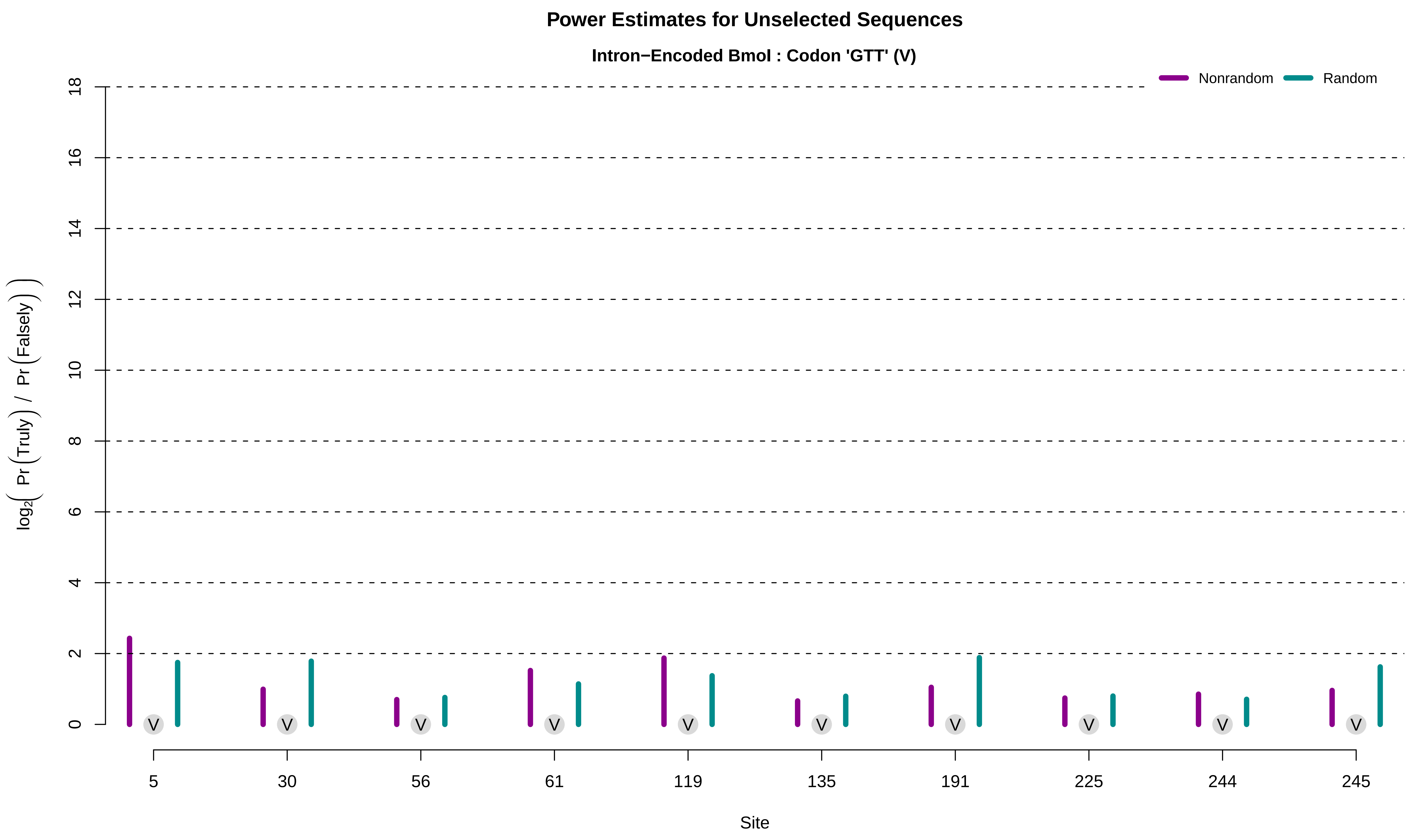

Power Estimates for Unselected Sequences
Intron−Encoded BmoI : Codon 'GTT' (V)
Nonrandom
Random
log2(Pr(Truly) / Pr(Falsely))
0
2
4
6
8
10
12
14
16
18
V
5
30
56
61
119
135
191
225
244
245
Site

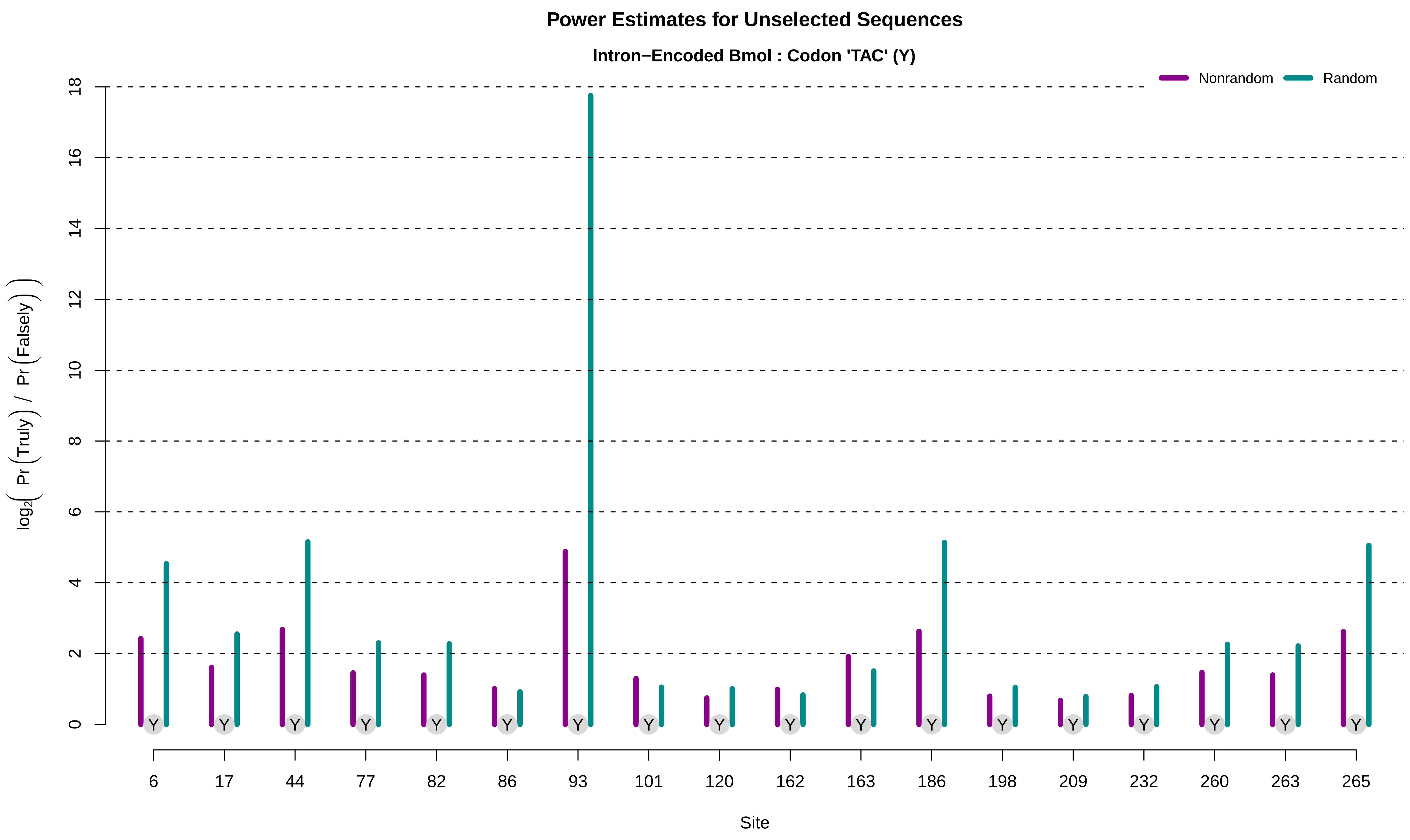

Power Estimates for Unselected Sequences
Intron−Encoded BmoI : Codon 'TAC' (Y)
Nonrandom
Random
log2(Pr(Truly) / Pr(Falsely))
0
2
4
6
8
10
12
14
16
18
Y
6
17
44
77
82
86
93
101
120
162
163
186
198
209
232
260
263
265
Site

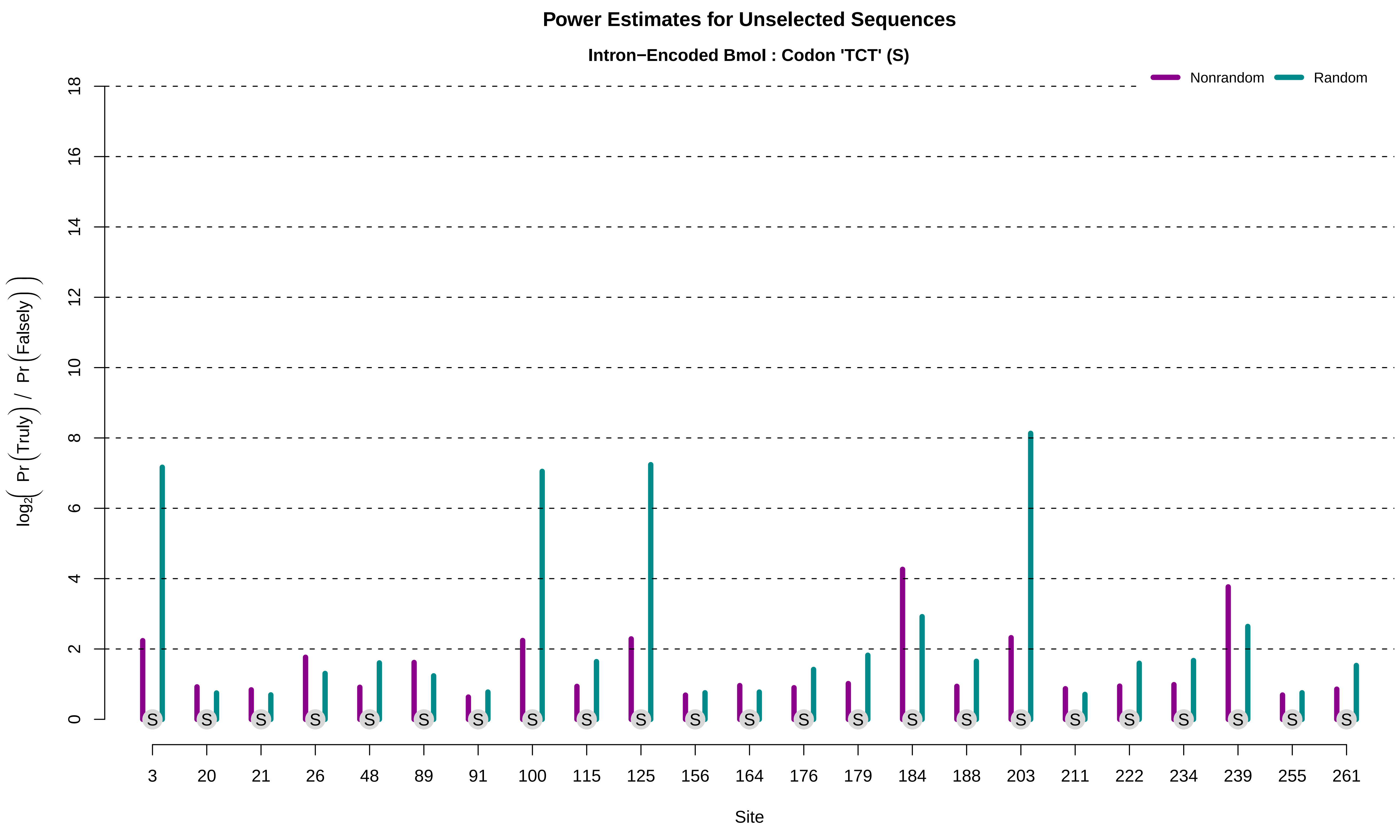

Power Estimates for Unselected Sequences
Intron−Encoded BmoI : Codon 'TCT' (S)
Nonrandom
Random
log2(Pr(Truly) / Pr(Falsely))
0
2
4
6
8
10
12
14
16
18
S
3
20
21
26
48
89
91
100
115
125
156
164
176
179
184
188
203
211
222
234
239
255
261
Site

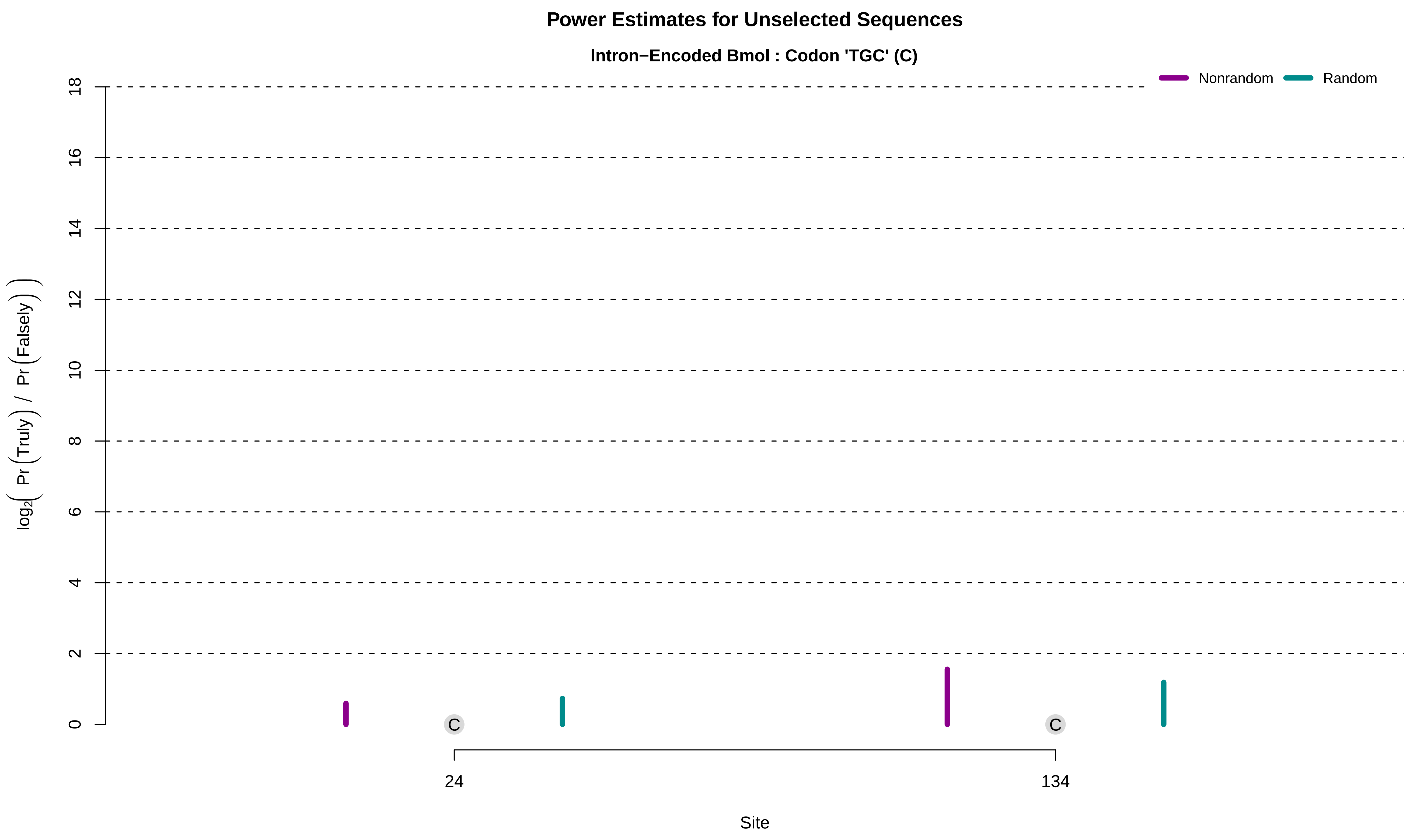
Power Estimates for Unselected Sequences
Intron−Encoded BmoI : Codon 'TGC' (C)
Nonrandom
Random
0
2
4
6
8
10
12
14
16
18
log2(Pr(Truly) / Pr(Falsely))
C
C
24
134
Site

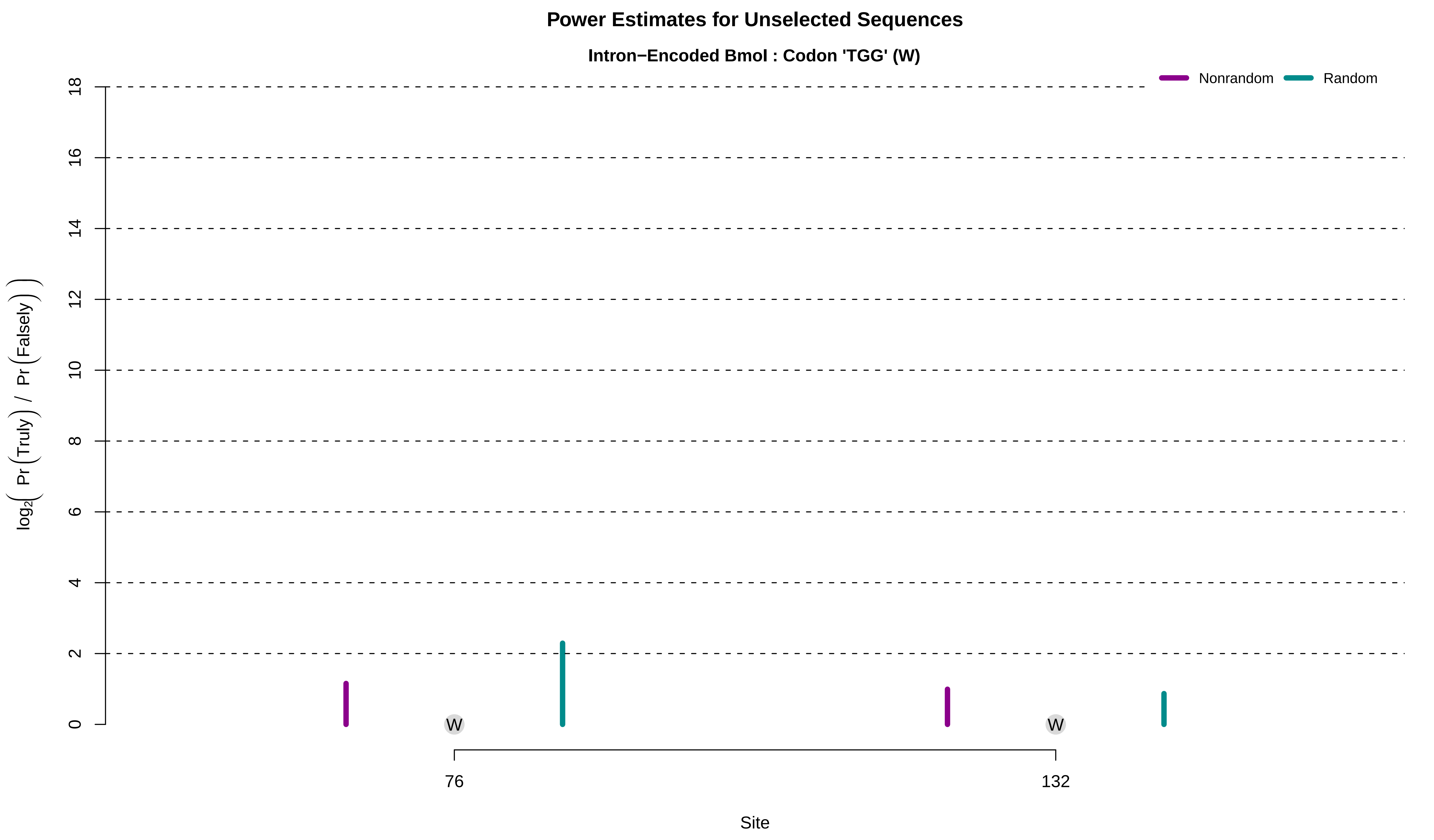
Power Estimates for Unselected Sequences
Intron−Encoded BmoI : Codon 'TGG' (W)
Nonrandom
Random
0
2
4
6
8
10
12
14
16
18
log2(Pr(Truly) / Pr(Falsely))
W
W
76
132
Site

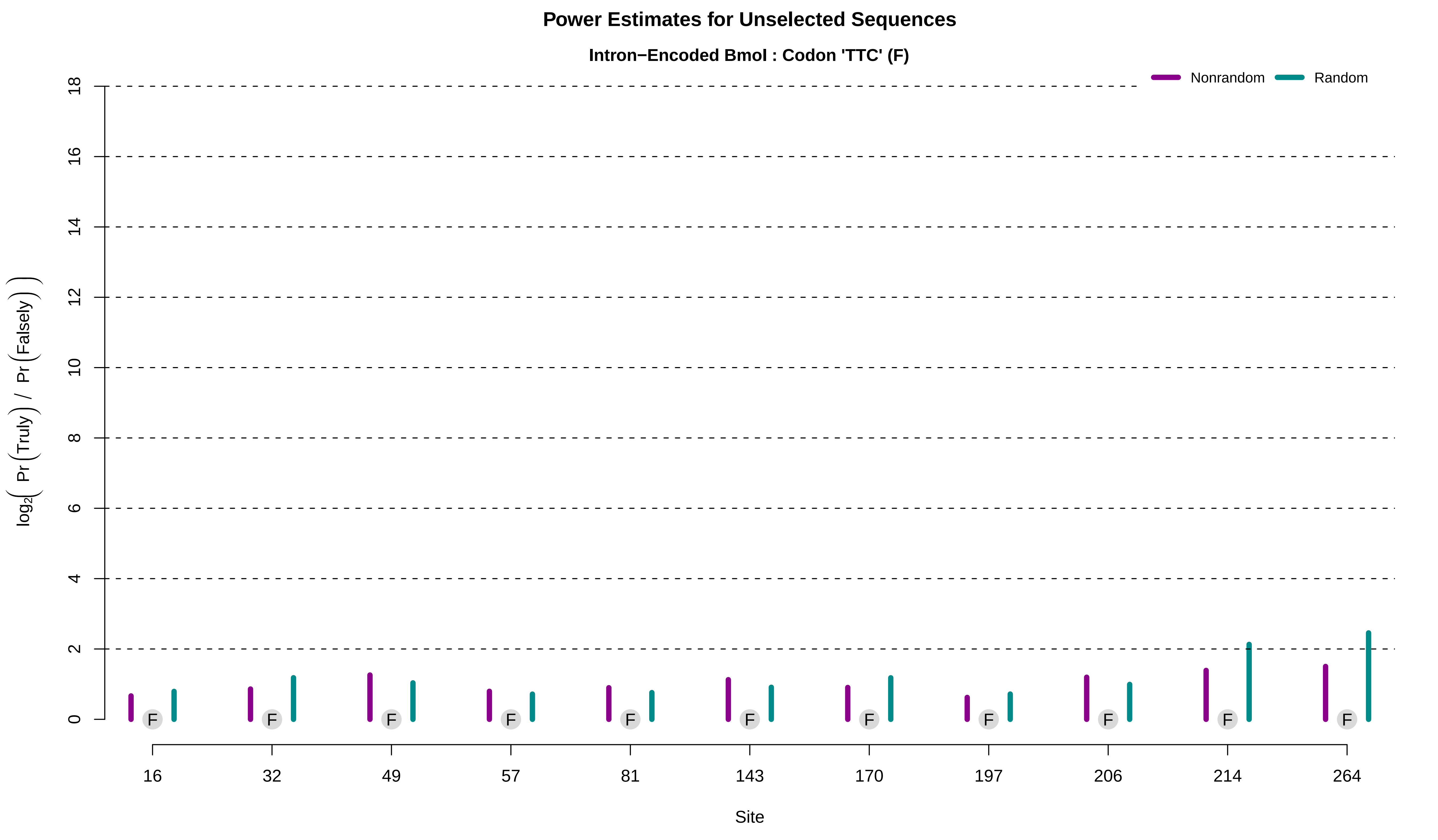

Power Estimates for Unselected Sequences
Intron−Encoded BmoI : Codon 'TTC' (F)
Nonrandom
Random
$\log_2\left(\Pr\left(\text{Truly}\right) / \Pr\left(\text{Falsely}\right) \right)$
0
2
4
6
8
10
12
14
16
18
F
16
32
49
57
81
143
170
197
206
214
264
Site
