# Supplementary material for: Estimating the evidence of selection and the reliability of inference in unigenic evolution
Source: Algorithms Mol Biol. 2010 Nov 8;5:35. doi: 10.1186/1748-7188-5-35 (PMC2994857; doi:10.1186/1748-7188-5-35)
Supplement: Additional file 7 — Sample Input and Output. Sample input, output, and driver files for the given software package. [file 1748-7188-5-35-S7.ZIP › unigenic_example/edgell.pol/by_site_ech.pdf]

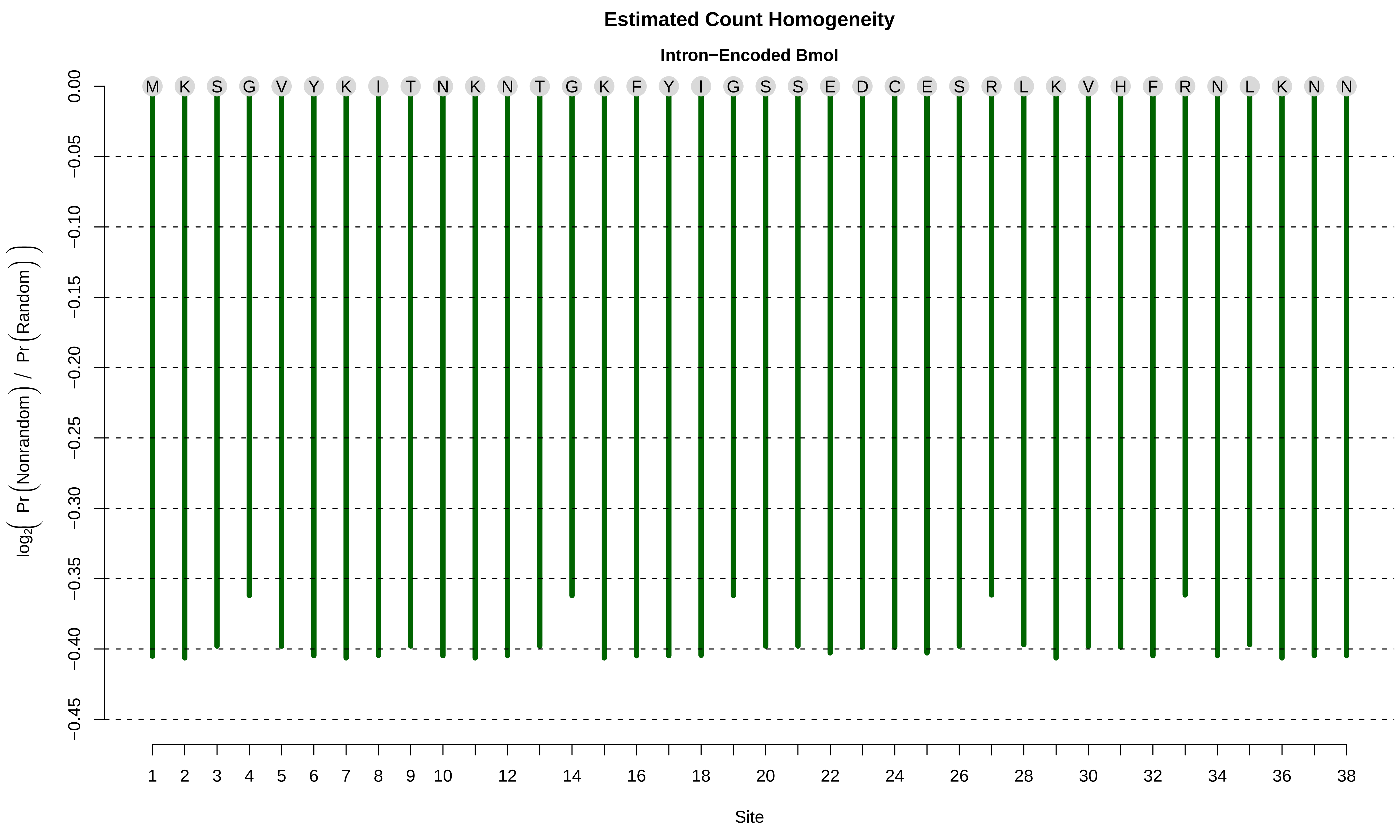
Estimated Count Homogeneity
Intron−Encoded BmoI
M K S G V Y K I T N K N T G K F Y I G S S E D C E S R L K V H F R N L K N N
0.00
−0.05
−0.10
−0.15
−0.20
−0.25
−0.30
−0.35
−0.40
−0.45
log2(Pr(Nonrandom) / Pr(Random))
1 2 3 4 5 6 7 8 9 10 12 14 16 18 20 22 24 26 28 30 32 34 36 38
Site

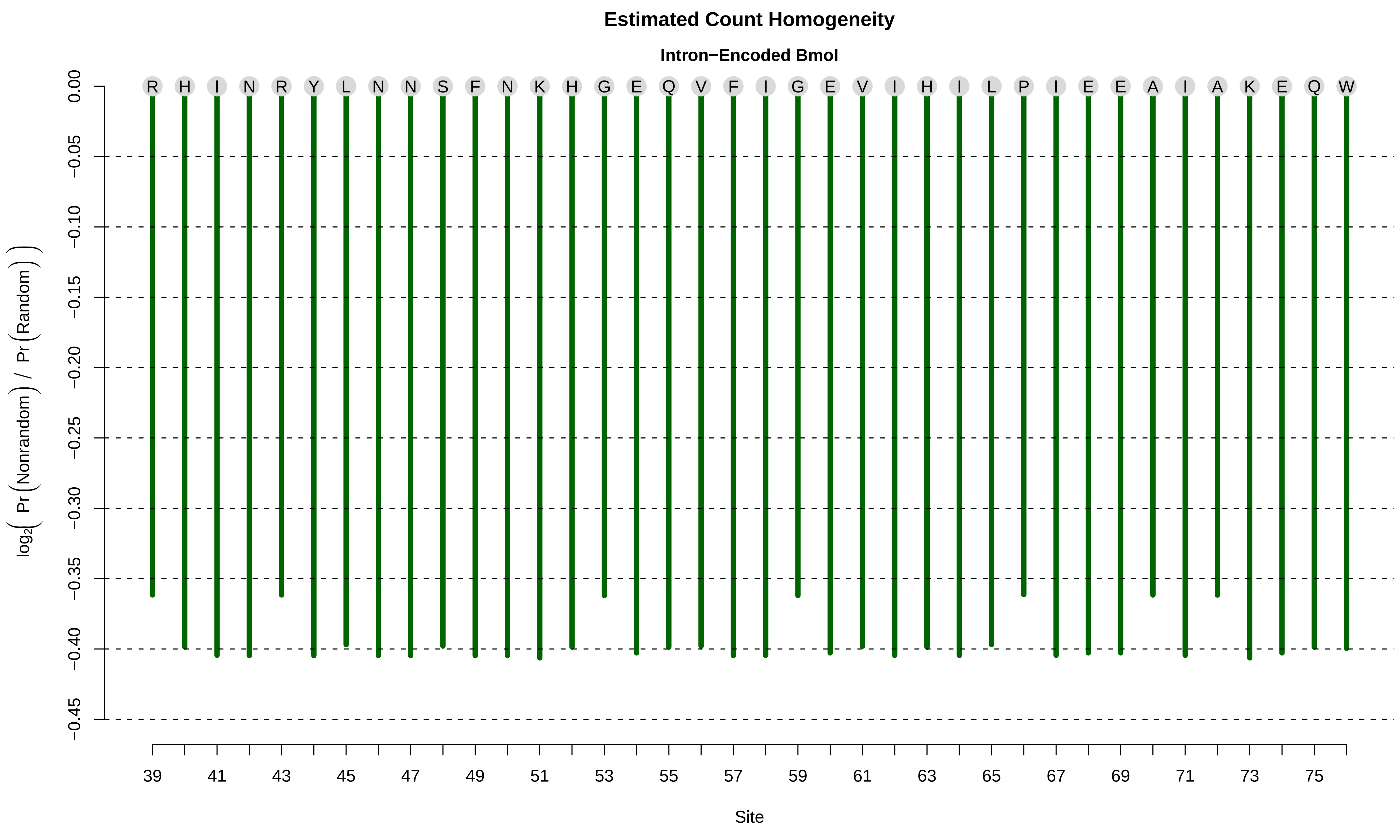
Estimated Count Homogeneity
Intron−Encoded BmoI
R H I N R Y L N N S F N K H G E Q V F I G E V I H I L P I E E A I A K E Q W
0.00
−0.05
−0.10
−0.15
−0.20
−0.25
−0.30
−0.35
−0.40
−0.45
39 41 43 45 47 49 51 53 55 57 59 61 63 65 67 69 71 73 75
Site
log2(Pr(Nonrandom) / Pr(Random))

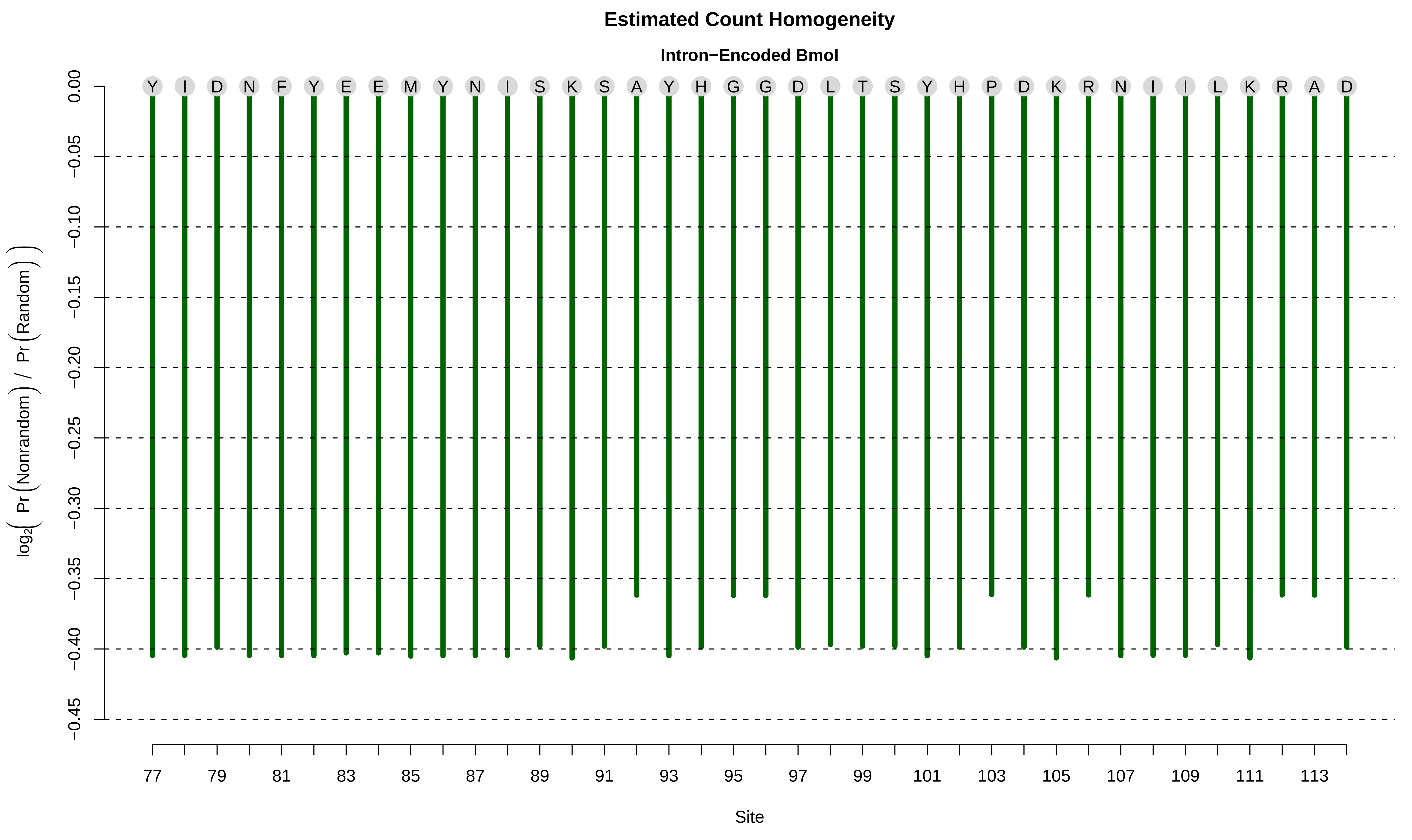

Estimated Count Homogeneity
Intron−Encoded BmoI
log2(Pr(Nonrandom) / Pr(Random))
0.00
−0.05
−0.10
−0.15
−0.20
−0.25
−0.30
−0.35
−0.40
−0.45
Y I D N F Y E E M Y N I S K S A Y H G G D L T S Y H P D K R N I I L K R A D
77 79 81 83 85 87 89 91 93 95 97 99 101 103 105 107 109 111 113
Site

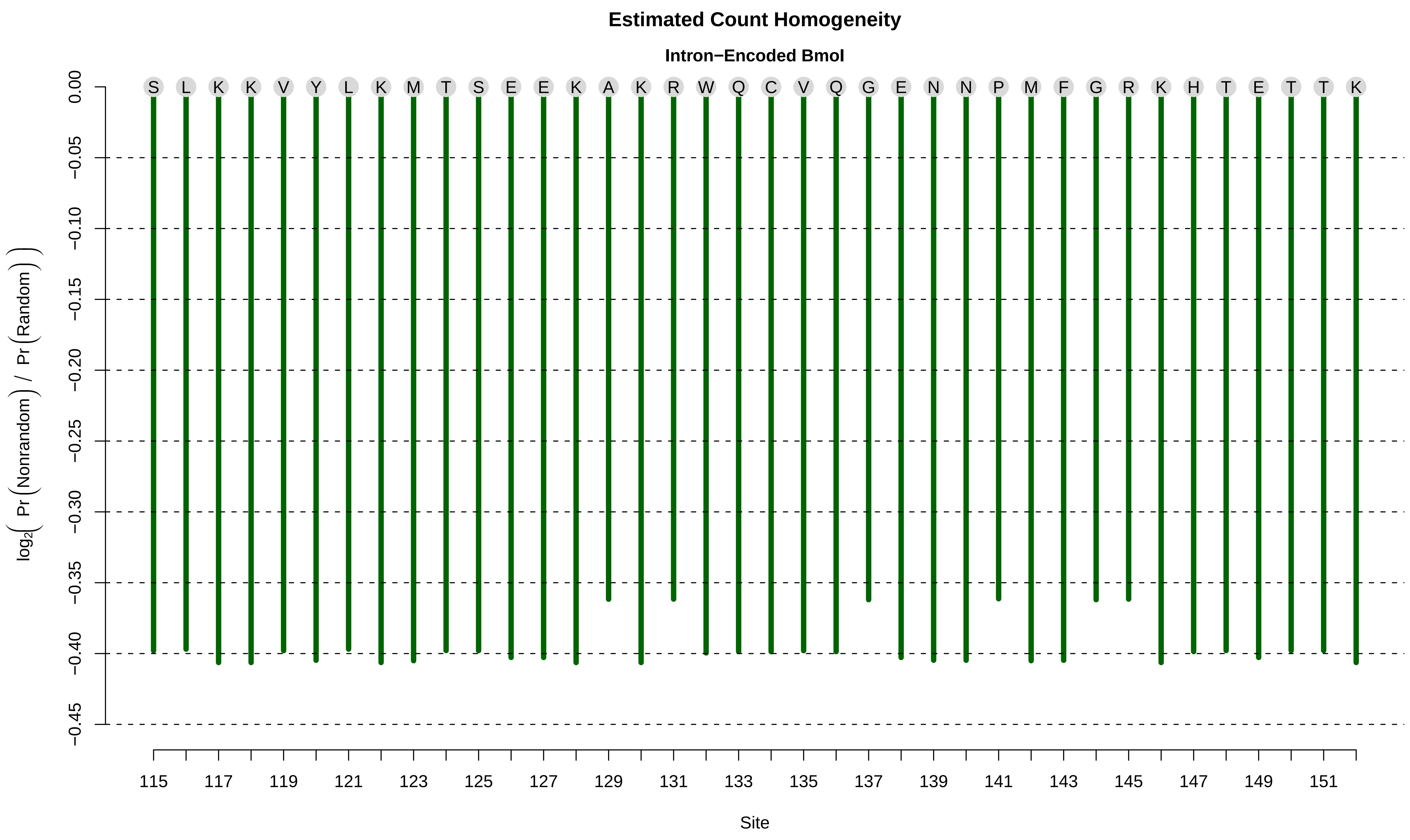
Estimated Count Homogeneity
Intron−Encoded BmoI
S L K K V Y L K M T S E E K A K R W Q C V Q G E N N P M F G R K H T E T T K
0.00
−0.05
−0.10
−0.15
−0.20
−0.25
−0.30
−0.35
−0.40
−0.45
log2(Pr(Nonrandom) / Pr(Random))
115
117
119
121
123
125
127
129
131
133
135
137
139
141
143
145
147
149
151
Site

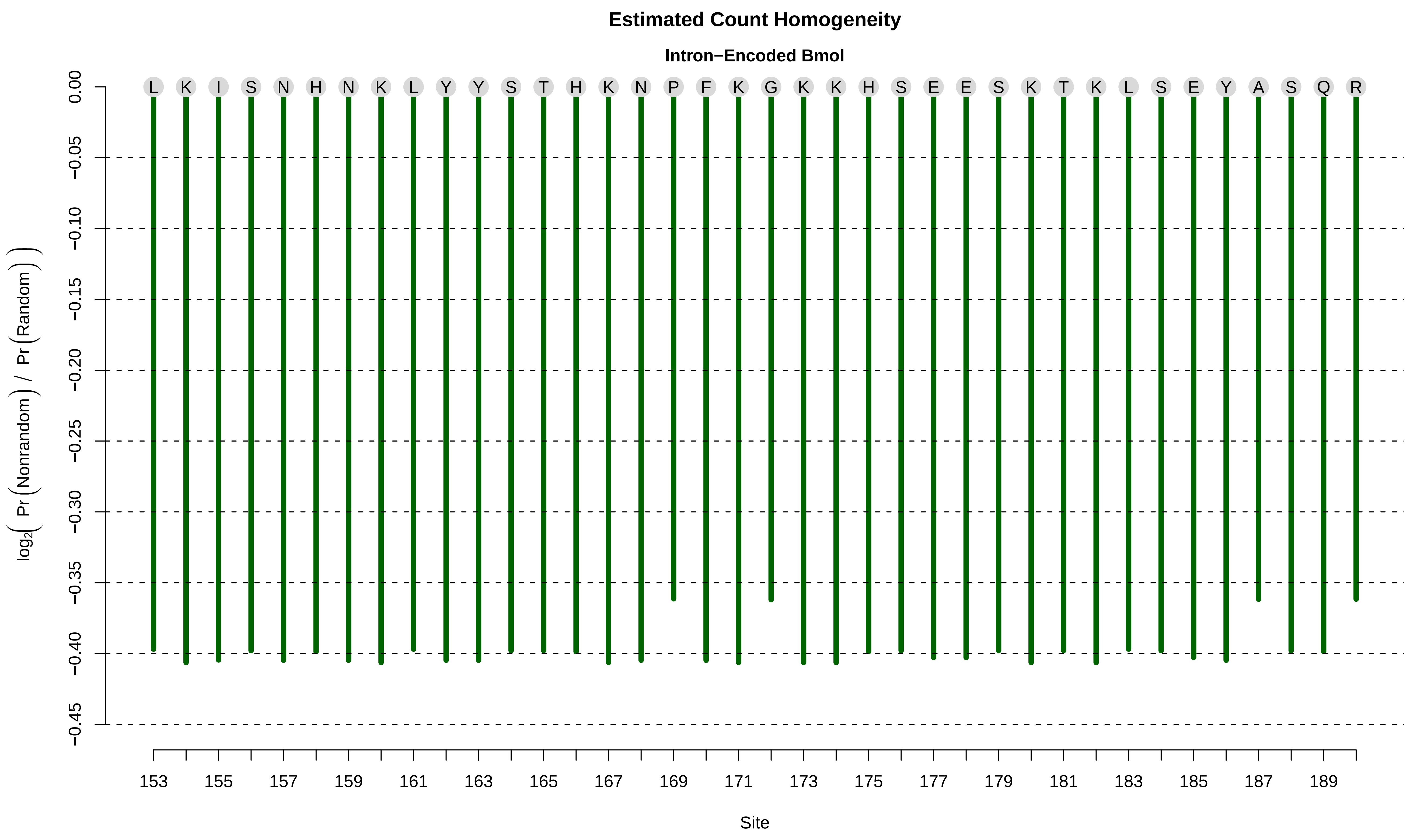
Estimated Count Homogeneity
Intron−Encoded BmoI
log2(Pr(Nonrandom) / Pr(Random))
0.00
−0.05
−0.10
−0.15
−0.20
−0.25
−0.30
−0.35
−0.40
−0.45
L K I S N H N K L Y Y S T H K N P F K G K K H S E E S K T K L S E Y A S Q R
153
155
157
159
161
163
165
167
169
171
173
175
177
179
181
183
185
187
189
Site

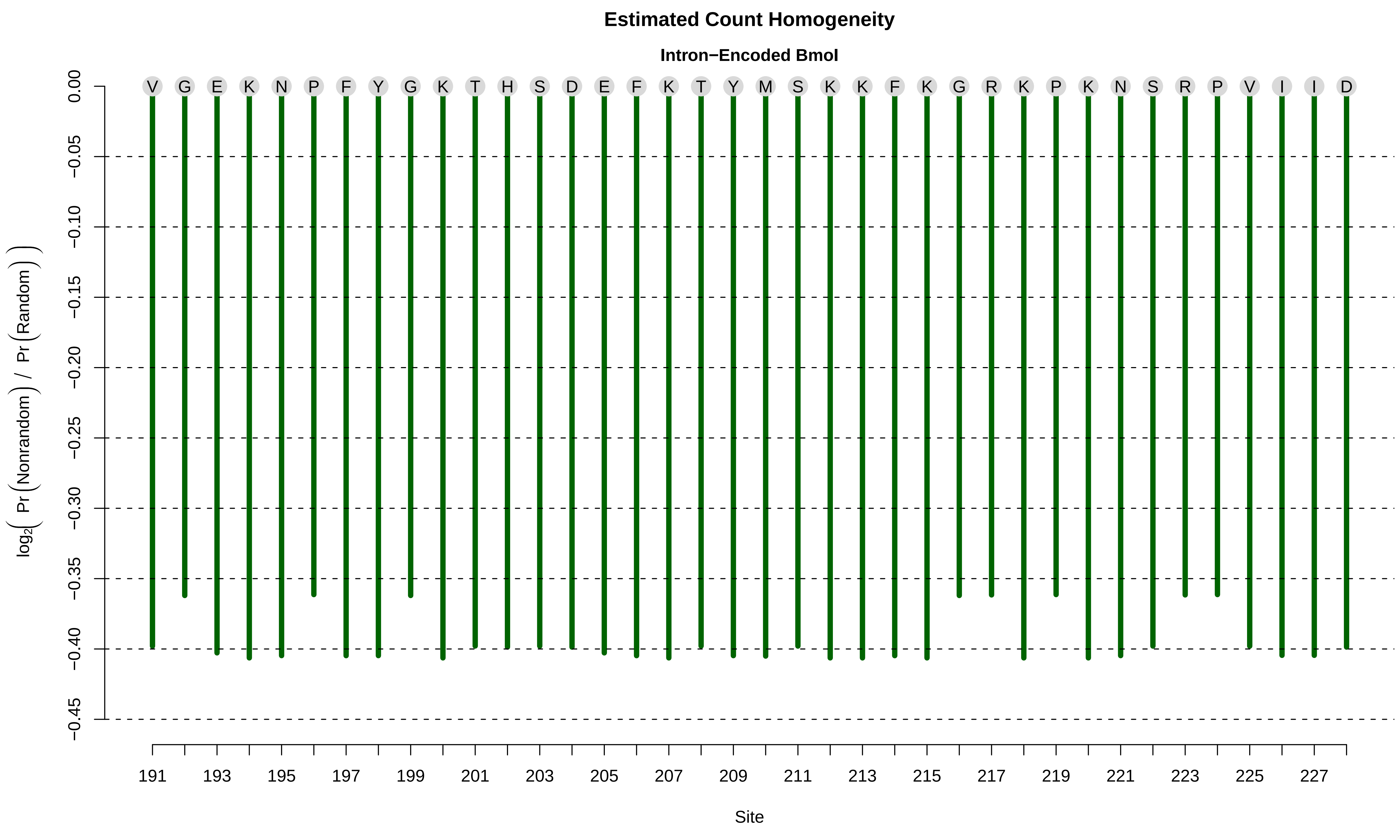
Estimated Count Homogeneity
Intron−Encoded BmoI
log2(Pr(Nonrandom) / Pr(Random))
0.00
−0.05
−0.10
−0.15
−0.20
−0.25
−0.30
−0.35
−0.40
−0.45
V G E K N P F Y G K T H S D E F K T Y M S K K F K G R K P K N S R P V I I D
191 193 195 197 199 201 203 205 207 209 211 213 215 217 219 221 223 225 227
Site

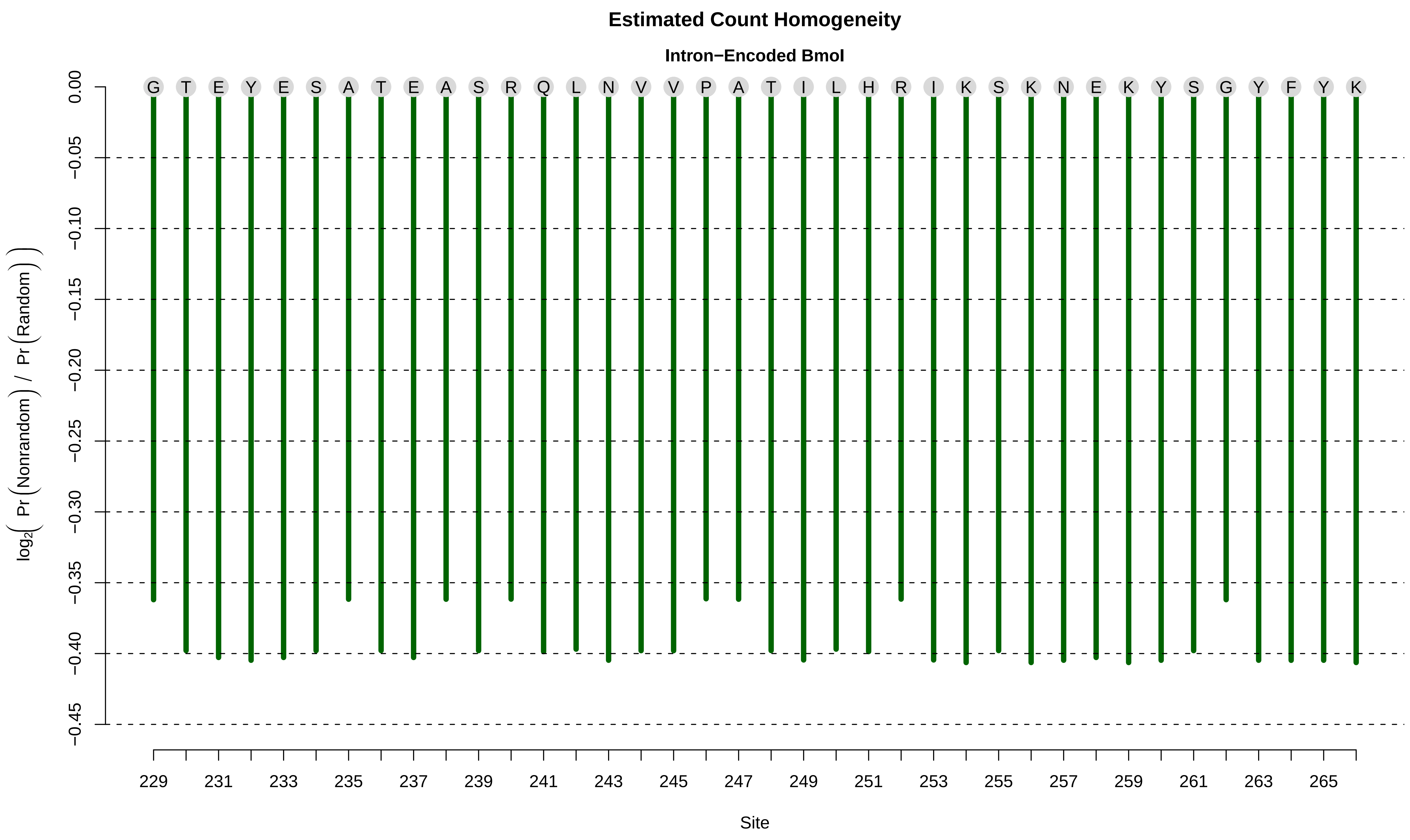
Estimated Count Homogeneity
Intron−Encoded BmoI
G T E Y E S A T E A S R Q L N V V P A T I L H R I K S K N E K Y S G Y F Y K
0.00
−0.05
−0.10
−0.15
−0.20
−0.25
−0.30
−0.35
−0.40
−0.45
log2(Pr(Nonrandom) / Pr(Random))
229
231
233
235
237
239
241
243
245
247
249
251
253
255
257
259
261
263
265
Site
