# Supplementary material for: Estimating the evidence of selection and the reliability of inference in unigenic evolution
Source: Algorithms Mol Biol. 2010 Nov 8;5:35. doi: 10.1186/1748-7188-5-35 (PMC2994857; doi:10.1186/1748-7188-5-35)
Supplement: Additional file 7 — Sample Input and Output. Sample input, output, and driver files for the given software package. [file 1748-7188-5-35-S7.ZIP › unigenic_example/edgell.pol/by_site_ehp.pdf]

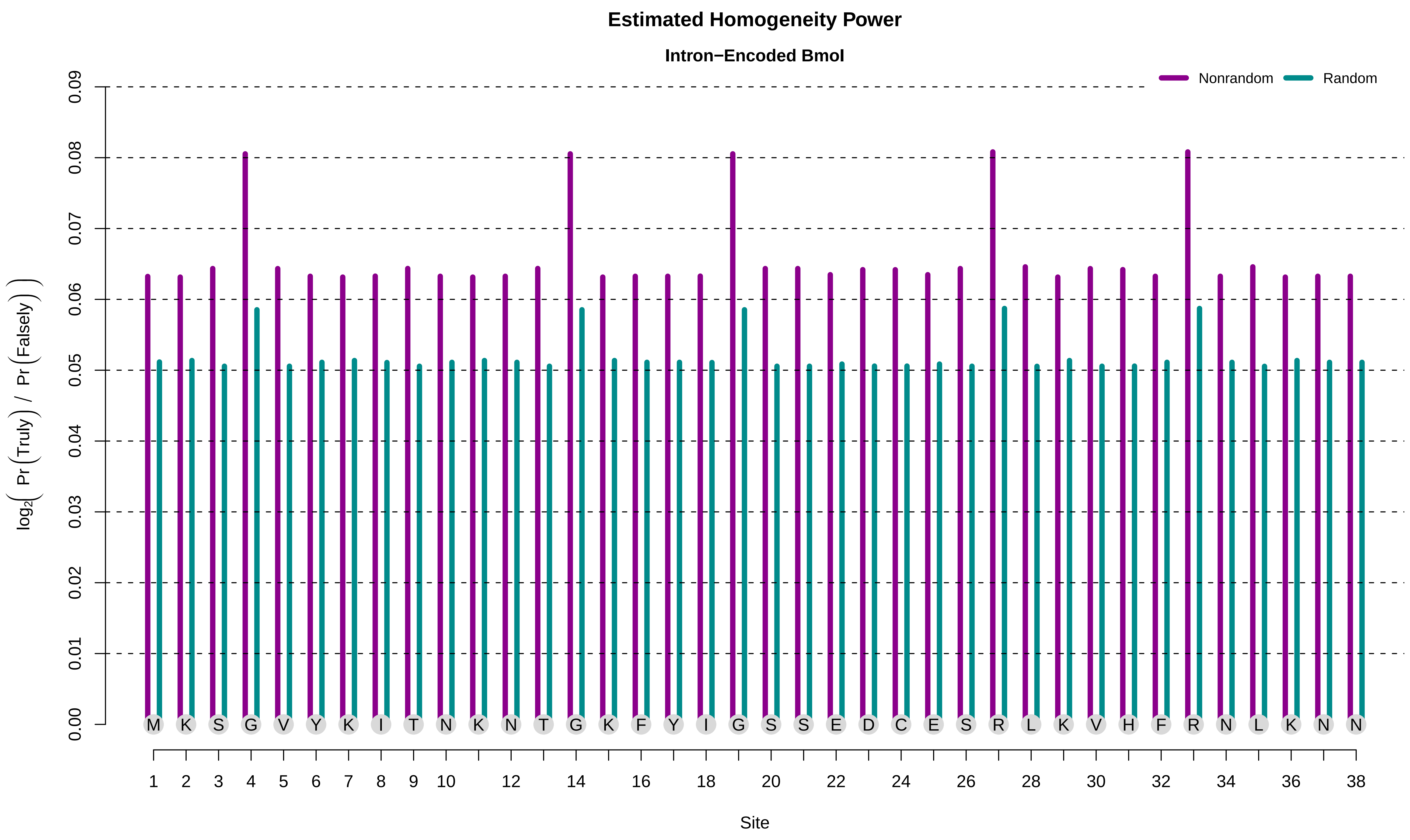

Estimated Homogeneity Power
Intron−Encoded BmoI
Nonrandom
Random
log2(Pr(Truly) / Pr(Falsely))
0.00
0.01
0.02
0.03
0.04
0.05
0.06
0.07
0.08
0.09
M K S G V Y K I T N K N T G K F Y I G S S E D C E S R L K V H F R N L K N N
1 2 3 4 5 6 7 8 9 10 12 14 16 18 20 22 24 26 28 30 32 34 36 38
Site

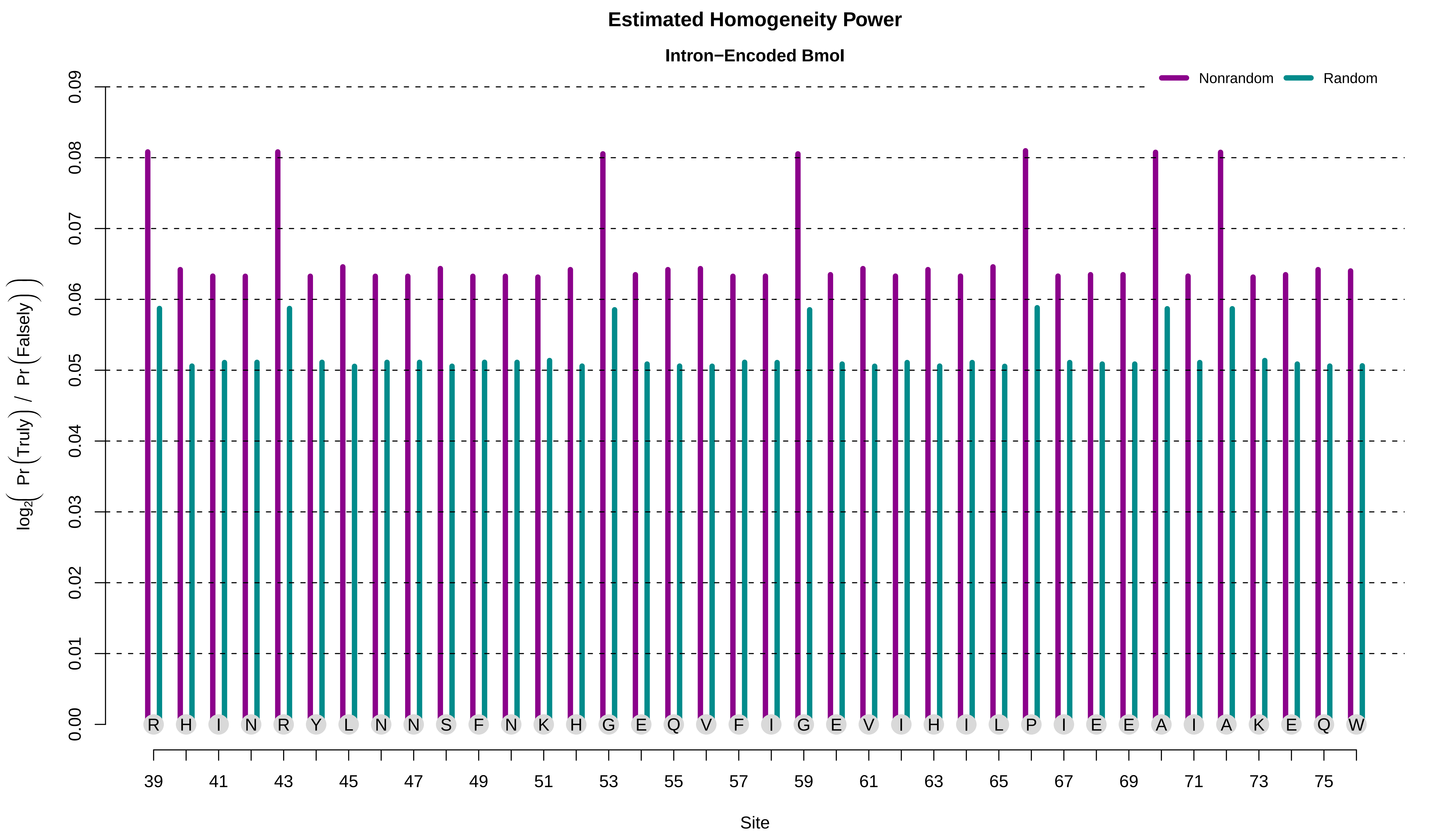

Estimated Homogeneity Power
Intron−Encoded BmoI
Nonrandom
Random
log2(Pr(Truly) / Pr(Falsely))
0.00
0.01
0.02
0.03
0.04
0.05
0.06
0.07
0.08
0.09
R H I N R Y L N N S F N K H G E Q V F I G E V I H I L P I E E A I A K E Q W
39
41
43
45
47
49
51
53
55
57
59
61
63
65
67
69
71
73
75
Site

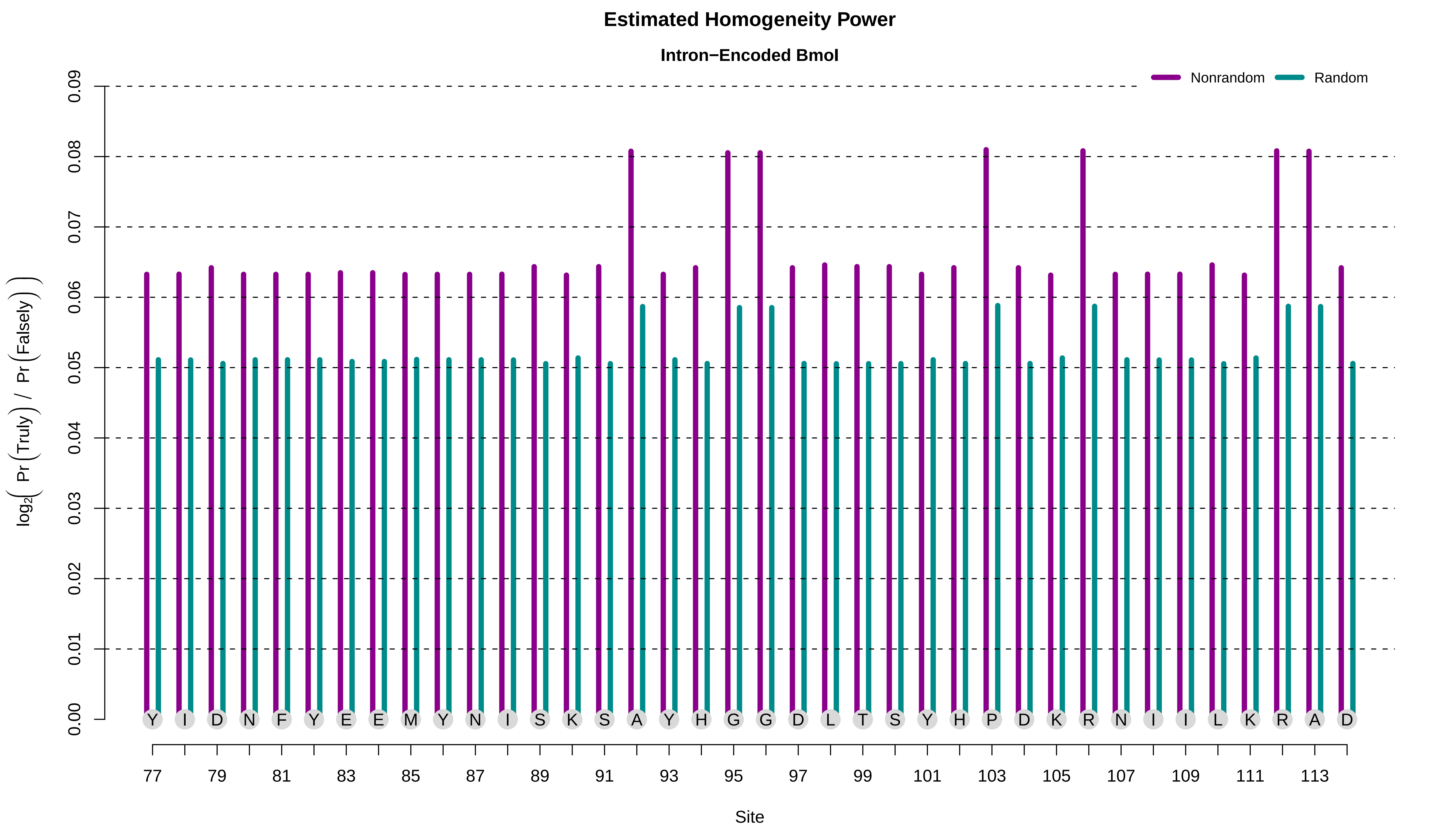

Estimated Homogeneity Power
Intron−Encoded BmoI
Nonrandom
Random
log2(Pr(Truly) / Pr(Falsely))
0.00
0.01
0.02
0.03
0.04
0.05
0.06
0.07
0.08
0.09
Y I D N F Y E E M Y N I S K S A Y H G G D L T S Y H P D K R N I I L K R A D
77
79
81
83
85
87
89
91
93
95
97
99
101
103
105
107
109
111
113
Site

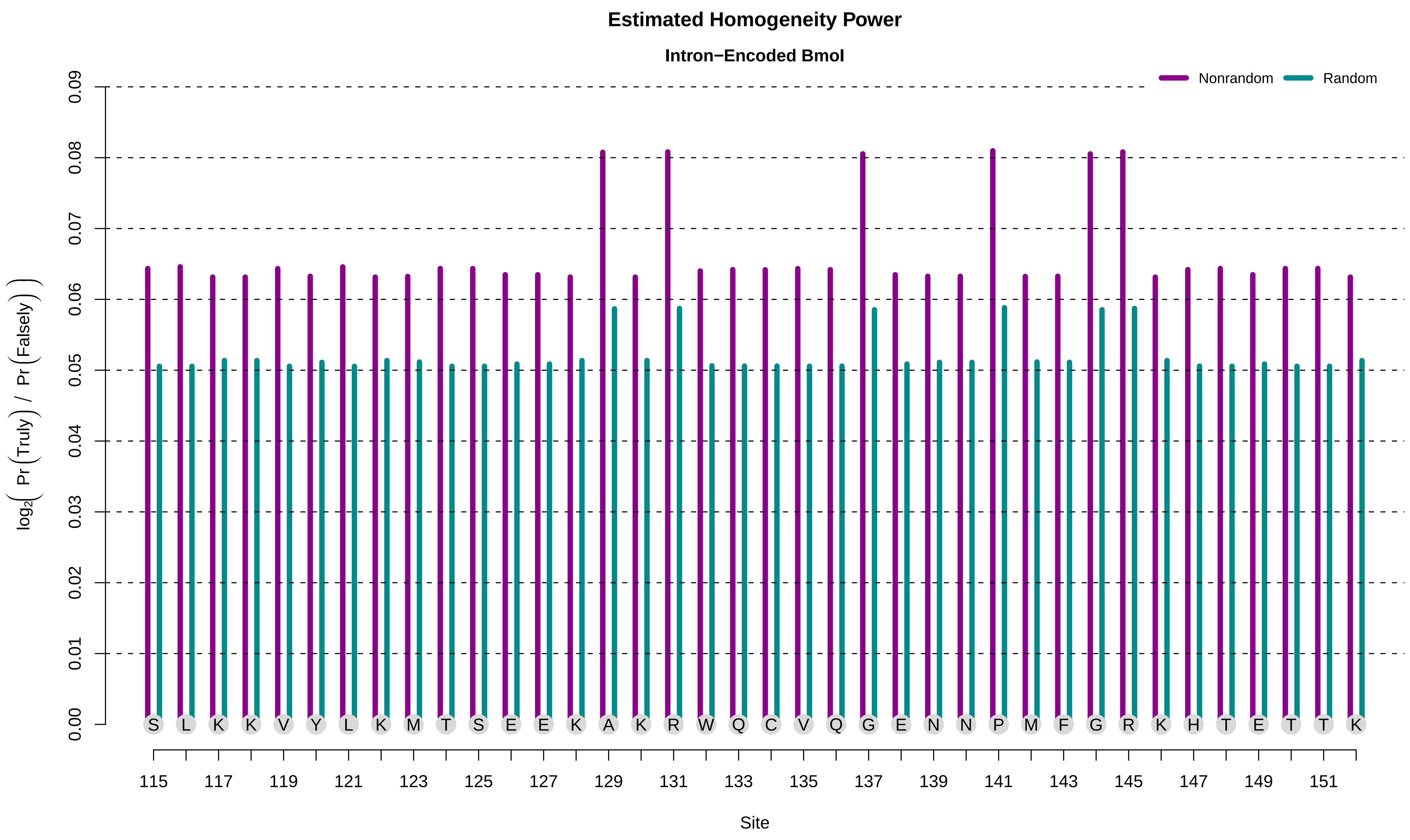

Estimated Homogeneity Power
Intron−Encoded BmoI
Nonrandom
Random
0.00
0.01
0.02
0.03
0.04
0.05
0.06
0.07
0.08
0.09
log2(Pr(Truly) / Pr(Falsely))
S L K K V Y L K M T S E E K A K R W Q C V Q G E N N P M F G R K H T E T T K
115
117
119
121
123
125
127
129
131
133
135
137
139
141
143
145
147
149
151
Site

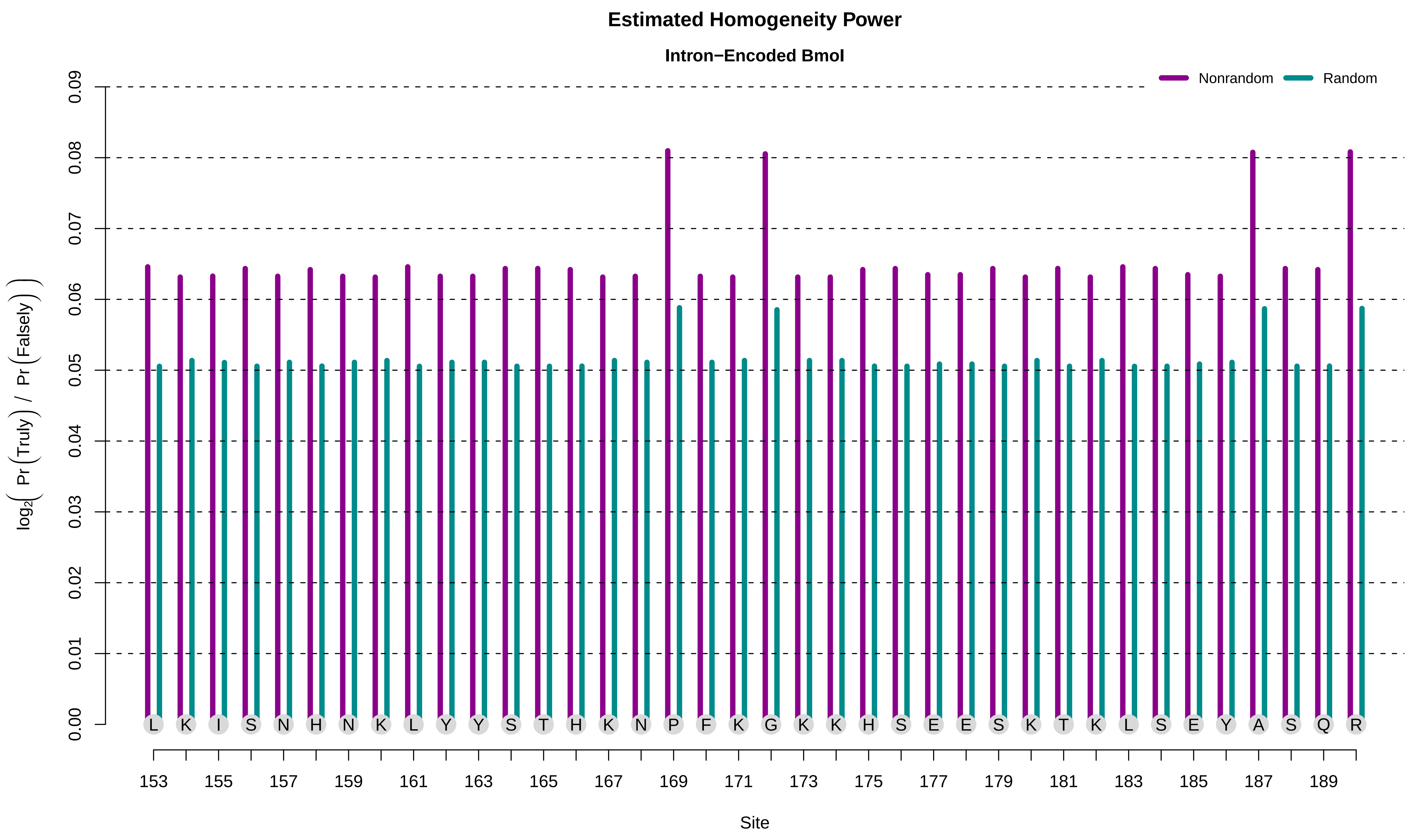

Estimated Homogeneity Power
Intron−Encoded BmoI
Nonrandom
Random
log2(Pr(Truly) / Pr(Falsely))
0.00
0.01
0.02
0.03
0.04
0.05
0.06
0.07
0.08
0.09
L K I S N H N K L Y Y S T H K N P F K G K K H S E E S K T K L S E Y A S Q R
153
155
157
159
161
163
165
167
169
171
173
175
177
179
181
183
185
187
189
Site

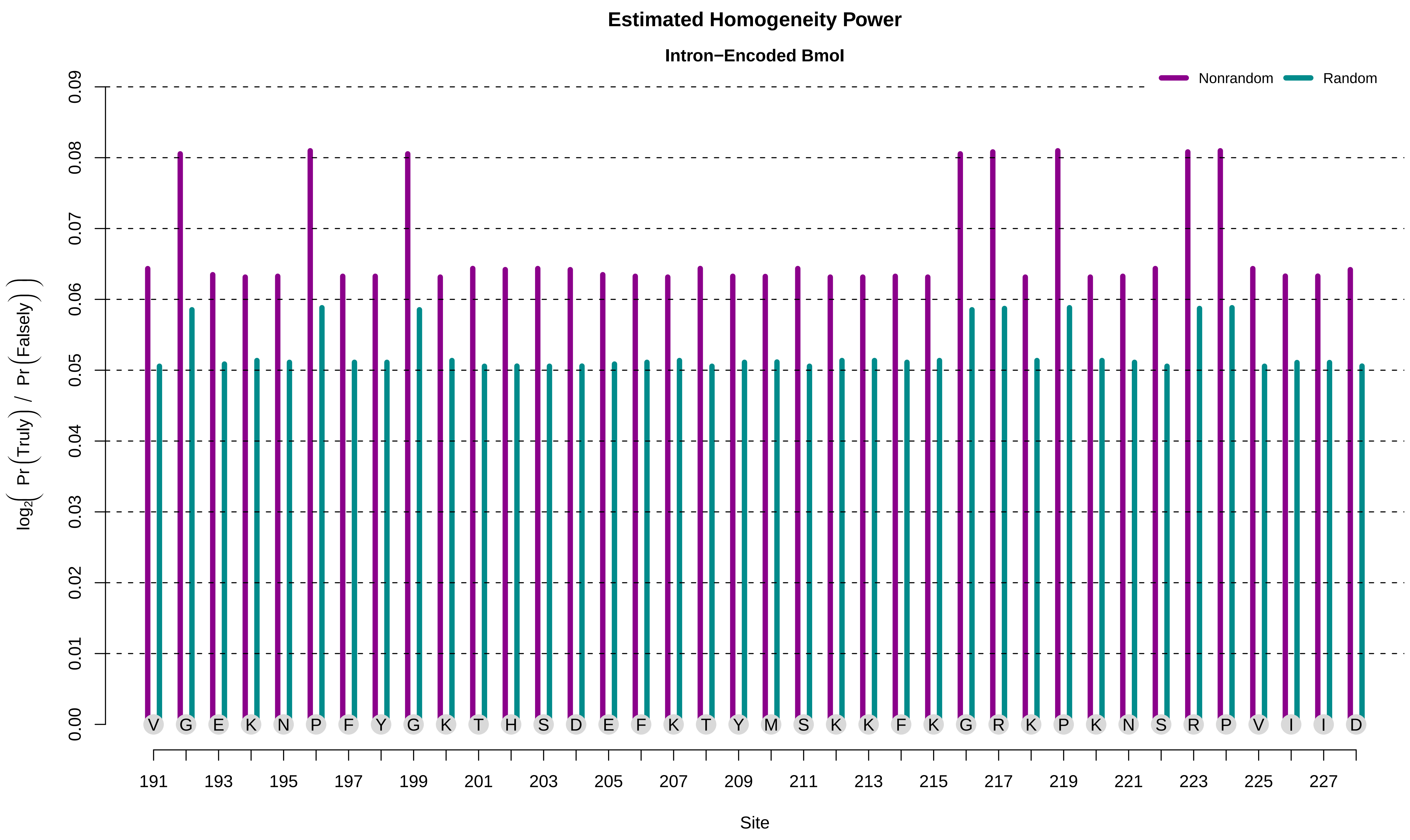

Estimated Homogeneity Power
Intron−Encoded BmoI
Nonrandom
Random
log2(Pr(Truly) / Pr(Falsely))
0.00
0.01
0.02
0.03
0.04
0.05
0.06
0.07
0.08
0.09
V G E K N P F Y G K T H S D E F K T Y M S K K F K G R K P K N S R P V I I D
191
193
195
197
199
201
203
205
207
209
211
213
215
217
219
221
223
225
227
Site

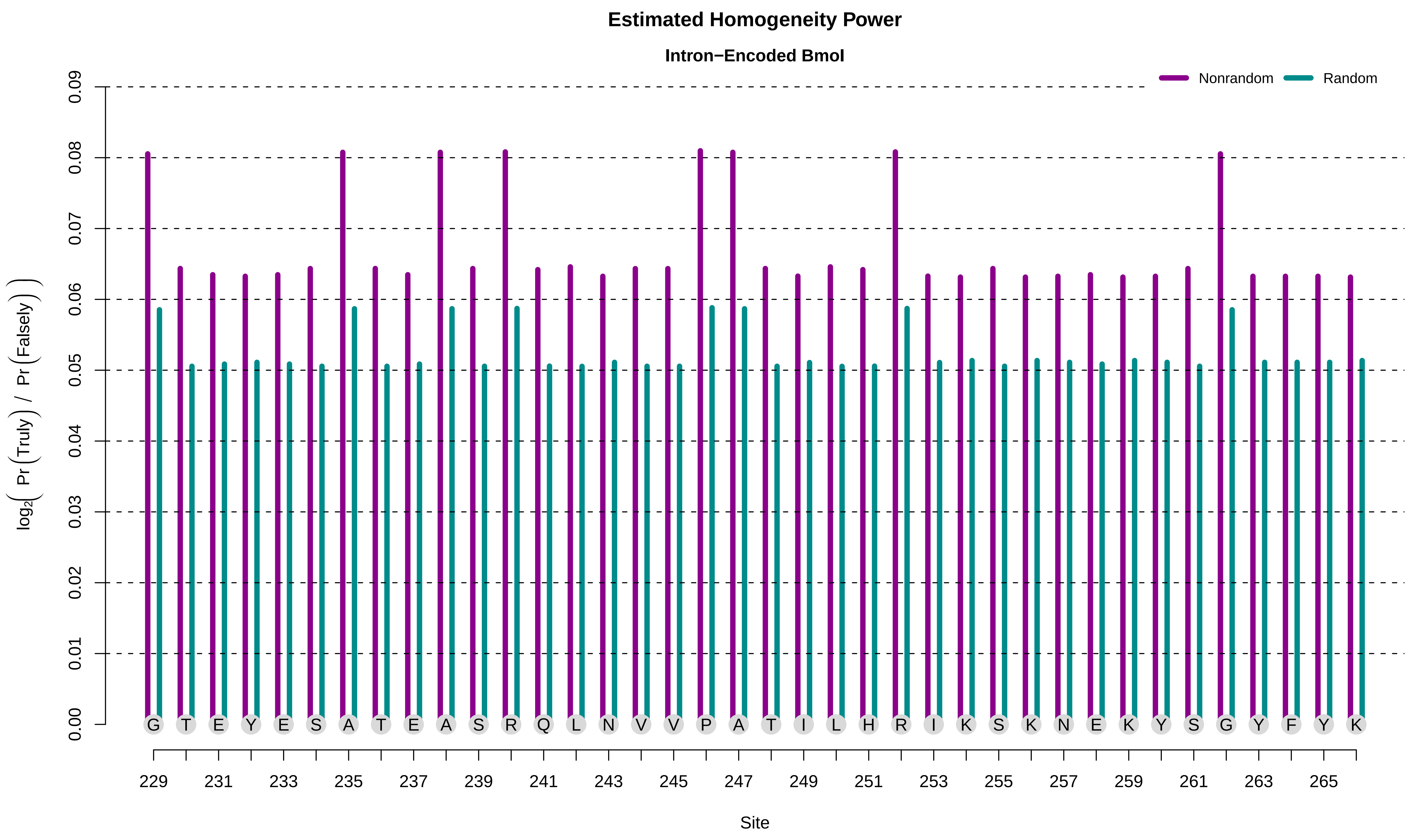
Estimated Homogeneity Power
Intron−Encoded BmoI
Nonrandom
Random
$\log_2\left(\Pr\left(\text{Truly}\right) / \Pr\left(\text{Falsely}\right)\right)$
0.00
0.01
0.02
0.03
0.04
0.05
0.06
0.07
0.08
0.09
G T E Y E S A T E A S R Q L N V V P A T I L H R I K S K N E K Y S G Y F Y K
229
231
233
235
237
239
241
243
245
247
249
251
253
255
257
259
261
263
265
Site
