# Supplementary material for: Estimating the evidence of selection and the reliability of inference in unigenic evolution
Source: Algorithms Mol Biol. 2010 Nov 8;5:35. doi: 10.1186/1748-7188-5-35 (PMC2994857; doi:10.1186/1748-7188-5-35)
Supplement: Additional file 7 — Sample Input and Output. Sample input, output, and driver files for the given software package. [file 1748-7188-5-35-S7.ZIP › unigenic_example/edgell.pol/by_struct.pdf]

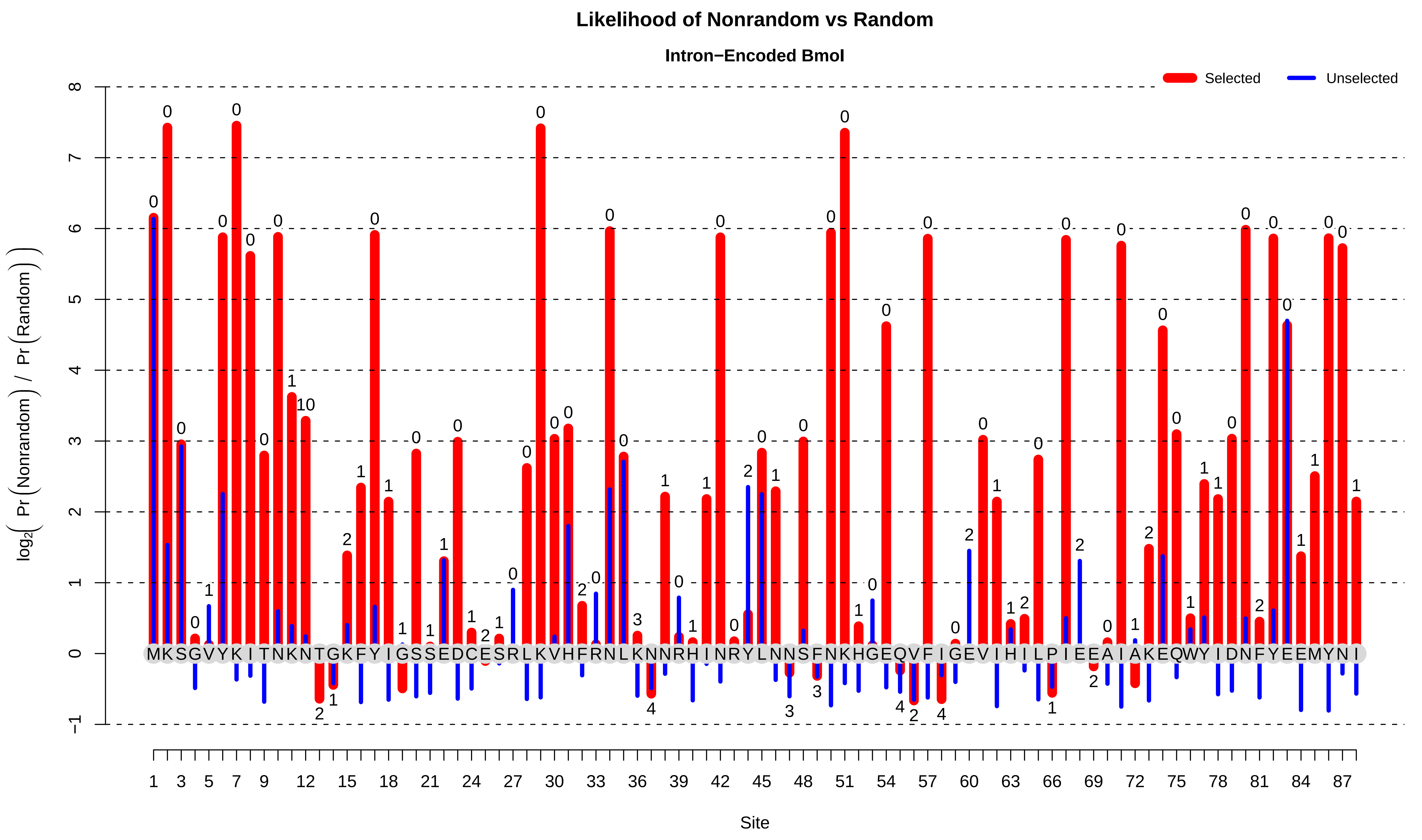

Likelihood of Nonrandom vs Random
Intron−Encoded BmoI
Selected
Unselected
$\log_2\left(Pr\left(Nonrandom\right) / Pr\left(Random\right) \right)$
−1 0 1 2 3 4 5 6 7 8
M K S G V Y K I T N K N T G K F Y I G S S E D C E S R L K V H F R N L K N N R H I N R Y L N N S F N K H G E Q V F I G E V I H I L P I E E A I A K E Q W Y I D N F Y E E M Y N I
1 3 5 7 9 12 15 18 21 24 27 30 33 36 39 42 45 48 51 54 57 60 63 66 69 72 75 78 81 84 87
Site

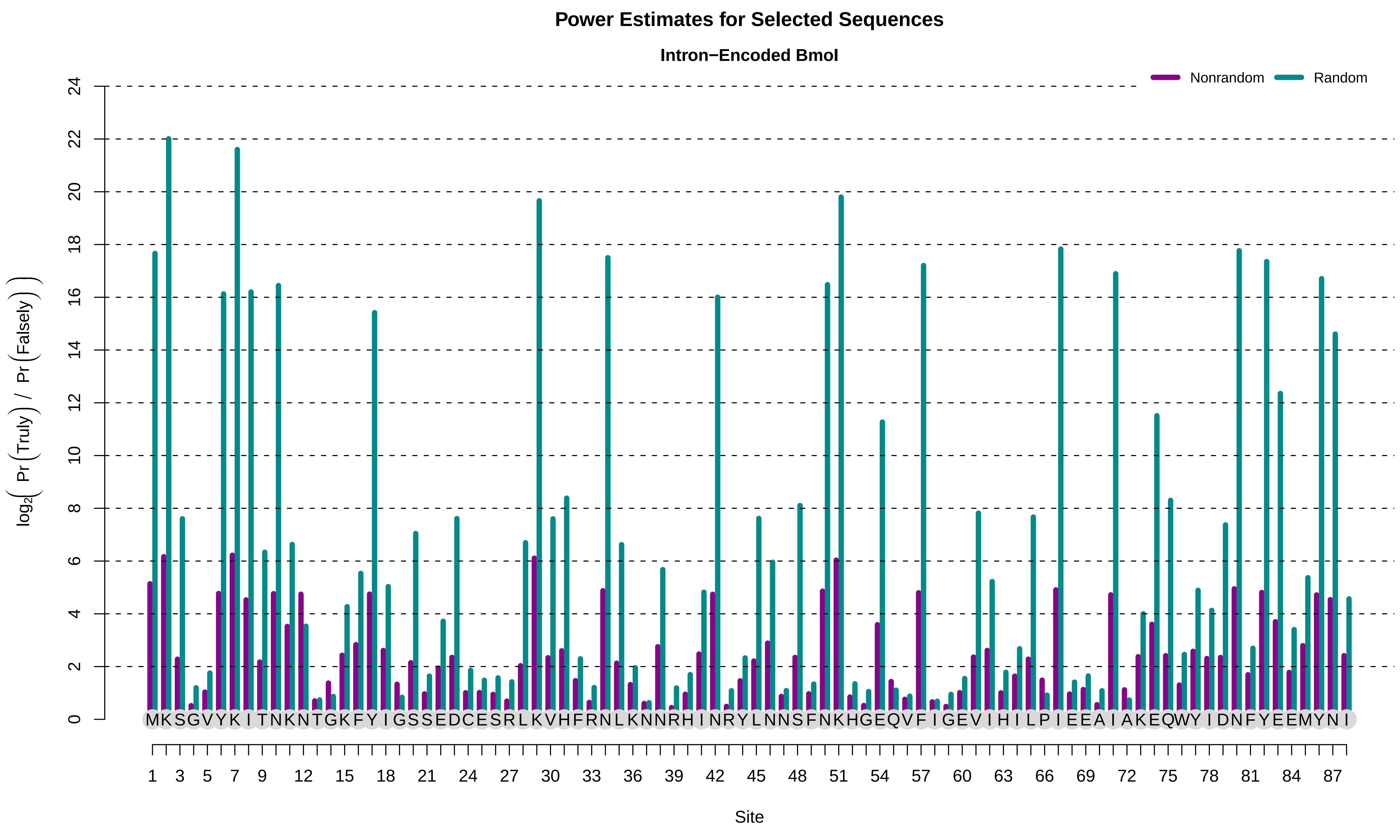
Power Estimates for Selected Sequences
Intron−Encoded BmoI
Nonrandom
Random
log2(Pr(Truly) / Pr(Falsely))
0 2 4 6 8 10 12 14 16 18 20 22 24
M K S G V Y K I T N K N T G K F Y I G S S E D C E S R L K V H F R N L K N N R H I N R Y L N N S F N K H G E Q V F I G E V I H I L P I E E A I A K E Q W Y I D N F Y E E M Y N I
1 3 5 7 9 12 15 18 21 24 27 30 33 36 39 42 45 48 51 54 57 60 63 66 69 72 75 78 81 84 87
Site

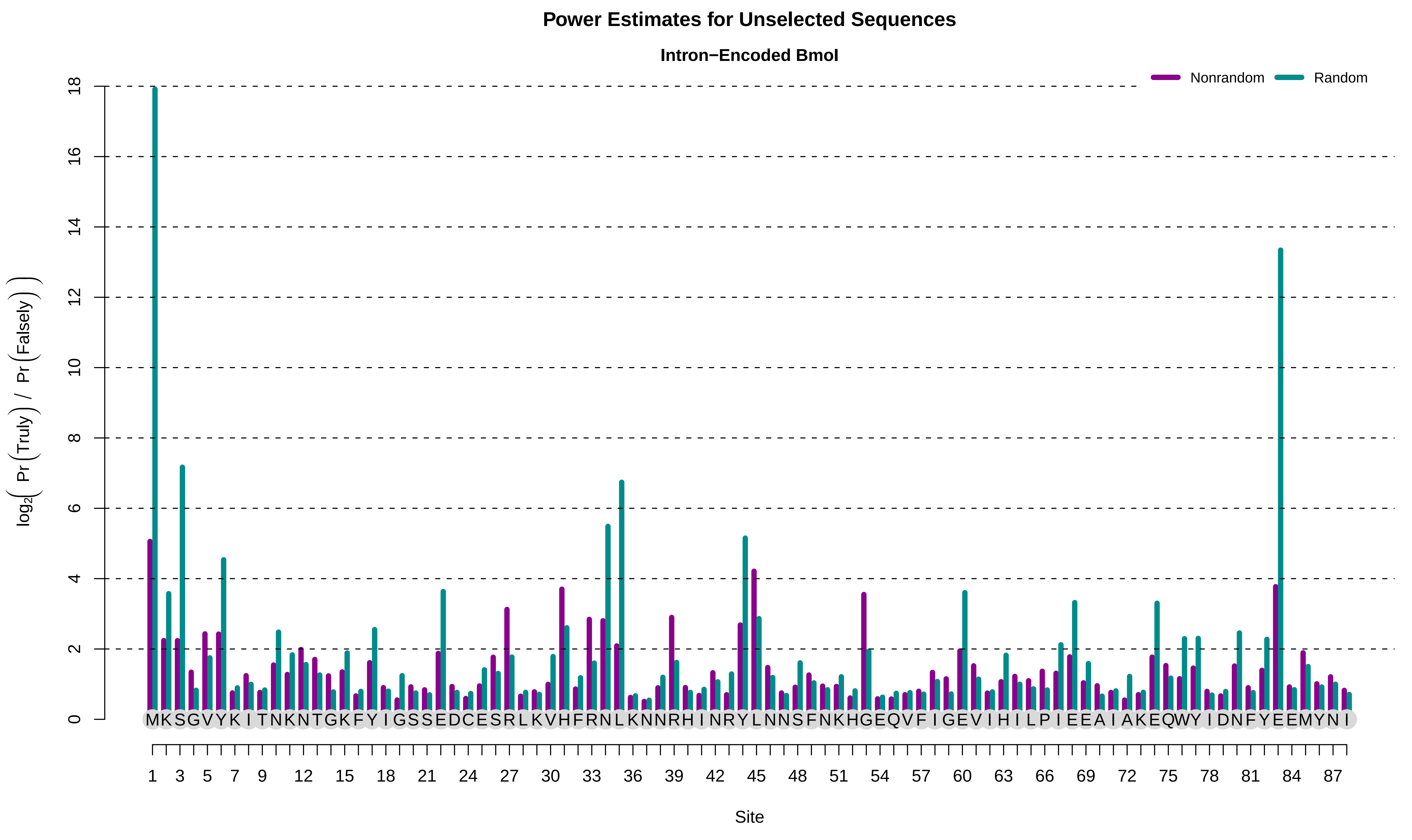

Power Estimates for Unselected Sequences
Intron−Encoded BmoI
Nonrandom
Random
$\log_2\left(\Pr\left(\text{Truly}\right) / \Pr\left(\text{Falsely}\right) \right)$
0
2
4
6
8
10
12
14
16
18
MKSGVYKITNKNTGKFYIGSSEDCESRLKVHFRNLKNNRHINRYLNNSFNKHGEQVFIGEVIHILPIEEAIAKEQWYIDNFYEEMYNI
1 3 5 7 9 12 15 18 21 24 27 30 33 36 39 42 45 48 51 54 57 60 63 66 69 72 75 78 81 84 87
Site

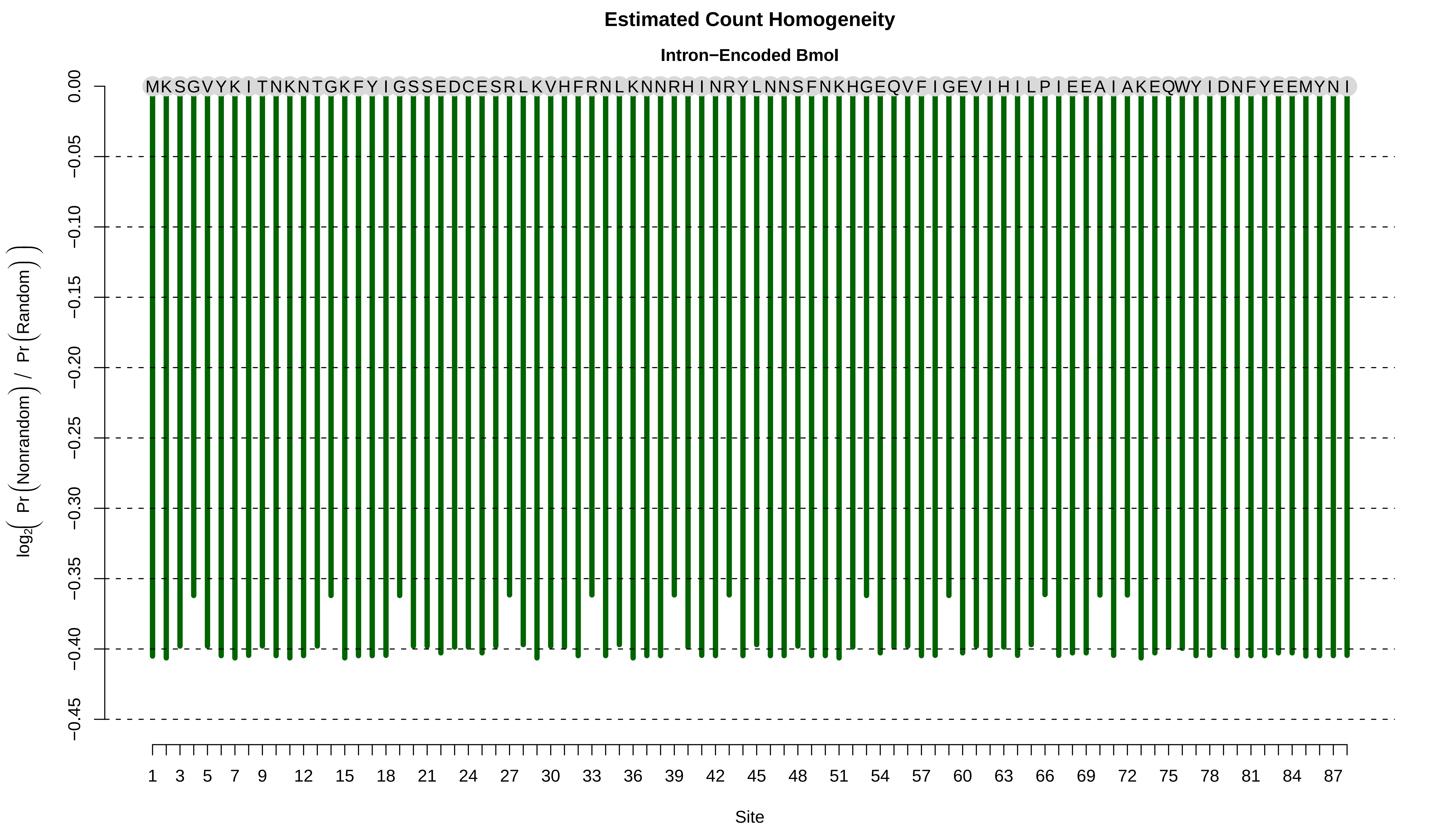

Estimated Count Homogeneity
Intron−Encoded BmoI
M K S G V Y K I T N K N T G K F Y I G S S E D C E S R L K V H F R N L K N N R H I N R Y L N N S F N K H G E Q V F I G E V I H I L P I E E A I A K E Q W Y I D N F Y E E M Y N I
0.00
−0.05
−0.10
−0.15
−0.20
−0.25
−0.30
−0.35
−0.40
−0.45
log2(Pr(Nonrandom) / Pr(Random))
1 3 5 7 9 12 15 18 21 24 27 30 33 36 39 42 45 48 51 54 57 60 63 66 69 72 75 78 81 84 87
Site

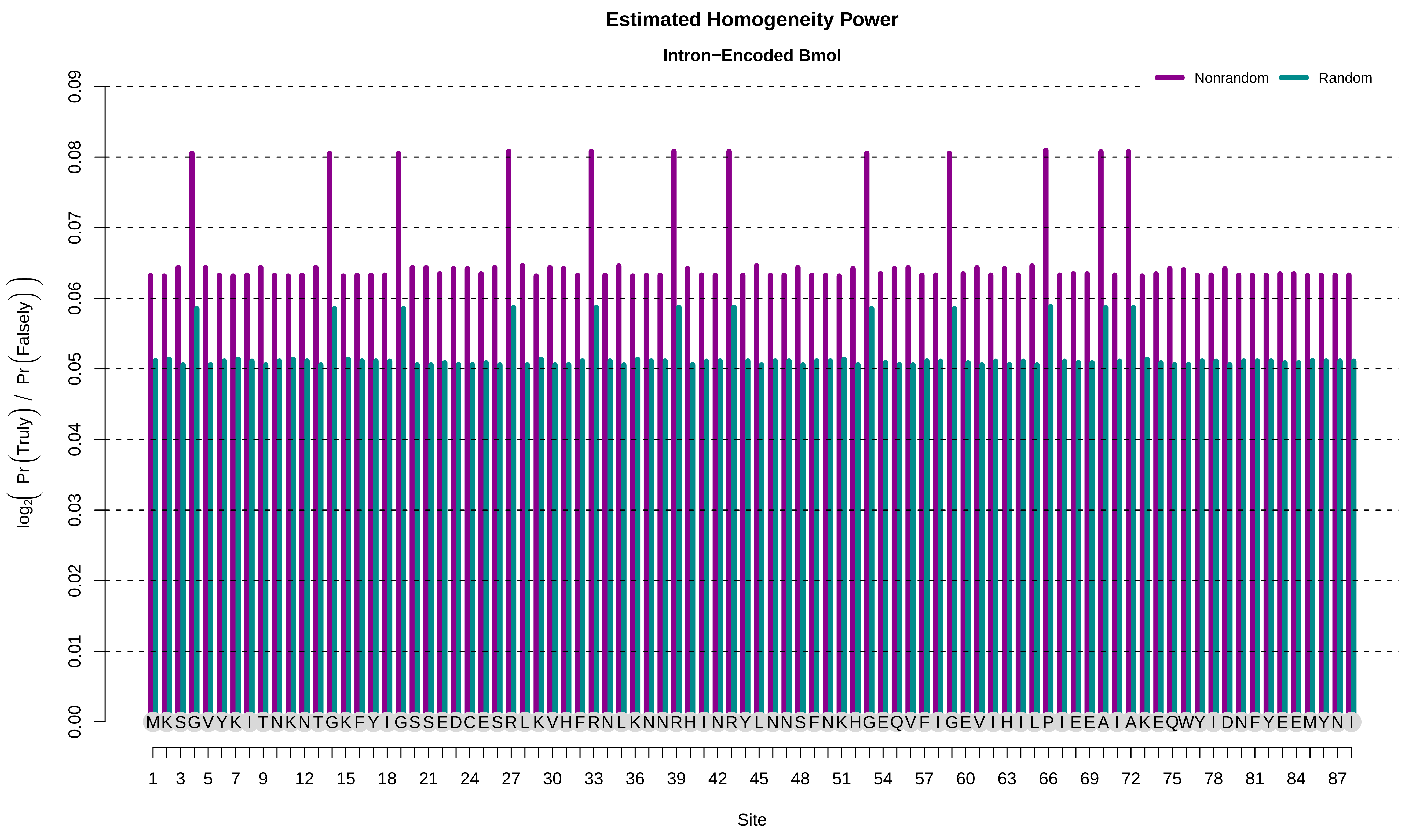

Estimated Homogeneity Power
Intron−Encoded BmoI
Nonrandom
Random
log2(Pr(Truly) / Pr(Falsely))
0.00
0.01
0.02
0.03
0.04
0.05
0.06
0.07
0.08
0.09
MKSGVYKITNKNTGKFYIGSSEDCESRLKVHFRNLKNNRHINRYLNNSFNKHGEQVFIGEVIHILPIEEAIAKEQWYIDNFYEEMYNI
1 3 5 7 9 12 15 18 21 24 27 30 33 36 39 42 45 48 51 54 57 60 63 66 69 72 75 78 81 84 87
Site
